# The role of flipped classroom models and web-based instruction in developing collaborative learning attitudes and learning strategies

**DOI:** 10.3389/fpsyg.2026.1792842

**Published:** 2026-06-30

**Authors:** Gizem Tabaru Örnek, Nihal Yildiz, Mustafa Erol

**Affiliations:** 1 Department of Primary Education, Faculty of Education, Karamanoglu Mehmetbey University, Karaman, Türkiye; 2 Department of Primary Education, Faculty of Education, Yildiz Teknik University, İstanbul, Türkiye

**Keywords:** cooperative learning attitude, flipped classroom, learning strategy, pre service teacher, web-based instruction

## Abstract

**Introduction:**

This study aims to compare and analyze the effects of the flipped classroom model and web-based instruction on pre-service classroom teachers' attitudes toward cooperative learning and their learning strategies in the Life Science Teaching course.

**Methods:**

The study employed a quasi-experimental pretest-posttest control-group design. A total of 74 third-year pre-service classroom teachers participated in the study. The implementation lasted 6 weeks and included 18 hours of instruction. Data were analyzed by comparing the experimental and control groups in terms of cooperative learning attitudes and learning strategies.

**Results:**

The findings revealed that instruction based on the flipped classroom model was more effective than web-based instruction in improving pre-service classroom teachers' attitudes toward cooperative learning. In addition, both the flipped classroom model and web-based instruction were significantly more effective than the traditional teaching method used in the control group in developing students' learning strategies.

**Discussion:**

The results suggest that technology-supported instructional approaches, particularly the flipped classroom model, can contribute positively to cooperative learning attitudes and learning strategies in teacher education. These findings highlight the importance of integrating innovative and student-centered instructional models into teacher training programs.

## 1 Introduction

Technological innovations, which are rapidly advancing globally, constitute a cornerstone of societal development and transformation. In particular, developing countries cannot afford to remain indifferent to technology if they are to compete and maintain a presence in the global economy (Jhurree, 2005). The United Nations General Assembly has emphasized the importance of digitalization, noting that the spread of information and communication technologies, along with the growing interconnectedness of societies, holds great potential to accelerate human progress, bridge the digital divide, and foster knowledge societies (United Nation, 2015). As the world becomes increasingly digital, the field of education has diversified through digital games, applications, websites, social media, and online learning environments. School closures during the COVID-19 pandemic, due to social distancing measures, accelerated this digital transformation on a global scale (Decuypere et al., 2021; Aivazidi and Michalakelis, 2021). However, the digitalization of higher education systems had already been recognized prior to the pandemic as an effective tool to support students' learning processes (Wekerle et al., 2022).

In the 21st century, both teachers and students are expected to design innovative learning tasks that utilize digital tools tailored to their specific needs (Velcheva and Garov, 2021). The effective use of technology by teachers is only possible through its integration into educational practices. However, research has consistently shown that novice teachers make limited use of technology in their educational practices (Bate, 2010; Dawson, 2008). This situation has prompted teacher education institutions worldwide to revise their curricula to better prepare future teachers for the effective integration of technology (Tondeur et al., 2013). It is now widely acknowledged that computer-based educational technologies are most effective when students use them extensively. Such technologies offer a range of powerful learning experiences, from communication and collaboration with others to searching and accessing information, engaging with simulations of otherwise unobservable phenomena, and even participating in educational games (Bernard et al., 2023).

The increasing presence of digital technologies in educational contexts not only transforms teaching processes but also influences the development and application of learning strategies. Learning strategies can be understood as the behaviors and thought processes in which students engage. Their purpose is to help learners integrate new knowledge with prior knowledge, identify cognitive gaps, and regulate both their motivational and emotional states during Learning (Dinsmore et al., 2020; Kikas et al., 2024). In this regard, learning strategies offer a novel approach to “learning how to learn” (Nisbet and Shucksmith, 2017), grounded in the philosophy of how Learning can occur most effectively (Hattie and Donoghue, 2016).

As a critical component of academic success, learning strategies encompass a range of techniques, from underlining or taking notes during lectures to summarizing and organizing new knowledge, and rephrasing learned material in one's own words (Dumford et al., 2016). Given that cognitive structures evolve, the quality and modes of learning strategy use vary across the educational trajectory, from early childhood to university (Brod, 2021). Age-related differences also shape how individuals apply these strategies in different learning environments. Research has shown that online learning applications both support and affect the use of learning strategies (Chen et al., 2023; Küçük, 2012; Zhao et al., 2025). Within the Context of digital education, online Learning has emerged as a primary means of achieving adequate progress in higher education (Wang et al., 2023). Recently, the flipped classroom model has been identified as an important trend in online learning environments. Similarly, this study focuses on the impact of digital content used in an undergraduate course on students' learning strategies. Designing activities using web tools and the flipped model diversifies learning strategies and supports the learning process by engaging multiple senses.

The flipped classroom model has emerged as an innovative approach closely associated with online learning environments, particularly by supporting pre-class preparation through digital video content. However, the theoretical foundations of the model are rooted in a long-standing pedagogical tradition and are not limited solely to digital or online modes of delivery. Indeed, this approach draws on the shift in the teacher's role from the “sage on the stage” to the “guide on the side” (King, 1993) and on active, student-centered learning paradigms, aiming to allocate classroom time to higher-order thinking, discussion, and collaborative activities (Lage et al., 2000; Bergmann and Sams, 2012). More recently, integrating the model with web-based tools has enabled the design of learning activities in more flexible, interactive ways, diversifying learning strategies and providing enriched experiences that engage multiple senses.

The diversification of methods and techniques in educational processes is crucial to establishing effective learning environments. When supported by technology, this diversity not only increases students' engagement with the course but also enhances their motivation (Richardson et al., 2017). Among technology-enhanced applications, web tools stand out due to their abundance and flexibility in preparation, implementation, and assessment. Beyond curricular requirements, Web 2.0 tools, which learners of all ages and levels widely use, offer practical means for self-expression, networking, knowledge acquisition, sharing, and identity construction (Collis and Moonen, 2008). Findings from this study contribute to educators' understanding of how integrating web tools and the flipped classroom model can better guide students' learning processes, strengthen their attitudes toward collaborative learning (CL), and enhance their learning skills. Consequently, this research sought to determine which type of learning environment—supported by web tools and the flipped classroom model—yields more advanced learning outcomes in terms of learning strategies and CL attitudes.

### 1.1 Conceptual framework

#### 1.1.1 Flipped classroom model

The flipped classroom model, often referred to as “flipped learning,” “inverted teaching,” or “blended learning,” represents an innovative pedagogical approach enabled by the proliferation of online resources and the flexibility of video-based materials (Bergmann and Sams, 2012). Unlike traditional classroom settings (del Arco et al., 2022; Sun et al., 2022; Ma et al., 2025), in this model, students engage with course content before class through digital resources (most frequently videos) (Zhang et al., 2024). At the same time, in-class time is reserved for more interactive activities such as assignments, applications, and discussions (Strayer, 2007). In this way, lower-order cognitive tasks are completed before class, whereas higher-order thinking skills are supported during class sessions (Oudbier et al., 2022).

This approach enables students to learn at their own pace, making classroom time more efficient. Ayrica bu yaklaşim ögrenciler arasinda etkileşimi ve iletişimi teşvik ederken öğrencilerin bağimsiz öğrenme yeteneklerini de geliştirir (Shi et al., 2025). Teachers can observe students' difficulties during in-class activities and develop strategies tailored to different learning styles (Bishop and Verleger, 2013; Herreid and Schiller, 2013; Van Alten et al., 2019; Mandasari and Wahyudin, 2021; Galindo-Dominguez, 2021; Vitta and Al-Hoorie, 2023). Nevertheless, challenges such as the need for pre-class preparation, increased teacher workload, and potential student resistance have been documented (Hwang et al., 2015). The flipped classroom model ultimately seeks to enhance students' active participation, personalize the learning process, and dedicate class time to CL and problem-solving activities (Fulton, 2012). Previous research has demonstrated that this model enhances students' motivation, improves higher-order cognitive skills, and fosters CL processes (Long et al., 2017; Baig and Yadegaridehkordi, 2023; Güler et al., 2023).

Although the relationship between the flipped classroom and CL has been widely examined (e.g., Chandra, 2015; Qureshi et al., 2021; Jovanović and Milosavljević, 2022; Liu et al., 2024), studies that focus specifically on CL attitudes remain limited. Similarly, research on the model's connection with learning strategies has primarily relied on interviews and document analyses (Roehl et al., 2013; Hwang et al., 2015; Jovanović et al., 2017; Qu and Miao, 2021; Hava, 2021). Therefore, conducting experimental studies is crucial for clarifying the impact of the flipped classroom on CL attitudes and learning strategies. Moreover, earlier findings suggest that students' ability to adapt to new learning situations and modify their strategies remains limited (Lust et al., 2013).

#### 1.1.2 Web-based instruction

Web tools have become a core element of contemporary technology due to their ability to cater to individuals of different age groups and diverse interests. With the growing use of the internet, these tools enable teachers to develop methods that better align with students' learning preferences (Robles, 2013; Hu et al., 2022). Web-based learning environments provide opportunities for personalized learning, facilitate interaction between authentic and digital resources, and enable independent learning regardless of location (Aljawarneh, 2020). Computer-Supported Collaborative Learning (CSCL), as a technology-enhanced extension of collaborative learning (CL), facilitates students' processes of shared knowledge construction, discussion, and meaning-making through computer- and internet-based tools (Stahl et al., 2006; Dillenbourg, 1999). Unlike simple group work, CSCL systematically structures positive interdependence, individual accountability, and supportive interaction through technology, thereby strengthening information sharing and collective cognitive processes. In this context, web-based tools directly support CSCL practices by providing rich learning environments for interaction and collaboration, contributing to the overall effectiveness of the learning process (Millis, 2010; Biasutti, 2011). CL refers to a process in which individuals aim to improve both their own achievement and that of their peers (Veenman et al., 2000).

The literature highlights that online collaborative environments have a positive influence on students' perceptions and attitudes (Johnson et al., 2007; So and Brush, 2008). CL is widely recognized as an effective method across all educational levels (Johnson et al., 2007) and has been particularly linked to student achievement in higher education (Loh and Ang, 2020). These outcomes have also been reflected in the higher education policies of many countries (Sidgi, 2022). However, despite the extensive literature on web tools and collaborative approaches, studies focusing directly on CL attitudes remain scarce.

Web tools also enable students to integrate schoolwork with online activities, thereby maintaining educational continuity during extraordinary circumstances, such as school closures (Avanzini et al., 2021; Aivazidi and Michalakelis, 2021). In the 21st century, teacher roles have been redefined in response to the demands of digital education. Teachers are now expected to utilize a range of versatile tools effectively, including cloud storage, interactive presentations, web content development, digital notebooks and whiteboards, information-sharing platforms, and assessment tools (Velcheva and Garov, 2021; Rubio and Conesa, 2022). Despite these benefits, the access issues faced by disadvantaged groups remain a limitation of web-based education (Brusilovsky et al., 1998).

#### 1.1.3 Collaborative learning (CL)

Establishing social relationships and belonging to a group are inherent aspects of human life from early childhood (Frania and Correia, 2022). Supporting these characteristics within educational environments has brought cooperative learning approaches to the forefront. Cooperative learning refers to a structured form of group learning in which students work together toward shared goals. In this process, students maintain their individual contributions to the group while simultaneously collaborating with other group members to achieve a common objective (Chang et al., 2022; Erol and Köksal, 2025). One of the core strategies of collaborative learning (CL) is that students take an active role while teachers act as facilitators (Johnson and Johnson, 2008). Rooted in Dewey's (1943) idea that Learning develops in social contexts, this approach was largely overlooked between the 1940s and 1970s but has since gained prominence in education (Johnson and Johnson, 2009). CL involves students working in small groups on tasks or projects according to specific criteria (Felder and Brent, 2007; Li and Lam, 2013; Sulistio and Haryanti, 2022).

This approach incorporates elements such as positive interdependence, individual accountability, face-to-face interaction, the use of collaborative skills, and group process evaluation—features that distinguish it from simple group work (Sharan, 2018). CL fosters more profound, lasting learning by enabling students to construct knowledge collectively (Silalahi and Hutauruk, 2020). Research has demonstrated that this approach positively impacts student achievement, attitudes, and motivation (Kyndt et al., 2013). Recent meta-analyses have demonstrated that cooperative learning has a moderate positive effect on academic achievement, higher-order thinking skills, and affective characteristics (Güngör et al., 2026; Kyndt et al., 2013). Moreover, integrating cooperative learning with the flipped classroom model further enriches the learning process, particularly through the use of Web 2.0 tools and online collaborative platforms. This integration contributes not only to students' academic achievement but also to the development of 21st-century skills, such as digital literacy and techno-pedagogical competence (Aslan, 2022; Erbil, 2020). Furthermore, integrating digital technologies into education enhances opportunities for CL and facilitates students' access to knowledge (Alenezi et al., 2023), thereby supporting active learning and reinforcing learning retention. On the other hand, (Rakha 2023) reported that integrating certain web-based tools into instruction does not necessarily lead to the achievement of intended educational outcomes. The author emphasized the need to explore ways of incorporating active learning strategies, such as cooperative learning, to enhance student engagement in distance education. In this context, the present study aimed to contribute to the field by comparing the flipped classroom model with instruction supported by web-based tools.

#### 1.1.4 Learning strategies (LS)

Learning strategies (LS) encompass a broad concept, referring to a set of competencies considered essential for effective learning and the long-term retention of knowledge (Weinstein and Underwood, 2014). Broadly defined, they are behaviors intended to shape the way students process information (Meyer, 1988). The primary purpose of these strategies is to select the most suitable methods to achieve learning goals and to structure the learning process effectively, taking into account learners' individual characteristics, experiences, and needs (Qu and Miao, 2021). Learning strategies are typically classified into several categories. Rehearsal strategies (e.g., copying, underlining), elaboration strategies (e.g., summarizing, elaborating), and organizational strategies (e.g., outlining, creating hierarchies) are among the most common. In addition, this framework includes metacognitive strategies, which involve monitoring the learning process and recognizing comprehension failures, as well as affective strategies, which aim to foster appropriate emotional states during learning (O'Malley and Chamot, 1990).

The concept of “learning to learn” encompasses planning one's learning performance, identifying difficulties, monitoring and evaluating progress, and adapting strategies when necessary. However, in school contexts, strategies are often taught in task-based ways, and students frequently struggle to apply them in broader contexts (Nisbet and Shucksmith, 2017). Therefore, the goal of learning strategies is not merely to acquire knowledge but also to organize it, integrate it with prior Learning, and continuously evaluate the learning process (Hattie and Donoghue, 2016). Research has demonstrated that effective learning strategies play a crucial role in long-term learning outcomes (Anthonysamy et al., 2020). When educators encourage the use of such strategies, students tend to apply them more extensively and effectively (Dumford et al., 2016). Consequently, developing methods that promote the use of learning strategies across disciplines is critical for creating high-quality learning environments (Ghalebi et al., 2020).

### 1.2 Current study

Research on the flipped classroom model has consistently indicated that, compared with traditional instruction, this approach may enhance students' motivation, active participation, problem-solving skills, and higher-order learning outcomes. Previous studies have suggested that the flipped learning model can support knowledge acquisition, knowledge application, metacognitive monitoring, self-regulated learning strategies, and student engagement (Bishop and Verleger, 2013; Foldnes, 2016; Galindo-Dominguez, 2021; Hava, 2021; Diningrat et al., 2024). Similarly, the growing body of literature on web-based learning has demonstrated that digital environments can foster interaction, engagement, satisfaction, motivation, and collaborative participation. Studies in the literature have reported positive attitudes toward online collaborative learning, significant relationships between the quality of digital interaction and perceived learning gains, and the contribution of communication and online collaboration tools, group work designs, and help-seeking supports to the improvement of learning processes and outcomes (Frania and Correia, 2022; Wekerle et al., 2022; Rakha, 2023; Ateş and Köroglu, 2024; Bach and Thiel, 2024; Cheng, 2025). Taken together, these studies suggest that the effectiveness of web-based learning depends not only on access to digital tools but also on the pedagogical design of interaction, guidance, and strategic support embedded within the learning environment.

Although studies examining attitudes toward cooperative learning and learning strategies among students from different age groups and educational levels have been conducted (Chatterjee and Correia, 2019; Diningrat et al., 2024; Kikas et al., 2024; Pürbudak, 2020), comparative experimental evidence in teacher education—particularly studies that address these two outcomes simultaneously within the same instructional context—remains limited. In this respect, cooperative learning attitudes and learning strategies should not be viewed merely as ancillary skills for teacher candidates. Rather, they are closely related to how future teachers participate in knowledge construction, regulate their own learning, and subsequently design interaction-rich classroom environments for their students. Therefore, strengthening teacher candidates' experiences in collaborative, strategically organized learning environments may have both immediate academic value and long-term pedagogical significance.

Within this framework, the present study aimed to compare the effects of flipped classroom-based activities and web-based instructional activities implemented in the Life Studies Teaching course on teacher candidates' attitudes toward cooperative learning and their learning strategies. To achieve this aim, participants were assigned to three groups: intervention group 1 received instruction through flipped classroom practices; intervention group 2 participated in web-based instructional activities supported by digital tools; and the control group followed the course through traditional instructional practices aligned with the existing learning outcomes. By comparing these three conditions within the same course context, the study sought not only to examine whether technology-supported instruction differs from traditional teaching, but also to clarify whether two distinct forms of technology-enhanced pedagogy—flipped learning and web-based instruction—differ in the extent to which they promote cooperative learning attitudes and learning strategie**s**. In line with this purpose, the following sub-problems were formulated:

1. Is there a significant difference between the pre-test and post-test scores of CL attitudes and LS among the groups instructed through the flipped classroom model, web tools, and the control group?
2. Is there a significant difference between the pre-test and post-test scores of CL attitudes and LS in the experimental groups?
3. Is there a significant difference between the pre-test and post-test scores of CL attitudes and LS in the control group?
4. Do the post-test scores of CL attitudes and LS in the experimental groups differ significantly when pre-test scores are controlled?

#### 1.2.1 Conceptual map of the study

**Figure 1:**
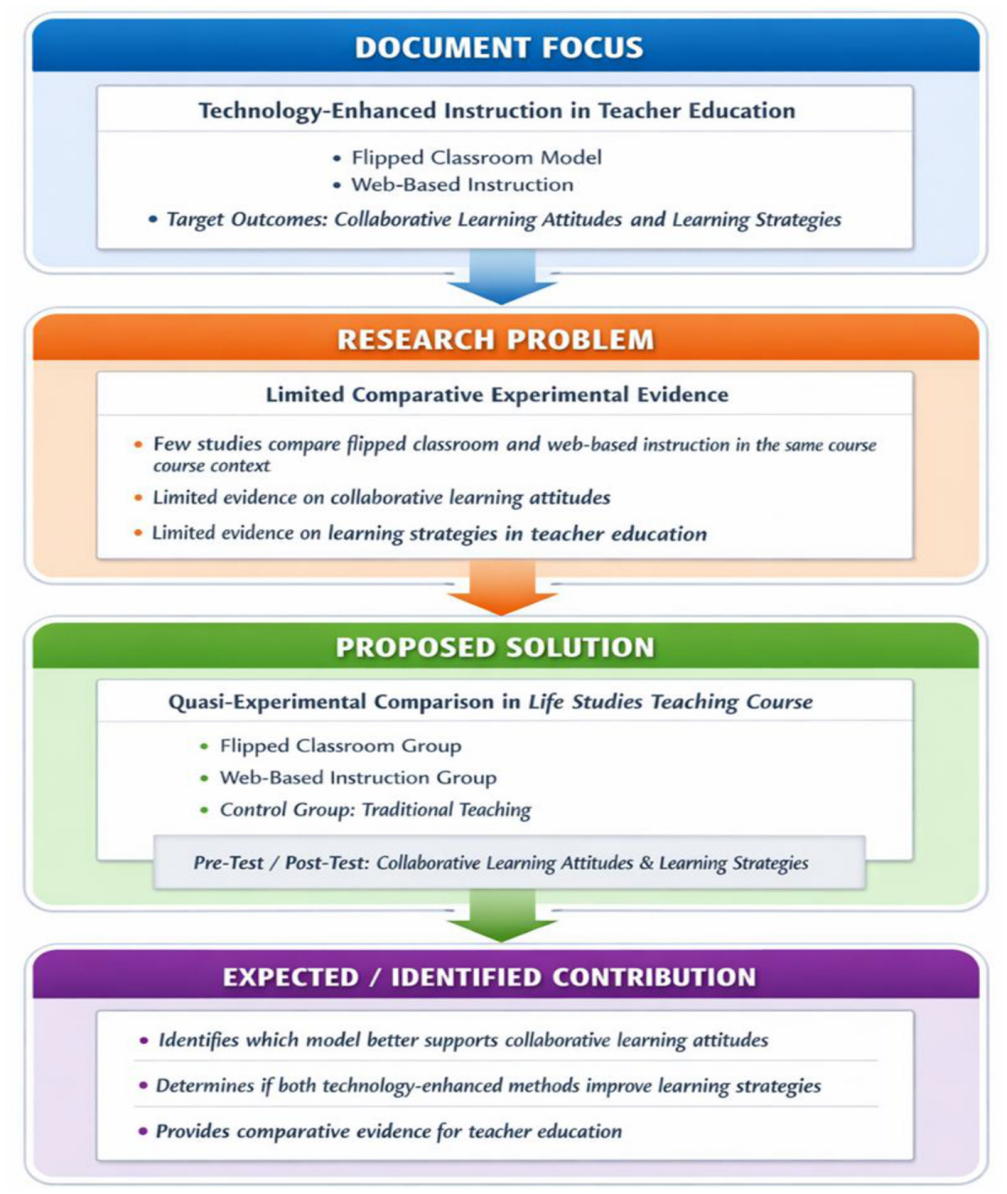
Conceptual graph of the study: document focus, research problem, and proposed solution.

The conceptual structure of this study is organized around three interrelated components. First, the study focuses on technology-supported instruction in teacher education, particularly on two pedagogical formats that have become increasingly prominent in higher education: the flipped classroom model and web-based instruction. Both approaches are associated with active participation, interaction, feedback, and enriched learning environments, and both are assumed to support cooperative learning processes and the strategic regulation of learning. Second, the study is grounded in a specific research problem. Although the literature has reported positive outcomes associated with flipped classroom practices and web-supported learning environments, comparative experimental evidence in teacher education—particularly regarding their simultaneous effects on cooperative learning attitudes and learning strategies within the same course context—remains limited. In other words, previous studies have generally examined these outcomes separately, focused on a single instructional approach, or relied on descriptive rather than comparative evidence. Third, this study proposes a direct response to this gap by comparing three different instructional conditions implemented in the Life Studies Teaching course: flipped classroom instruction, web-based instruction, and traditional instruction. In doing so, the study aims not only to determine whether technology-supported instruction differs from traditional teaching but also to clarify whether two distinct forms of technology-enhanced pedagogy produce different outcomes in terms of cooperative learning attitudes and learning strategies. Accordingly, the study explains how instructional design may shape both the social and strategic dimensions of learning in teacher education. The conceptual map of the study is visually presented in Figure 1.

## 2 Method

### 2.1 Research design

This study employed a quasi-experimental design with a pre-test–post-test control group, a quantitative research approach. This design enables the comparison of intervention and control groups, allowing for a reliable assessment of the effects of the applied intervention. (Creswell 2012) emphasizes that such designs are a strong method in experimental research, as they permit an objective analysis of group differences through pre-test and post-test results. In this study, the effectiveness of activities carried out through the flipped classroom model (intervention group 1) and web tools–supported instruction (intervention group 2) was examined. The control group participants followed only the current *Life Studies* course curriculum. Prior to the intervention, pre-tests were administered to all groups, followed by instructional activities over 7 weeks. After the intervention, post-test measurements were conducted. This research model is significant because it compares the effectiveness of the flipped classroom model and web tools–supported instruction. Moreover, the study was grounded on the hypothesis that the flipped classroom model would be more effective than both web-based and traditional instructional methods.

### 2.2 Study group

According to the results of one-way analysis of variance (ANOVA) applied to the pre-intervention scores of the measurement instruments in Intervention 1, Intervention 2, and control groups, no significant differences were found across groups [CLA (*F* = 2.57, *p* > 0.05); LSHE (*F* = 1.38, *p* > 0.05)]. Regarding gender distribution, the control group included six male students (25%) and 18 female students (75%); Intervention 1 included nine male students (34.6%) and 17 female students (65.4%); and Intervention 2 included five male students (20.8%) and 19 female students (79.2%). The average age of students was 21.7 in the control group, 21.3 in Intervention 1, and 21.4 in Intervention 2.

### 2.3 Data collection instruments

#### 2.3.1 Collaborative learning attitude scale (CLA)

In this study, the scale developed by (Heba and Nouby 2008) was used. The adaptation of the scale into Turkish was carried out by (Kiper 2016). For the adaptation study, the scale was administered to 415 high school students and 356 university students. Confirmatory factor analysis supported the unidimensional, 20-item structure of the scale. The reliability coefficient for the scale was 0.73. The scale uses a five-point Likert-type scale ranging from 1 (“strongly disagree”) to 5 (“strongly agree”). Sample items include:

- “An important role of education is to teach how to get along with others.”
- “Group work makes students dependent on others.”

#### 2.3.2 Learning strategies in higher education scale (LSHE)

Developed by (Boerner et al. 2005) to examine the key factors influencing the academic success of pre-service teachers, this scale assesses learning strategies through self-reports. It was created based on a 12-factor model representing the primary dimensions of learning strategies. The Turkish adaptation of the scale was conducted by (Aydin and Kecici 2019). The scale consists of 67 items rated on a six-point Likert scale, ranging from 1 (“completely false for me”) to 6 (“completely true for me”). The scale comprises 12 sub-dimensions: organization, effort management, planning, regulation, concentration, time management, study environment, peer collaboration, use of literature, structuring, critical thinking, and rehearsal. Together, these factors explain 59.89% of the total variance. The overall reliability coefficient of the scale was calculated as 0.94. Sample items include:

- “I make time to exchange ideas on the subject with my peers.”
- “Compared to most of my peers at university, I devote more time to studying.”

### 2.4 Intervention process

The intervention program was designed to enhance pre-service primary school teachers' use of learning strategies and to foster positive attitudes toward CL. The program's core structure was based on multimedia resources. In preparing these materials, instruction was adopted through the flipped classroom model and web tools. The process was implemented through both in-class activities and distance education. For both experimental groups, instruction was carried out simultaneously in classroom settings and through online learning. Each week, predetermined learning outcomes were addressed by providing teacher candidates with interactive learning environments enriched with web tools and supported by the flipped classroom approach.

#### 2.4.1 Instructional content for intervention group 1

In intervention group 1, instructional content was delivered using the flipped classroom model. The flipped classroom is a contemporary instructional approach that requires students to engage in lower-level cognitive processes (e.g., remembering, understanding) individually before class. In contrast, higher-order cognitive skills, such as analysis, synthesis, and evaluation, are reinforced through hands-on, teacher-guided classroom activities. Within this model, a learner-centered structure was adopted, emphasizing teacher candidates' active participation. For each learning outcome, the researcher prepared the necessary instructional content. Video materials were recorded via *Perculus*, the distance education platform of the researcher's institution, and made accessible to the participants. These videos were shared with teacher candidates 2 days before the face-to-face sessions, enabling them to prepare in advance and acquire foundational concepts beforehand, thereby allowing for a more efficient use of classroom time.

To monitor video engagement and comprehension, interactive assignments and questions were embedded in the video content. The solutions to these questions and the evaluation of the assigned tasks were subsequently discussed during the classroom sessions, where feedback was provided. This process increased student participation and made the learning experience more dynamic and interactive.

**Figure 2:**
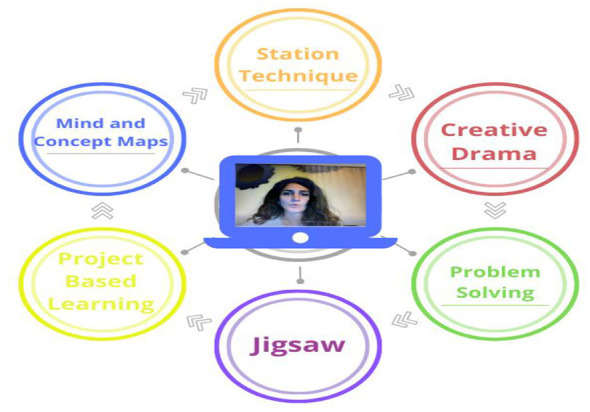
Teaching techniques used in the intervention 1 group.

In the intervention 1 group, the outdoor portion of the lessons was conducted using prepared videos, while in-class instruction employed techniques that fostered collaborative environments. The techniques used in the intervention 1 group are presented in Figure 2.

In classroom practices, active learning methods such as the station technique, creative drama, the jigsaw technique, mind and concept mapping, and project-based learning were employed to enhance students' CL experiences and deepen their learning. These methods were designed to enhance students' interactions with one another, develop their critical thinking skills, and enable them to achieve the course's learning outcomes more effectively. The process was structured to support students' learning strategies and was conducted in a manner that fostered the development of their CL attitudes.

#### 2.4.2 Instructional content for intervention group 2

**Figure 3:**
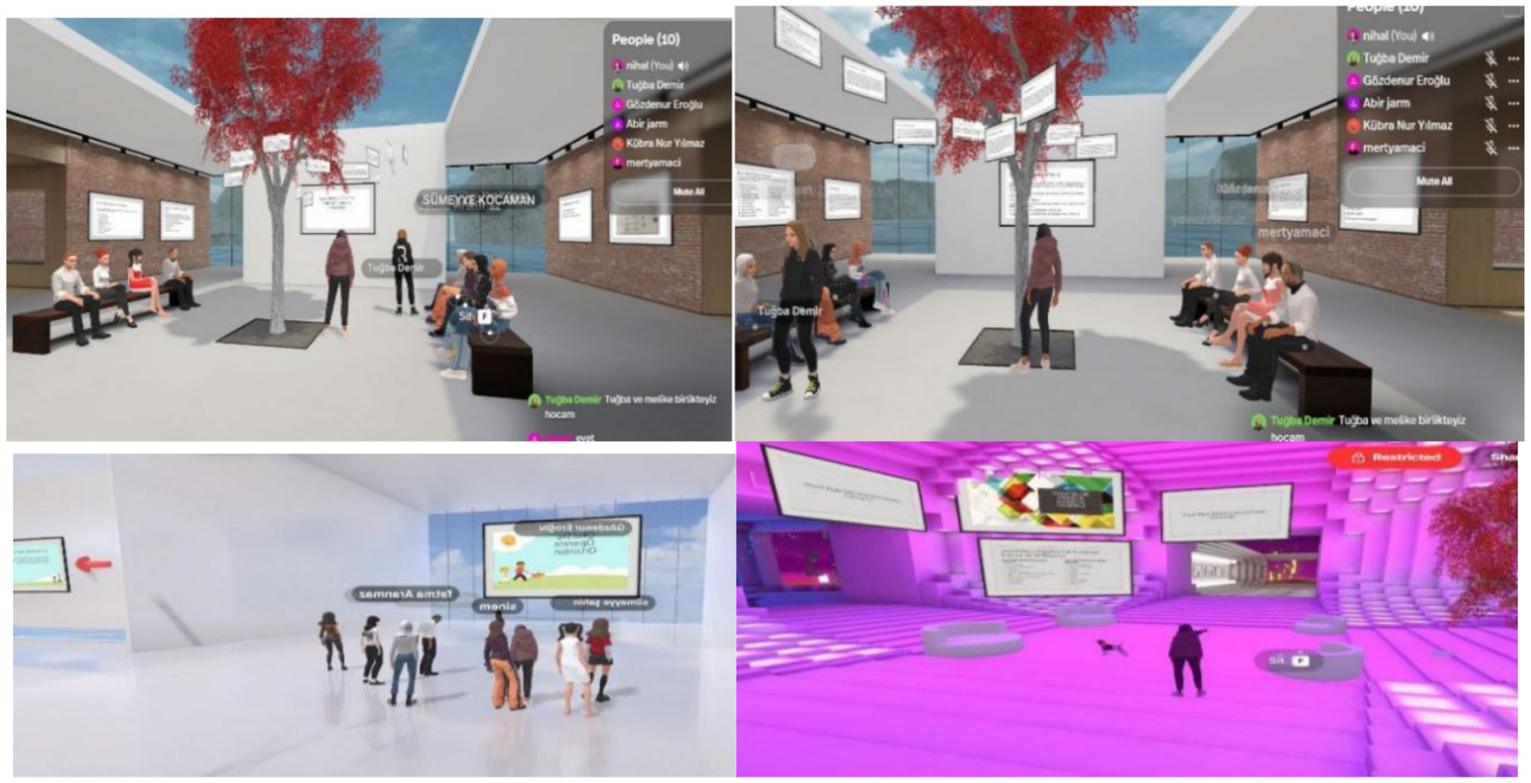
Visuals of the course held on Spatial Web 3.0. Adapted by the authors from the Web 3.0 tool interface used in the study.

In intervention group 2, instructional content was delivered through web tools. Based on the researchers' learning outcomes, the course content was planned, and the Web 3.0 tool *Spatial* was selected as the virtual classroom platform. The platform provided a basis for students to design their own avatars, share course materials, engage in discussions, and receive feedback. Within this environment, students actively participated in lessons supported by interactive questions and immediate feedback, and they worked collaboratively on group projects. Classes in the virtual classroom were conducted on Spatial on the same day and time each week, as determined by the researcher. Examples of visuals from the *Life Studies Teaching* course delivered through *Spatial* are presented in Figure 3.

**Figure 4:**
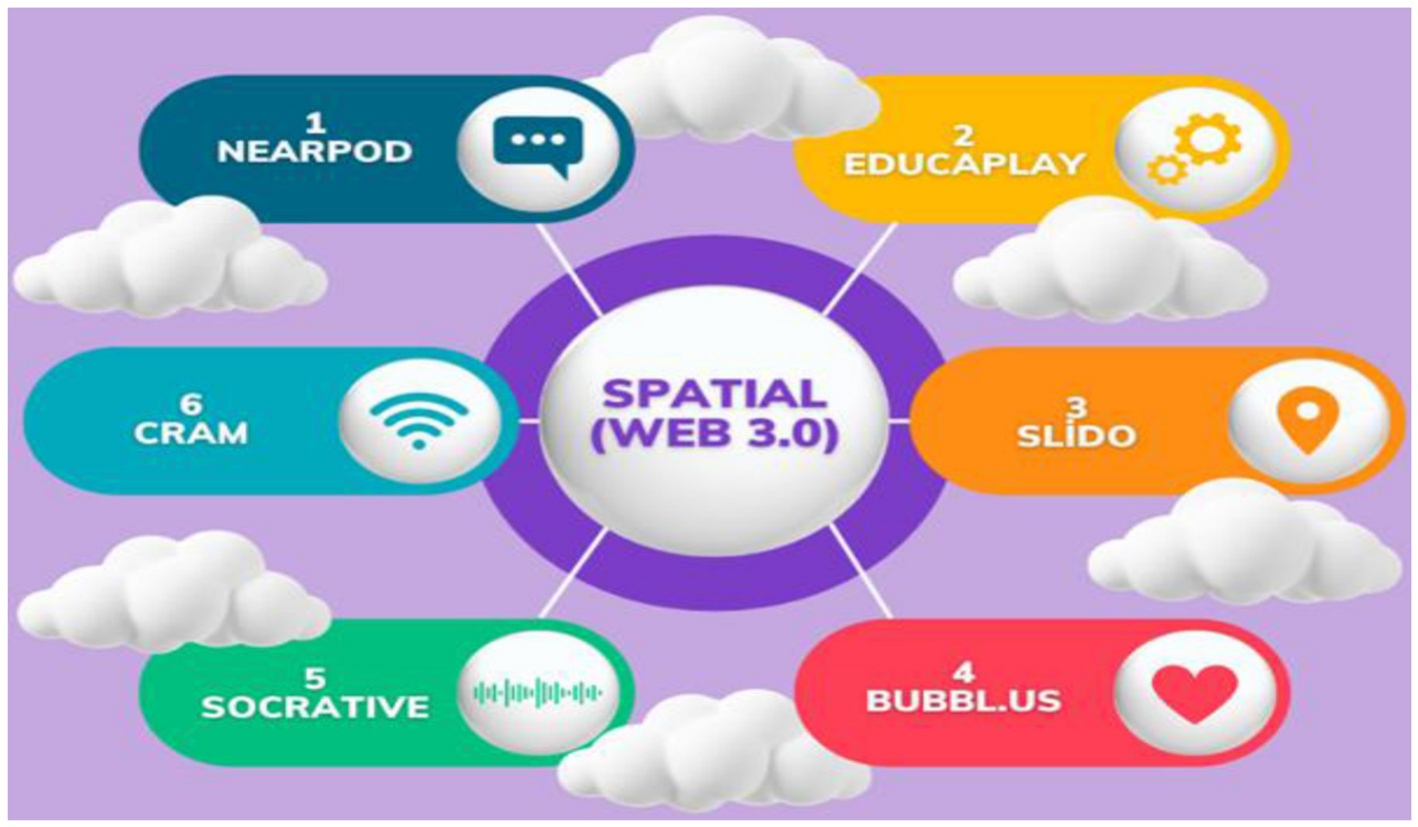
Web tools used in the intervention 2 group.

While lessons were taught in a virtual environment outside the school, they were conducted in the classroom using web tools determined by the researchers. The Web 2.0 tools used throughout the process in intervention group 2 are presented in Figure 4.

Six different web tools were used, each selected to support different learning outcomes. Content sharing, collaboration, visualization, and assessment activities were conducted using these tools. These tools included online document editing and sharing platforms, quiz and survey tools, and software for creating visual materials. Using these tools, students participated in both individual and group activities, developed collaborative projects, and completed structured tasks aligned with the learning outcomes. A summary of the weekly thematic structure of the intervention process, including in-class and distance-learning activities, is presented in Table 1.

**Table 1:** Weekly activity contents.

Week	Intervention group 1 (flipped classroom)	Intervention group 2 (web-based)
Week 1—Purpose, scope, and content of life studies course	Pre-prepared videos explained the aims, scope, and content areas. In class, the station technique was used across four stations (objectives, scope, content, outcomes). Findings were consolidated through group discussion.	Students joined Spatial with avatars. Materials were uploaded to digital boards. Groups collaboratively created a table using an online tool and presented it in the virtual classroom.
Week 2—Teaching basic skills in life studies	Videos introduced basic skills (communication, problem-solving, empathy). In class, creative drama scenarios were enacted, allowing teacher candidates to explore gamified methods for teaching skills.	Breakout rooms were used in Spatial. Students designed drama scenarios and created interactive games via Educaplay. Avatars shared and discussed the products with peers.
Week 3—Value education in life studies	Preliminary videos introduced values (e.g., responsibility, respect). The jigsaw technique was applied. Expert groups prepared activity proposals and returned to the original groups to share and draft example plans.	Expert groups collaborated using Web 2.0 tools. Activities were visualized on digital boards and shared on Spatial. Polls through Slido captured perceptions (e.g., patriotism). Reports were uploaded online.
Week 4—Activities, skills, and concepts	Videos provided examples of competencies, skills, and concepts. Students created mind and concept maps in class to visualize and compare relationships.	Students collaboratively created concept maps with Bubbl.us (LKCollab, LLC, Florida, USA). Completed maps were presented and discussed on the Spatial platform.
Week 5—Strategies, methods, and techniques	Videos introduced teaching strategies. Project-based learning was applied. Groups designed small projects (e.g., teaching values through drama) and presented results.	Groups in Spatial conducted projects. Instant quizzes (Socrative) and polls collected data on strategies. Projects were uploaded to virtual boards with peer feedback.
Week 6—Assessment and evaluation	Videos introduced assessment approaches. Groups designed rubrics, checklists, and performance tasks. Products were presented and peer-reviewed in class.	Online quizzes and Cram were used to design rubrics, flashcards, and tasks. Groups presented assessment tools on Spatial with real-time feedback from the researcher.

#### 2.4.3 Instructional content for the control group

**Figure 5:**
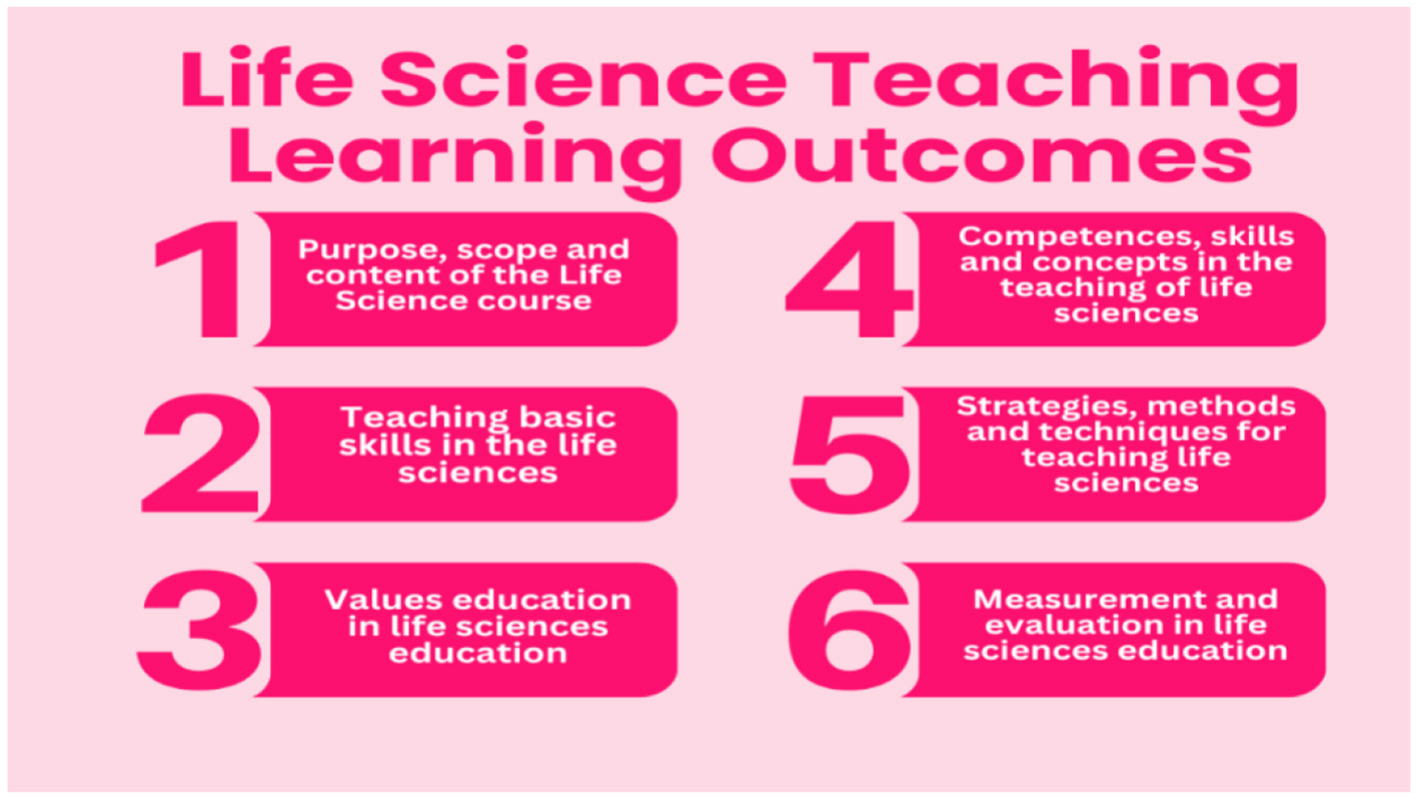
Life sciences course learning outcomes.

The researchers organized the learning outcomes based on the *Life Studies Teaching* course outcomes available in the Bologna course content systems of Karamanoglu Mehmetbey University and Necmettin Erbakan University, as well as the *Primary School Teacher Education Undergraduate Curriculum* of the Turkish Council of Higher Education (see Figure 5) (Karamanoğlu Mehmetbey University, n.d.; Necmettin Erbakan University, 2022; Yüksekögretim Kurulu, 2018). Before instruction began, pre-tests were administered to all three groups. Instruction was then implemented over 6 weeks (18 h in total), with the flipped classroom model applied in intervention group 1, web tools-based instruction in intervention group 2, and conventional instruction aligned with the specified learning outcomes in the Control Group. At the end of the intervention process, post-tests were administered.

### 2.5 Data analysis

The data were analyzed using SPSS 22.0 (IBM Corp., Armonk, NY, USA). Prior to the analysis, miscoding, outliers, and missing values were checked. The assumption of normality was tested using the Shapiro–Wilk test and by examining skewness and kurtosis; the homogeneity of variances was assessed using Levene's test. The findings indicated that the data were normally distributed and that variances were homogeneous. In addition, prior to conducting ANCOVA, the linearity between the covariates (pre-test scores) and the dependent variables (post-test scores) was examined through scatterplots for each group. The plots indicated an approximately linear relationship for both CLA and LSHE. The homogeneity of regression slopes assumption was tested by including the interaction term between group and pre-test scores in the model. These assumption checks were considered before interpreting the ANCOVA results. Descriptive statistics (mean, median, mode) were calculated for the experimental and control groups. A one-way ANOVA was conducted to determine whether there were significant differences among the groups' pre-test scores, and no significant differences were found between the pre-test scores of the experimental and control groups [CLA (*F* = 2.57, *p* > 0.05); LSHE (*F* = 1.38, *p* > 0.05)].

To examine pre- and post-intervention changes within groups, paired-samples *t*-tests were performed, and effect sizes were calculated for analyses that revealed significant differences. A one-way ANOVA was conducted to compare post-test scores, and one-way analysis of covariance (ANCOVA) was employed on post-test scores with pre-test scores as covariates. Where significant differences were observed, effect sizes were reported. For paired samples, Cohen's *d* values were calculated and interpreted as small (*d* < 0.20), medium (0.20 ≤ *d* < 0.80), and large (*d* ≥ 0.80) (Cohen, 1988; Ellis, 2010). For ANCOVA analyses, partial eta squared (η^2^) values were used, with 0.01 interpreted as small, 0.06 as medium, and 0.14 as large effect sizes (Cohen, 1988; Lakens, 2013).

### 2.6 Ethical considerations

Ethical approval for this research was obtained from the Karamanoglu Mehmetbey University Social and Human Sciences Ethics Committee on 06.02.2025 (Decision No: 245123). Throughout the study, ethical principles were observed. Written informed consent was obtained from all participating pre-service teachers. The consent form clearly stated that participation was entirely voluntary and that participants could withdraw from the study at any time. In addition, the confidentiality of participants' personal information was ensured.

## 3 Findings

In this study, the research findings comparing the flipped classroom model, web-based teaching, and presentation-based teaching techniques in a life sciences course taught at universities are presented in Table 2.

**Table 2:** Pre- and post-test comparisons of control and intervention groups.

Group	Measurement	*n*	* $\bar{X}$ *	*S*	sd	*t*	*p*	*d*
CLA								
Intervention 1	Pre-test	26	64.46	7.14	25	−4.96	0.00^*^	0.95
	Post-test	26	73.38	11.12				
Intervention 2	Pre-test	24	61.71	9.43	23	−2.54	0.02^*^	0.23
	Post-test	24	64.29	13.14				
Control	Pre-test	24	59.29	7.55	23	1.82	0.08	-
	Post-test	24	58.13	7.20				
LSHE								
Intervention 1	Pre-test	26	272.23	36.14	25	−5.02	0.00^*^	0.46
	Post-test	26	297.73	37.95				
Intervention 2	Pre-test	24	262.46	24.16	23	6.99	−0.00^*^	0.66
	Post-test	24	280.46	29.98				
Control	Pre-test	24	279.58	44.15	23	6.56	0.00^*^	-
	Post-test	24	256.33	31.45				

^*^Exact p-values are reported where applicable; values smaller than 0.001 are reported as p < 0.001.

In intervention group 1, a significant difference was found between the pre-test and post-test scores of the teacher candidates on the CLA [*t*_(25)_ = −4.96, *p* < 0.001] and LSHE [*t*_(25)_ = −5.02, *p* < 0.001; see Table 2]. Similarly, in intervention group 2, significant increases were observed in the CLA [*t*_(23)_ = −2.54, *p* = 0.02] and LSHE [*t*_(23)_ = 6.99, *p* < 0.001] scores. In both groups, the post-test means were higher than the pre-test means. These findings indicate that both the flipped classroom model and web tools-based instruction were effective in enhancing CLA and LSHE levels. In terms of effect size, the flipped classroom model showed a substantial effect on CLA (*d* = 0.95) and a moderate effect on LSHE (*d* = 0.46). Web tools-based instruction, on the other hand, demonstrated a small-to-moderate effect on CLA (*d* = 0.23) and a moderate effect on LSHE (*d* = 0.66). In the Control Group, no significant difference was found in CLA [*t*_(23)_ = 1.82, *p* > 0.005]. In contrast, a significant adverse change was detected in LSHE [*t*_(23)_ = 6.56, *p* < 0.001]. To test the intervention effects while controlling for pre-test scores, an analysis of covariance (ANCOVA) was conducted. Before conducting ANCOVA, the assumptions of normality, homogeneity of variances, linearity between the covariate and dependent variable, and homogeneity of regression slopes were examined. Normality was evaluated using skewness and kurtosis, and homogeneity of variances was assessed using Levene's test. In addition, scatterplots were inspected to determine whether the relationships between pre-test and post-test scores were approximately linear for each dependent variable. The homogeneity of regression slopes assumption was tested by examining the interaction between group membership and pre-test scores.

**Figure 6:**
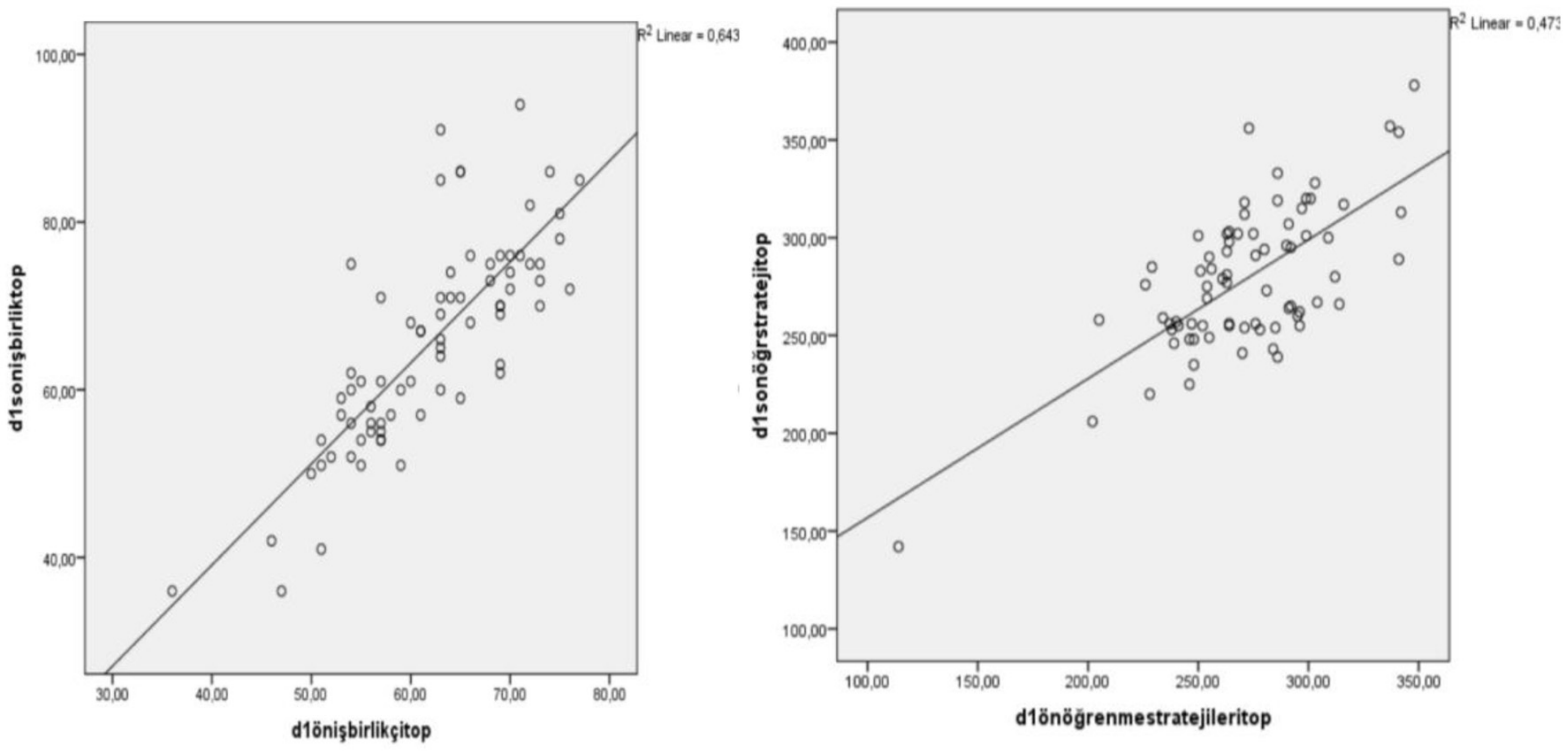
Linear relationships between pre-test and post-test scores for CLA and LSHE.

Before performing ANCOVA, the assumptions were evaluated. Normality was assessed using skewness and kurtosis. The results showed that the values for CLA (skewness = −0.064, kurtosis = −0.134) and LSHE (skewness = −0.181, kurtosis = 1.956) were within acceptable limits (±2), indicating that the data were approximately normally distributed. In addition, the linearity between the covariates (pre-test scores) and the dependent variables (post-test scores) was examined through scatterplots. Visual inspection of the plots suggested an approximately linear relationship between pre-test and post-test scores for both CLA and LSHE across groups (see Figure 6). The homogeneity-of-regression-slopes assumption was tested by examining the interaction between group membership and pre-test scores. For CLA, the interaction effect was not statistically significant, *F*_(2, 68)_ = 3.045, *p* = 0.054, indicating that the assumption was met. However, for LSHE, the interaction effect was statistically significant, *F*_(2, 68)_ = 3.494, *p* = 0.036, suggesting that the homogeneity-of-regression-slopes assumption may not have been fully met. Therefore, the ANCOVA results for LSHE should be interpreted with caution.

As shown in Table 3, after controlling for pre-test scores, a significant difference was found among the adjusted post-test mean scores of the groups for CLA, *F*_(2, 70)_ = 13.70, *p* < 0.001, η^2^ = 0.28. This finding indicates that group membership accounted for a substantial proportion of the variance in CLA post-test scores after adjustment for baseline differences. For LSHE, ANCOVA also yielded a statistically significant group effect, *F*_(2, 70)_ = 45.26, *p* < 0.001, η^2^ = 0.56. However, because the homogeneity of regression slopes assumption was not fully supported for LSHE, this result should be interpreted more cautiously. Although the adjusted group differences remained statistically meaningful, the violation suggests that the relationship between pre-test and post-test LSHE scores may have differed across groups.

**Table 3:** ANCOVA results for CLA and LSHE post-test scores by group.

Source of variance	Sum of squares	Sd	Mean of squares	*F*	*p*	η^2^
CLA						
Corrected model	8333.33	3	2777.78	67.58	0.00	0.74
Pre-test	5376.59	1	5376.59	130.81	0.00	0.65
Group	1126.48	2	563.24	13.70	0.00^*^	**0.28**
Error	2877.15	70	41.10			
Total	328558.00	74				
LSHE						
Corrected model	77722.54	3	25907.51	78.16	0.00	0.77
Pre-test	56225.48	1	56225.48	169.62	0.00	0.71
Group	30005.08	2	15002.54	45.26	0.00^*^	**0.56**
Error	23202.92	70	331.47			
Total	5848890.00	74				

^*^Exact p-values are reported where applicable; values smaller than 0.001 are reported as p < 0.001. Bold values indicate statistically significant results and large effect sizes.

**Figure 7:**
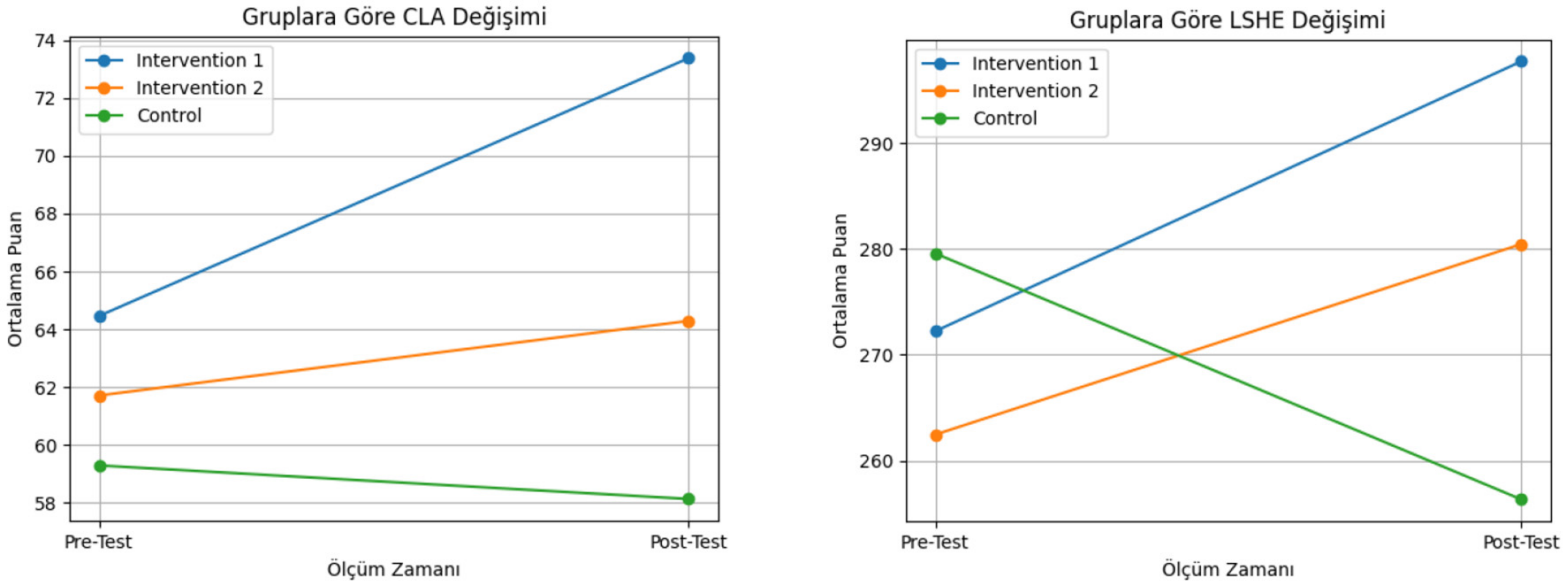
Changes in pre-test and post-test scores for the groups.

As shown in Table 4, the CLA scores of teacher candidates in intervention group 1 ($\bar{x}$ = 73.38) were significantly higher than those in both intervention group 2 ($\bar{x}$ = 64.29, MD = 6.13, *p* < 0.005) and the Control Group ($\bar{x}$ = 58.13, MD = 9.69, *p* < 0.001). These findings suggest that the flipped classroom model enhanced teacher candidates' attitudes toward CL more effectively than either web tools-based instruction or instruction based solely on learning outcomes. In addition, the LSHE scores indicate that both intervention group 1 ($\bar{x}$ = 297.73) and intervention group 2 ($\bar{x}$ = 280.46) achieved significantly higher scores than the Control Group ($\bar{x}$ = 256.33, MD = 47.18, *p* < 0.001). This demonstrates that, in terms of learning strategies, both the flipped classroom model and web tools-based instruction were more effective than traditional outcome-based teaching. Additionally, changes in pre- and post-test scores across groups are illustrated in Figure 7.

**Table 4:** Bonferroni pairwise comparisons for adjusted post-test CLA and LSHE scores.

To compare		Difference average	*S*	*p*	% 95 confidence interval	
					Lower limit	Upper limit
CLA						
Intervention 1	Control	9.69	1.88	0.00^*^	5.08	14.30
	Intervention 2	6.13	1.83	0.00^*^	1.63	10.62
Intervention 2	Control	3.56	1.87	0.18	−1.01	8.14
	Intervention 1	−6.13	1.83	0.00^*^	−10.62	−1.63
Control	Intervention 1	−9.69	1.88	0.00^*^	−14.30	−5.08
	Intervention 2	−3.56	1.87	0.18	−8.14	1.01
LSHE						
Intervention 1	Control	47.18	5.17	0.00^*^	34.49	59.87
	Intervention 2	9.59	5.19	0.21	−3.14	22.31
Intervention 2	Control	37.59	5.36	0.00^*^	24.46	50.73
	Intervention 1	−9.59	5.19	0.21	−22.31	3.14
Control	Intervention 1	−47.18	5.17	0.00^*^	−59.87	−34.49
	Intervention 2	−37.59	5.36	0.00^*^	−50.73	−24.46

Mean differences are based on adjusted post-test means. ^*^Exact p-values are reported where applicable; values smaller than 0.001 are reported as p < 0.001.

## 4 Discussion

This study examined the effects of flipped classroom model-based and web tools-based activities on pre-service teachers' CL attitudes and learning strategies. The findings revealed that both approaches had a positive effect on CL attitudes. A review of the literature shows that the flipped classroom method is more effective than traditional approaches in enhancing learning achievement at both secondary and higher education levels. Some researchers have suggested that students' positive or negative perceptions of CL depend on various factors, including their prior learning experiences and their self-awareness of how they learn (Stover and Holland, 2018). CL has also been reported to provide advantages in related domains such as motivation, self-efficacy, and engagement (Galindo-Dominguez, 2021). Flipped classroom applications have also been shown to improve both learning outcomes and CL attitudes compared to traditional methods (Bishop and Verleger, 2013; Strayer, 2012; Foldnes, 2016). These findings support the hypothesis that the flipped classroom approach enhances CL attitudes. In this context, the study goes beyond demonstrating the benefits of technology-supported instruction by providing comparative evidence that different technology-supported teaching models do not contribute to the same outcomes in the same way. The flipped classroom model yielded stronger gains in cooperative learning attitudes compared to web-based instruction, while both technology-supported conditions were more effective than traditional lecture-based instruction in supporting learning strategies. This distinction is important for teacher education because it indicates that not merely the use of technology, but the pedagogical organization of technology, shapes the nature of learning outcomes.

The integration of digital technologies into higher education offers students more engaging learning opportunities and encourages participation in a range of activities, from passive to active and interactive. Participation in such activities is positively associated with learning outcomes (Wekerle et al., 2022). As digital-age learners, students are expected to acquire skills in knowledge production, sharing, and collaboration through the use of web tools in lifelong learning processes (Robles, 2013). The literature also provides evidence that digital technologies and content can enhance CL attitudes (Barreto et al., 2022; Chatterjee and Correia, 2019; Jang and Park, 2014; Kiper, 2022; Pürbudak, 2020; Yildirim Yakar, 2022). However, some studies have reported limited effects. For instance, (Kiper 2022) found that cloud-computing-based programming instruction did not significantly affect CL attitudes, while (Yildirim Yakar 2022) reported no significant differences in attitudes toward online CL. Nevertheless, students highlighted the usefulness of web tools for collaboration. Conversely, other studies—such as (Cankaya and Yunkul 2018), who used Edmodo, (Jang and Park 2014), who explored Web 2.0 environments, and (Pürbudak 2020), who employed web-based group activities—identified positive changes in CL attitudes. These findings indicate that, overall, instruction supported by web tools contributes to the improvement of CL attitudes. The stronger effect of the flipped classroom model on cooperative learning attitudes may be related to the way collaboration was pedagogically structured in this study. In the flipped condition, following pre-class preparation, face-to-face, interdependent classroom practices such as station work, jigsaw activities, creative drama, concept mapping, and project-based tasks were implemented. This structure may have fostered more sustained peer interaction, shared responsibility, and visible group accountability compared to participation limited to web-based environments. Therefore, the findings suggest that cooperative learning attitudes can be strengthened when technology is combined with structured in-class social interaction.

Research also suggests that both web tools and the flipped classroom model are effective in influencing pre-service teachers' learning strategies. Learning strategies are closely linked to the diversity and effectiveness of instructional methods. In this sense, the teaching approaches employed by instructors play a key role in helping students “learn how to learn.” In the present study, both the flipped classroom model and web tools enhanced the learning environment and supported the use of strategies. Similar findings have been reported in previous studies. For example, (Küçük 2012) demonstrated that online learning environments support the use of strategy, (Ma 2012) found that web-based environments contribute to the adoption of language learning strategies, and (Zlatovic et al. 2015) showed that online assessments influence the emergence of specific strategies. (Clayton et al. 2010) reported that students' learning environment preferences are associated with motivational orientations and strategy use.

Furthermore, (Diningrat et al. 2024) demonstrated that online flipped classroom models enhance self-regulated learning strategies, while (Hava 2021) found that flipped classrooms improve deep learning strategies and student engagement. However, in the Context of the *Life Studies* course, the findings revealed an adverse effect on learning strategies. This may be attributed to the limited integration of traditional course objectives and content with digital technologies. Therefore, approaches such as web tools and the flipped classroom model necessitate the redesign of pedagogical structures and the integration of digital content (Zainuddin and Halili, 2016).

## 5 Conclusion

The findings of this study indicate that both instructional approaches positively influenced the CL attitudes and LS of pre-service teachers. However, these effects varied depending on the method. The flipped classroom model, with its student-centered structure and structured group activities, was more effective in enhancing CL attitudes. In contrast, instruction supported by web tools more effectively fostered LS by providing interactive and enriched learning environments. Traditional methods, on the other hand, appeared to limit strategic LS. Taken together, these results suggest that improving students' attitudes toward cooperative learning and learning strategies in higher education may be supported by student-centered and collaboration-oriented instructional approaches, with technology serving as a facilitating component rather than a necessary condition.

## 6 Limitations and recommendations

This study is subject to several limitations. An additional limitation concerns the ANCOVA results for LSHE, for which the homogeneity of regression slopes assumption was not fully supported. Accordingly, although the adjusted group differences were statistically significant, these findings should be interpreted with some caution. First, the findings are limited to the measurement instruments employed. This means that participants' views and experiences were assessed within the scope of these tools. Future research should triangulate quantitative findings with qualitative data by incorporating interviews, observations, and open-ended surveys to enhance the validity of the results. Second, the study was restricted to two universities in the Central Anatolia region and to the *Life Studies* curriculum taught in these institutions. Broader research should be conducted in other regions and across different teacher education programs. Furthermore, as the data relied on students' self-reports, potential measurement errors such as social desirability bias may have occurred; thus, longitudinal studies are recommended. For future research, studies conducted with larger and more diverse samples could enhance the generalizability of the results. Research across different age groups, educational levels, and cultural contexts would also contribute significantly to the field. The integration of technology-enhanced instructional tools into courses may positively influence pre-service teachers' attitudes toward technology. Virtual platforms, interactive content, and web-based tools can be supportive elements for CL attitudes. Finally, practices that encourage pre-service teachers to use a variety of digital tools effectively should be incorporated. A focus on the application of diverse learning strategies by educators and researchers can contribute to improving both individual Learning and the overall quality of instruction.

## Data Availability

The original contributions presented in the study are included in the article/Supplementary material, further inquiries can be directed to the corresponding author/s.
