# Supplementary material for: The role of flipped classroom models and web-based instruction in developing collaborative learning attitudes and learning strategies
Source: Front Psychol. 2026 Jun 30;17:1792842. doi: 10.3389/fpsyg.2026.1792842 (PMC13365127; doi:10.3389/fpsyg.2026.1792842)

```
 GET
  FILE='C:\Users\Gizem\Pictures\NİHAL HOCAYLA ÇALIŞMA 2023\tersyüz-web ana analiz\tersyüz-we
DATASET NAME DataSet1 WINDOW=FRONT.
FREQUENCIES VARIABLES=d1önişbirlikçitop d1öndijitaltektop d1önöğrenmestratejileritop d1soniş
    d1sonöğrstratejitop
  /STATISTICS=MEAN MEDIAN MODE SKEWNESS SESKEW KURTOSIS SEKURT
  /ORDER=ANALYSIS.
```

Frequencies

Notes

Output Created		03-NOV-2024 21:01:01
Comments		
Input	Data	C:\Users\Gizem\Pictures\NİHAL HOCAYLA ÇALIŞMA 2023\tersyüz-web ana analiz\tersyüz-web ana analiz.sav
	Active Dataset	DataSet1
	Filter	<none>
	Weight	<none>
	Split File	<none>
	N of Rows in Working Data File	74
Missing Value Handling	Definition of Missing	User-defined missing values are treated as missing.
	Cases Used	Statistics are based on all cases with valid data.
Syntax		FREQUENCIES VARIABLES=d1önişbirlikçitop d1öndijitaltektop d1önöğrenmestratejileritop d1sonişbirliktop d1sondijitaltektop d1sonöğrstratejitop /STATISTICS=MEAN MEDIAN MODE SKEWNESS SESKEW KURTOSIS SEKURT /ORDER=ANALYSIS.
Resources	Processor Time	00:00:00,02
	Elapsed Time	00:00:00,03

[DataSet1] C:\Users\Gizem\Pictures\NİHAL HOCAYLA ÇALIŞMA 2023\tersyüz-web ana analiz\tersyüz-web ana analiz.sav

Statistics

		d1önişbirlikçitop	d1öndijitaltektop	d1önöğrenme stratejileritop	d1sonişbirliktop
N	Valid	74	74	74	74
	Missing	0	0	0	0
Mean		61,8919	127,5000	271,4459	65,4865
Median		63,0000	126,0000	271,0000	66,5000
Mode		63,00	123,00[a]	263,00[a]	54,00[a]
Skewness		-,320	,121	-,862	-,064
Std. Error of Skewness		,279	,279	,279	,279
Kurtosis		-,025	,362	4,172	-,134
Std. Error of Kurtosis		,552	,552	,552	,552

Statistics

		d1sondijitaltektop	d1sonöğrstratejitop
N	Valid	74	74
	Missing	0	0
Mean		133,3514	278,7027
Median		132,0000	276,5000
Mode		125,00	255,00[a]
Skewness		,609	-,181
Std. Error of Skewness		,279	,279
Kurtosis		,935	1,956
Std. Error of Kurtosis		,552	,552

a. Multiple modes exist. The smallest value is shown

Frequency Table

d1önişbirlikçitop

		Frequency	Percent	Valid Percent	Cumulative Percent
Valid	36,00	1	1,4	1,4	1,4
	46,00	1	1,4	1,4	2,7
	47,00	1	1,4	1,4	4,1
	50,00	1	1,4	1,4	5,4
	51,00	3	4,1	4,1	9,5
	52,00	1	1,4	1,4	10,8
	53,00	2	2,7	2,7	13,5
	54,00	5	6,8	6,8	20,3
	55,00	3	4,1	4,1	24,3
	56,00	3	4,1	4,1	28,4
	57,00	6	8,1	8,1	36,5
	58,00	1	1,4	1,4	37,8
	59,00	2	2,7	2,7	40,5
	60,00	2	2,7	2,7	43,2
	61,00	3	4,1	4,1	47,3
	63,00	8	10,8	10,8	58,1
	64,00	2	2,7	2,7	60,8
	65,00	4	5,4	5,4	66,2
	66,00	2	2,7	2,7	68,9
	68,00	2	2,7	2,7	71,6
	69,00	6	8,1	8,1	79,7
	70,00	3	4,1	4,1	83,8
	71,00	2	2,7	2,7	86,5
	72,00	2	2,7	2,7	89,2
	73,00	3	4,1	4,1	93,2
	74,00	1	1,4	1,4	94,6
	75,00	2	2,7	2,7	97,3
	76,00	1	1,4	1,4	98,6
	77,00	1	1,4	1,4	100,0
	Total	74	100,0	100,0	

d1öndijitaltektop

		Frequency	Percent	Valid Percent	Cumulative Percent
Valid	94,00	1	1,4	1,4	1,4
	111,00	1	1,4	1,4	2,7
	112,00	2	2,7	2,7	5,4
	113,00	2	2,7	2,7	8,1
	114,00	1	1,4	1,4	9,5
	115,00	1	1,4	1,4	10,8
	116,00	2	2,7	2,7	13,5
	117,00	4	5,4	5,4	18,9
	118,00	1	1,4	1,4	20,3
	119,00	2	2,7	2,7	23,0
	120,00	3	4,1	4,1	27,0
	121,00	1	1,4	1,4	28,4
	122,00	3	4,1	4,1	32,4
	123,00	5	6,8	6,8	39,2
	124,00	3	4,1	4,1	43,2
	125,00	1	1,4	1,4	44,6
	126,00	5	6,8	6,8	51,4
	127,00	4	5,4	5,4	56,8
	128,00	2	2,7	2,7	59,5
	129,00	2	2,7	2,7	62,2
	130,00	2	2,7	2,7	64,9
	131,00	1	1,4	1,4	66,2
	132,00	1	1,4	1,4	67,6
	133,00	4	5,4	5,4	73,0
	134,00	2	2,7	2,7	75,7
	135,00	1	1,4	1,4	77,0
	137,00	4	5,4	5,4	82,4
	138,00	2	2,7	2,7	85,1
	140,00	1	1,4	1,4	86,5
	141,00	2	2,7	2,7	89,2
	142,00	1	1,4	1,4	90,5
	143,00	1	1,4	1,4	91,9
	146,00	1	1,4	1,4	93,2
	148,00	1	1,4	1,4	94,6
	149,00	2	2,7	2,7	97,3
	151,00	1	1,4	1,4	98,6
	152,00	1	1,4	1,4	100,0
	Total	74	100,0	100,0	

d1önöğrenmestratejileritop

		Frequency	Percent	Valid Percent	Cumulative Percent
Valid	114,00	1	1,4	1,4	1,4
	202,00	1	1,4	1,4	2,7
	205,00	1	1,4	1,4	4,1
	226,00	1	1,4	1,4	5,4
	228,00	1	1,4	1,4	6,8
	229,00	1	1,4	1,4	8,1
	234,00	1	1,4	1,4	9,5
	237,00	1	1,4	1,4	10,8
	238,00	1	1,4	1,4	12,2
	239,00	1	1,4	1,4	13,5
	240,00	1	1,4	1,4	14,9
	241,00	1	1,4	1,4	16,2
	246,00	2	2,7	2,7	18,9
	247,00	1	1,4	1,4	20,3
	248,00	2	2,7	2,7	23,0
	250,00	1	1,4	1,4	24,3
	251,00	1	1,4	1,4	25,7
	252,00	1	1,4	1,4	27,0
	254,00	2	2,7	2,7	29,7
	255,00	2	2,7	2,7	32,4
	256,00	1	1,4	1,4	33,8
	261,00	1	1,4	1,4	35,1
	263,00	4	5,4	5,4	40,5
	264,00	4	5,4	5,4	45,9
	268,00	1	1,4	1,4	47,3
	270,00	1	1,4	1,4	48,6
	271,00	3	4,1	4,1	52,7
	273,00	1	1,4	1,4	54,1
	275,00	1	1,4	1,4	55,4
	276,00	2	2,7	2,7	58,1
	278,00	1	1,4	1,4	59,5
	280,00	1	1,4	1,4	60,8
	281,00	1	1,4	1,4	62,2
	284,00	1	1,4	1,4	63,5
	285,00	1	1,4	1,4	64,9
	286,00	3	4,1	4,1	68,9
	290,00	1	1,4	1,4	70,3
	291,00	2	2,7	2,7	73,0
	292,00	2	2,7	2,7	75,7
	295,00	1	1,4	1,4	77,0
	296,00	2	2,7	2,7	79,7
	297,00	1	1,4	1,4	81,1

d1önöğrenmestratejileritop

		Frequency	Percent	Valid Percent	Cumulative Percent
	299,00	2	2,7	2,7	83,8
	301,00	1	1,4	1,4	85,1
	303,00	1	1,4	1,4	86,5
	304,00	1	1,4	1,4	87,8
	309,00	1	1,4	1,4	89,2
	312,00	1	1,4	1,4	90,5
	314,00	1	1,4	1,4	91,9
	316,00	1	1,4	1,4	93,2
	337,00	1	1,4	1,4	94,6
	341,00	2	2,7	2,7	97,3
	342,00	1	1,4	1,4	98,6
	348,00	1	1,4	1,4	100,0
	Total	74	100,0	100,0	

d1sonişbirliktop

		Frequency	Percent	Valid Percent	Cumulative Percent
Valid	36,00	2	2,7	2,7	2,7
	41,00	1	1,4	1,4	4,1
	42,00	1	1,4	1,4	5,4
	50,00	1	1,4	1,4	6,8
	51,00	3	4,1	4,1	10,8
	52,00	2	2,7	2,7	13,5
	54,00	4	5,4	5,4	18,9
	55,00	2	2,7	2,7	21,6
	56,00	3	4,1	4,1	25,7
	57,00	3	4,1	4,1	29,7
	58,00	1	1,4	1,4	31,1
	59,00	2	2,7	2,7	33,8
	60,00	3	4,1	4,1	37,8
	61,00	3	4,1	4,1	41,9
	62,00	2	2,7	2,7	44,6
	63,00	1	1,4	1,4	45,9
	64,00	1	1,4	1,4	47,3
	65,00	1	1,4	1,4	48,6
	66,00	1	1,4	1,4	50,0
	67,00	2	2,7	2,7	52,7
	68,00	2	2,7	2,7	55,4
	69,00	2	2,7	2,7	58,1
	70,00	3	4,1	4,1	62,2
	71,00	4	5,4	5,4	67,6
	72,00	2	2,7	2,7	70,3

d1sonişbirliktop

	Frequency	Percent	Valid Percent	Cumulative Percent
73,00	2	2,7	2,7	73,0
74,00	2	2,7	2,7	75,7
75,00	4	5,4	5,4	81,1
76,00	4	5,4	5,4	86,5
78,00	1	1,4	1,4	87,8
81,00	1	1,4	1,4	89,2
82,00	1	1,4	1,4	90,5
85,00	2	2,7	2,7	93,2
86,00	3	4,1	4,1	97,3
91,00	1	1,4	1,4	98,6
94,00	1	1,4	1,4	100,0
Total	74	100,0	100,0	

d1sondijitaltektop

		Frequency	Percent	Valid Percent	Cumulative Percent
Valid	103,00	1	1,4	1,4	1,4
	105,00	1	1,4	1,4	2,7
	108,00	1	1,4	1,4	4,1
	111,00	1	1,4	1,4	5,4
	112,00	2	2,7	2,7	8,1
	114,00	1	1,4	1,4	9,5
	115,00	1	1,4	1,4	10,8
	117,00	1	1,4	1,4	12,2
	118,00	2	2,7	2,7	14,9
	119,00	1	1,4	1,4	16,2
	120,00	2	2,7	2,7	18,9
	122,00	4	5,4	5,4	24,3
	123,00	1	1,4	1,4	25,7
	124,00	3	4,1	4,1	29,7
	125,00	5	6,8	6,8	36,5
	126,00	1	1,4	1,4	37,8
	127,00	2	2,7	2,7	40,5
	128,00	2	2,7	2,7	43,2
	129,00	1	1,4	1,4	44,6
	130,00	3	4,1	4,1	48,6
	132,00	3	4,1	4,1	52,7
	134,00	2	2,7	2,7	55,4
	135,00	2	2,7	2,7	58,1
	137,00	3	4,1	4,1	62,2
	138,00	1	1,4	1,4	63,5
	139,00	2	2,7	2,7	66,2

d1sondijitaltektop

	Frequency	Percent	Valid Percent	Cumulative Percent
141,00	2	2,7	2,7	68,9
142,00	1	1,4	1,4	70,3
143,00	2	2,7	2,7	73,0
144,00	3	4,1	4,1	77,0
145,00	3	4,1	4,1	81,1
146,00	2	2,7	2,7	83,8
147,00	1	1,4	1,4	85,1
148,00	1	1,4	1,4	86,5
149,00	1	1,4	1,4	87,8
150,00	1	1,4	1,4	89,2
151,00	1	1,4	1,4	90,5
152,00	1	1,4	1,4	91,9
154,00	1	1,4	1,4	93,2
156,00	1	1,4	1,4	94,6
157,00	1	1,4	1,4	95,9
160,00	1	1,4	1,4	97,3
175,00	1	1,4	1,4	98,6
184,00	1	1,4	1,4	100,0
Total	74	100,0	100,0	

d1sonöğrstratejitop

		Frequency	Percent	Valid Percent	Cumulative Percent
Valid	142,00	1	1,4	1,4	1,4
	206,00	1	1,4	1,4	2,7
	220,00	1	1,4	1,4	4,1
	225,00	1	1,4	1,4	5,4
	235,00	1	1,4	1,4	6,8
	239,00	1	1,4	1,4	8,1
	241,00	1	1,4	1,4	9,5
	243,00	1	1,4	1,4	10,8
	246,00	1	1,4	1,4	12,2
	248,00	2	2,7	2,7	14,9
	249,00	1	1,4	1,4	16,2
	253,00	2	2,7	2,7	18,9
	254,00	2	2,7	2,7	21,6
	255,00	4	5,4	5,4	27,0
	256,00	4	5,4	5,4	32,4
	257,00	1	1,4	1,4	33,8
	258,00	1	1,4	1,4	35,1
	259,00	1	1,4	1,4	36,5
	260,00	1	1,4	1,4	37,8

d1sonöğrstratejitop

	Frequency	Percent	Valid Percent	Cumulative Percent
262,00	1	1,4	1,4	39,2
264,00	1	1,4	1,4	40,5
265,00	1	1,4	1,4	41,9
266,00	1	1,4	1,4	43,2
267,00	1	1,4	1,4	44,6
269,00	1	1,4	1,4	45,9
273,00	1	1,4	1,4	47,3
275,00	1	1,4	1,4	48,6
276,00	1	1,4	1,4	50,0
277,00	1	1,4	1,4	51,4
279,00	1	1,4	1,4	52,7
280,00	1	1,4	1,4	54,1
281,00	1	1,4	1,4	55,4
283,00	1	1,4	1,4	56,8
284,00	1	1,4	1,4	58,1
285,00	1	1,4	1,4	59,5
289,00	1	1,4	1,4	60,8
290,00	1	1,4	1,4	62,2
291,00	1	1,4	1,4	63,5
293,00	1	1,4	1,4	64,9
294,00	1	1,4	1,4	66,2
295,00	1	1,4	1,4	67,6
296,00	1	1,4	1,4	68,9
298,00	1	1,4	1,4	70,3
300,00	1	1,4	1,4	71,6
301,00	2	2,7	2,7	74,3
302,00	3	4,1	4,1	78,4
303,00	1	1,4	1,4	79,7
307,00	1	1,4	1,4	81,1
312,00	1	1,4	1,4	82,4
313,00	1	1,4	1,4	83,8
315,00	1	1,4	1,4	85,1
317,00	1	1,4	1,4	86,5
318,00	1	1,4	1,4	87,8
319,00	1	1,4	1,4	89,2
320,00	2	2,7	2,7	91,9
328,00	1	1,4	1,4	93,2
333,00	1	1,4	1,4	94,6
354,00	1	1,4	1,4	95,9
356,00	1	1,4	1,4	97,3
357,00	1	1,4	1,4	98,6
378,00	1	1,4	1,4	100,0

d1sonöğrstratejitop

	Frequency	Percent	Valid Percent	Cumulative Percent
Total	74	100,0	100,0	

```
 USE ALL.
COMPUTE filter_$=(grup = 1).
VARIABLE LABELS filter_$ 'grup = 1 (FILTER)'.
VALUE LABELS filter_$ 0 'Not Selected' 1 'Selected'.
FORMATS filter_$ (f1.0).
FILTER BY filter_$.
EXECUTE.
FREQUENCIES VARIABLES=d1önişbirlikçitop d1öndijitaltektop d1önöğrenmestratejileritop d1soniş
    d1sonöğrstratejitop
  /STATISTICS=MEAN MEDIAN MODE SKEWNESS SESKEW KURTOSIS SEKURT
  /ORDER=ANALYSIS.
```

Frequencies

Notes

Output Created		03-NOV-2024 21:02:11
Comments		
Input	Data	C: \Users\Gizem\Pictures\NİHAL HOCAYLA ÇALIŞMA 2023\tersyüz-web ana analiz\tersyüz-web ana analiz.sav
	Active Dataset	DataSet1
	Filter	grup = 1 (FILTER)
	Weight	<none>
	Split File	<none>
	N of Rows in Working Data File	26
Missing Value Handling	Definition of Missing	User-defined missing values are treated as missing.
	Cases Used	Statistics are based on all cases with valid data.
Syntax		FREQUENCIES VARIABLES=d1önişbirlikçitop d1öndijitaltektop d1önöğrenmestratejileritop d1sonişbirliktop d1sondijitaltektop d1sonöğrstratejitop /STATISTICS=MEAN MEDIAN MODE SKEWNESS SESKEW KURTOSIS SEKURT /ORDER=ANALYSIS.

Notes

Resources	Processor Time	00:00:00,00
	Elapsed Time	00:00:00,02

```
[DataSet1] C:\Users\Gizem\Pictures\NİHAL HOCAYLA ÇALIŞMA 2023\tersyüz-web ana analiz\tersyüz-web ana analiz.sav
```

Statistics

		d1önişbirlikçitop	d1öndijitaltektop	d1önöğrenme stratejileritop	d1sonişbirliktop
N	Valid	26	26	26	26
	Missing	0	0	0	0
Mean		64,4615	129,3846	272,2308	73,3846
Median		64,5000	130,5000	267,5000	74,5000
Mode		63,00	123,00	264,00[a]	71,00[a]
Skewness		-,162	-,389	,477	-,211
Std. Error of Skewness		,456	,456	,456	,456
Kurtosis		-,871	,247	-,075	-,361
Std. Error of Kurtosis		,887	,887	,887	,887

Statistics

		d1sondijitaltektop	d1sonöğrstratejitop
N	Valid	26	26
	Missing	0	0
Mean		135,1923	297,7308
Median		136,0000	299,5000
Mode		122,00[a]	302,00
Skewness		,043	,158
Std. Error of Skewness		,456	,456
Kurtosis		-,600	-,166
Std. Error of Kurtosis		,887	,887

a. Multiple modes exist. The smallest value is shown

Frequency Table

d1önişbirlikçitop

		Frequency	Percent	Valid Percent	Cumulative Percent
Valid	51,00	1	3,8	3,8	3,8
	54,00	2	7,7	7,7	11,5
	55,00	1	3,8	3,8	15,4
	56,00	1	3,8	3,8	19,2
	57,00	1	3,8	3,8	23,1
	59,00	1	3,8	3,8	26,9
	60,00	1	3,8	3,8	30,8
	63,00	3	11,5	11,5	42,3
	64,00	2	7,7	7,7	50,0
	65,00	2	7,7	7,7	57,7
	66,00	1	3,8	3,8	61,5
	68,00	1	3,8	3,8	65,4
	69,00	2	7,7	7,7	73,1
	70,00	1	3,8	3,8	76,9
	71,00	1	3,8	3,8	80,8
	72,00	1	3,8	3,8	84,6
	73,00	2	7,7	7,7	92,3
	75,00	1	3,8	3,8	96,2
	77,00	1	3,8	3,8	100,0
	Total	26	100,0	100,0	

d1öndijitaltektop

		Frequency	Percent	Valid Percent	Cumulative Percent
Valid	94,00	1	3,8	3,8	3,8
	112,00	2	7,7	7,7	11,5
	113,00	1	3,8	3,8	15,4
	117,00	1	3,8	3,8	19,2
	120,00	1	3,8	3,8	23,1
	121,00	1	3,8	3,8	26,9
	123,00	3	11,5	11,5	38,5
	124,00	1	3,8	3,8	42,3
	127,00	1	3,8	3,8	46,2
	130,00	1	3,8	3,8	50,0
	131,00	1	3,8	3,8	53,8
	133,00	2	7,7	7,7	61,5
	134,00	1	3,8	3,8	65,4
	137,00	2	7,7	7,7	73,1
	138,00	1	3,8	3,8	76,9
	141,00	1	3,8	3,8	80,8
	143,00	1	3,8	3,8	84,6
	146,00	1	3,8	3,8	88,5
	149,00	1	3,8	3,8	92,3
	151,00	1	3,8	3,8	96,2
	152,00	1	3,8	3,8	100,0
	Total	26	100,0	100,0	

d1önöğrenmestratejileritop

		Frequency	Percent	Valid Percent	Cumulative Percent
Valid	205,00	1	3,8	3,8	3,8
	226,00	1	3,8	3,8	7,7
	229,00	1	3,8	3,8	11,5
	234,00	1	3,8	3,8	15,4
	239,00	1	3,8	3,8	19,2
	241,00	1	3,8	3,8	23,1
	246,00	1	3,8	3,8	26,9
	250,00	1	3,8	3,8	30,8
	255,00	1	3,8	3,8	34,6
	261,00	1	3,8	3,8	38,5
	263,00	1	3,8	3,8	42,3
	264,00	2	7,7	7,7	50,0
	271,00	1	3,8	3,8	53,8
	273,00	1	3,8	3,8	57,7
	275,00	1	3,8	3,8	61,5
	280,00	1	3,8	3,8	65,4
	286,00	2	7,7	7,7	73,1
	292,00	1	3,8	3,8	76,9
	297,00	1	3,8	3,8	80,8
	299,00	1	3,8	3,8	84,6
	316,00	1	3,8	3,8	88,5
	337,00	1	3,8	3,8	92,3
	341,00	1	3,8	3,8	96,2
	348,00	1	3,8	3,8	100,0
	Total	26	100,0	100,0	

d1sonişbirliktop

		Frequency	Percent	Valid Percent	Cumulative Percent
Valid	51,00	1	3,8	3,8	3,8
	54,00	1	3,8	3,8	7,7
	56,00	1	3,8	3,8	11,5
	61,00	1	3,8	3,8	15,4
	62,00	2	7,7	7,7	23,1
	68,00	1	3,8	3,8	26,9
	70,00	1	3,8	3,8	30,8
	71,00	3	11,5	11,5	42,3
	73,00	1	3,8	3,8	46,2
	74,00	1	3,8	3,8	50,0
	75,00	3	11,5	11,5	61,5
	76,00	2	7,7	7,7	69,2
	78,00	1	3,8	3,8	73,1
	82,00	1	3,8	3,8	76,9
	85,00	2	7,7	7,7	84,6
	86,00	2	7,7	7,7	92,3
	91,00	1	3,8	3,8	96,2
	94,00	1	3,8	3,8	100,0
	Total	26	100,0	100,0	

d1sondijitaltektop

		Frequency	Percent	Valid Percent	Cumulative Percent
Valid	112,00	1	3,8	3,8	3,8
	119,00	1	3,8	3,8	7,7

d1sondijitaltektop

		Frequency	Percent	Valid Percent	Cumulative Percent
	122,00	2	7,7	7,7	15,4
	125,00	2	7,7	7,7	23,1
	127,00	1	3,8	3,8	26,9
	128,00	2	7,7	7,7	34,6
	130,00	1	3,8	3,8	38,5
	132,00	2	7,7	7,7	46,2
	135,00	1	3,8	3,8	50,0
	137,00	2	7,7	7,7	57,7
	138,00	1	3,8	3,8	61,5
	139,00	2	7,7	7,7	69,2
	141,00	1	3,8	3,8	73,1
	145,00	2	7,7	7,7	80,8
	147,00	1	3,8	3,8	84,6
	148,00	1	3,8	3,8	88,5
	152,00	1	3,8	3,8	92,3
	154,00	1	3,8	3,8	96,2
	156,00	1	3,8	3,8	100,0
	Total	26	100,0	100,0	

d1sonöğrstratejitop

		Frequency	Percent	Valid Percent	Cumulative Percent
Valid	225,00	1	3,8	3,8	3,8
	239,00	1	3,8	3,8	7,7
	246,00	1	3,8	3,8	11,5
	255,00	1	3,8	3,8	15,4
	258,00	1	3,8	3,8	19,2
	259,00	1	3,8	3,8	23,1
	276,00	1	3,8	3,8	26,9
	279,00	1	3,8	3,8	30,8
	285,00	1	3,8	3,8	34,6
	290,00	1	3,8	3,8	38,5
	294,00	1	3,8	3,8	42,3
	295,00	1	3,8	3,8	46,2
	298,00	1	3,8	3,8	50,0
	301,00	1	3,8	3,8	53,8
	302,00	2	7,7	7,7	61,5
	303,00	1	3,8	3,8	65,4
	315,00	1	3,8	3,8	69,2
	317,00	1	3,8	3,8	73,1
	318,00	1	3,8	3,8	76,9
	319,00	1	3,8	3,8	80,8

d1sonöğrstratejitop

	Frequency	Percent	Valid Percent	Cumulative Percent
320,00	1	3,8	3,8	84,6
354,00	1	3,8	3,8	88,5
356,00	1	3,8	3,8	92,3
357,00	1	3,8	3,8	96,2
378,00	1	3,8	3,8	100,0
Total	26	100,0	100,0	

```
EXAMINE VARIABLES=d1önişbirlikçitop d1öndijitaltektop d1önöğrenmestratejileritop d1sonişbirl
    d1sonöğrstratejitop
  /PLOT BOXPLOT STEMLEAF NPPLOT
  /COMPARE GROUPS
  /STATISTICS DESCRIPTIVES
  /CINTERVAL 95
  /MISSING LISTWISE
  /NOTOTAL.
```

Explore

Notes

Output Created		03-NOV-2024 21:03:04
Comments		
Input	Data	C: \Users\Gizem\Pictures\NİHAL HOCAYLA ÇALIŞMA 2023\tersyüz-web ana analiz\tersyüz-web ana analiz.sav
	Active Dataset	DataSet1
	Filter	grup = 1 (FILTER)
	Weight	<none>
	Split File	<none>
	N of Rows in Working Data File	26
Missing Value Handling	Definition of Missing	User-defined missing values for dependent variables are treated as missing.
	Cases Used	Statistics are based on cases with no missing values for any dependent variable or factor used.

Notes

Syntax		EXAMINE VARIABLES=d1önişbirlikçitop d1öndijitaltektop d1önöğrenmestratejileritop d1sonişbirliktop d1sondijitaltektop d1sonöğrstratejitop /PLOT BOXPLOT STEMLEAF NPPLOT /COMPARE GROUPS /STATISTICS DESCRIPTIVES /CINTERVAL 95 /MISSING LISTWISE /NOTOTAL.
Resources	Processor Time	00:00:06,03
	Elapsed Time	00:00:04,06

```
[DataSet1] C:\Users\Gizem\Pictures\NİHAL HOCAYLA ÇALIŞMA 2023\tersyüz-web
ana analiz\tersyüz-web ana analiz.sav
```

Case Processing Summary

	Cases					
	Valid		Missing		Total	
	N	Percent	N	Percent	N	Percent
d1önişbirlikçitop	26	100,0%	0	0,0%	26	100,0%
d1öndijitaltektop	26	100,0%	0	0,0%	26	100,0%
d1önöğrenmestratejileritop	26	100,0%	0	0,0%	26	100,0%
d1sonişbirliktop	26	100,0%	0	0,0%	26	100,0%
d1sondijitaltektop	26	100,0%	0	0,0%	26	100,0%
d1sonöğrstratejitop	26	100,0%	0	0,0%	26	100,0%

Descriptives

			Statistic	Std. Error
d1önişbirlikçitop	Mean		64,4615	1,40025
	95% Confidence Interval for Mean	Lower Bound	61,5777	
		Upper Bound	67,3454	
	5% Trimmed Mean		64,5000	
	Median		64,5000	
	Variance		50,978	
	Std. Deviation		7,13992	
	Minimum		51,00	
	Maximum		77,00	
	Range		26,00	
	Interquartile Range		11,75	
	Skewness		-,162	,456
	Kurtosis		-,871	,887
d1öndijitaltektop	Mean		129,3846	2,71581
	95% Confidence Interval for Mean	Lower Bound	123,7913	
		Upper Bound	134,9779	
	5% Trimmed Mean		129,8761	
	Median		130,5000	
	Variance		191,766	
	Std. Deviation		13,84797	
	Minimum		94,00	
	Maximum		152,00	
	Range		58,00	
	Interquartile Range		18,00	
	Skewness		-,389	,456
	Kurtosis		,247	,887
d1önöğrenmestratejilerito p	Mean		272,2308	7,08678
	95% Confidence Interval for Mean	Lower Bound	257,6353	
		Upper Bound	286,8263	
	5% Trimmed Mean		271,5769	
	Median		267,5000	
	Variance		1305,785	
	Std. Deviation		36,13564	
	Minimum		205,00	
	Maximum		348,00	
	Range		143,00	
	Interquartile Range		48,50	
	Skewness		,477	,456
	Kurtosis		-,075	,887
d1sonişbirliktop	Mean		73,3846	2,18039
	95% Confidence Interval for Mean	Lower Bound	68,8940	
		Upper Bound	77,8752	
	5% Trimmed Mean		73,4829	

Descriptives

			Statistic	Std. Error
	Median		74,5000	
	Variance		123,606	
	Std. Deviation		11,11783	
	Minimum		51,00	
	Maximum		94,00	
	Range		43,00	
	Interquartile Range		16,25	
	Skewness		-,211	,456
	Kurtosis		-,361	,887
d1sondijitaltektop	Mean		135,1923	2,22435
	95% Confidence Interval for Mean	Lower Bound	130,6112	
		Upper Bound	139,7735	
	5% Trimmed Mean		135,2607	
	Median		136,0000	
	Variance		128,642	
	Std. Deviation		11,34203	
	Minimum		112,00	
	Maximum		156,00	
	Range		44,00	
	Interquartile Range		18,50	
	Skewness		,043	,456
	Kurtosis		-,600	,887
d1sonöğrstratejitop	Mean		297,7308	7,44282
	95% Confidence Interval for Mean	Lower Bound	282,4020	
		Upper Bound	313,0595	
	5% Trimmed Mean		297,4017	
	Median		299,5000	
	Variance		1440,285	
	Std. Deviation		37,95108	
	Minimum		225,00	
	Maximum		378,00	
	Range		153,00	
	Interquartile Range		46,50	
	Skewness		,158	,456
	Kurtosis		-,166	,887

Tests of Normality

	Kolmogorov-Smirnov[a]			Shapiro-Wilk		
	Statistic	df	Sig.	Statistic	df	Sig.
d1önişbirlikçitop	,111	26	,200*	,970	26	,620
d1öndijitaltektop	,074	26	,200*	,973	26	,700
d1önöğrenmestratejilerito p	,090	26	,200*	,968	26	,566
d1sonişbirliktop	,111	26	,200*	,972	26	,663
d1sondijitaltektop	,083	26	,200*	,983	26	,935
d1sonöğrstratejitop	,125	26	,200*	,972	26	,685

*. This is a lower bound of the true significance.

a. Lilliefors Significance Correction

d1önişbirlikçitop

```
d1önişbirlikçitop Stem-and-Leaf Plot

 Frequency    Stem &  Leaf

     3,00        5 .  144
     4,00        5 .  5679
     6,00        6 .  033344
     6,00        6 .  556899
     5,00        7 .  01233
     2,00        7 .  57

 Stem width:      10,00
 Each leaf:        1 case(s)
```

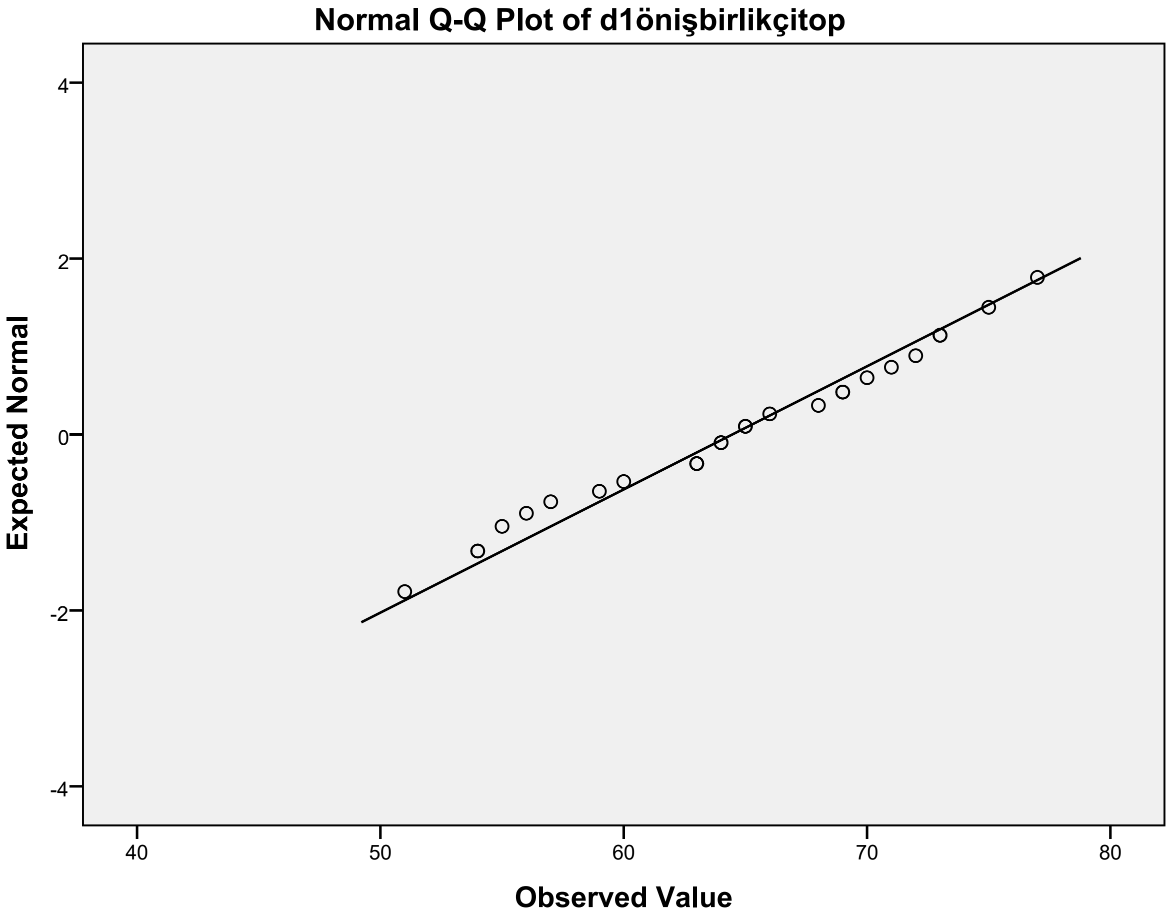
Normal Q-Q Plot of d1önişbirlikçitop
Expected Normal
4
2
0
-2
-4
40
50
60
70
80
Observed Value

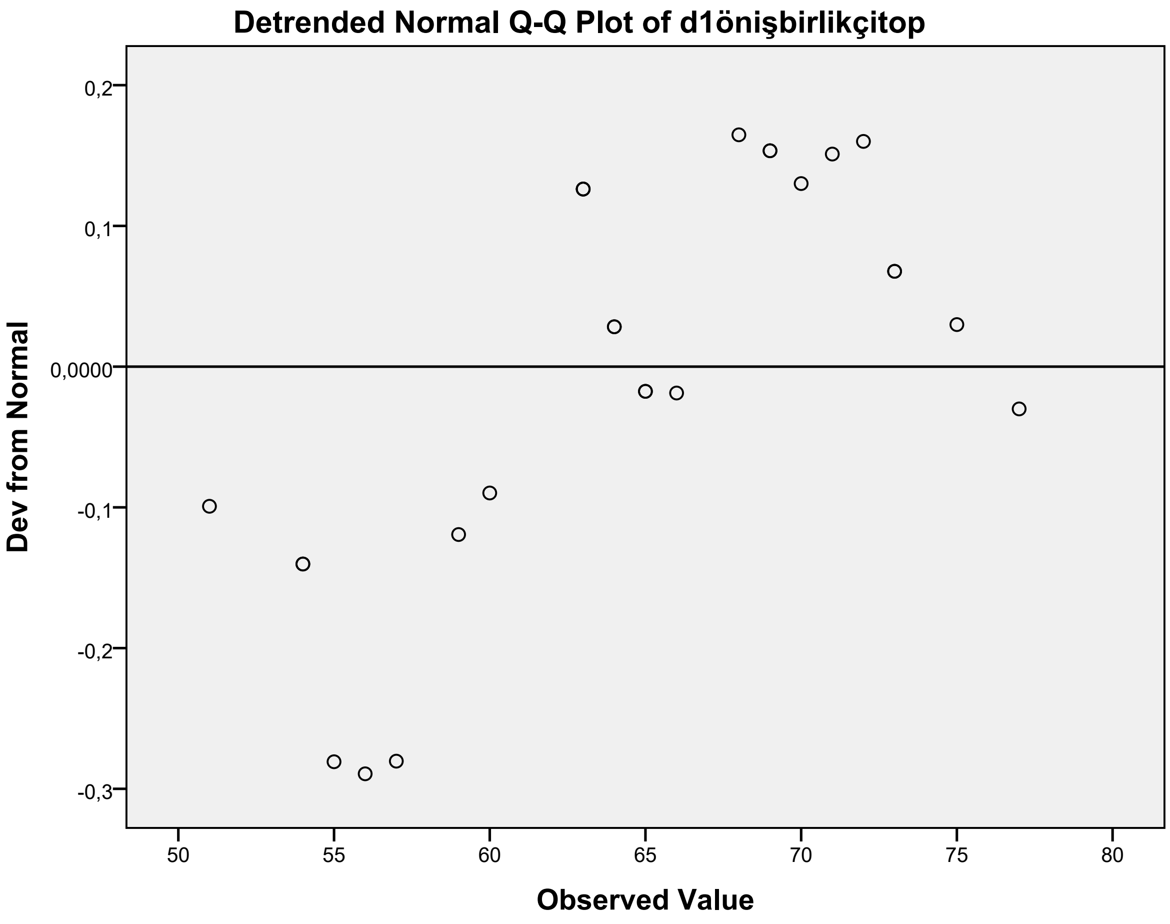

Detrended Normal Q-Q Plot of d1önişbirlikçitop
Dev from Normal
0,2
0,1
0,0000
-0,1
-0,2
-0,3
50
55
60
65
70
75
80
Observed Value

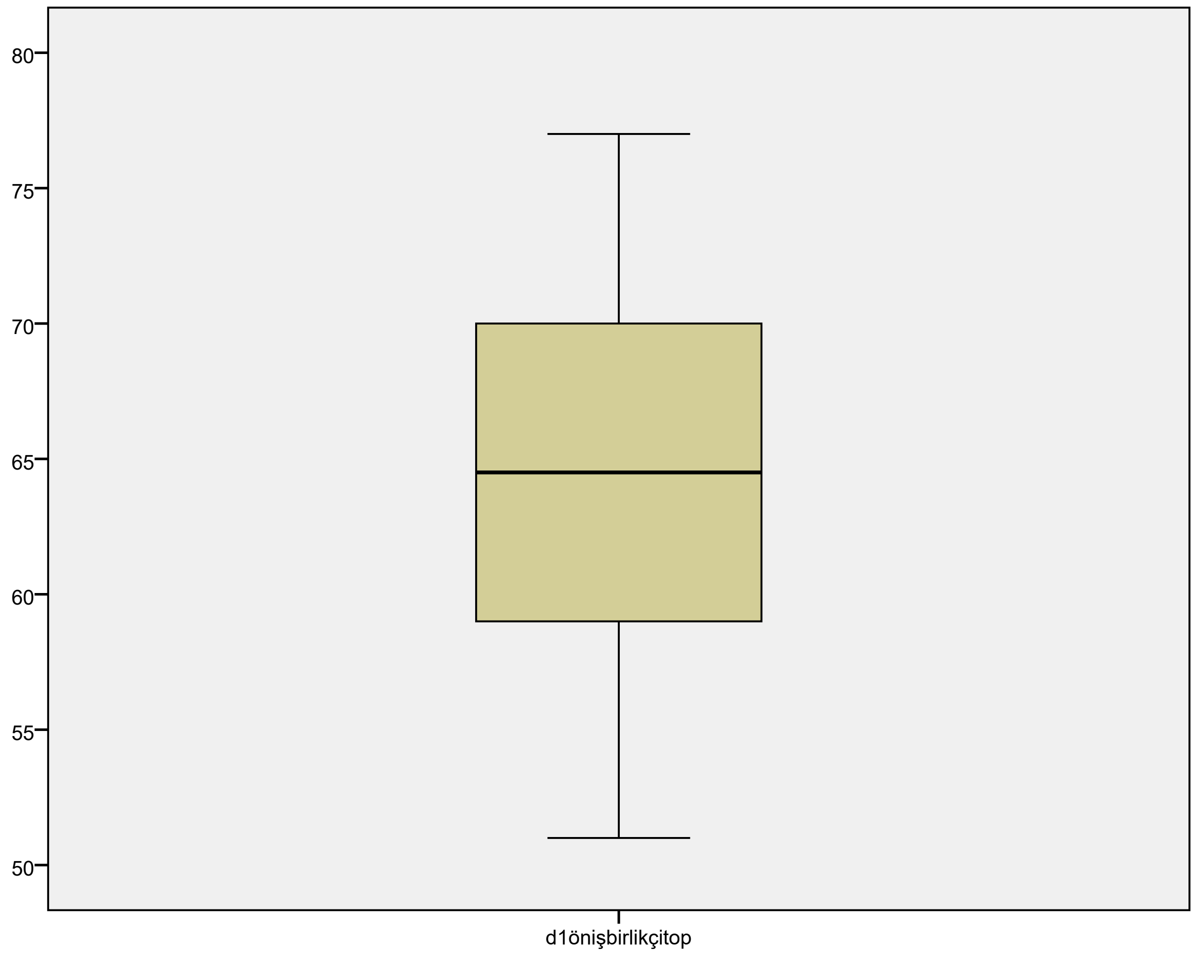

d1öndijitaltektop

```
d1öndijitaltektop Stem-and-Leaf Plot

 Frequency    Stem &  Leaf

     1,00 Extremes    (=<94)
     3,00       11 .  223
     1,00       11 .  7
     6,00       12 .  013334
     1,00       12 .  7
     5,00       13 .  01334
     3,00       13 .  778
     2,00       14 .  13
     2,00       14 .  69
     2,00       15 .  12

 Stem width:     10,00
 Each leaf:       1 case(s)
```

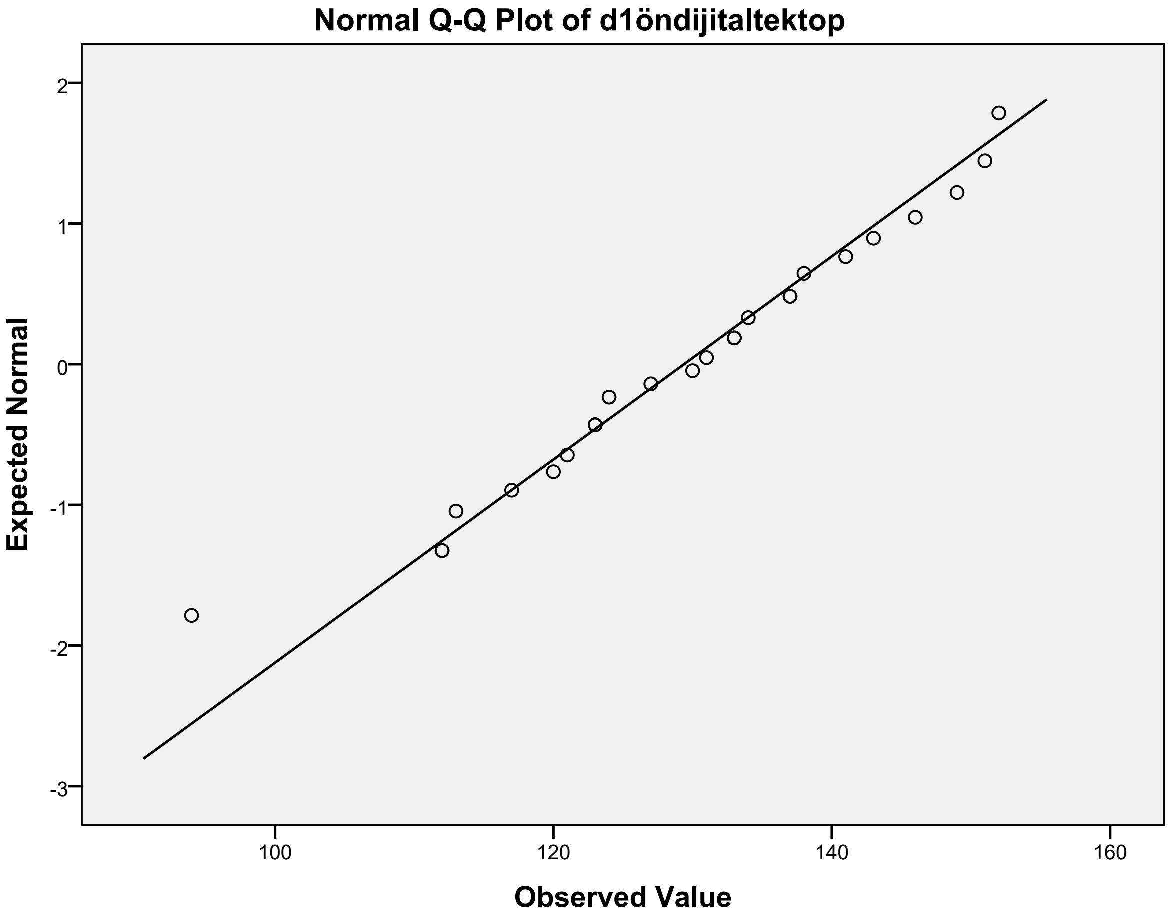
Normal Q-Q Plot of d1öndijitaltektop
Expected Normal
2
1
0
-1
-2
-3
100
120
140
160
Observed Value

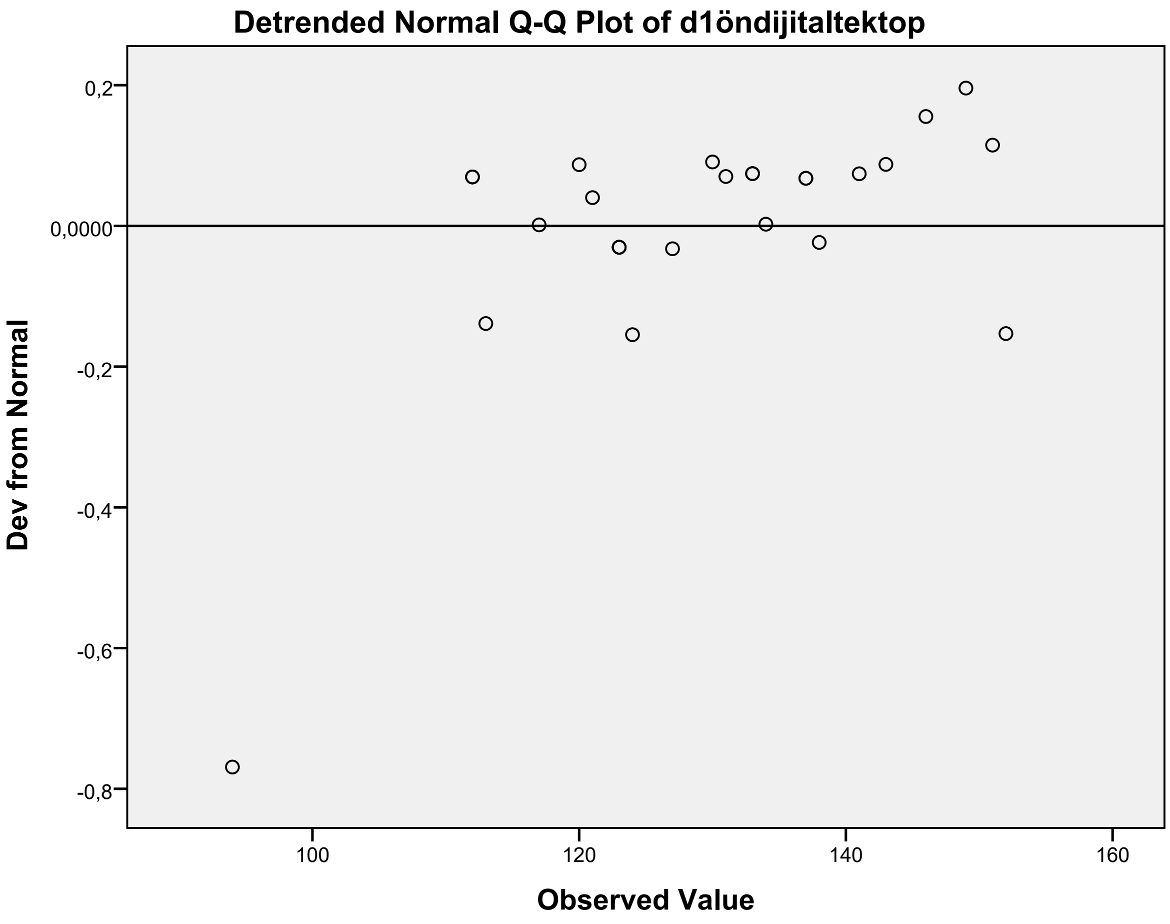
Detrended Normal Q-Q Plot of d1öndijitaltektop
Dev from Normal
0,2
0,0000
-0,2
-0,4
-0,6
-0,8
100
120
140
160
Observed Value

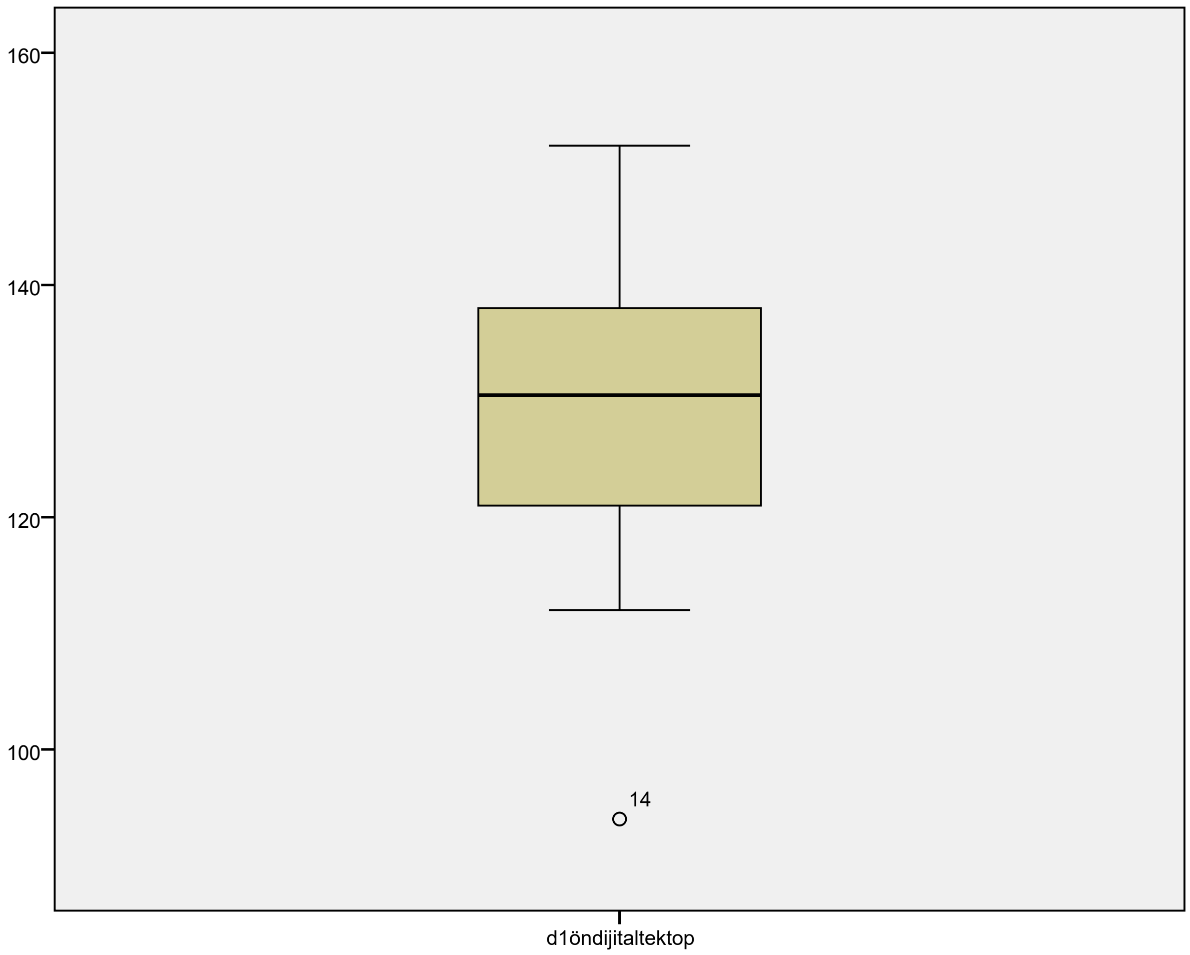

d1önöğrenmestratejileritop

```
d1önöğrenmestratejileritop Stem-and-Leaf Plot

 Frequency    Stem &  Leaf

     1,00        2 .  0
     4,00        2 .  2233
     4,00        2 .  4455
     7,00        2 .  6666777
     6,00        2 .  888999
     1,00        3 .  1
     1,00        3 .  3
     2,00        3 .  44

 Stem width:    100,00
 Each leaf:       1 case(s)
```

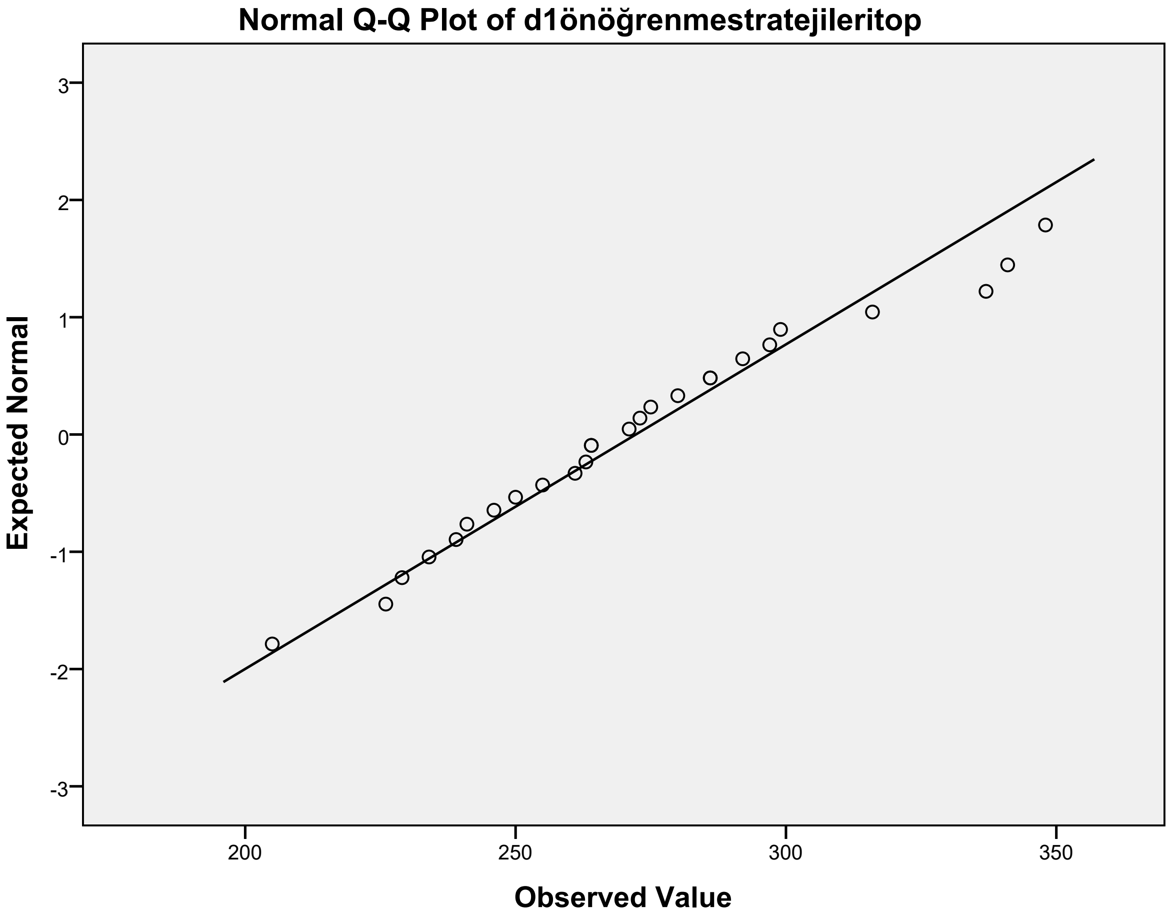
Normal Q-Q Plot of d1önöğrenmestratejileritop
Expected Normal
3
2
1
0
-1
-2
-3
200
250
300
350
Observed Value

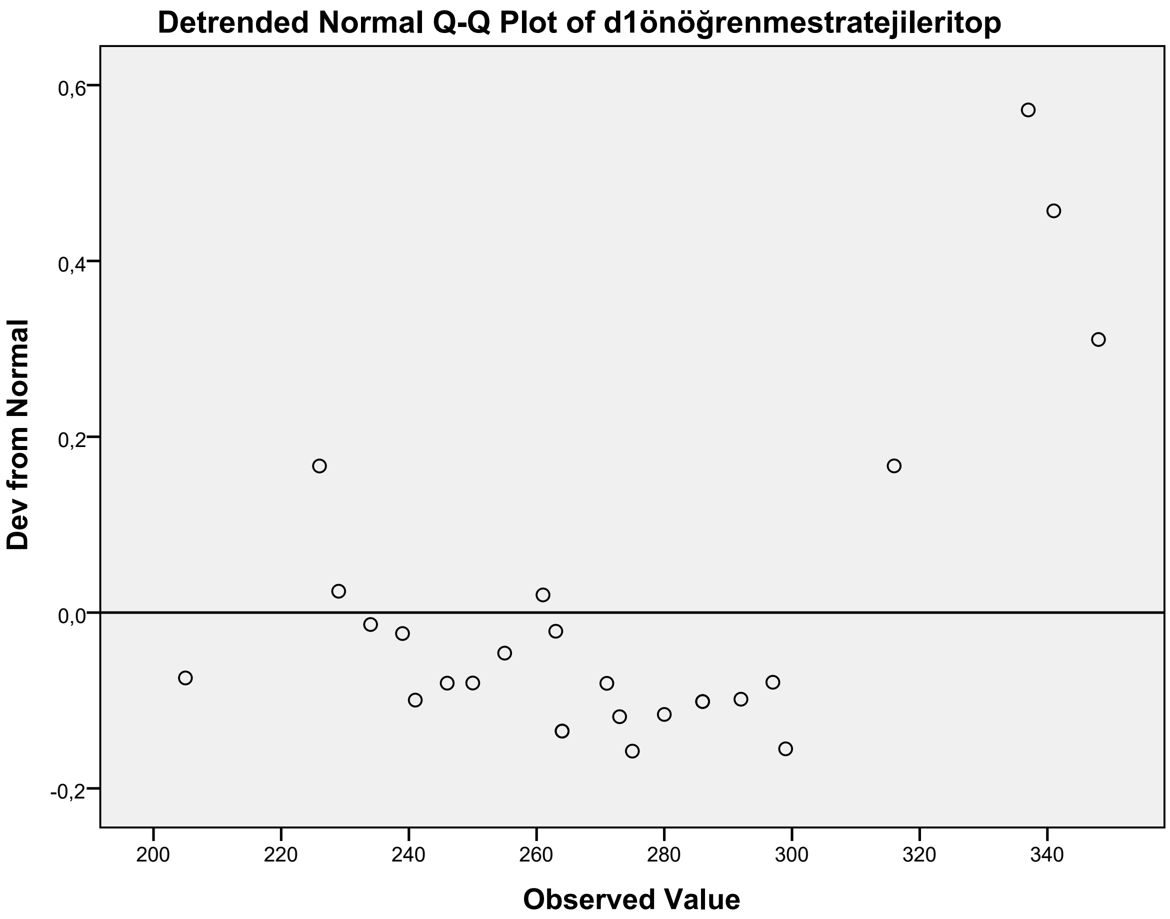
Detrended Normal Q-Q Plot of d1önöğrenmestratejileritop
Dev from Normal
0,6
0,4
0,2
0,0
-0,2
200
220
240
260
280
300
320
340
Observed Value

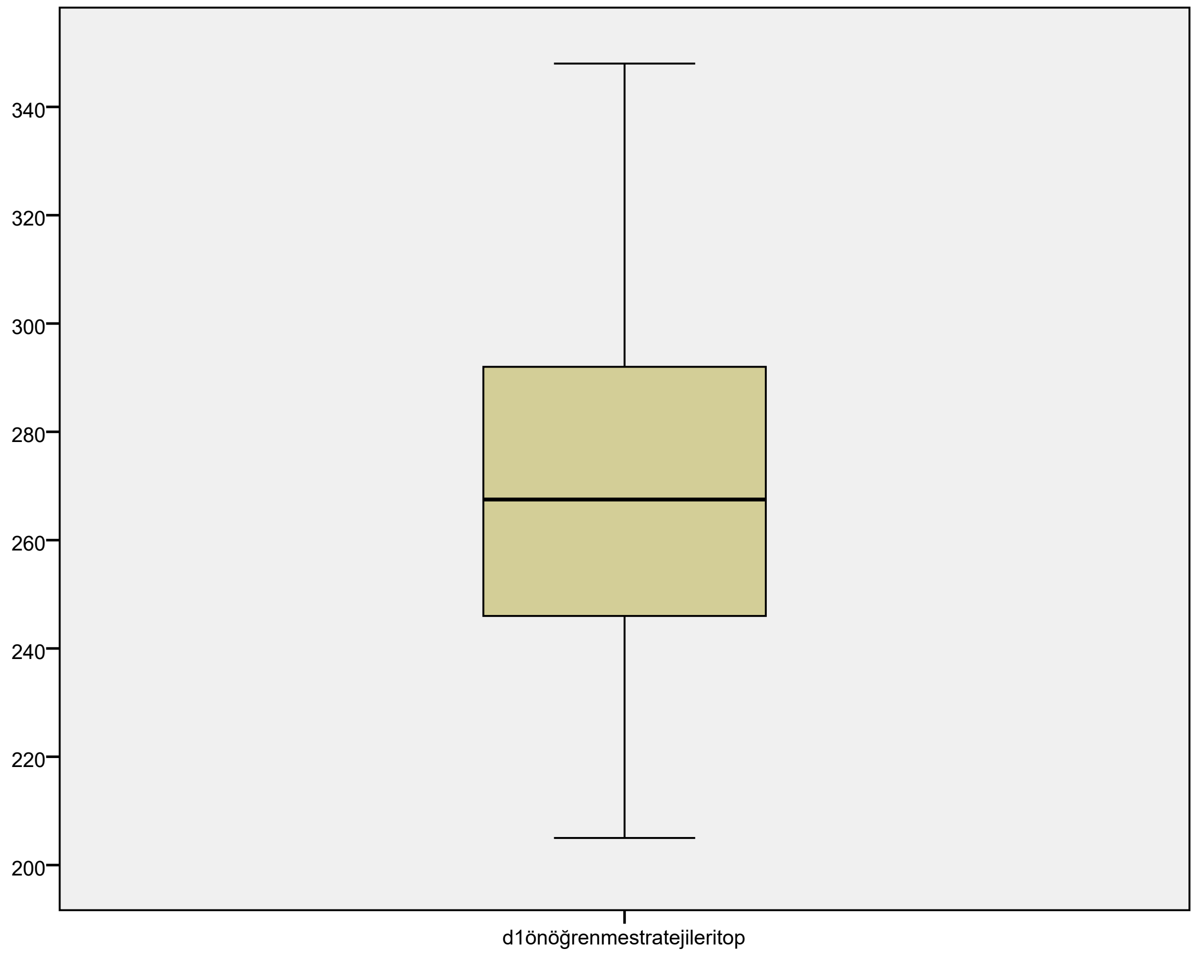

d1sonişbirliktop

```
d1sonişbirliktop Stem-and-Leaf Plot

 Frequency    Stem &  Leaf

     2,00        5 .  14
     1,00        5 .  6
     3,00        6 .  122
     1,00        6 .  8
     6,00        7 .  011134
     6,00        7 .  555668
     1,00        8 .  2
     4,00        8 .  5566
     2,00        9 .  14

 Stem width:      10,00
 Each leaf:        1 case(s)
```

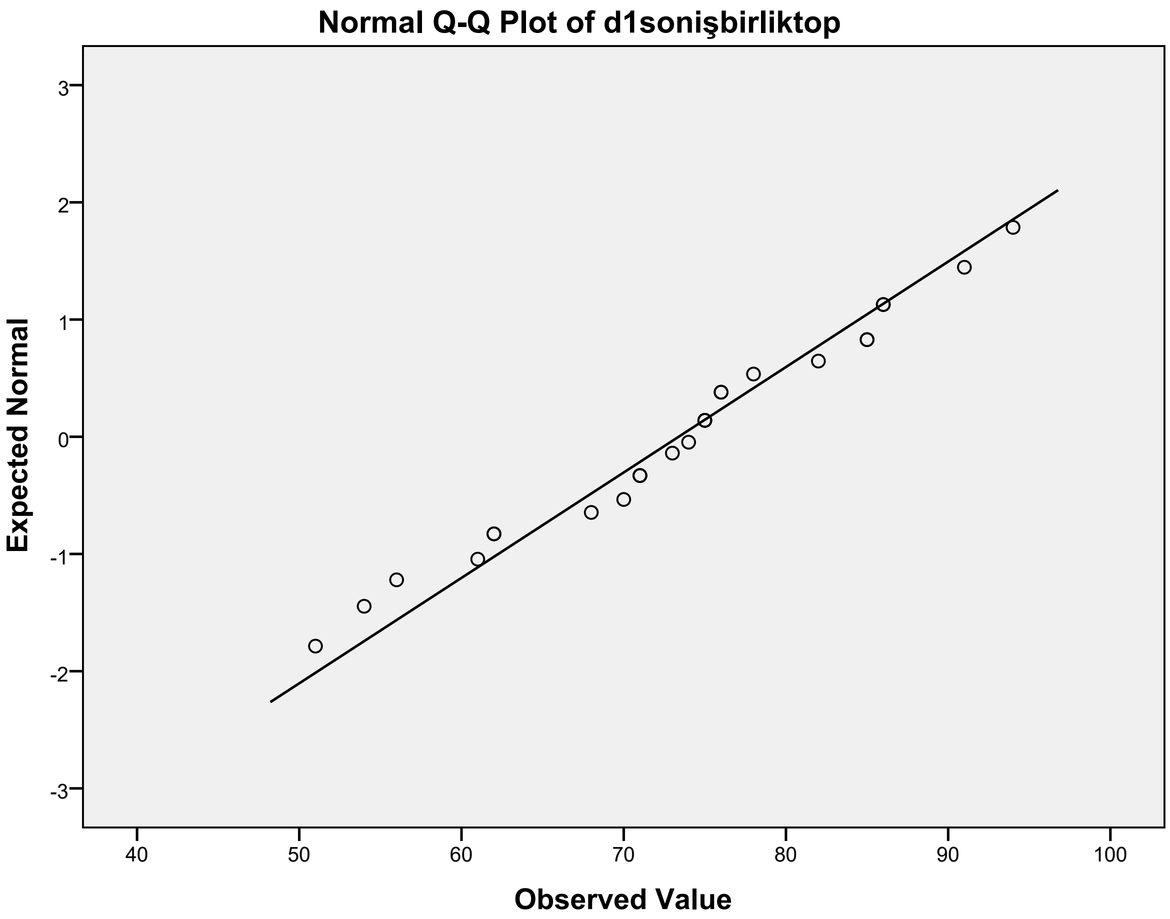
Normal Q-Q Plot of d1sonişbirliktop
Expected Normal
3
2
1
0
-1
-2
-3
40
50
60
70
80
90
100
Observed Value

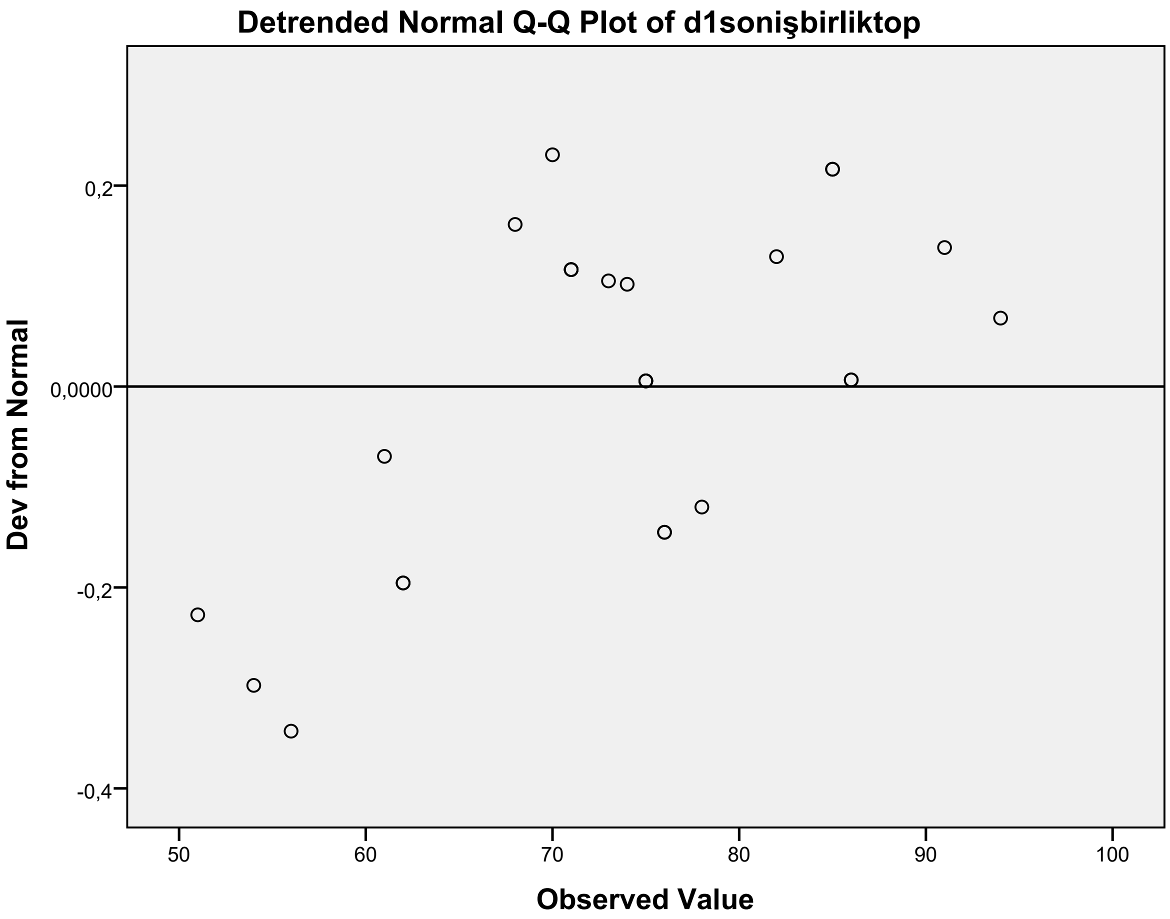
Detrended Normal Q-Q Plot of d1sonişbirliktop
Dev from Normal
0,2
0,0000
-0,2
-0,4
50
60
70
80
90
100
Observed Value

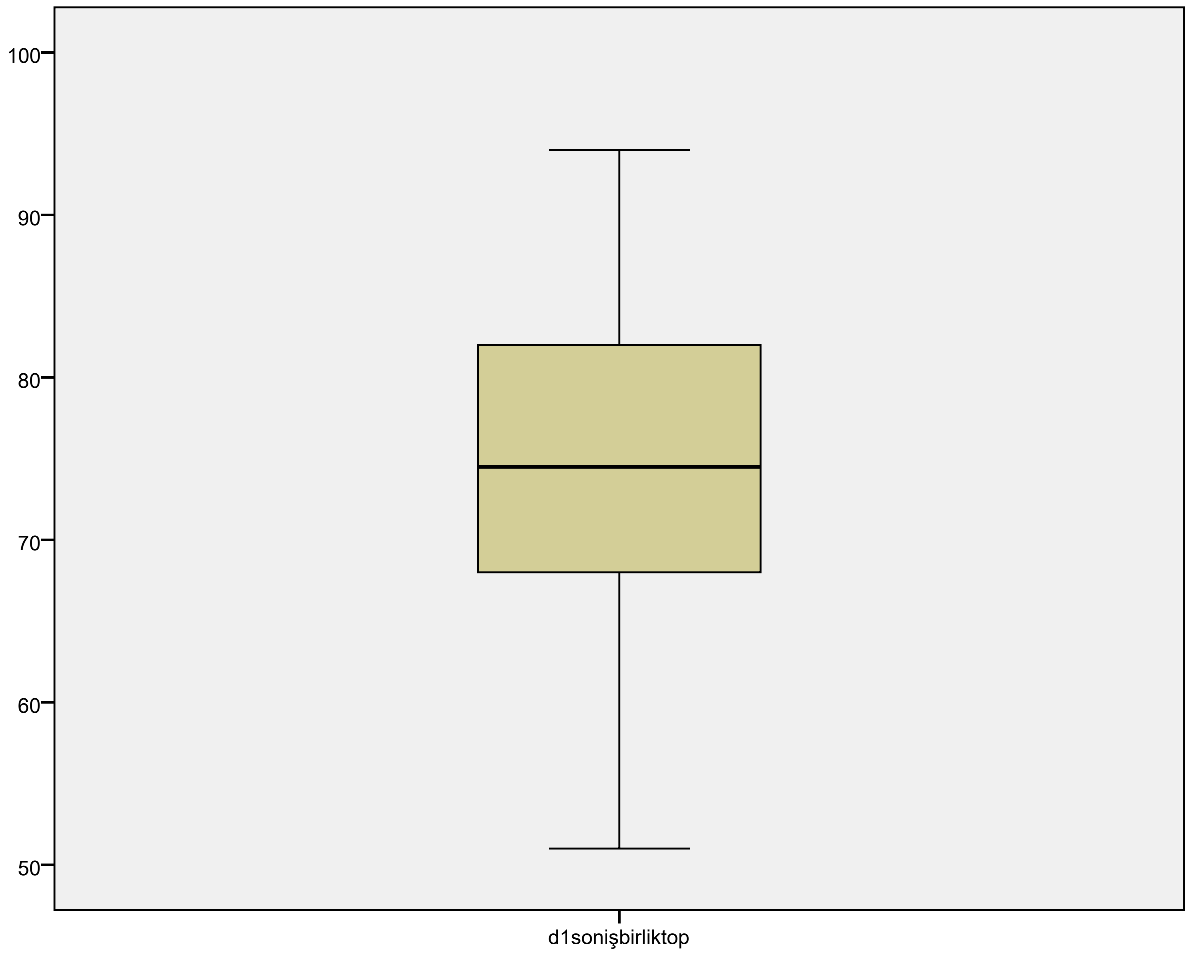

d1sondijitaltektop

```
d1sondijitaltektop Stem-and-Leaf Plot

 Frequency    Stem &  Leaf

     1,00       11 .  2
     1,00       11 .  9
     2,00       12 .  22
     5,00       12 .  55788
     3,00       13 .  022
     6,00       13 .  577899
     1,00       14 .  1
     4,00       14 .  5578
     2,00       15 .  24
     1,00       15 .  6

 Stem width:     10,00
 Each leaf:        1 case(s)
```

Normal Q-Q Plot of d1sondijitaltektop

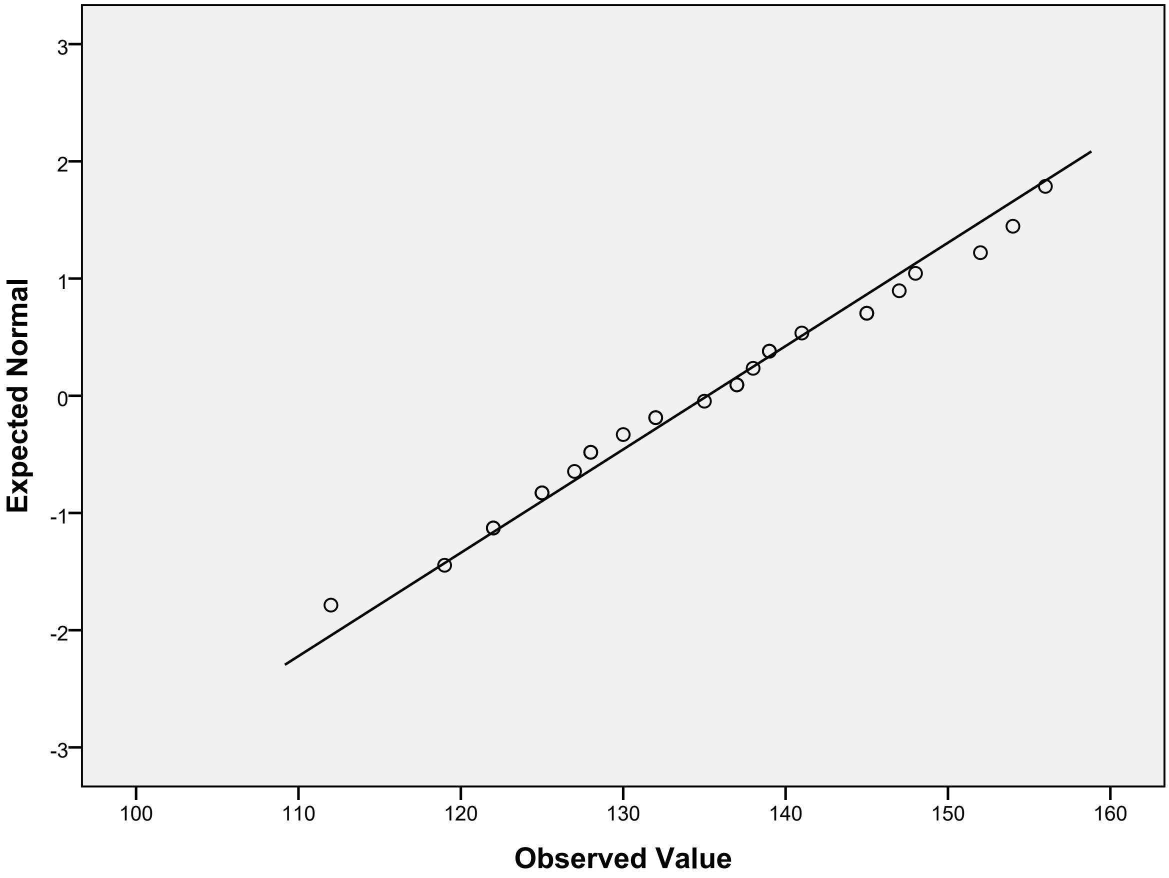

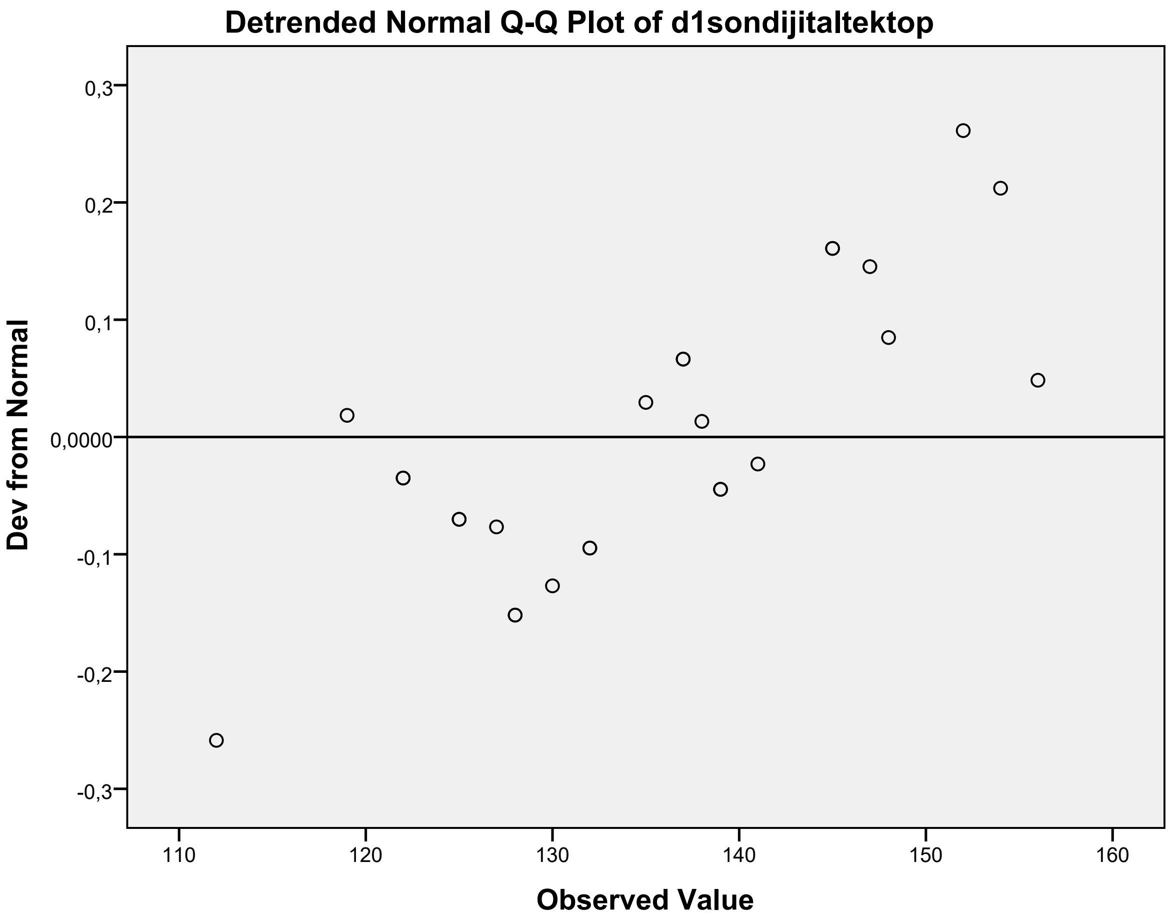
Detrended Normal Q-Q Plot of d1sondijitaltektop
Dev from Normal
0,3
0,2
0,1
0,0000
-0,1
-0,2
-0,3
110
120
130
140
150
160
Observed Value

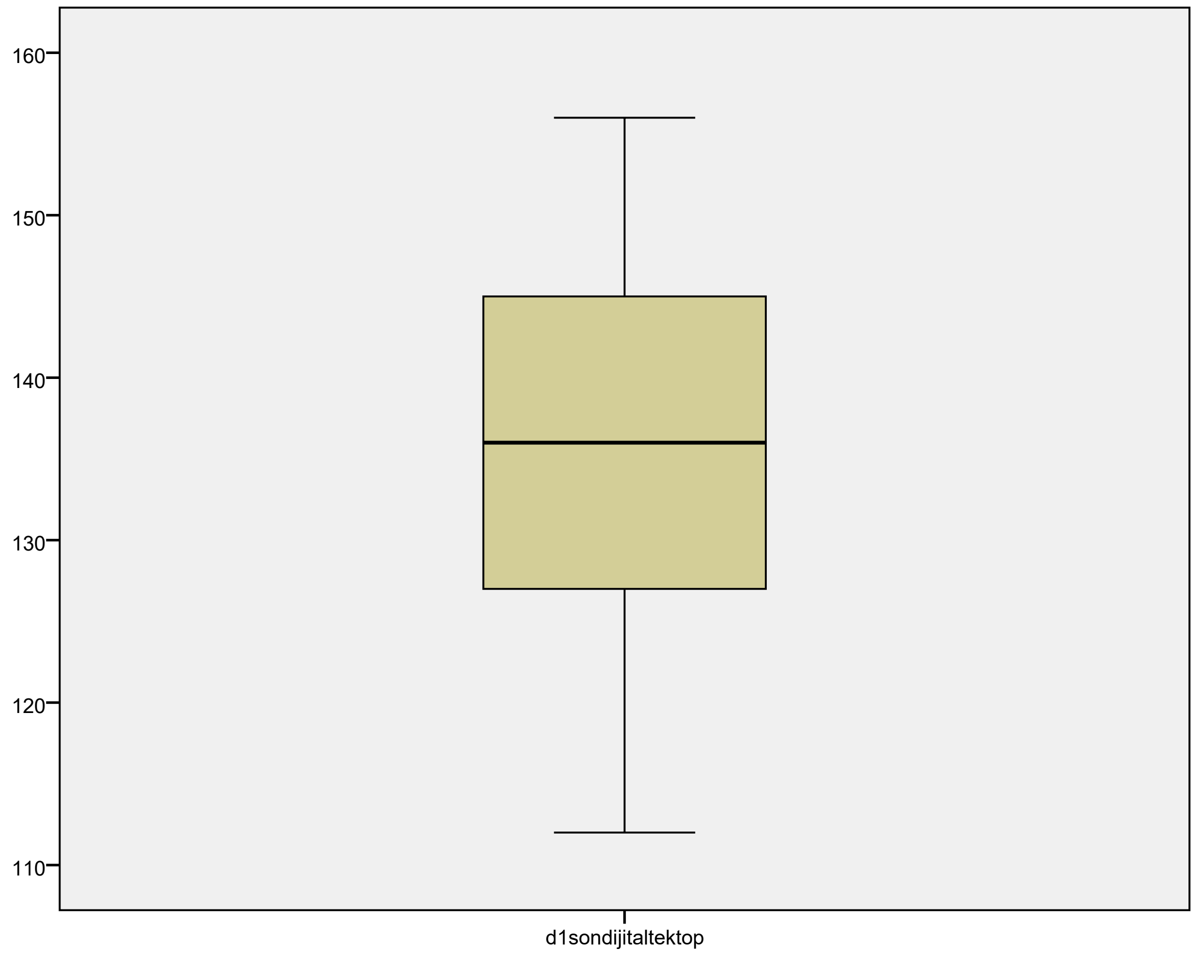

d1sonöğrstratejitop

```
d1sonöğrstratejitop Stem-and-Leaf Plot

 Frequency    Stem &  Leaf

     2,00        2 .  23
     4,00        2 .  4555
     2,00        2 .  77
     5,00        2 .  89999
     8,00        3 .  00001111
     1,00        3 .  2
     3,00        3 .  555
     1,00        3 .  7

 Stem width:    100,00
 Each leaf:       1 case(s)
```

Normal Q-Q Plot of d1sonöğrstratejitop

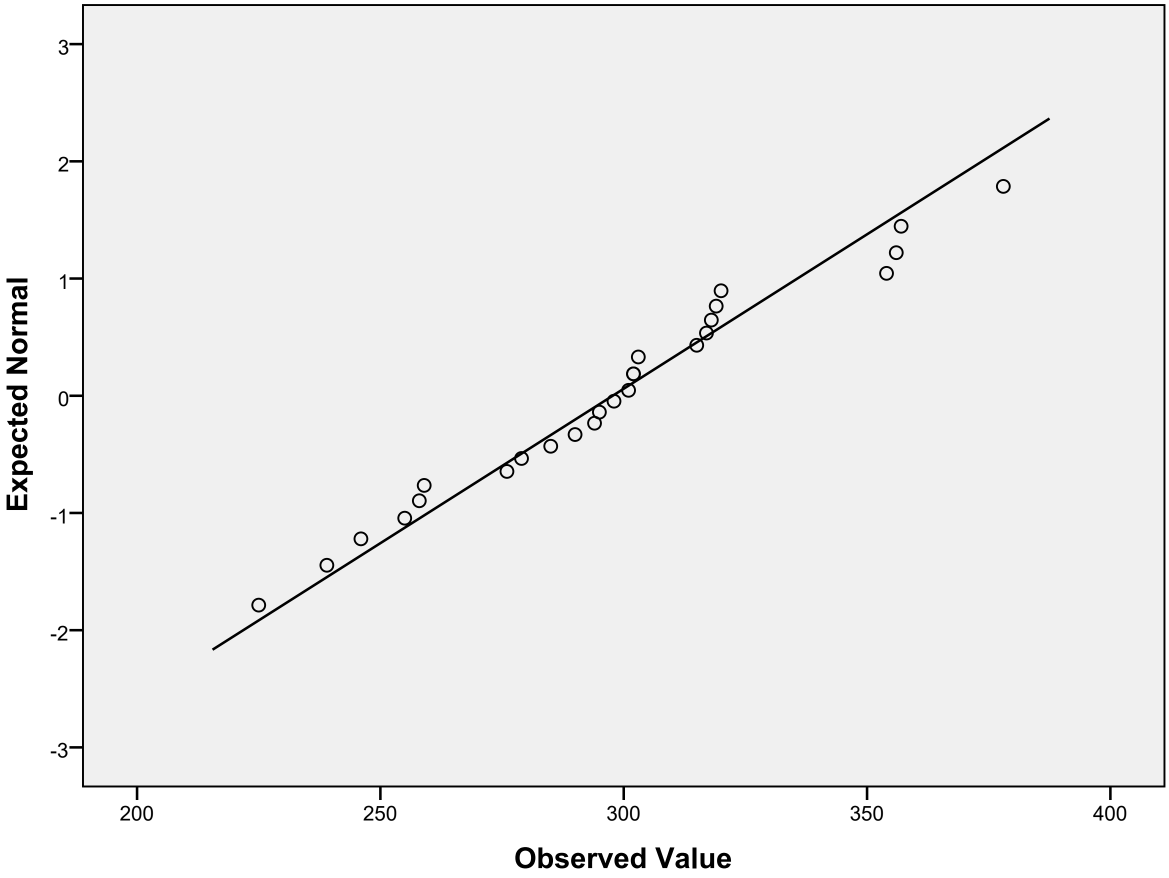

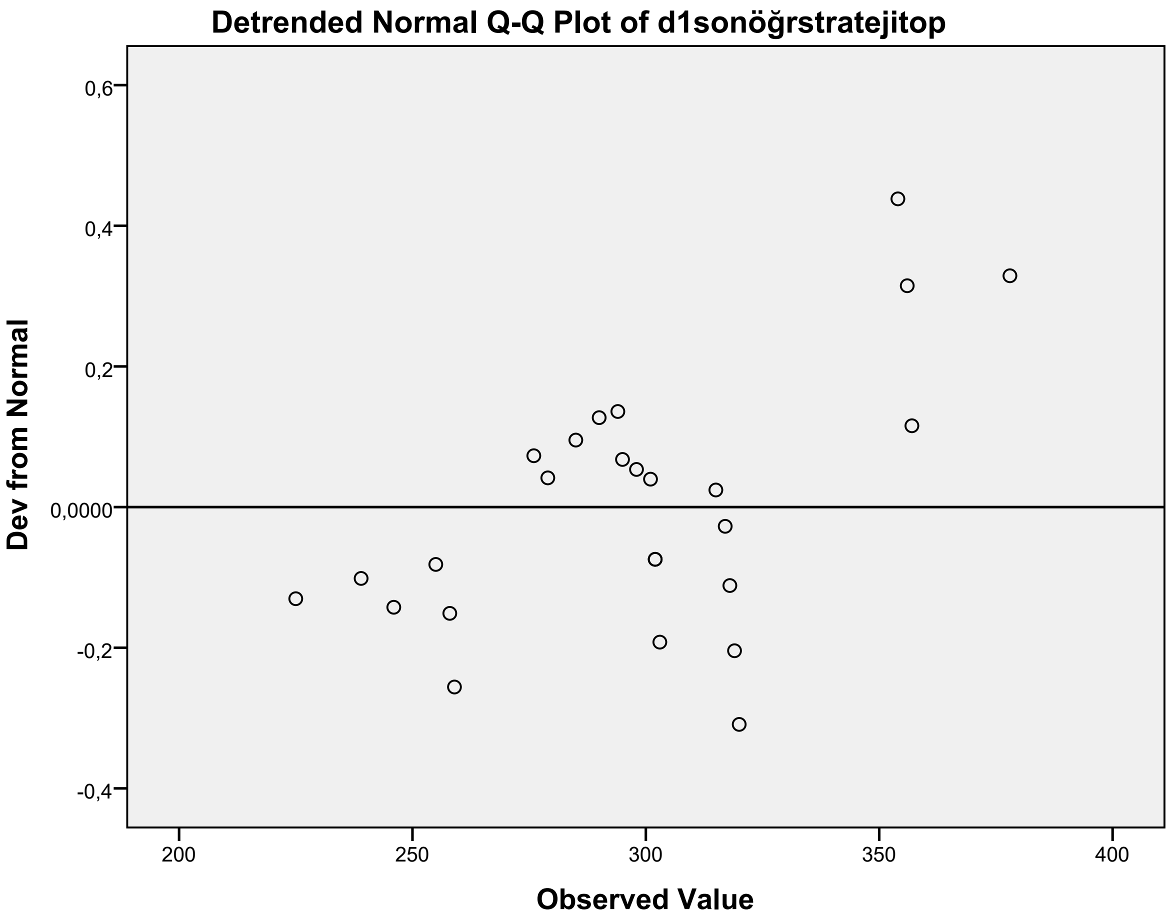
Detrended Normal Q-Q Plot of d1sonöğrstratejitop
Dev from Normal
0,6
0,4
0,2
0,0000
-0,2
-0,4
200
250
300
350
400
Observed Value

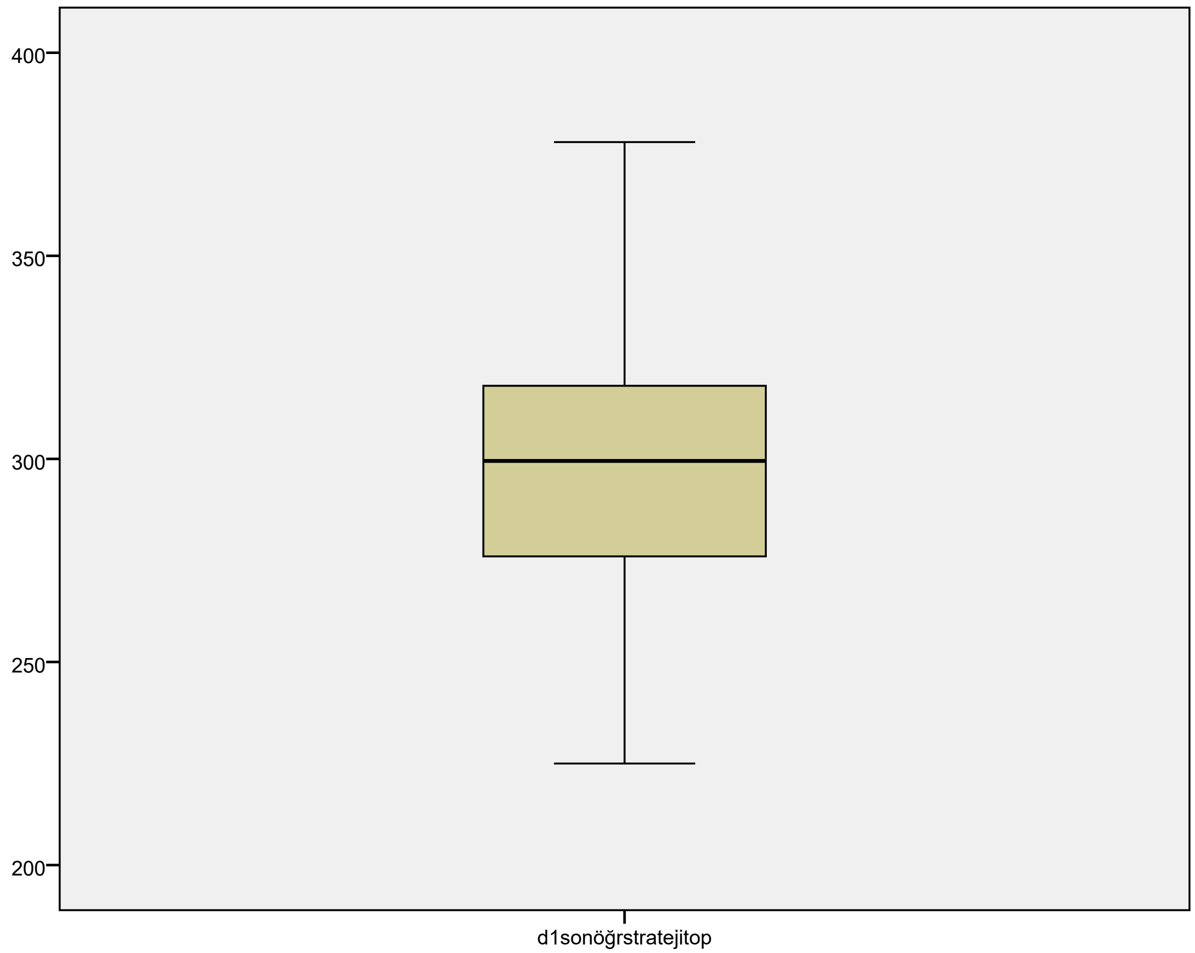


```
 USE ALL.
COMPUTE filter_$=(grup = 2).
VARIABLE LABELS filter_$ 'grup = 2 (FILTER)'.
VALUE LABELS filter_$ 0 'Not Selected' 1 'Selected'.
FORMATS filter_$ (f1.0).
FILTER BY filter_$.
EXECUTE.
EXAMINE VARIABLES=d1önişbirlikçitop d1öndijitaltektop d1önöğrenmestratejileritop d1sonişbirl
    d1sonöğrstratejitop
  /PLOT BOXPLOT STEMLEAF NPPLOT
  /COMPARE GROUPS
  /STATISTICS DESCRIPTIVES
  /CINTERVAL 95
  /MISSING LISTWISE
  /NOTOTAL.
```

Explore

Notes

Output Created		03-NOV-2024 21:04:13
Comments		
Input	Data	C: \Users\Gizem\Pictures\NİHAL HOCAYLA ÇALIŞMA 2023\tersyüz-web ana analiz\tersyüz-web ana analiz.sav
	Active Dataset	DataSet1
	Filter	grup = 2 (FILTER)
	Weight	<none>
	Split File	<none>
	N of Rows in Working Data File	24
Missing Value Handling	Definition of Missing	User-defined missing values for dependent variables are treated as missing.
	Cases Used	Statistics are based on cases with no missing values for any dependent variable or factor used.
Syntax		EXAMINE VARIABLES=d1önişbirlikçitop d1öndijitaltektop d1önöğrenmestratejileritop d1sonişbirliktop d1sondijitaltektop d1sonöğrstratejitop /PLOT BOXPLOT STEMLEAF NPPLOT /COMPARE GROUPS /STATISTICS DESCRIPTIVES /CINTERVAL 95 /MISSING LISTWISE /NOTOTAL.
Resources	Processor Time	00:00:02,23
	Elapsed Time	00:00:02,11

[DataSet1] C:\Users\Gizem\Pictures\NİHAL HOCAYLA ÇALIŞMA 2023\tersyüz-web ana analiz\tersyüz-web ana analiz.sav

Case Processing Summary

	Cases					
	Valid		Missing		Total	
	N	Percent	N	Percent	N	Percent
d1önişbirlikçitop	24	100,0%	0	0,0%	24	100,0%
d1öndijitaltektop	24	100,0%	0	0,0%	24	100,0%
d1önöğrenmestratejilerito p	24	100,0%	0	0,0%	24	100,0%
d1sonişbirliktop	24	100,0%	0	0,0%	24	100,0%
d1sondijitaltektop	24	100,0%	0	0,0%	24	100,0%
d1sonöğrstratejitop	24	100,0%	0	0,0%	24	100,0%

Descriptives

			Statistic	Std. Error
d1önişbirlikçitop	Mean		61,7083	1,92474
	95% Confidence Interval for Mean	Lower Bound	57,7267	
		Upper Bound	65,6900	
	5% Trimmed Mean		62,3056	
	Median		63,0000	
	Variance		88,911	
	Std. Deviation		9,42928	
	Minimum		36,00	
	Maximum		75,00	
	Range		39,00	
	Interquartile Range		13,75	
	Skewness		-,914	,472
	Kurtosis		,903	,918
d1öndijitaltektop	Mean		129,5000	1,67462
	95% Confidence Interval for Mean	Lower Bound	126,0358	
		Upper Bound	132,9642	
	5% Trimmed Mean		129,2222	
	Median		127,5000	
	Variance		67,304	
	Std. Deviation		8,20392	
	Minimum		116,00	
	Maximum		149,00	
	Range		33,00	
	Interquartile Range		12,25	
	Skewness		,623	,472
	Kurtosis		-,011	,918
d1önöğrenmestratejilerito p	Mean		262,4583	4,93251
	95% Confidence Interval for Mean	Lower Bound	252,2547	
		Upper Bound	272,6620	
	5% Trimmed Mean		263,2593	
	Median		259,5000	

Descriptives

			Statistic	Std. Error
	Variance		583,911	
	Std. Deviation		24,16426	
	Minimum		202,00	
	Maximum		303,00	
	Range		101,00	
	Interquartile Range		36,25	
	Skewness		-,153	,472
	Kurtosis		,355	,918
d1sonişbirliktop	Mean		64,2917	2,68212
	95% Confidence Interval for Mean	Lower Bound	58,7433	
		Upper Bound	69,8401	
	5% Trimmed Mean		64,7037	
	Median		67,0000	
	Variance		172,650	
	Std. Deviation		13,13965	
	Minimum		36,00	
	Maximum		86,00	
	Range		50,00	
	Interquartile Range		15,25	
	Skewness		-,852	,472
	Kurtosis		,354	,918
d1sondijitaltektop	Mean		144,5833	2,96227
	95% Confidence Interval for Mean	Lower Bound	138,4554	
		Upper Bound	150,7113	
	5% Trimmed Mean		143,8148	
	Median		144,0000	
	Variance		210,601	
	Std. Deviation		14,51211	
	Minimum		120,00	
	Maximum		184,00	
	Range		64,00	
	Interquartile Range		14,25	
	Skewness		,923	,472
	Kurtosis		1,785	,918
d1sonöğrstratejitop	Mean		280,4583	6,11958
	95% Confidence Interval for Mean	Lower Bound	267,7990	
		Upper Bound	293,1176	
	5% Trimmed Mean		281,3333	
	Median		282,0000	
	Variance		898,781	
	Std. Deviation		29,97967	
	Minimum		206,00	
	Maximum		333,00	

Descriptives

		Statistic	Std. Error
	Range	127,00	
	Interquartile Range	45,75	
	Skewness	-,303	,472
	Kurtosis	,209	,918

Tests of Normality

	Kolmogorov-Smirnov[a]			Shapiro-Wilk		
	Statistic	df	Sig.	Statistic	df	Sig.
d1önişbirlikçitop	,138	24	,200*	,943	24	,193
d1öndijitaltektop	,156	24	,136	,950	24	,266
d1önöğrenmestratejileritop	,116	24	,200*	,953	24	,309
d1sonişbirliktop	,158	24	,126	,926	24	,077
d1sondijitaltektop	,169	24	,073	,924	24	,071
d1sonöğrstratejitop	,116	24	,200*	,971	24	,685

*. This is a lower bound of the true significance.

a. Lilliefors Significance Correction

d1önişbirlikçitop

```
d1önişbirlikçitop Stem-and-Leaf Plot

 Frequency    Stem &  Leaf

     1,00        3 .  6
     1,00        4 .  7
     6,00        5 .  014569
    11,00        6 .  11333356899
     5,00        7 .  01245

 Stem width:      10,00
 Each leaf:        1 case(s)
```

Normal Q-Q Plot of d1önişbirlikçitop

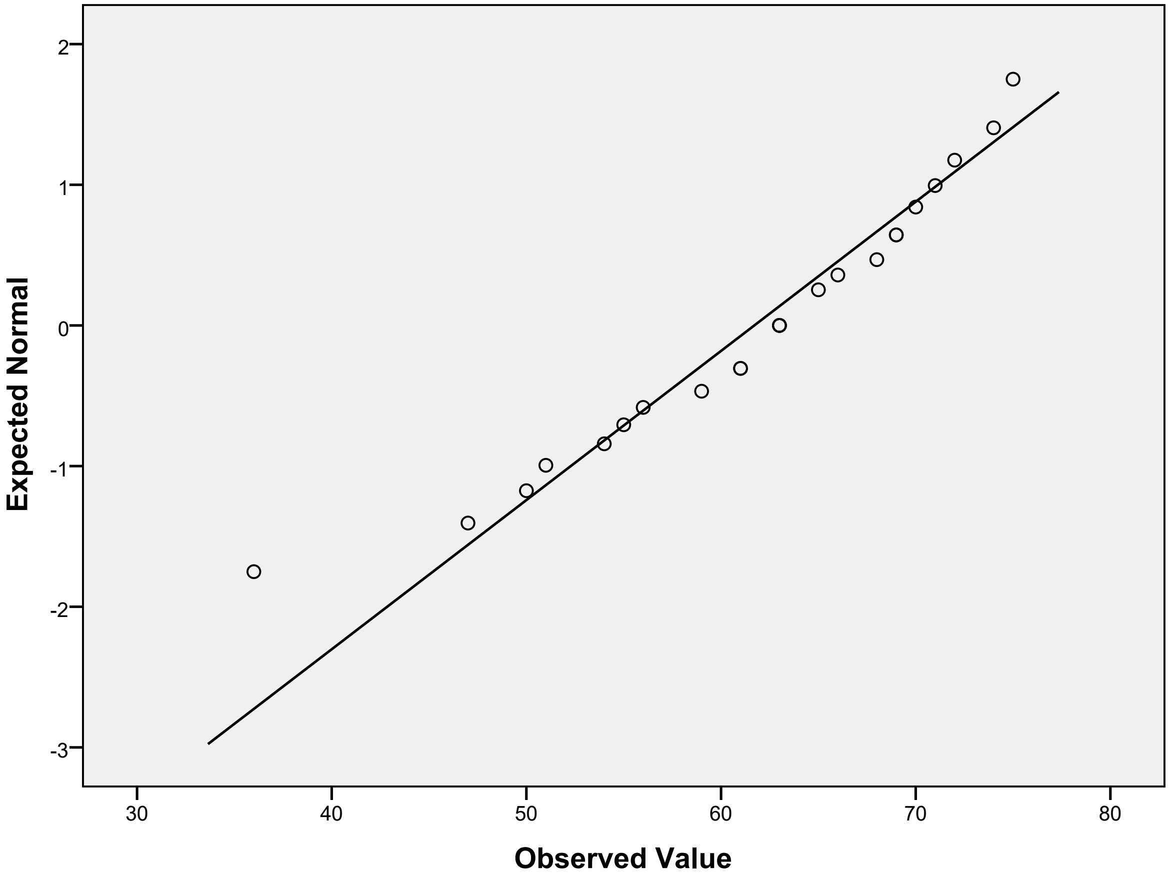

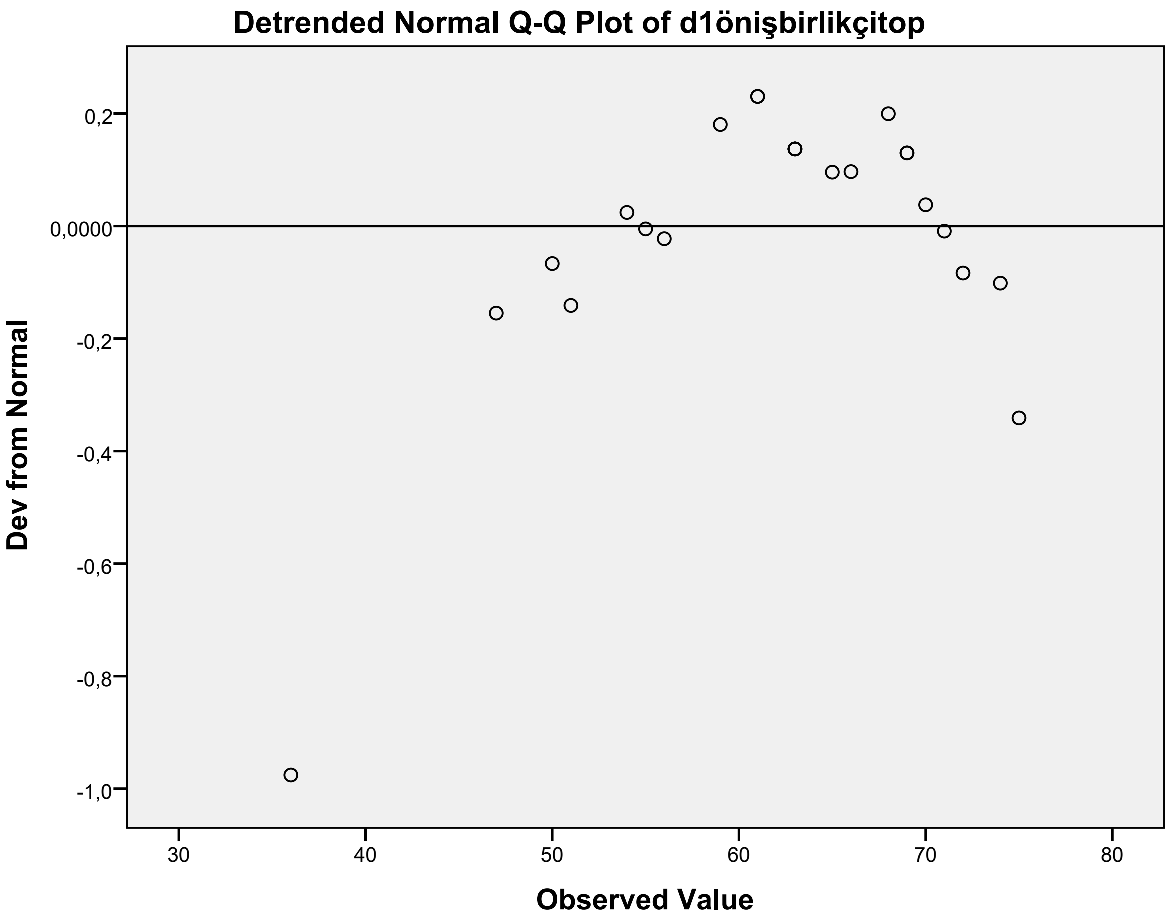
Detrended Normal Q-Q Plot of d1önişbirlikçitop
0,2
0,0000
-0,2
-0,4
-0,6
-0,8
-1,0
Dev from Normal
30
40
50
60
70
80
Observed Value

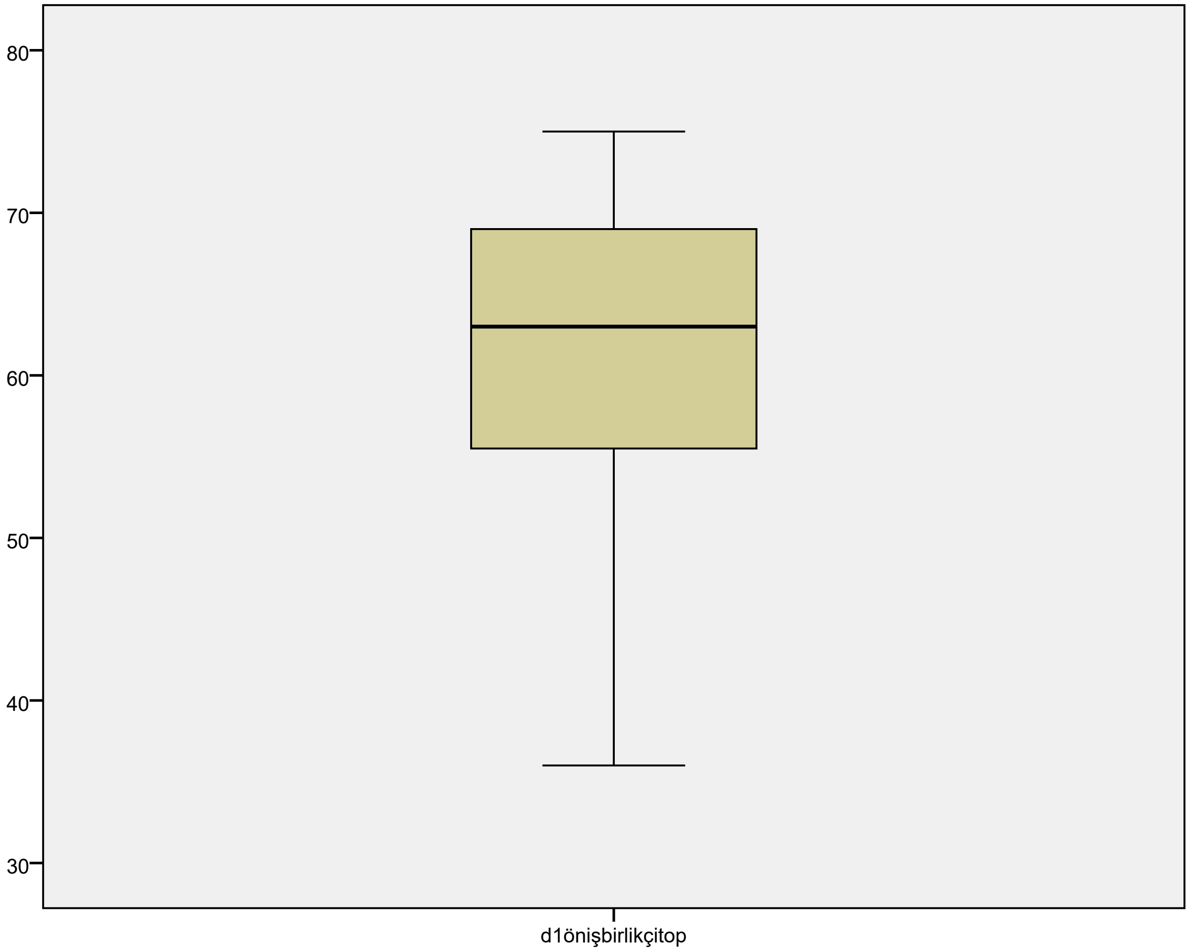

d1öndijitaltektop

```
d1öndijitaltektop Stem-and-Leaf Plot

 Frequency    Stem &  Leaf

     2,00       11 .  67
     5,00       12 .  23344
     8,00       12 .  56667889
     3,00       13 .  034
     2,00       13 .  78
     3,00       14 .  012
     1,00       14 .  9

 Stem width:     10,00
 Each leaf:       1 case(s)
```

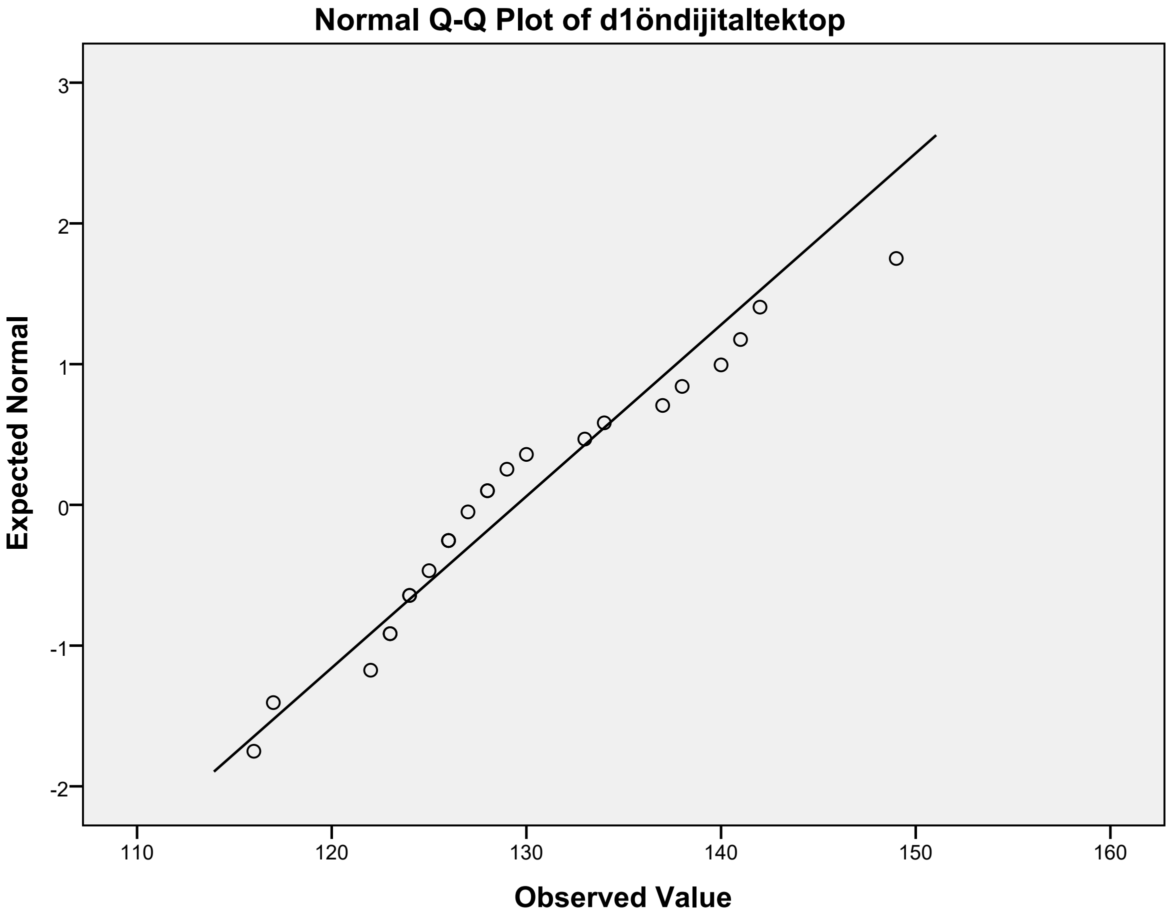
Normal Q-Q Plot of d1öndijitaltektop
Expected Normal
Observed Value
3
2
1
0
-1
-2
110
120
130
140
150
160

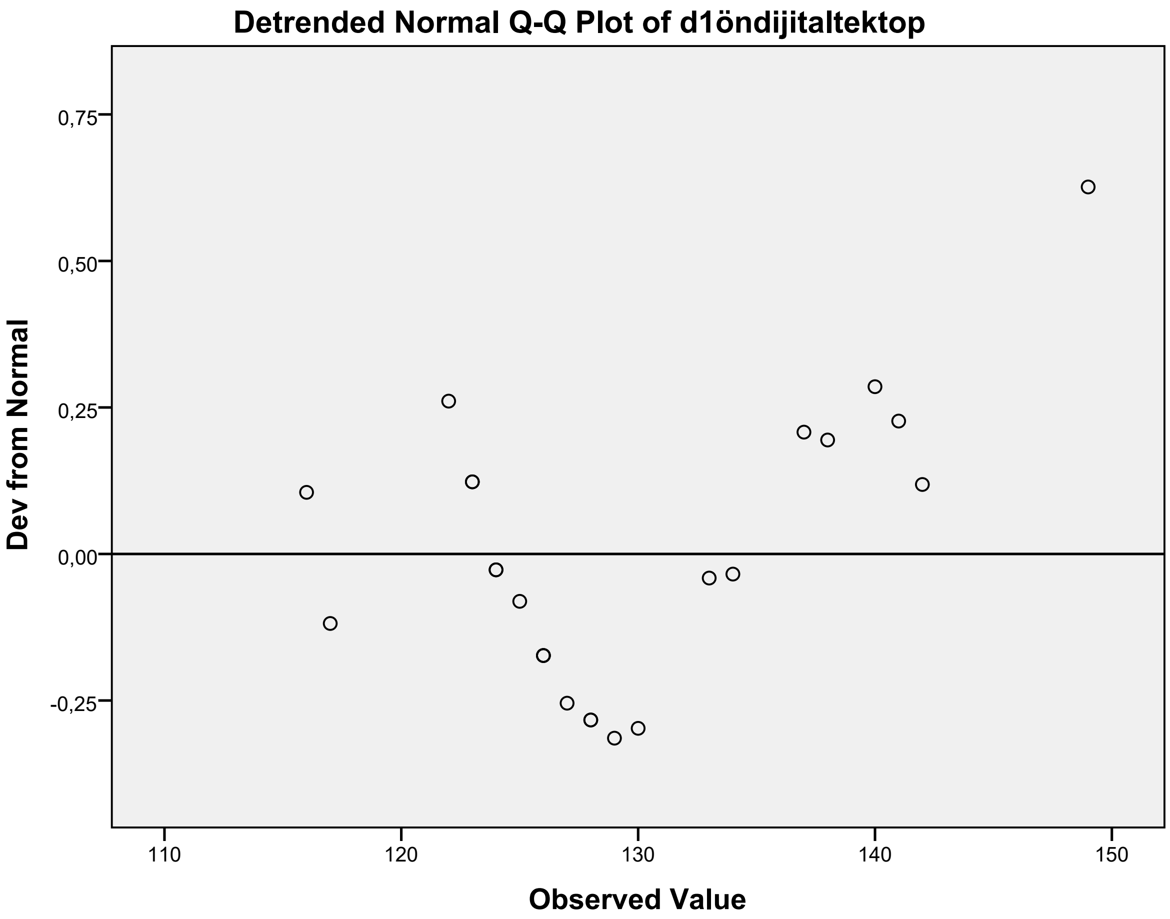
Detrended Normal Q-Q Plot of d1öndijitaltektop
Dev from Normal
0,75
0,50
0,25
0,00
-0,25
110
120
130
140
150
Observed Value

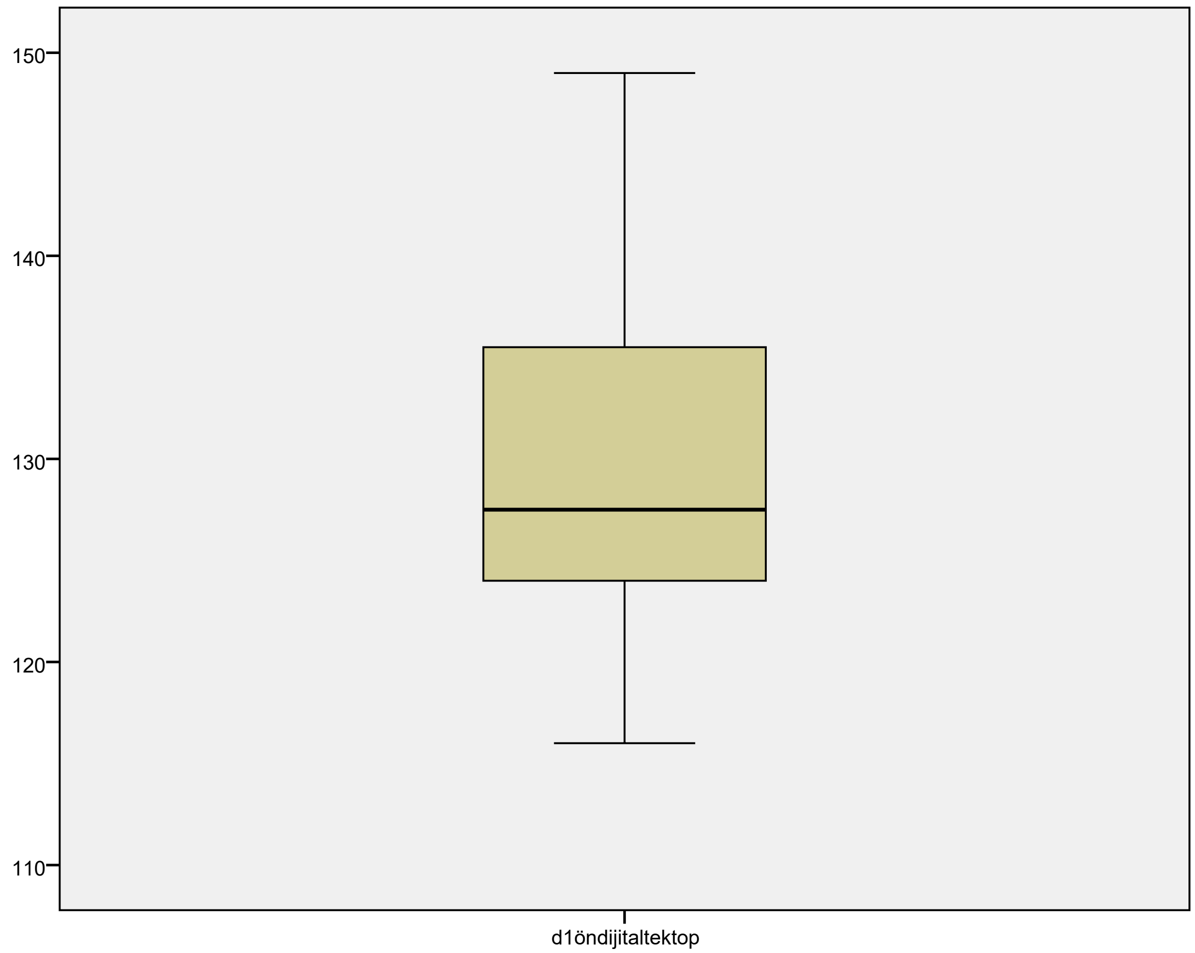

d1önöğrenmestratejileritop

```
d1önöğrenmestratejileritop Stem-and-Leaf Plot

 Frequency    Stem &  Leaf

     7,00        2 .  0334444
    15,00        2 .  555556666778999
     2,00        3 .  00

 Stem width:    100,00
 Each leaf:       1 case(s)
```

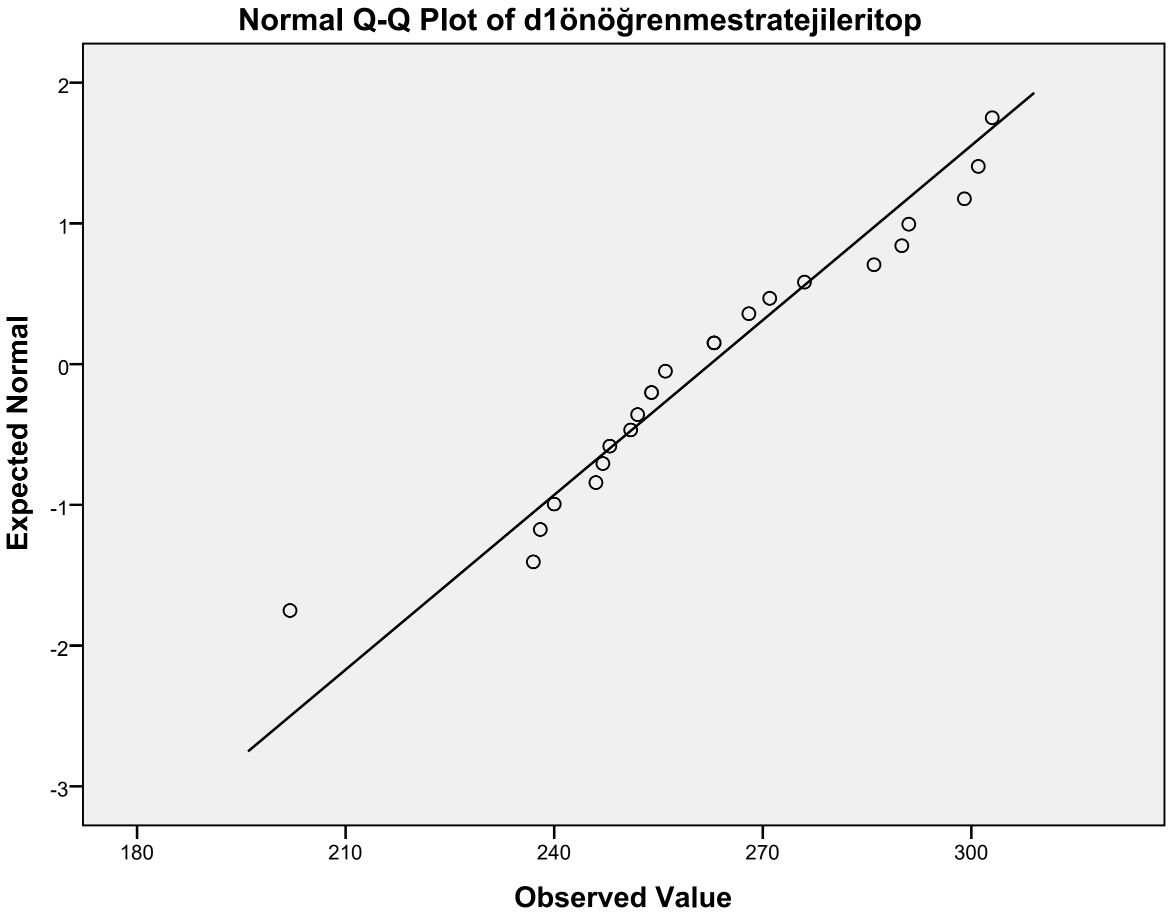
Normal Q-Q Plot of d1önöğrenmestratejileritop
Expected Normal
2
1
0
-1
-2
-3
180
210
240
270
300
Observed Value

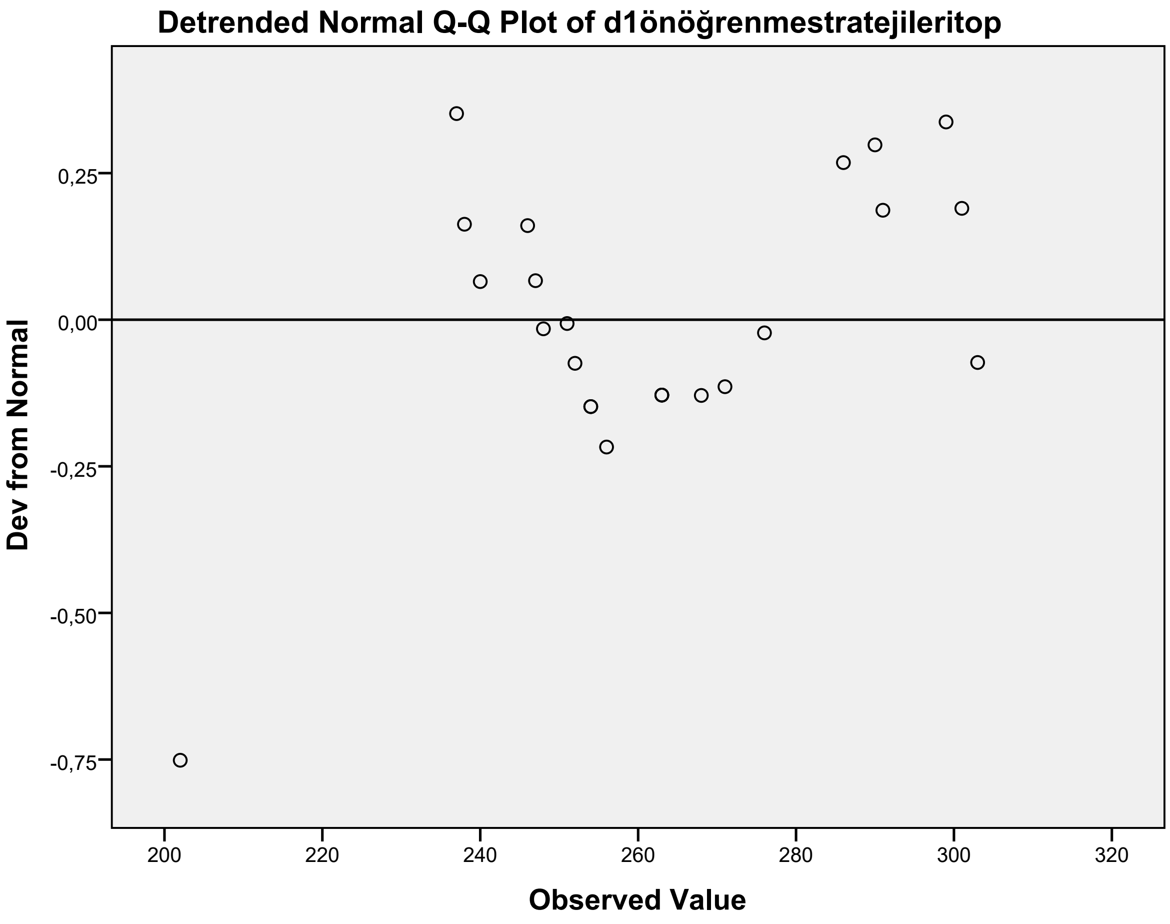
Detrended Normal Q-Q Plot of d1önöğrenmestratejileritop
0,25
0,00
-0,25
-0,50
-0,75
Dev from Normal
200
220
240
260
280
300
320
Observed Value

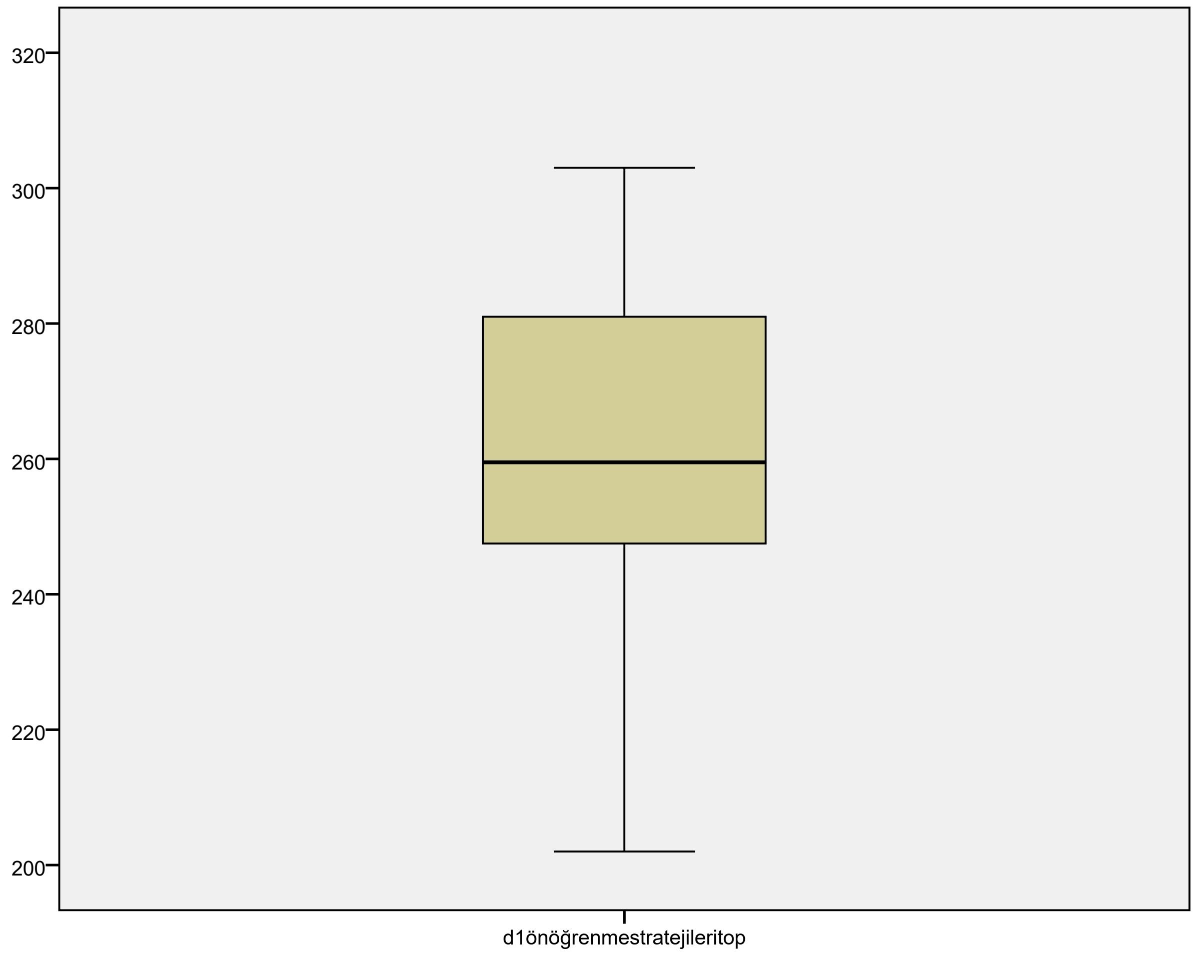

d1sonişbirliktop

```
d1sonişbirliktop Stem-and-Leaf Plot

 Frequency    Stem &  Leaf

     2,00 Extremes    (=<36)
     1,00        4 .  1
     3,00        5 .  048
     9,00        6 .  004567789
     7,00        7 .  0134566
     2,00        8 .  16

 Stem width:     10,00
 Each leaf:       1 case(s)
```

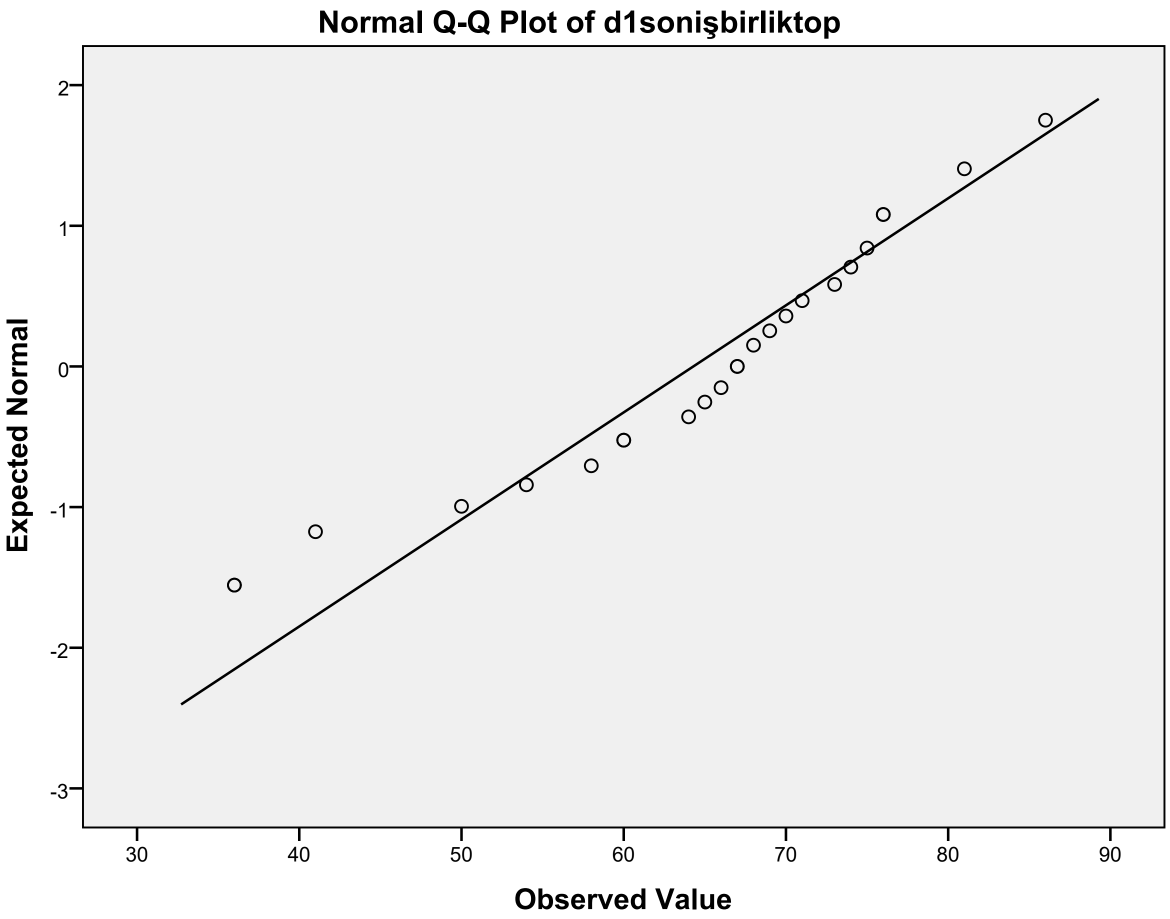
Normal Q-Q Plot of d1sonişbirliktop
Expected Normal
Observed Value
2
1
0
-1
-2
-3
30
40
50
60
70
80
90

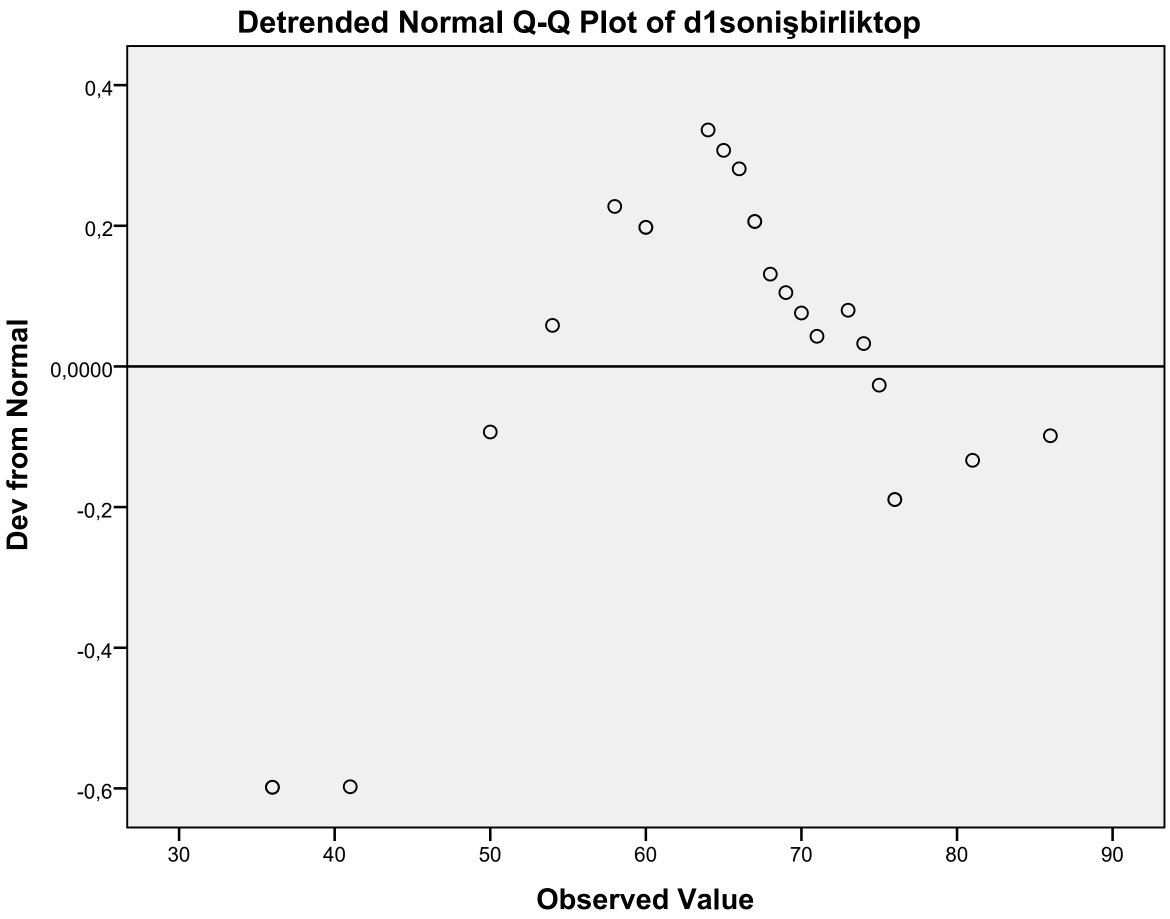
Detrended Normal Q-Q Plot of d1sonişbirliktop
Dev from Normal
0,4
0,2
0,0000
-0,2
-0,4
-0,6
30
40
50
60
70
80
90
Observed Value

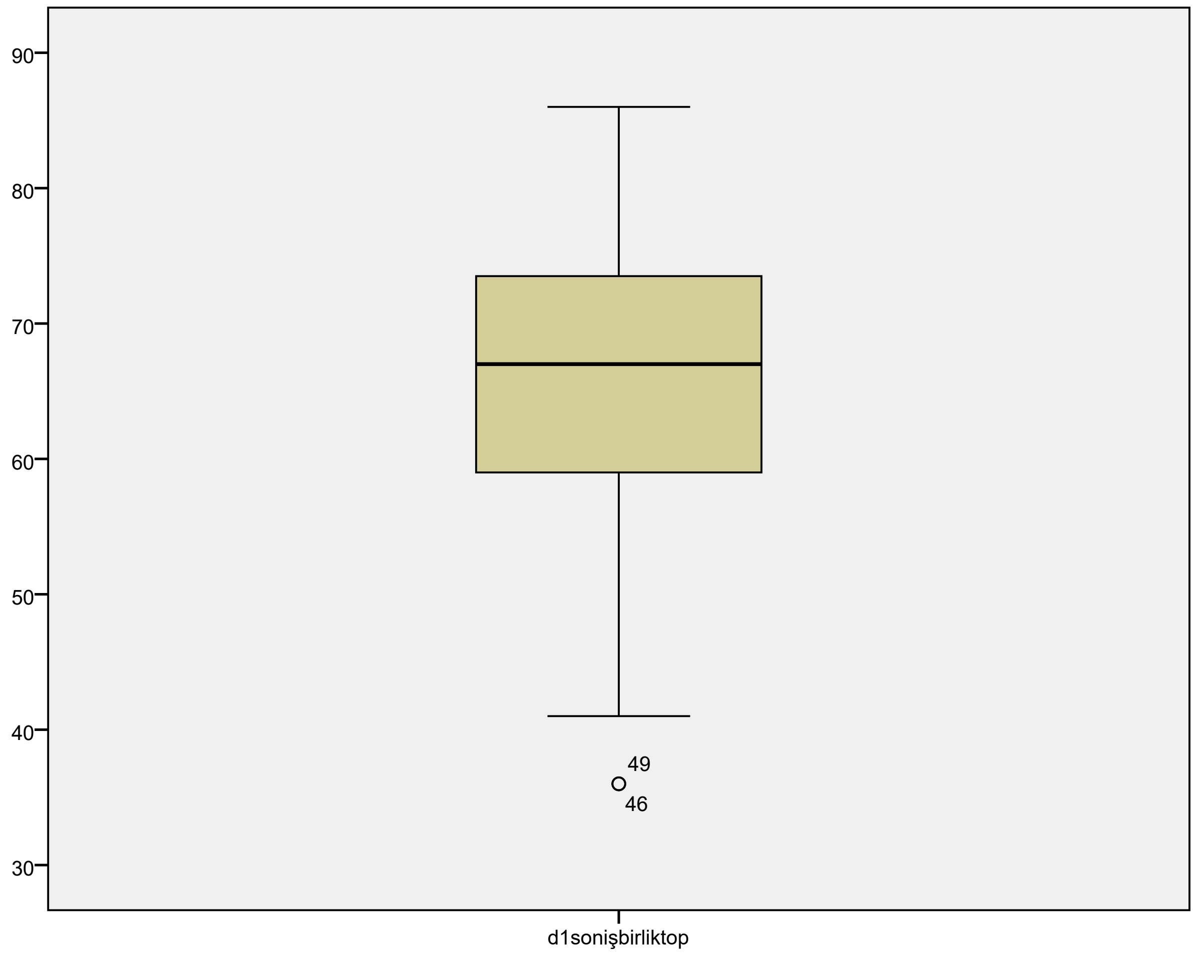

d1sondijitaltektop

```
d1sondijitaltektop Stem-and-Leaf Plot

 Frequency    Stem &  Leaf

     3,00       12 .  037
     4,00       13 .  0457
    11,00       14 .  12334445669
     3,00       15 .  017
     1,00       16 .  0
     2,00 Extremes    (>=175)

 Stem width:     10,00
 Each leaf:       1 case(s)
```

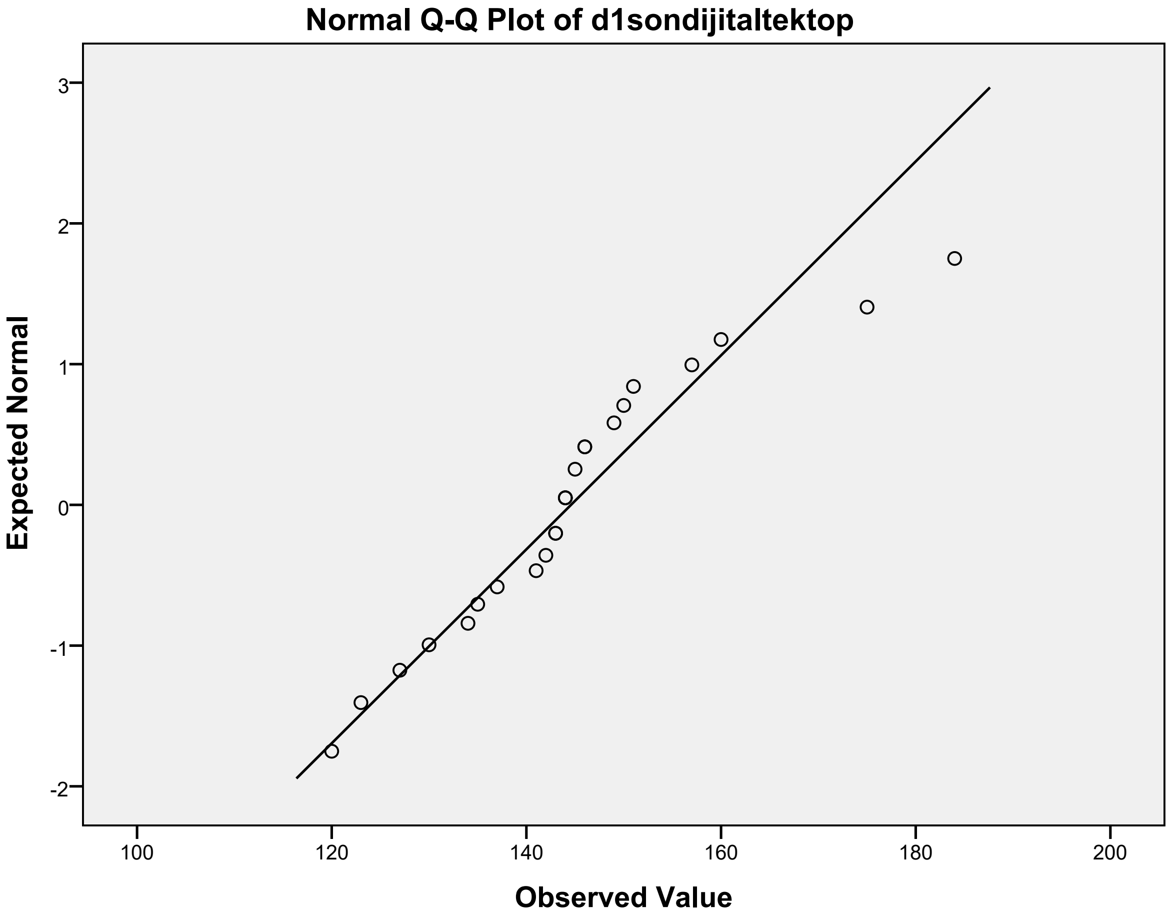
Normal Q-Q Plot of d1sondijitaltektop
Expected Normal
3
2
1
0
-1
-2
100
120
140
160
180
200
Observed Value

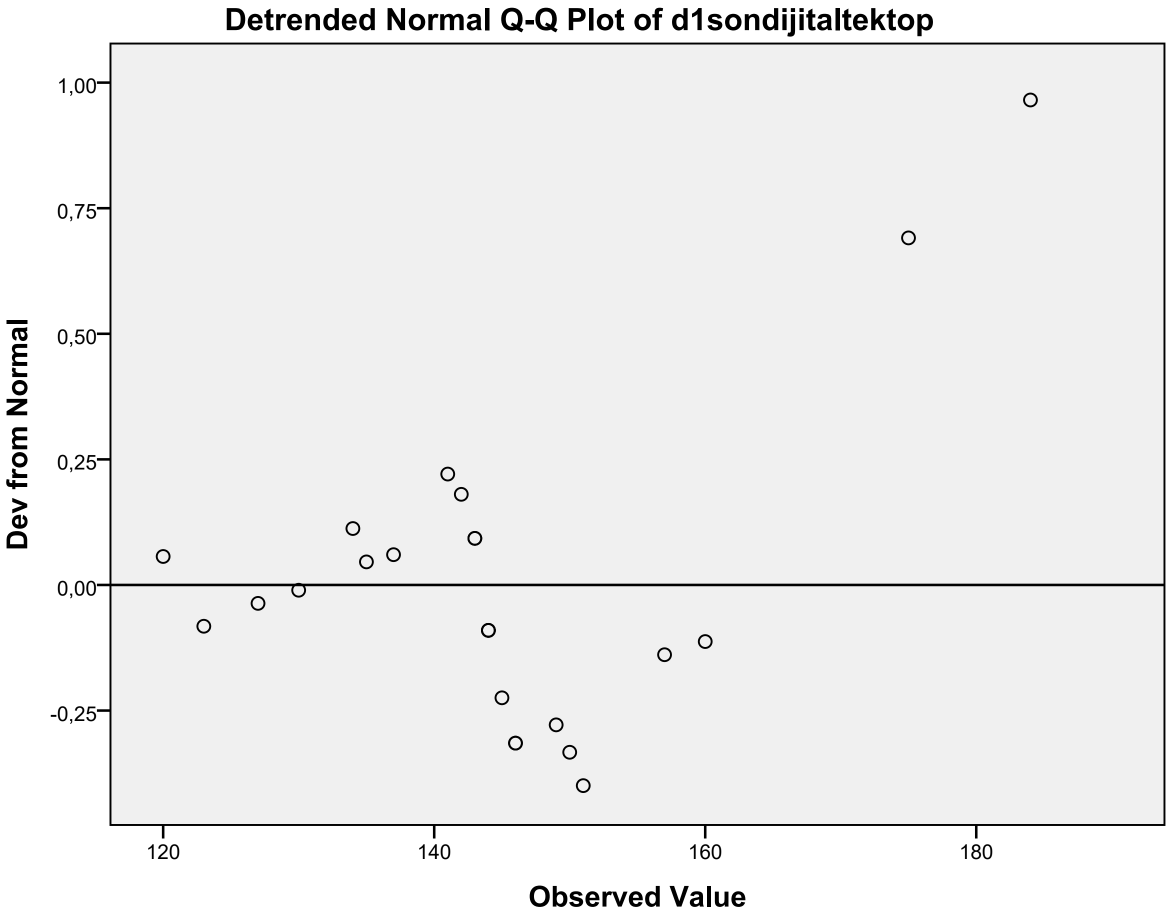

Detrended Normal Q-Q Plot of d1sondijitaltektop
Dev from Normal
1,00
0,75
0,50
0,25
0,00
-0,25
120
140
160
180
Observed Value

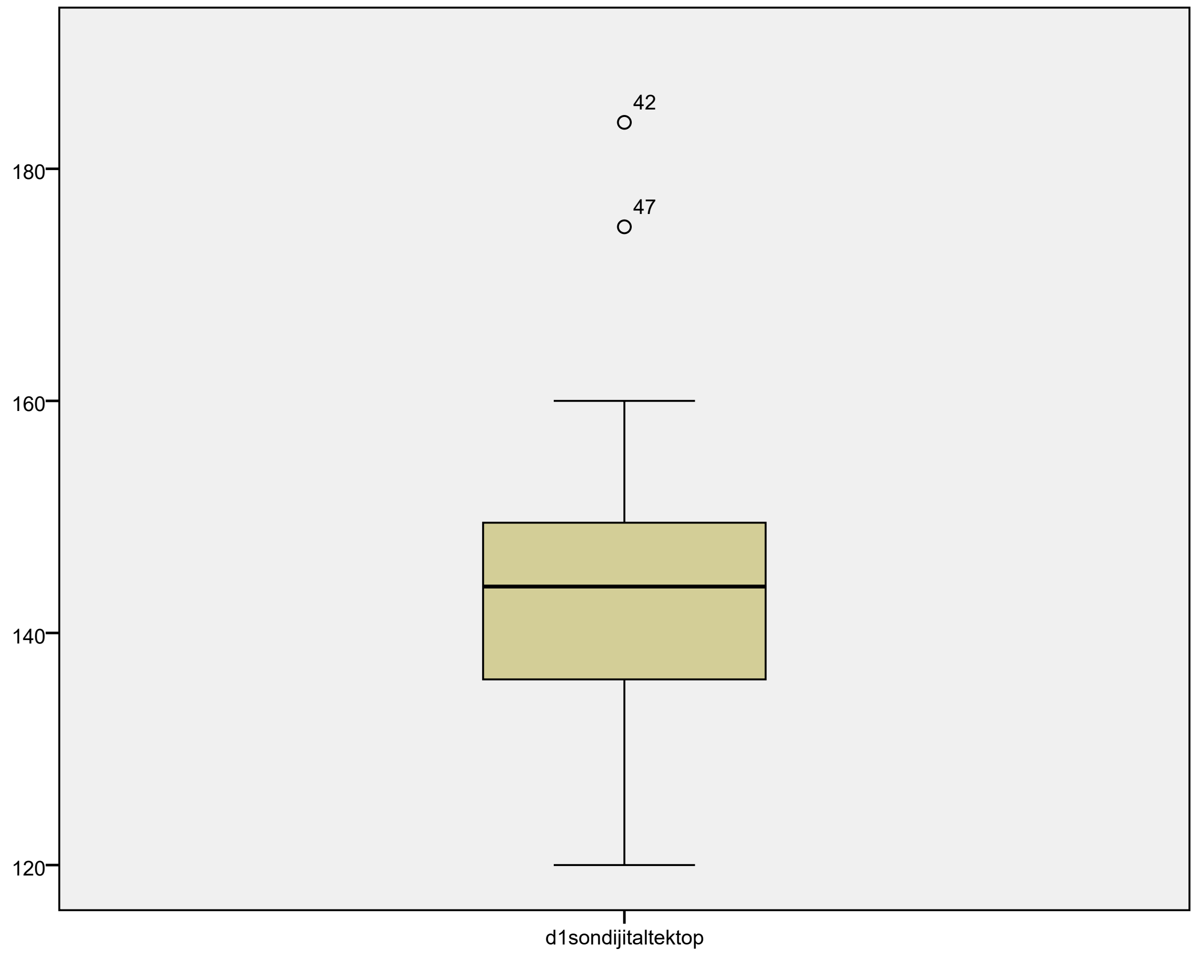

d1sonöğrstratejitop

```
d1sonöğrstratejitop Stem-and-Leaf Plot

 Frequency    Stem &  Leaf

     3,00        2 .  044
    14,00        2 .  55555677888999
     7,00        3 .  0001223

 Stem width:    100,00
 Each leaf:       1 case(s)
```

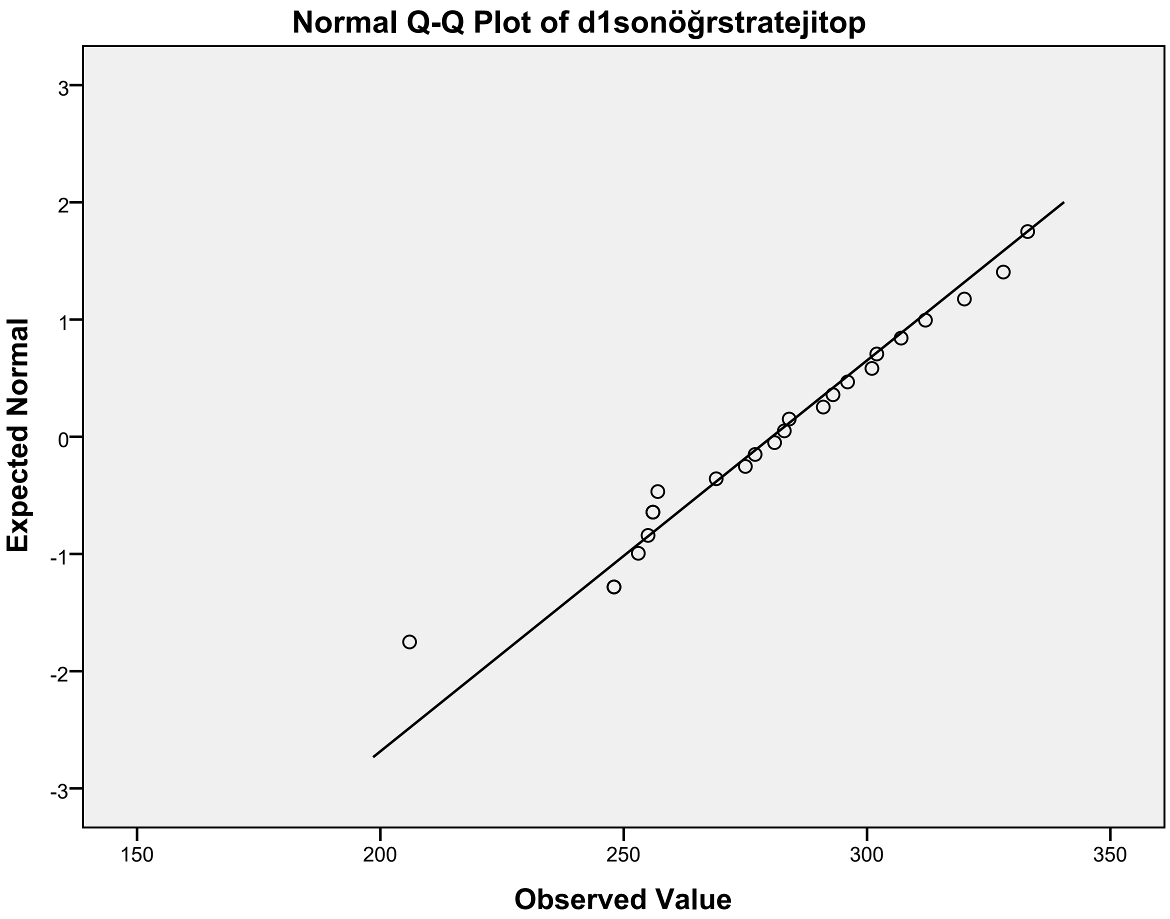
Normal Q-Q Plot of d1sonöğrstratejitop
Expected Normal
Observed Value
3
2
1
0
-1
-2
-3
150
200
250
300
350

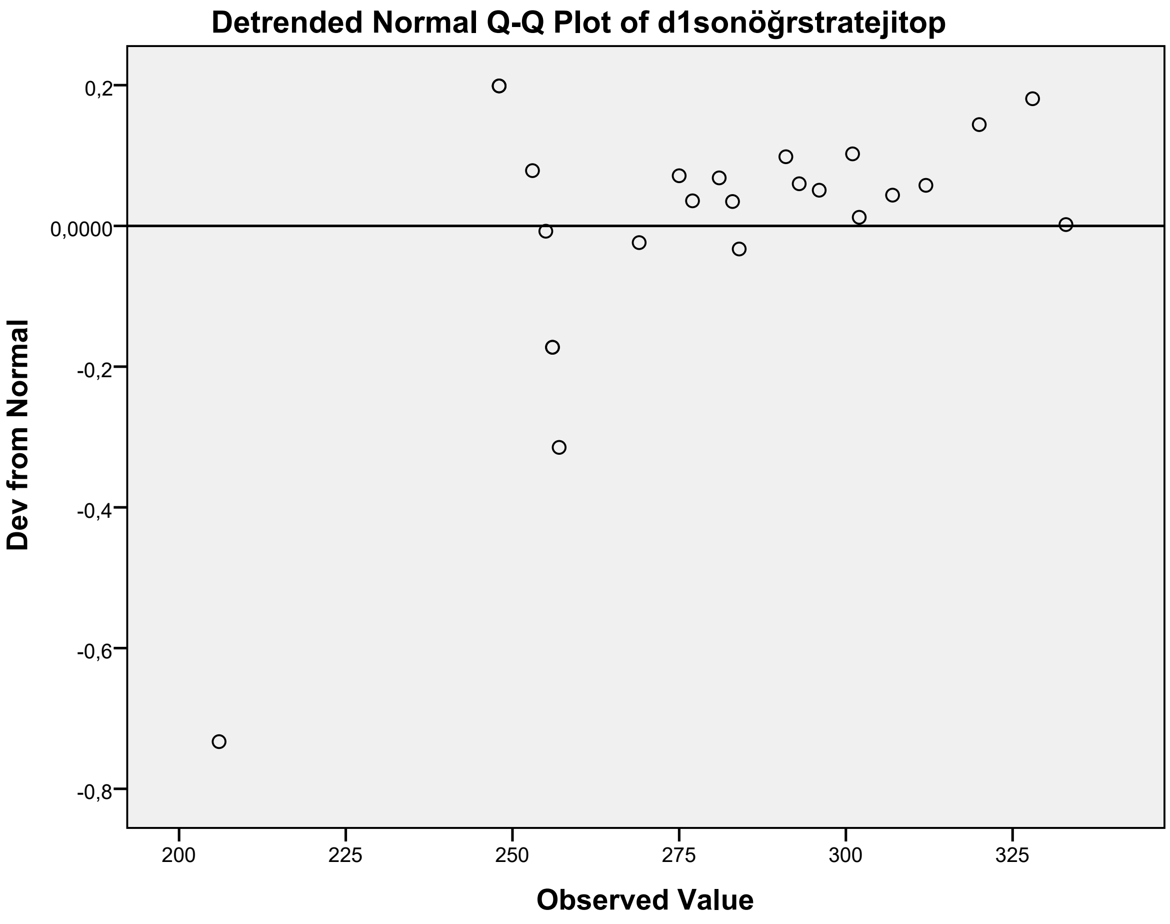
Detrended Normal Q-Q Plot of d1sonöğrstratejitop
Dev from Normal
0,2
0,0000
-0,2
-0,4
-0,6
-0,8
200
225
250
275
300
325
Observed Value

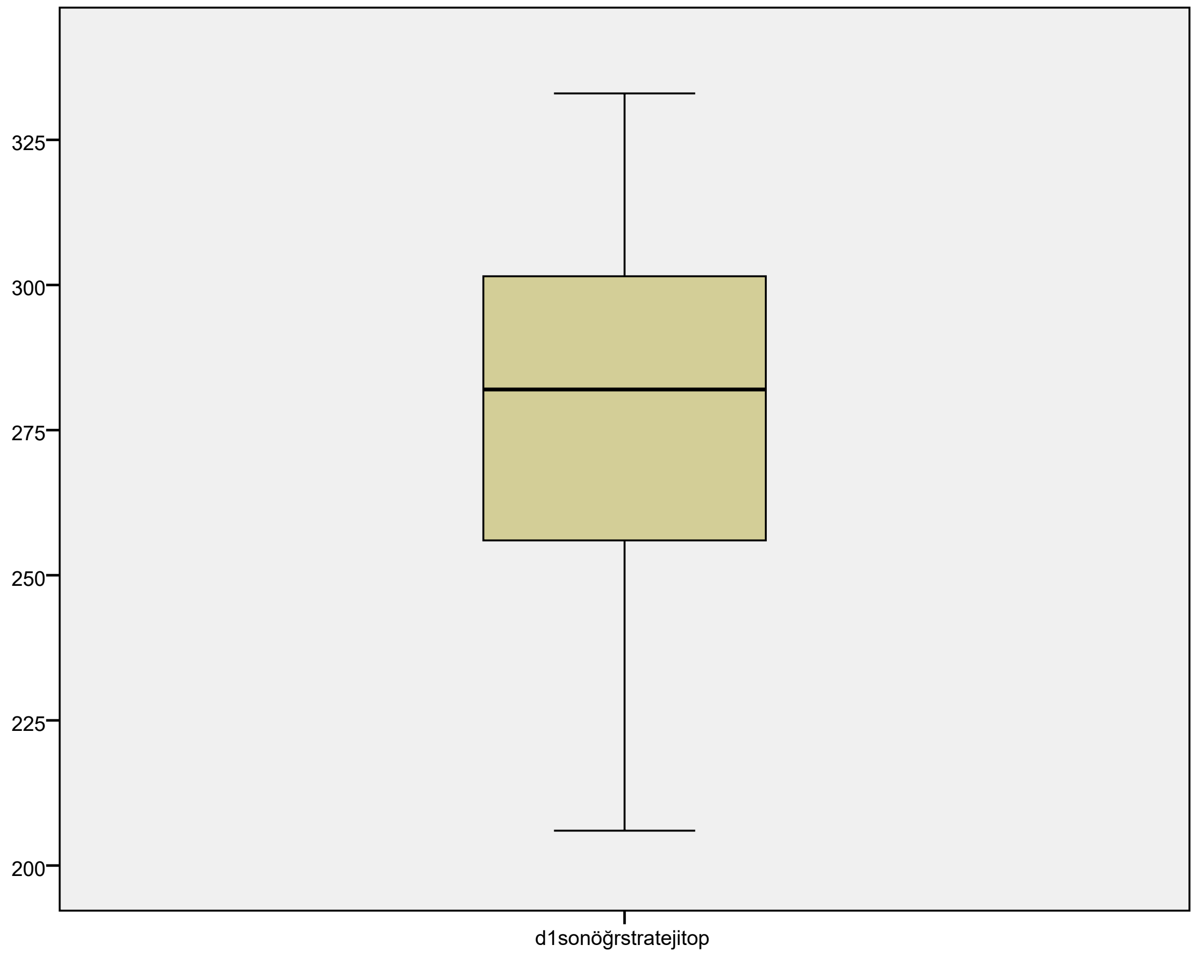


```
FREQUENCIES VARIABLES=d1önişbirlikçitop d1öndijitaltektop d1önöğrenmestratejileritop d1soniş
    d1sonöğrstratejitop
  /STATISTICS=MEAN MEDIAN MODE SKEWNESS SESKEW KURTOSIS SEKURT
  /ORDER=ANALYSIS.
```

Frequencies

Notes

Output Created		03-NOV-2024 21:04:49
Comments		
Input	Data	C:\Users\Gizem\Pictures\NİHAL HOCAYLA ÇALIŞMA 2023\tersyüz-web ana analiz\tersyüz-web ana analiz.sav
	Active Dataset	DataSet1
	Filter	grup = 2 (FILTER)
	Weight	<none>
	Split File	<none>
	N of Rows in Working Data File	24
Missing Value Handling	Definition of Missing	User-defined missing values are treated as missing.
	Cases Used	Statistics are based on all cases with valid data.
Syntax		FREQUENCIES VARIABLES=d1önişbirlikçitop d1öndijitaltektop d1önöğrenmestratejileritop d1sonişbirliktop d1sondijitaltektop d1sonöğrstratejitop /STATISTICS=MEAN MEDIAN MODE SKEWNESS SESKEW KURTOSIS SEKURT /ORDER=ANALYSIS.
Resources	Processor Time	00:00:00,02
	Elapsed Time	00:00:00,01

```
[DataSet1] C:\Users\Gizem\Pictures\NİHAL HOCAYLA ÇALIŞMA 2023\tersyüz-web
ana analiz\tersyüz-web ana analiz.sav
```

Statistics

		d1önişbirlikçitop	d1öndijitaltektop	d1önöğrenmestratejileritop	d1sonişbirliktop
N	Valid	24	24	24	24
	Missing	0	0	0	0
Mean		61,7083	129,5000	262,4583	64,2917
Median		63,0000	127,5000	259,5000	67,0000
Mode		63,00	126,00	263,00	36,00[a]
Skewness		-,914	,623	-,153	-,852
Std. Error of Skewness		,472	,472	,472	,472
Kurtosis		,903	-,011	,355	,354
Std. Error of Kurtosis		,918	,918	,918	,918

Statistics

		d1sondijitaltektop	d1sonöğrstratejitop
N	Valid	24	24
	Missing	0	0
Mean		144,5833	280,4583
Median		144,0000	282,0000
Mode		144,00	248,00[a]
Skewness		,923	-,303
Std. Error of Skewness		,472	,472
Kurtosis		1,785	,209
Std. Error of Kurtosis		,918	,918

a. Multiple modes exist. The smallest value is shown

Frequency Table

d1önişbirlikçitop

		Frequency	Percent	Valid Percent	Cumulative Percent
Valid	36,00	1	4,2	4,2	4,2
	47,00	1	4,2	4,2	8,3
	50,00	1	4,2	4,2	12,5
	51,00	1	4,2	4,2	16,7
	54,00	1	4,2	4,2	20,8
	55,00	1	4,2	4,2	25,0
	56,00	1	4,2	4,2	29,2
	59,00	1	4,2	4,2	33,3
	61,00	2	8,3	8,3	41,7
	63,00	4	16,7	16,7	58,3
	65,00	1	4,2	4,2	62,5
	66,00	1	4,2	4,2	66,7
	68,00	1	4,2	4,2	70,8
	69,00	2	8,3	8,3	79,2
	70,00	1	4,2	4,2	83,3
	71,00	1	4,2	4,2	87,5
	72,00	1	4,2	4,2	91,7
	74,00	1	4,2	4,2	95,8
	75,00	1	4,2	4,2	100,0
	Total	24	100,0	100,0	

d1öndijitaltektop

		Frequency	Percent	Valid Percent	Cumulative Percent
Valid	116,00	1	4,2	4,2	4,2
	117,00	1	4,2	4,2	8,3
	122,00	1	4,2	4,2	12,5
	123,00	2	8,3	8,3	20,8
	124,00	2	8,3	8,3	29,2
	125,00	1	4,2	4,2	33,3
	126,00	3	12,5	12,5	45,8
	127,00	1	4,2	4,2	50,0
	128,00	2	8,3	8,3	58,3
	129,00	1	4,2	4,2	62,5
	130,00	1	4,2	4,2	66,7
	133,00	1	4,2	4,2	70,8
	134,00	1	4,2	4,2	75,0
	137,00	1	4,2	4,2	79,2
	138,00	1	4,2	4,2	83,3
	140,00	1	4,2	4,2	87,5
	141,00	1	4,2	4,2	91,7
	142,00	1	4,2	4,2	95,8
	149,00	1	4,2	4,2	100,0
	Total	24	100,0	100,0	

d1önöğrenmestratejileritop

		Frequency	Percent	Valid Percent	Cumulative Percent
Valid	202,00	1	4,2	4,2	4,2
	237,00	1	4,2	4,2	8,3
	238,00	1	4,2	4,2	12,5
	240,00	1	4,2	4,2	16,7
	246,00	1	4,2	4,2	20,8
	247,00	1	4,2	4,2	25,0
	248,00	1	4,2	4,2	29,2
	251,00	1	4,2	4,2	33,3
	252,00	1	4,2	4,2	37,5
	254,00	2	8,3	8,3	45,8
	256,00	1	4,2	4,2	50,0
	263,00	3	12,5	12,5	62,5
	268,00	1	4,2	4,2	66,7
	271,00	1	4,2	4,2	70,8
	276,00	1	4,2	4,2	75,0
	286,00	1	4,2	4,2	79,2
	290,00	1	4,2	4,2	83,3
	291,00	1	4,2	4,2	87,5
	299,00	1	4,2	4,2	91,7
	301,00	1	4,2	4,2	95,8
	303,00	1	4,2	4,2	100,0
	Total	24	100,0	100,0	

d1sonişbirliktop

		Frequency	Percent	Valid Percent	Cumulative Percent
Valid	36,00	2	8,3	8,3	8,3
	41,00	1	4,2	4,2	12,5
	50,00	1	4,2	4,2	16,7
	54,00	1	4,2	4,2	20,8
	58,00	1	4,2	4,2	25,0
	60,00	2	8,3	8,3	33,3
	64,00	1	4,2	4,2	37,5
	65,00	1	4,2	4,2	41,7
	66,00	1	4,2	4,2	45,8
	67,00	2	8,3	8,3	54,2
	68,00	1	4,2	4,2	58,3
	69,00	1	4,2	4,2	62,5
	70,00	1	4,2	4,2	66,7
	71,00	1	4,2	4,2	70,8
	73,00	1	4,2	4,2	75,0
	74,00	1	4,2	4,2	79,2
	75,00	1	4,2	4,2	83,3
	76,00	2	8,3	8,3	91,7
	81,00	1	4,2	4,2	95,8
	86,00	1	4,2	4,2	100,0
	Total	24	100,0	100,0	

d1sondijitaltektop

		Frequency	Percent	Valid Percent	Cumulative Percent
Valid	120,00	1	4,2	4,2	4,2
	123,00	1	4,2	4,2	8,3
	127,00	1	4,2	4,2	12,5
	130,00	1	4,2	4,2	16,7
	134,00	1	4,2	4,2	20,8
	135,00	1	4,2	4,2	25,0
	137,00	1	4,2	4,2	29,2
	141,00	1	4,2	4,2	33,3
	142,00	1	4,2	4,2	37,5
	143,00	2	8,3	8,3	45,8
	144,00	3	12,5	12,5	58,3
	145,00	1	4,2	4,2	62,5
	146,00	2	8,3	8,3	70,8
	149,00	1	4,2	4,2	75,0
	150,00	1	4,2	4,2	79,2
	151,00	1	4,2	4,2	83,3
	157,00	1	4,2	4,2	87,5
	160,00	1	4,2	4,2	91,7
	175,00	1	4,2	4,2	95,8
	184,00	1	4,2	4,2	100,0
	Total	24	100,0	100,0	

d1sonöğrstratejitop

		Frequency	Percent	Valid Percent	Cumulative Percent
Valid	206,00	1	4,2	4,2	4,2
	248,00	2	8,3	8,3	12,5
	253,00	1	4,2	4,2	16,7
	255,00	1	4,2	4,2	20,8
	256,00	2	8,3	8,3	29,2
	257,00	1	4,2	4,2	33,3
	269,00	1	4,2	4,2	37,5
	275,00	1	4,2	4,2	41,7
	277,00	1	4,2	4,2	45,8
	281,00	1	4,2	4,2	50,0
	283,00	1	4,2	4,2	54,2
	284,00	1	4,2	4,2	58,3
	291,00	1	4,2	4,2	62,5
	293,00	1	4,2	4,2	66,7
	296,00	1	4,2	4,2	70,8
	301,00	1	4,2	4,2	75,0
	302,00	1	4,2	4,2	79,2
	307,00	1	4,2	4,2	83,3
	312,00	1	4,2	4,2	87,5
	320,00	1	4,2	4,2	91,7
	328,00	1	4,2	4,2	95,8
	333,00	1	4,2	4,2	100,0
	Total	24	100,0	100,0	

```
 USE ALL.
COMPUTE filter_$=(grup = 3).
VARIABLE LABELS filter_$ 'grup = 3 (FILTER)'.
VALUE LABELS filter_$ 0 'Not Selected' 1 'Selected'.
FORMATS filter_$ (f1.0).
FILTER BY filter_$.
EXECUTE.
EXAMINE VARIABLES=d1önişbirlikçitop d1öndijitaltektop d1önöğrenmestratejileritop d1sonişbirl
    d1sonöğrstratejitop
  /PLOT BOXPLOT STEMLEAF NPPLOT
  /COMPARE GROUPS
  /STATISTICS DESCRIPTIVES
  /CINTERVAL 95
  /MISSING LISTWISE
  /NOTOTAL.
```

Explore

Notes

Output Created		03-NOV-2024 21:05:17
Comments		
Input	Data	C: \Users\Gizem\Pictures\NİHAL HOCAYLA ÇALIŞMA 2023\tersyüz-web ana analiz\tersyüz-web ana analiz.sav
	Active Dataset	DataSet1
	Filter	grup = 3 (FILTER)
	Weight	<none>
	Split File	<none>
	N of Rows in Working Data File	24
Missing Value Handling	Definition of Missing	User-defined missing values for dependent variables are treated as missing.
	Cases Used	Statistics are based on cases with no missing values for any dependent variable or factor used.
Syntax		EXAMINE VARIABLES=d1önişbirlikçitop d1öndijitaltektop d1önöğrenmestratejileritop d1sonişbirliktop d1sondijitaltektop d1sonöğrstratejitop /PLOT BOXPLOT STEMLEAF NPPLOT /COMPARE GROUPS /STATISTICS DESCRIPTIVES /CINTERVAL 95 /MISSING LISTWISE /NOTOTAL.
Resources	Processor Time	00:00:02,05
	Elapsed Time	00:00:01,99

```
[DataSet1] C:\Users\Gizem\Pictures\NİHAL HOCAYLA ÇALIŞMA 2023\tersyüz-web
ana analiz\tersyüz-web ana analiz.sav
```

Case Processing Summary

	Cases					
	Valid		Missing		Total	
	N	Percent	N	Percent	N	Percent
d1önişbirlikçitop	24	100,0%	0	0,0%	24	100,0%
d1öndijitaltektop	24	100,0%	0	0,0%	24	100,0%
d1önöğrenmestratejileritop	24	100,0%	0	0,0%	24	100,0%
d1sonişbirliktop	24	100,0%	0	0,0%	24	100,0%
d1sondijitaltektop	24	100,0%	0	0,0%	24	100,0%
d1sonöğrstratejitop	24	100,0%	0	0,0%	24	100,0%

Descriptives

			Statistic	Std. Error
d1önişbirlikçitop	Mean		59,2917	1,54108
	95% Confidence Interval for Mean	Lower Bound	56,1037	
		Upper Bound	62,4796	
	5% Trimmed Mean		59,0833	
	Median		57,0000	
	Variance		56,998	
	Std. Deviation		7,54971	
	Minimum		46,00	
	Maximum		76,00	
	Range		30,00	
	Interquartile Range		10,50	
	Skewness		,690	,472
	Kurtosis		-,142	,918
d1öndijitaltektop	Mean		123,4583	1,81278
	95% Confidence Interval for Mean	Lower Bound	119,7083	
		Upper Bound	127,2083	
	5% Trimmed Mean		122,8704	
	Median		121,0000	
	Variance		78,868	
	Std. Deviation		8,88075	
	Minimum		111,00	
	Maximum		148,00	
	Range		37,00	
	Interquartile Range		11,50	
	Skewness		,994	,472
	Kurtosis		,984	,918
d1önöğrenmestratejileritop	Mean		279,5833	9,01125
	95% Confidence Interval for Mean	Lower Bound	260,9421	
		Upper Bound	298,2245	
	5% Trimmed Mean		284,2685	
	Median		284,5000	

Descriptives

			Statistic	Std. Error
	Variance		1948,862	
	Std. Deviation		44,14592	
	Minimum		114,00	
	Maximum		342,00	
	Range		228,00	
	Interquartile Range		36,50	
	Skewness		-2,275	,472
	Kurtosis		8,363	,918
d1sonişbirliktop	Mean		58,1250	1,46988
	95% Confidence Interval for Mean	Lower Bound	55,0843	
		Upper Bound	61,1657	
	5% Trimmed Mean		58,1667	
	Median		57,0000	
	Variance		51,853	
	Std. Deviation		7,20092	
	Minimum		42,00	
	Maximum		72,00	
	Range		30,00	
	Interquartile Range		7,00	
	Skewness		,365	,472
	Kurtosis		,452	,918
d1sondijitaltektop	Mean		120,1250	1,68574
	95% Confidence Interval for Mean	Lower Bound	116,6378	
		Upper Bound	123,6122	
	5% Trimmed Mean		120,3056	
	Median		122,0000	
	Variance		68,201	
	Std. Deviation		8,25839	
	Minimum		103,00	
	Maximum		134,00	
	Range		31,00	
	Interquartile Range		10,75	
	Skewness		-,440	,472
	Kurtosis		-,404	,918
d1sonöğrstratejitop	Mean		256,3333	6,41970
	95% Confidence Interval for Mean	Lower Bound	243,0532	
		Upper Bound	269,6135	
	5% Trimmed Mean		258,9352	
	Median		256,0000	
	Variance		989,101	
	Std. Deviation		31,44998	
	Minimum		142,00	
	Maximum		313,00	

Descriptives

		Statistic	Std. Error
	Range	171,00	
	Interquartile Range	16,75	
	Skewness	-1,887	,472
	Kurtosis	7,440	,918

Tests of Normality

	Kolmogorov-Smirnov[a]			Shapiro-Wilk		
	Statistic	df	Sig.	Statistic	df	Sig.
d1önişbirlikçitop	,203	24	,012	,932	24	,110
d1öndijitaltektop	,152	24	,162	,932	24	,111
d1önöğrenmestratejileritop	,195	24	,018	,796	24	,000
d1sonişbirliktop	,145	24	,200*	,935	24	,129
d1sondijitaltektop	,139	24	,200*	,966	24	,581
d1sonöğrstratejitop	,208	24	,009	,811	24	,000

*. This is a lower bound of the true significance.

a. Lilliefors Significance Correction

d1önişbirlikçitop

```
d1önişbirlikçitop Stem-and-Leaf Plot

 Frequency    Stem &  Leaf

     1,00        4 .  6
     6,00        5 .  123344
     8,00        5 .  56777778
     3,00        6 .  013
     3,00        6 .  599
     2,00        7 .  03
     1,00        7 .  6

 Stem width:      10,00
 Each leaf:        1 case(s)
```

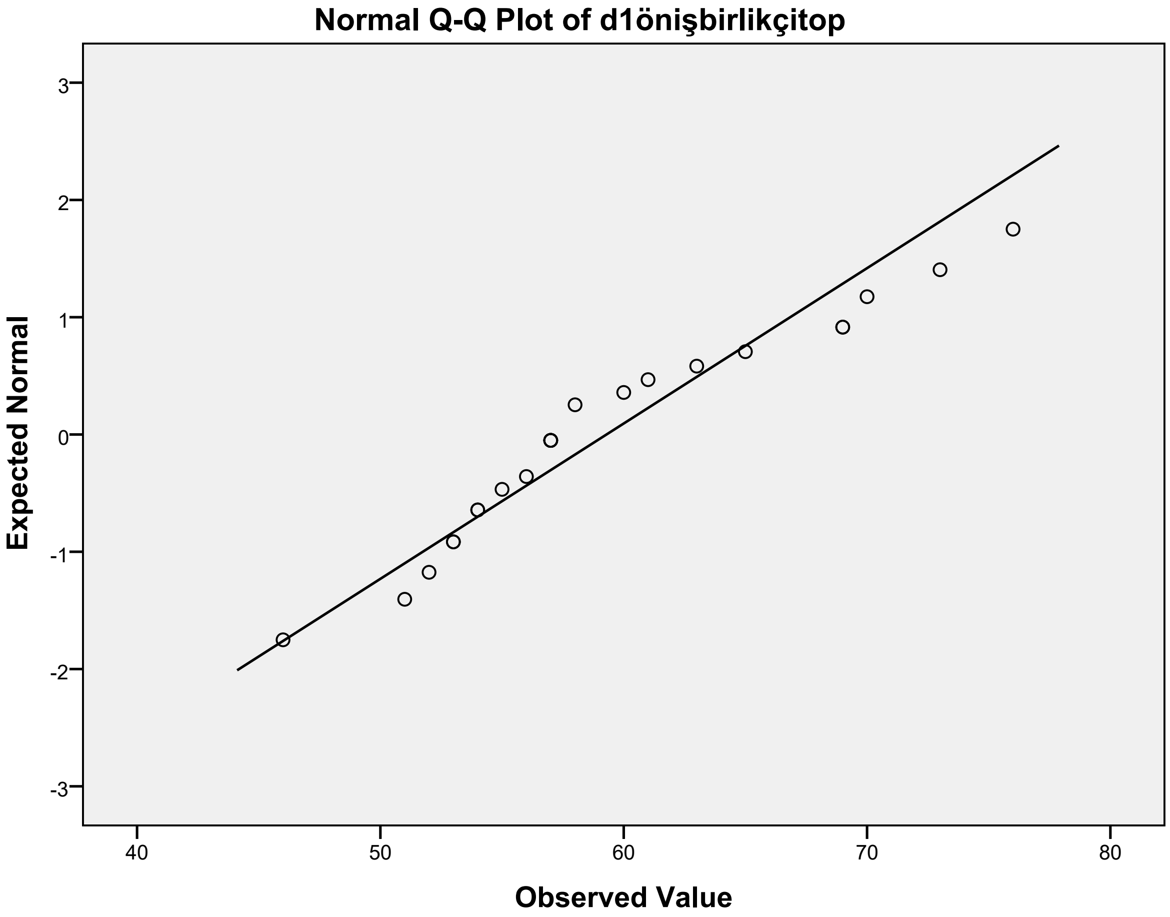
Normal Q-Q Plot of d1önişbirlikçitop
Expected Normal
3
2
1
0
-1
-2
-3
40
50
60
70
80
Observed Value

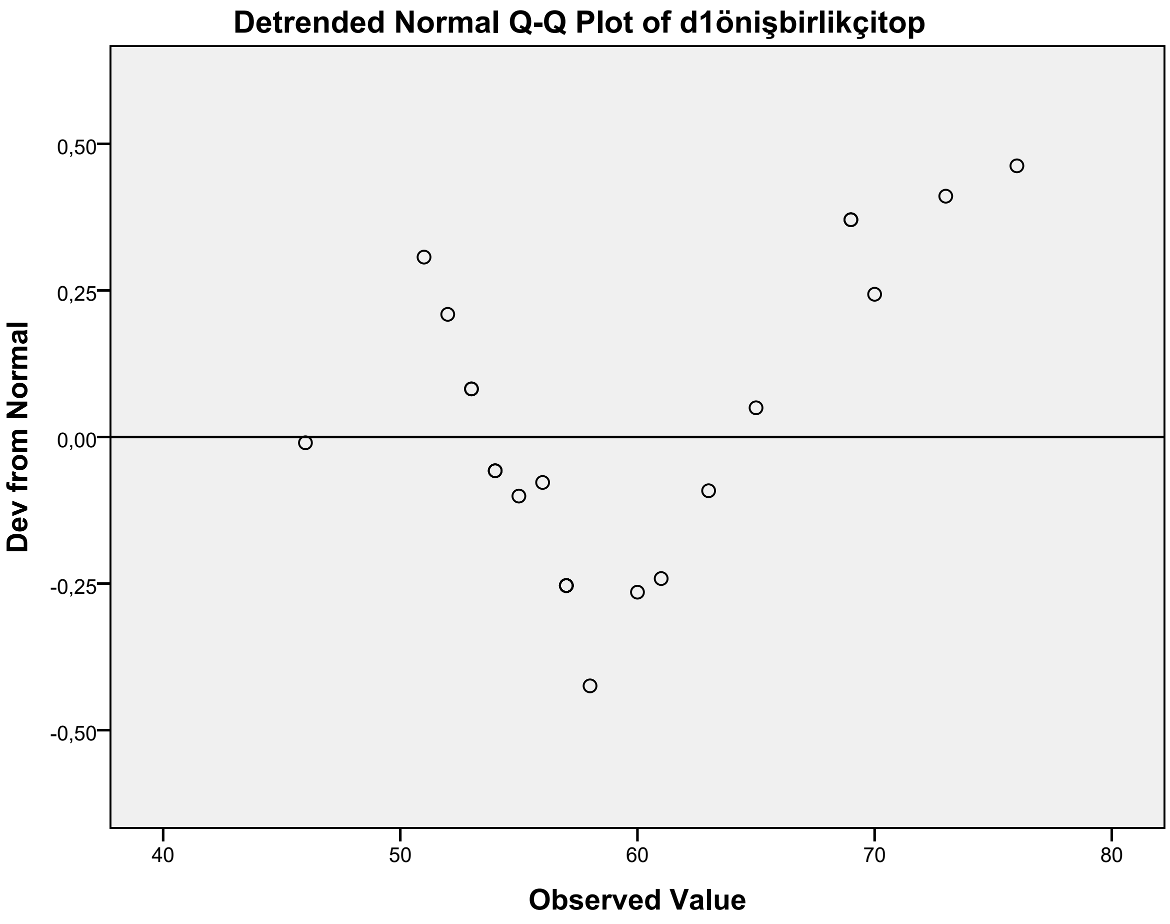
Detrended Normal Q-Q Plot of d1önişbirlikçitop
Dev from Normal
0,50
0,25
0,00
-0,25
-0,50
40
50
60
70
80
Observed Value

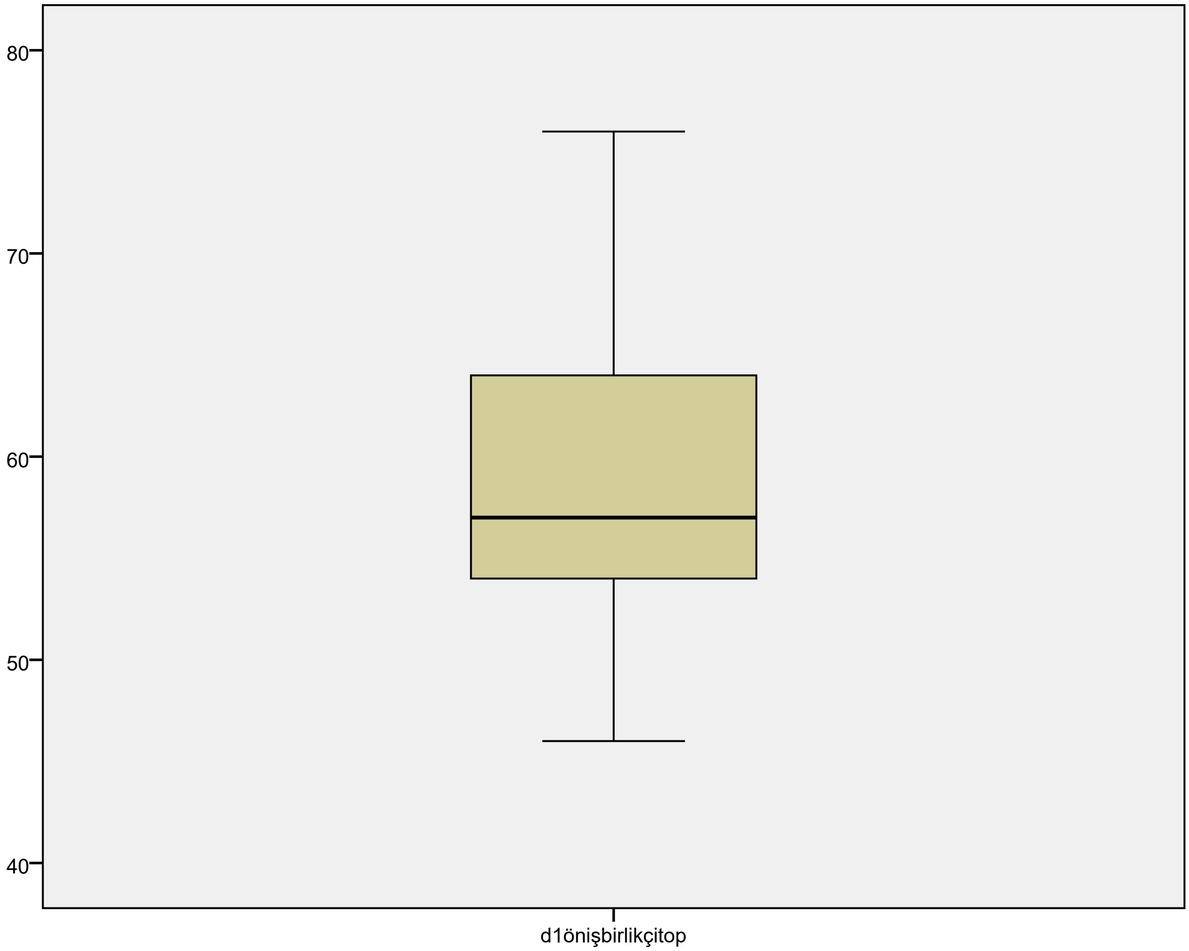

d1öndijitaltektop

```
d1öndijitaltektop Stem-and-Leaf Plot

 Frequency    Stem &  Leaf

     3,00       11 .  134
     7,00       11 .  5677899
     4,00       12 .  0022
     5,00       12 .  66779
     2,00       13 .  23
     2,00       13 .  57
     1,00 Extremes    (>=148)

 Stem width:     10,00
 Each leaf:       1 case(s)
```

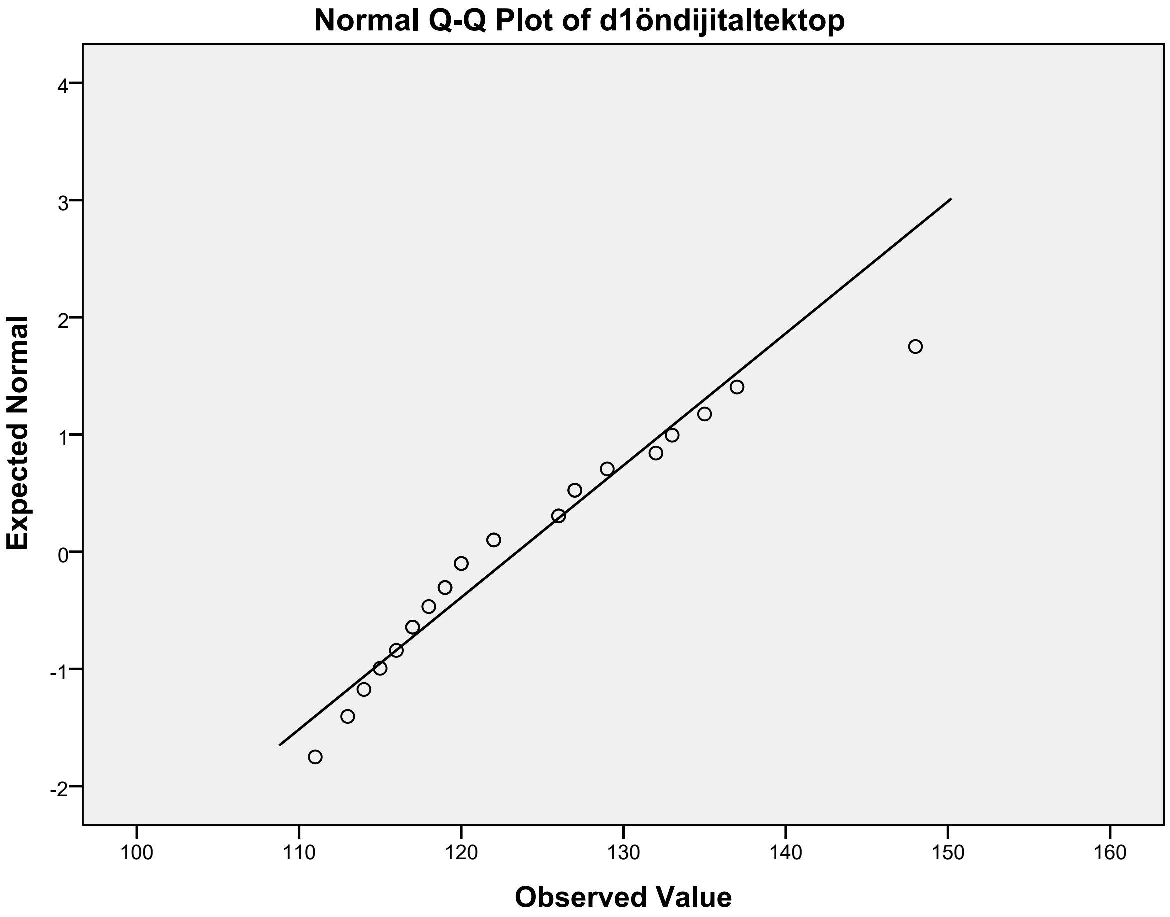
Normal Q-Q Plot of d1öndijitaltektop
Expected Normal
Observed Value
4
3
2
1
0
-1
-2
100
110
120
130
140
150
160

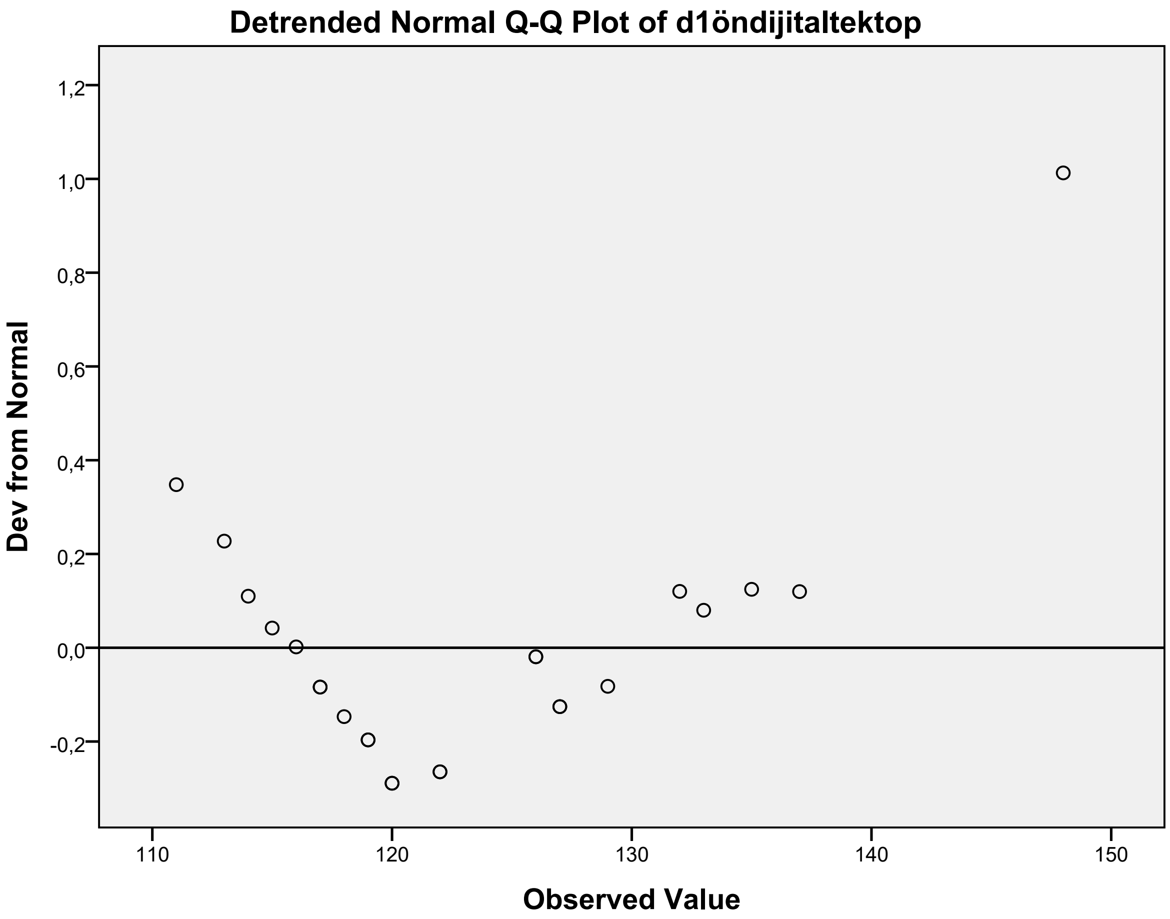

Detrended Normal Q-Q Plot of d1öndijitaltektop
Dev from Normal
1,2
1,0
0,8
0,6
0,4
0,2
0,0
-0,2
110
120
130
140
150
Observed Value

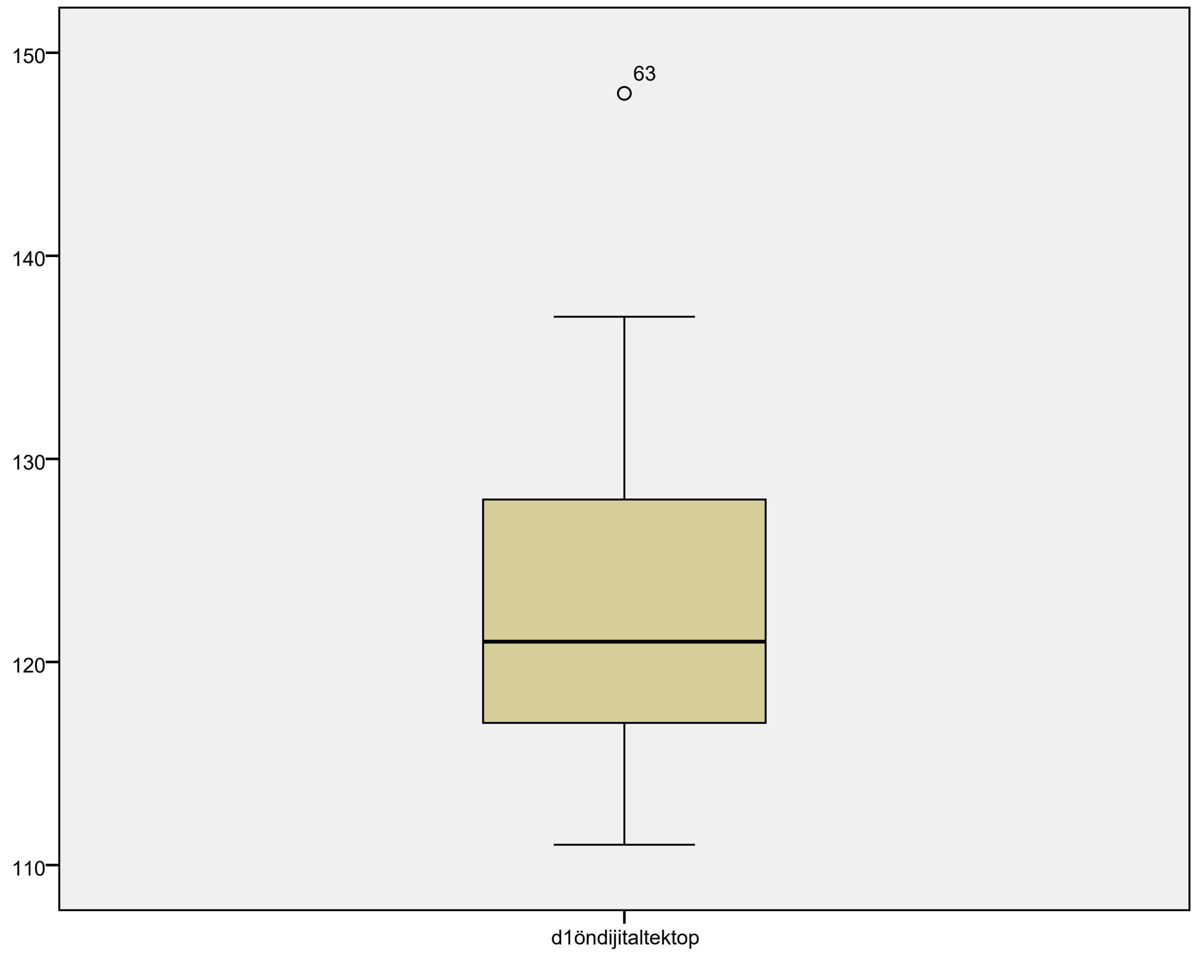

d1önöğrenmestratejileritop

```
d1önöğrenmestratejileritop Stem-and-Leaf Plot

 Frequency    Stem &  Leaf

     1,00 Extremes    (=<114)
     2,00        2 .  24
    15,00        2 .  566777788899999
     6,00        3 .  001144

 Stem width:    100,00
 Each leaf:       1 case(s)
```

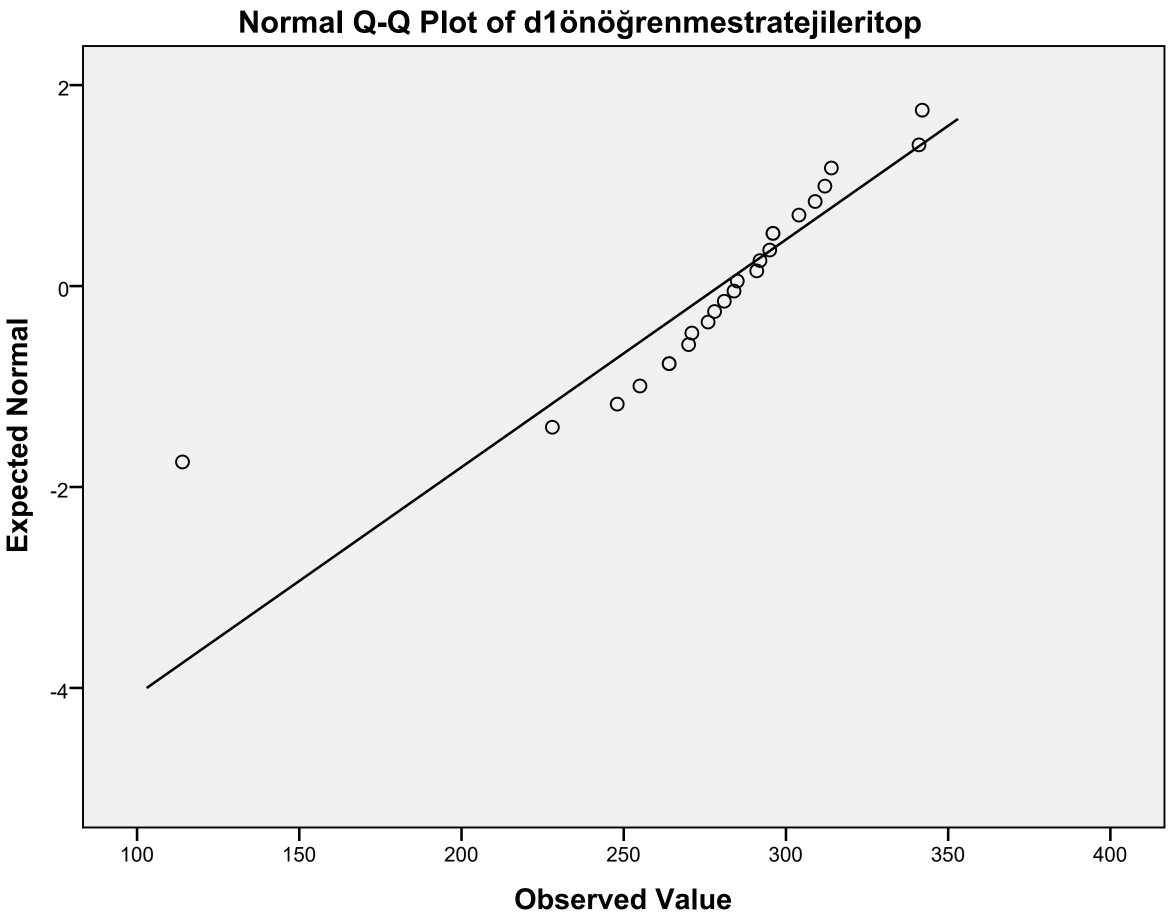
Normal Q-Q Plot of d1önöğrenmestratejileritop
Expected Normal
2
0
-2
-4
100
150
200
250
300
350
400
Observed Value

Detrended Normal Q-Q Plot of d1önöğrenmestratejileritop

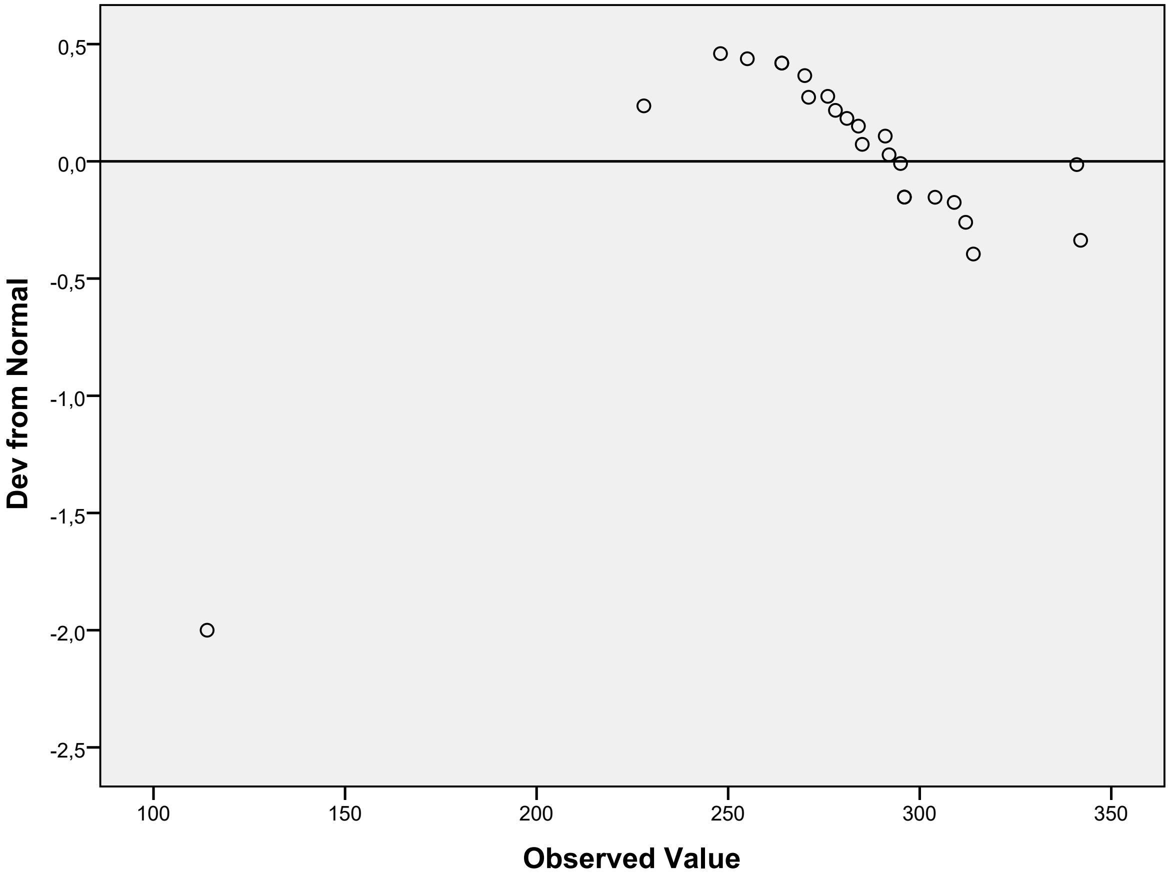

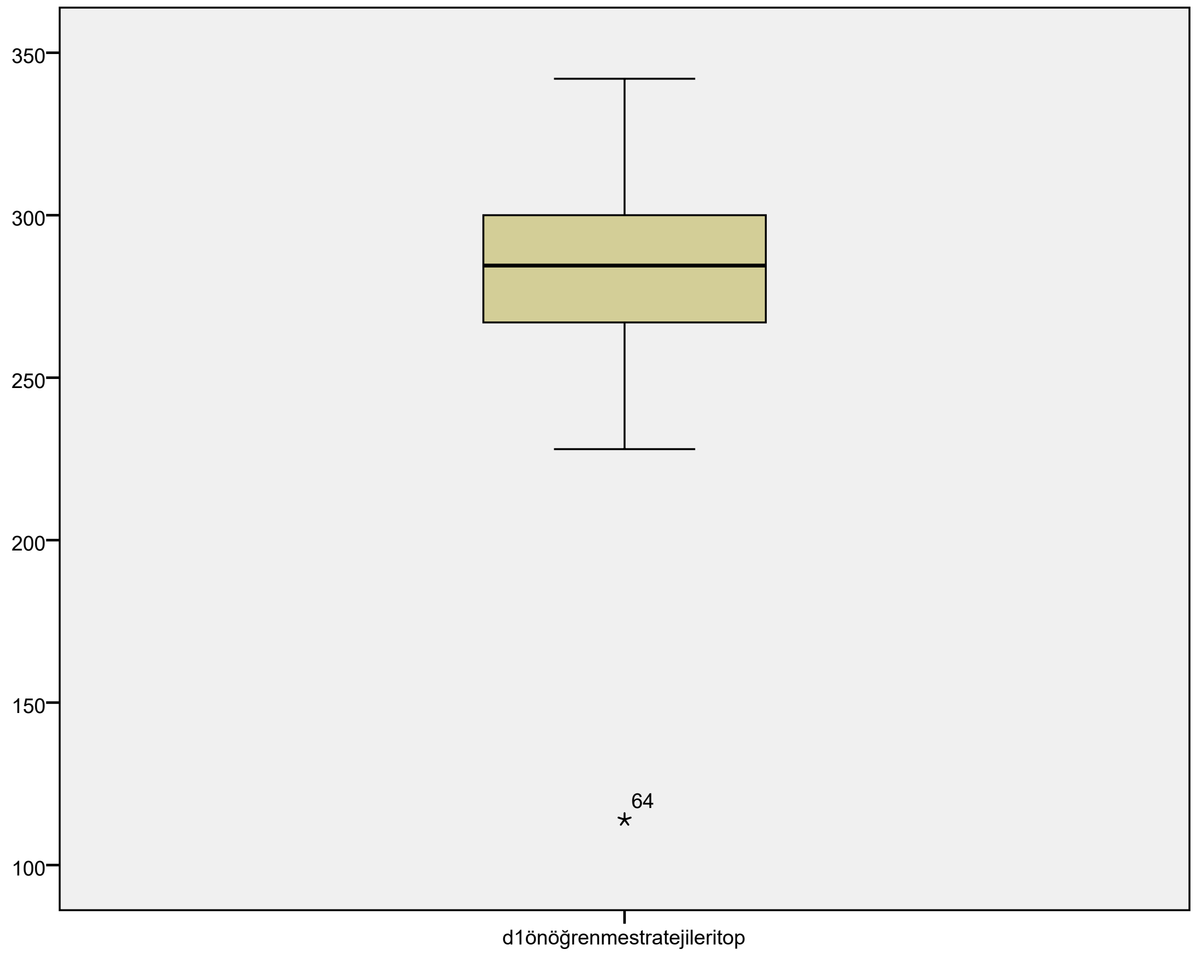

d1sonişbirliktop

```
d1sonişbirliktop Stem-and-Leaf Plot

 Frequency    Stem &  Leaf

     1,00 Extremes    (=<42)
     6,00        5 .  112244
     9,00        5 .  556677799
     4,00        6 .  0113
     1,00        6 .  9
     1,00        7 .  0
     2,00 Extremes    (>=72)

 Stem width:     10,00
 Each leaf:       1 case(s)
```

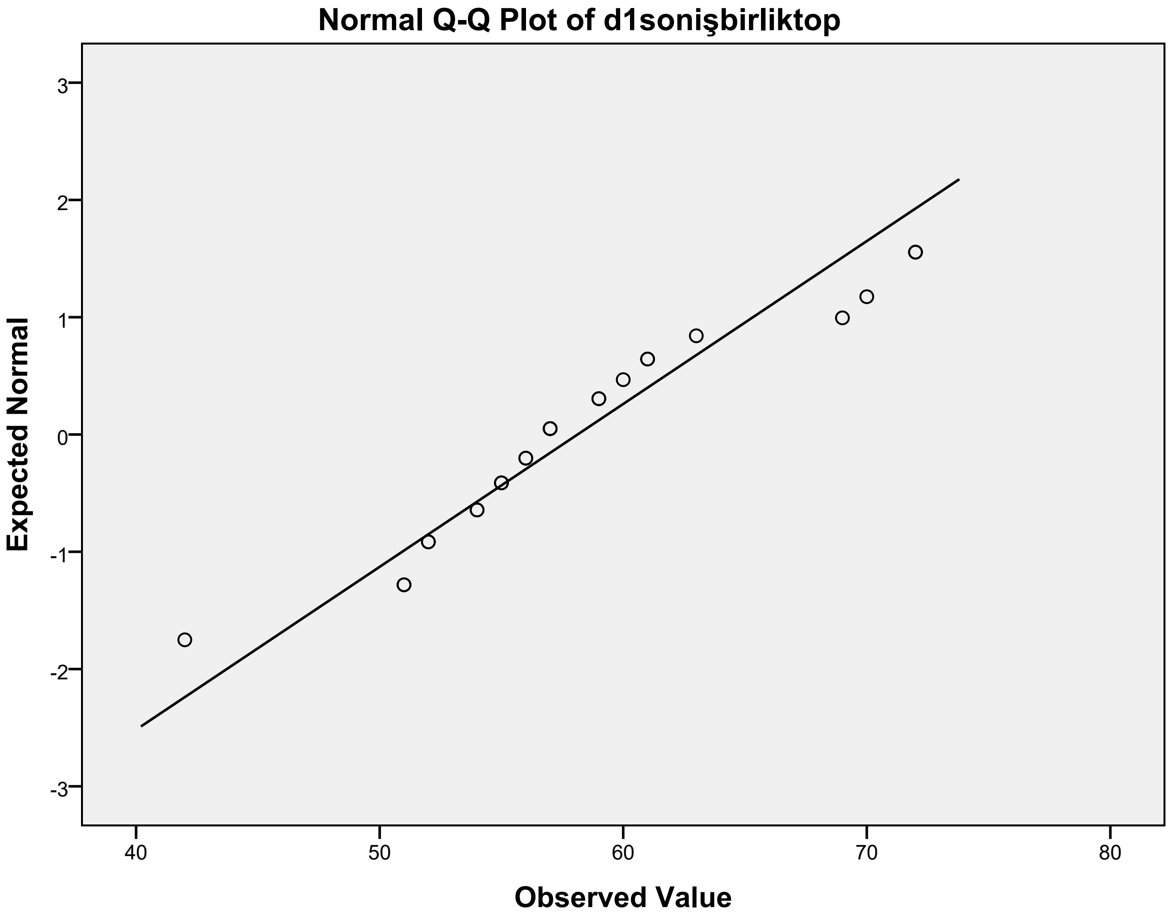

Normal Q-Q Plot of d1sonişbirliktop
Expected Normal
Observed Value
3
2
1
0
-1
-2
-3
40
50
60
70
80

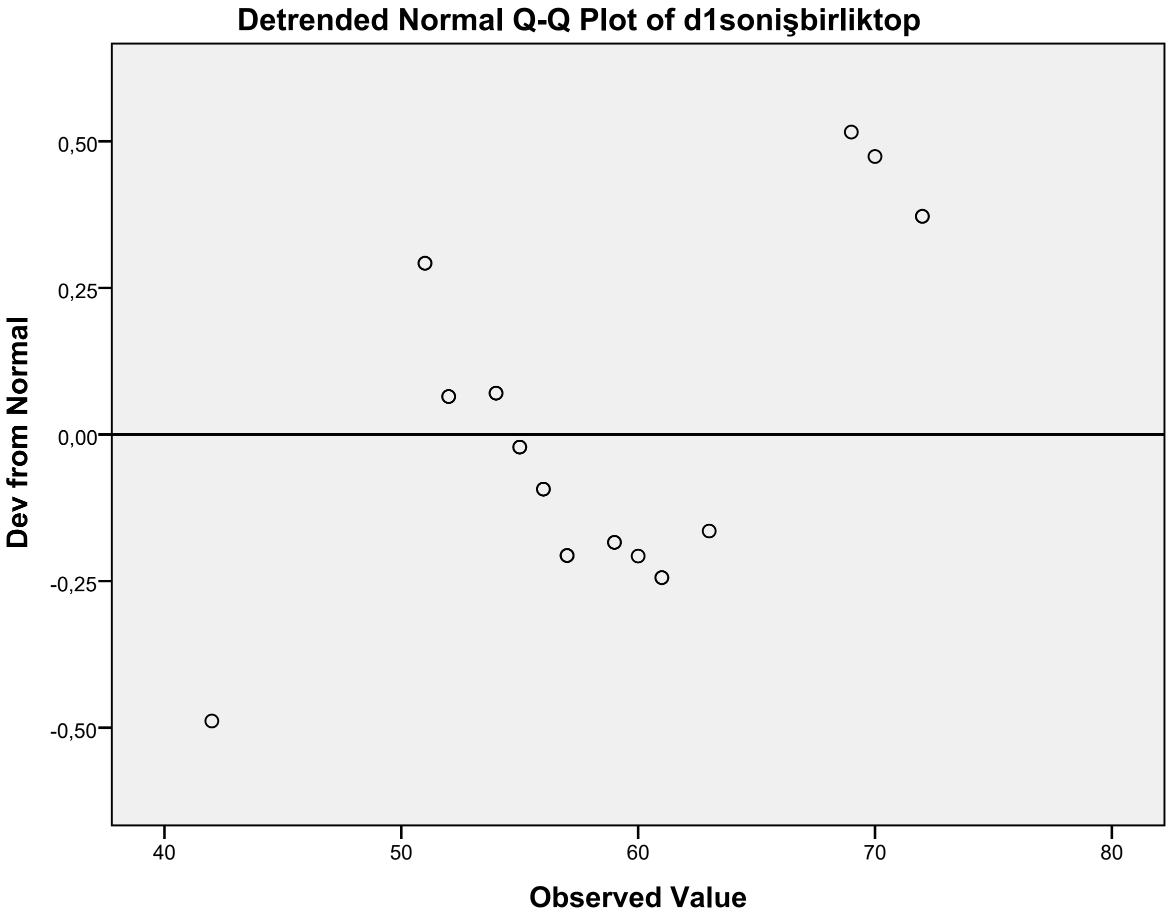
Detrended Normal Q-Q Plot of d1sonişbirliktop
Dev from Normal
0,50
0,25
0,00
-0,25
-0,50
40
50
60
70
80
Observed Value

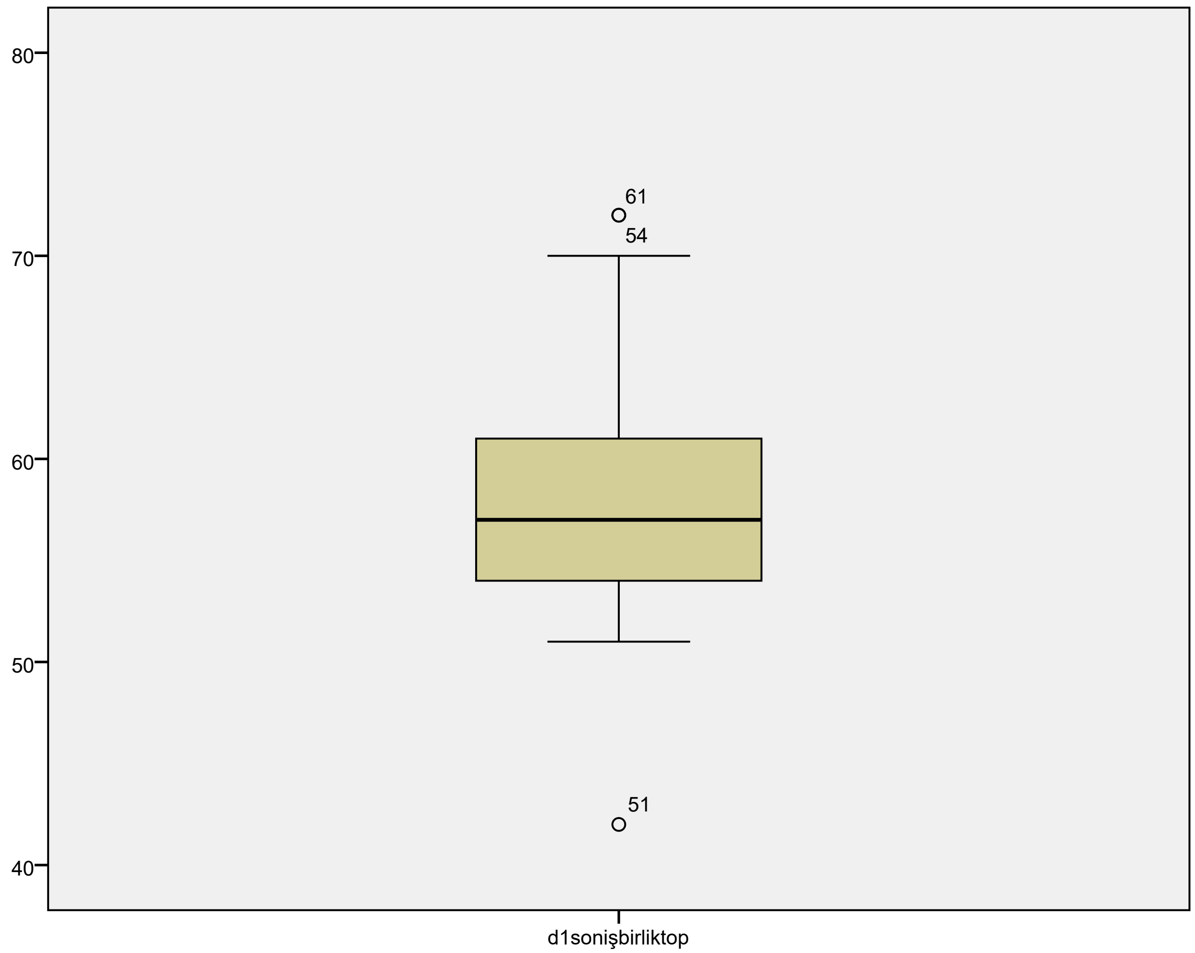

d1sondijitaltektop

```
d1sondijitaltektop Stem-and-Leaf Plot

 Frequency    Stem &  Leaf

     1,00       10 .  3
     2,00       10 .  58
     3,00       11 .  124
     4,00       11 .  5788
     6,00       12 .  022444
     5,00       12 .  55569
     3,00       13 .  024

 Stem width:     10,00
 Each leaf:       1 case(s)
```

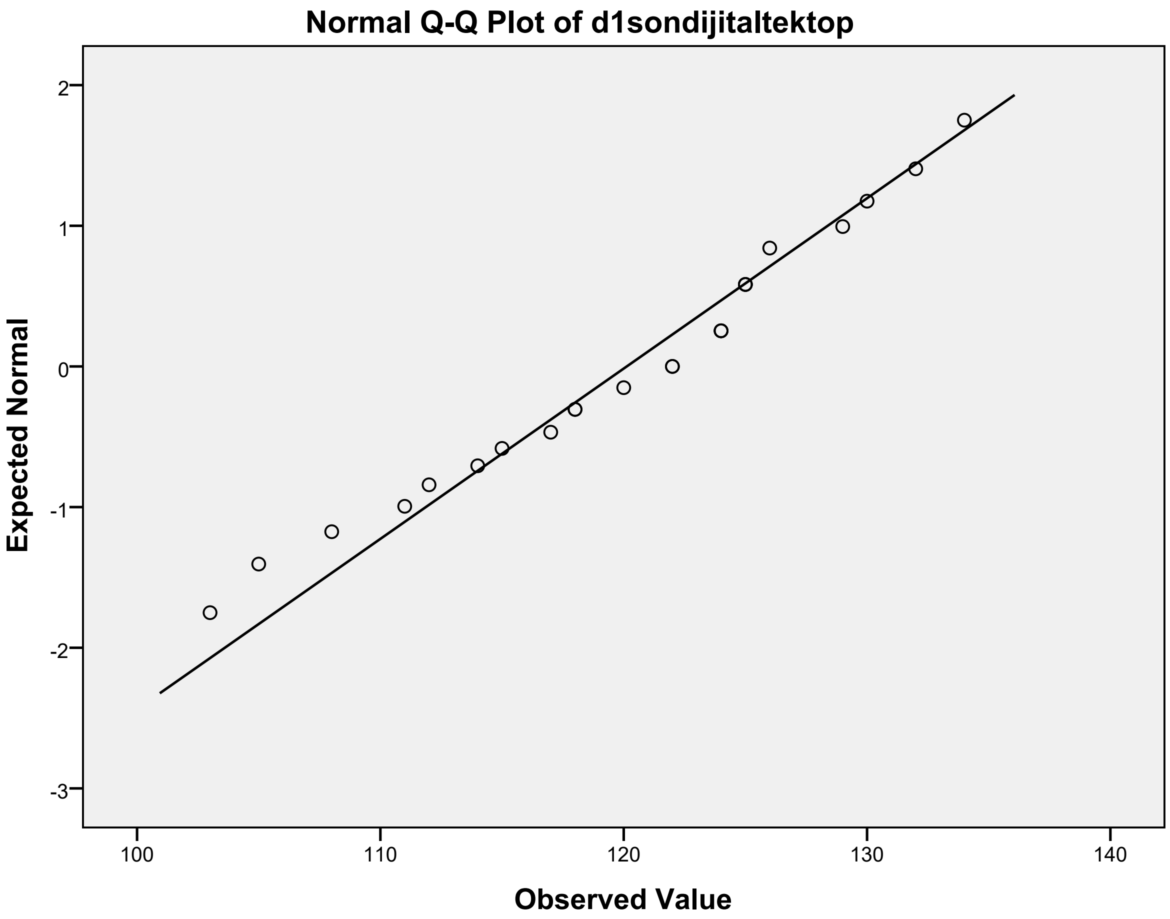
Normal Q-Q Plot of d1sondijitaltektop
Expected Normal
Observed Value
2
1
0
-1
-2
-3
100
110
120
130
140

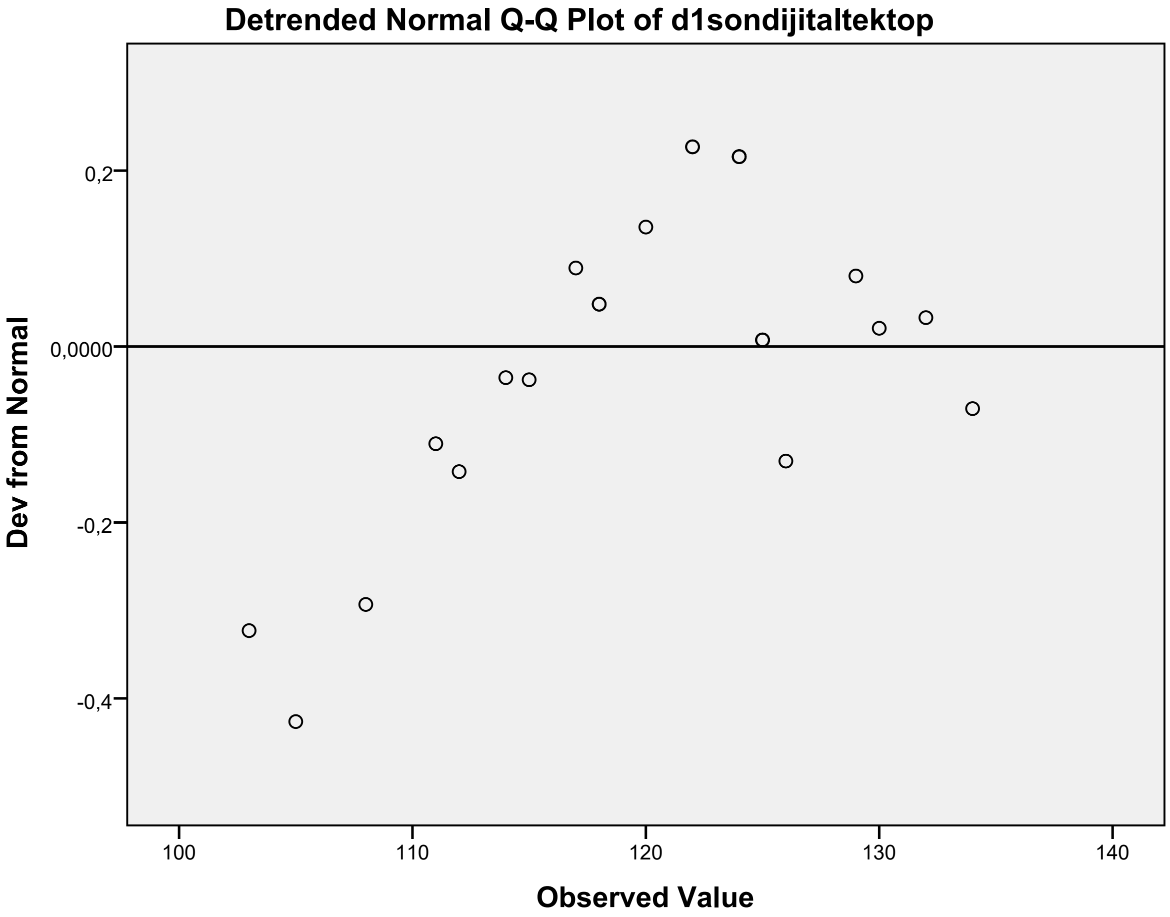

Detrended Normal Q-Q Plot of d1sondijitaltektop
Dev from Normal
0,2
0,0000
-0,2
-0,4
100
110
120
130
140
Observed Value

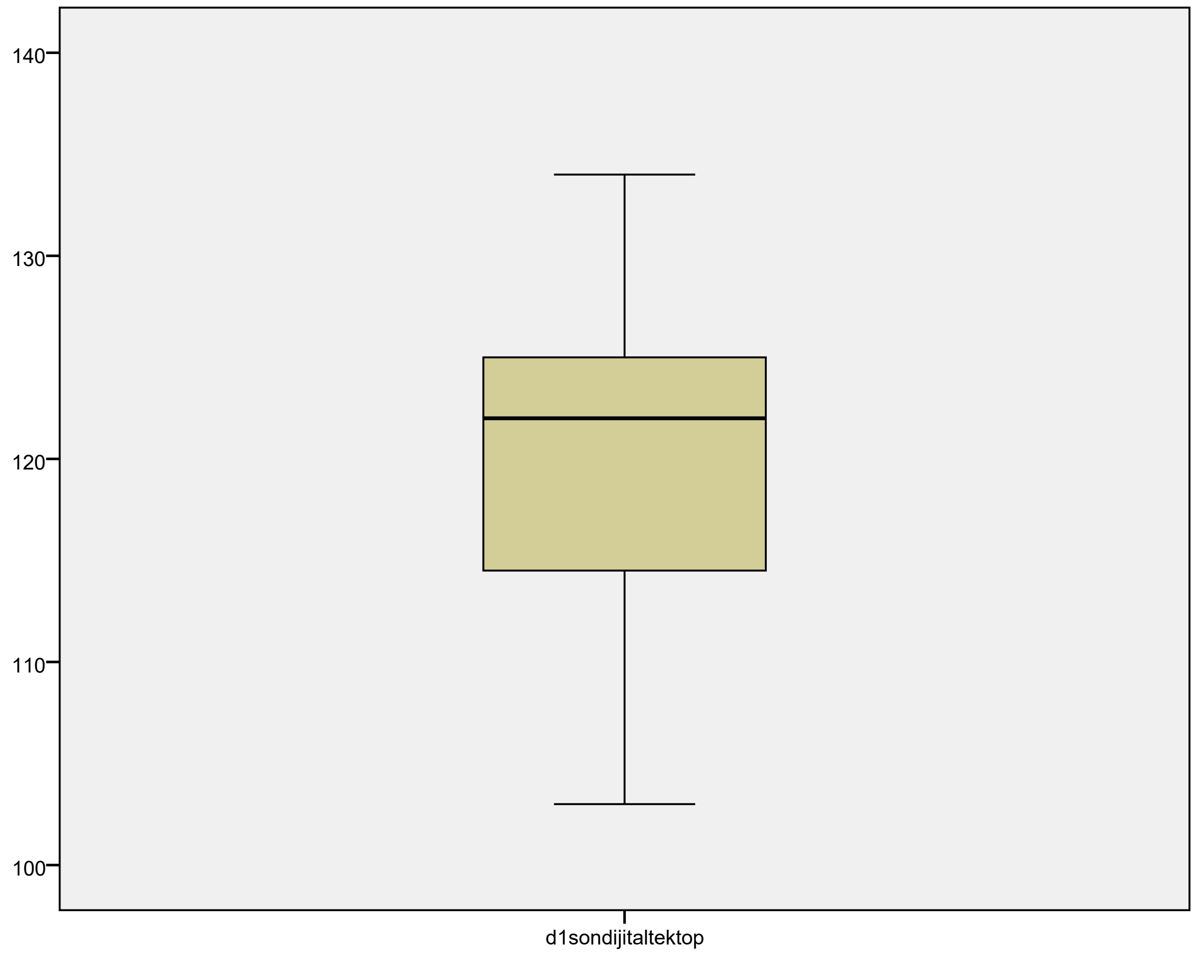

d1sonöğrstratejitop

```
d1sonöğrstratejitop Stem-and-Leaf Plot

 Frequency    Stem &  Leaf

     2,00 Extremes    (=<220)
     1,00       23 .  5
     3,00       24 .  139
     7,00       25 .  3445566
     6,00       26 .  024567
     1,00       27 .  3
     2,00       28 .  09
     2,00 Extremes    (>=300)

 Stem width:     10,00
 Each leaf:       1 case(s)
```

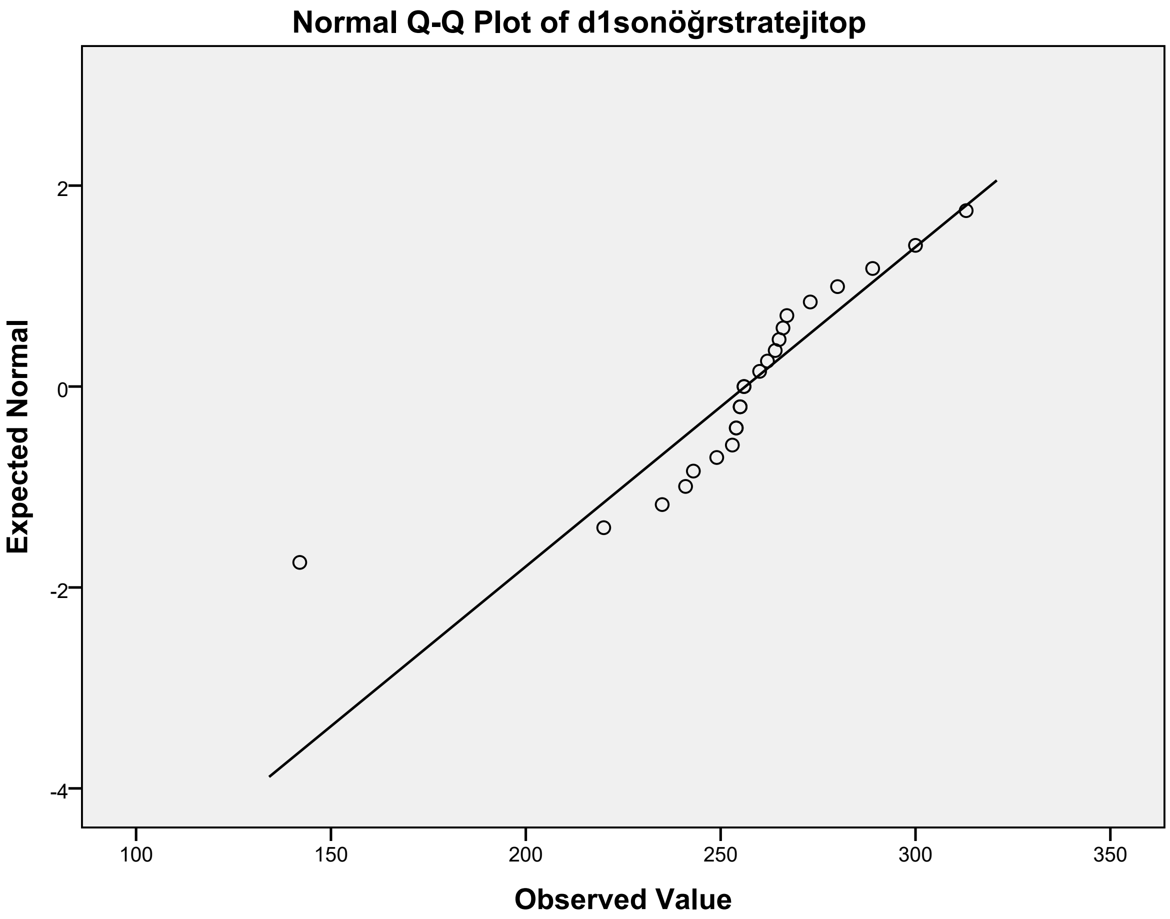
Normal Q-Q Plot of d1sonöğrstratejitop
Expected Normal
Observed Value
2
0
-2
-4
100
150
200
250
300
350

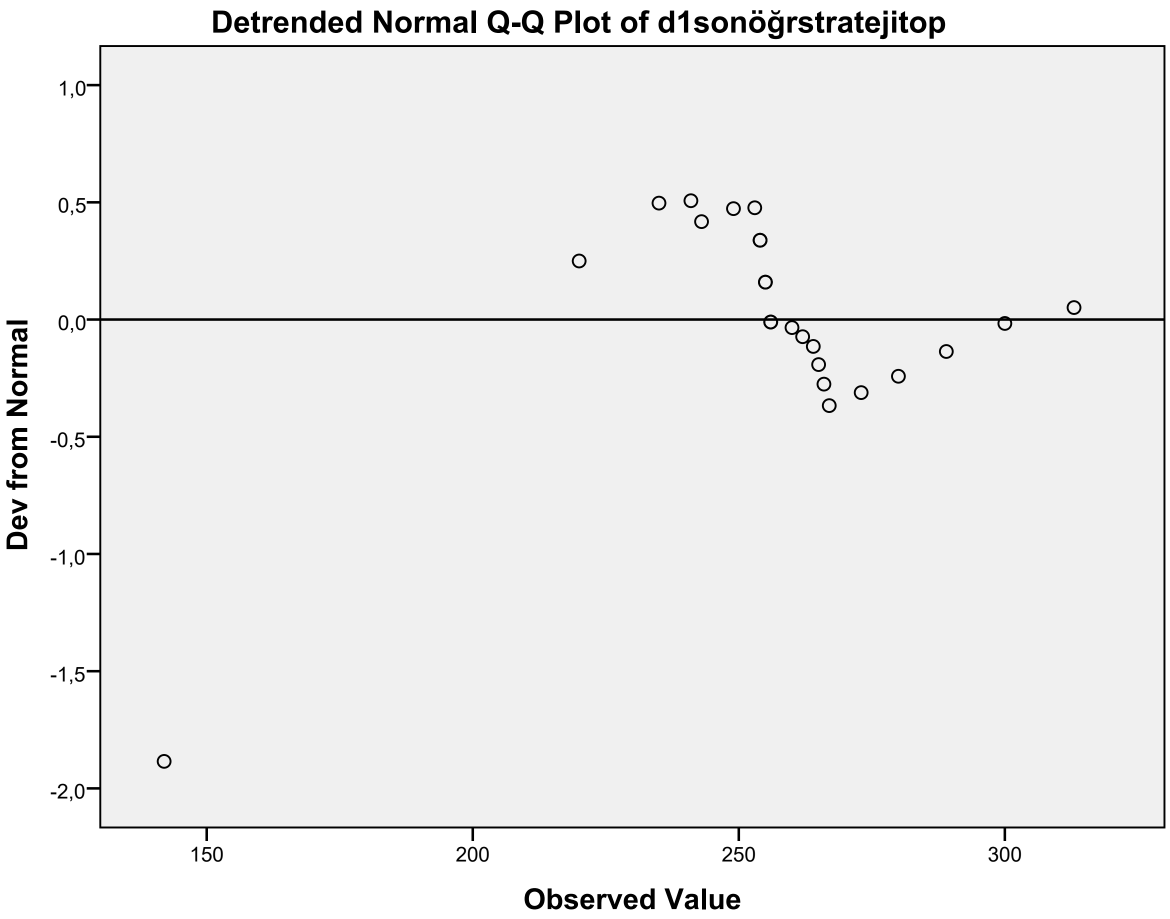
Detrended Normal Q-Q Plot of d1sonöğrstratejitop
Dev from Normal
1,0
0,5
0,0
-0,5
-1,0
-1,5
-2,0
150
200
250
300
Observed Value

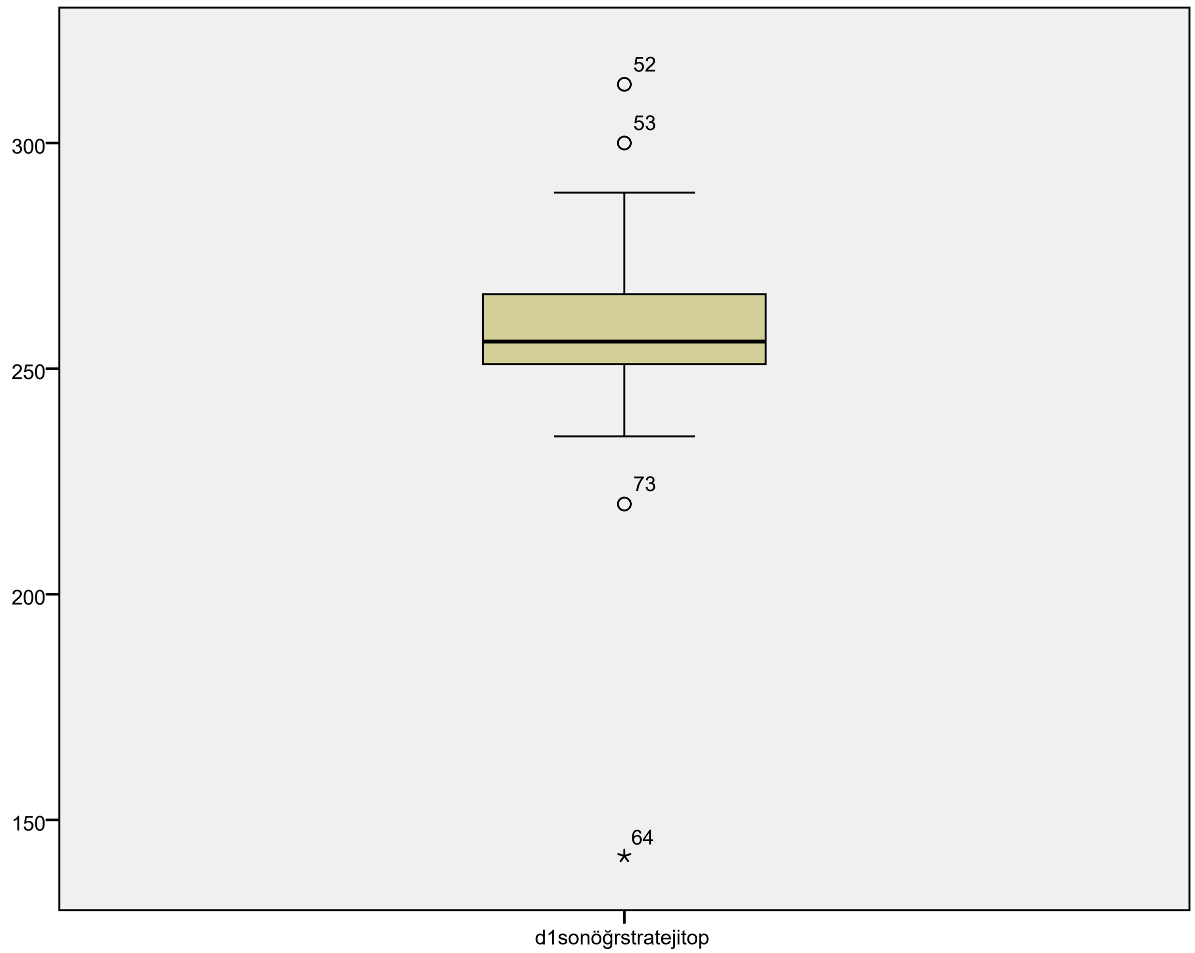


```
 T-
TEST PAIRS=d1önişbirlikçitop d1öndijitaltektop d1önöğrenmestratejileritop WITH d1sonişbirlik
    d1sonöğrstratejitop (PAIRED)
  /CRITERIA=CI(.9500)
  /MISSING=ANALYSIS.
```

T-Test

Notes

Output Created		03-NOV-2024 21:06:32
Comments		
Input	Data	C: \Users\Gizem\Pictures\NİHAL HOCAYLA ÇALIŞMA 2023\tersyüz-web ana analiz\tersyüz-web ana analiz.sav
	Active Dataset	DataSet1
	Filter	grup = 3 (FILTER)
	Weight	<none>
	Split File	<none>
	N of Rows in Working Data File	24
Missing Value Handling	Definition of Missing	User defined missing values are treated as missing.
	Cases Used	Statistics for each analysis are based on the cases with no missing or out-of-range data for any variable in the analysis.
Syntax		T-TEST PAIRS=d1önişbirlikçitop d1öndijitaltektop d1önöğrenmestratejileritop WITH d1sonişbirliktop d1sondijitaltektop d1sonöğrstratejitop (PAIRED) /CRITERIA=CI(.9500) /MISSING=ANALYSIS.
Resources	Processor Time	00:00:00,02
	Elapsed Time	00:00:00,02

```
[DataSet1] C:\Users\Gizem\Pictures\NİHAL HOCAYLA ÇALIŞMA 2023\tersyüz-web
ana analiz\tersyüz-web ana analiz.sav
```

Paired Samples Statistics

		Mean	N	Std. Deviation	Std. Error Mean
Pair 1	d1önişbirlikçitop	59,2917	24	7,54971	1,54108
	d1sonişbirliktop	58,1250	24	7,20092	1,46988
Pair 2	d1öndijitaltektop	123,4583	24	8,88075	1,81278
	d1sondijitaltektop	120,1250	24	8,25839	1,68574
Pair 3	d1önöğrenmestratejileritop	279,5833	24	44,14592	9,01125
	d1sonöğrstratejitop	256,3333	24	31,44998	6,41970

Paired Samples Correlations

		N	Correlation	Sig.
Pair 1	d1önişbirlikçitop & d1sonişbirliktop	24	,910	,000
Pair 2	d1öndijitaltektop & d1sondijitaltektop	24	,348	,096
Pair 3	d1önöğrenmestratejileritop & d1sonöğrstratejitop	24	,950	,000

Paired Samples Test

		Paired Differences			
					95% Confidence ...
		Mean	Std. Deviation	Std. Error Mean	Lower
Pair 1	d1önişbirlikçitop - d1sonişbirliktop	1,16667	3,14389	,64174	-,16088
Pair 2	d1öndijitaltektop - d1sondijitaltektop	3,33333	9,80092	2,00060	-,80523
Pair 3	d1önöğrenmestratejileritop - d1sonöğrstratejitop	23,25000	17,35624	3,54283	15,92110

Paired Samples Test

		Paired ...			
		95% Confidence ...			
		Upper	t	df	Sig. (2-tailed)
Pair 1	d1önişbirlikçitop - d1sonişbirliktop	2,49422	1,818	23	,082
Pair 2	d1öndijitaltektop - d1sondijitaltektop	7,47190	1,666	23	,109
Pair 3	d1önöğrenmestratejileritop - d1sonöğrstratejitop	30,57890	6,563	23	,000

```
 USE ALL.
COMPUTE filter_$=(grup = 1).
VARIABLE LABELS filter_$ 'grup = 1 (FILTER)'.
VALUE LABELS filter_$ 0 'Not Selected' 1 'Selected'.
FORMATS filter_$ (f1.0).
FILTER BY filter_$.
EXECUTE.
T-
TEST PAIRS=d1önişbirlikçitop d1öndijitaltektop d1önöğrenmestratejileritop WITH d1sonişbirlik
    d1sonöğrstratejitop (PAIRED)
  /CRITERIA=CI(.9500)
  /MISSING=ANALYSIS.
```

T-Test

Notes

Output Created		03-NOV-2024 21:09:56
Comments		
Input	Data	C:\Users\Gizem\Pictures\NİHAL HOCAYLA ÇALIŞMA 2023\tersyüz-web ana analiz\tersyüz-web ana analiz.sav
	Active Dataset	DataSet1
	Filter	grup = 1 (FILTER)
	Weight	<none>
	Split File	<none>
	N of Rows in Working Data File	26
Missing Value Handling	Definition of Missing	User defined missing values are treated as missing.
	Cases Used	Statistics for each analysis are based on the cases with no missing or out-of-range data for any variable in the analysis.
Syntax		T-TEST PAIRS=d1önişbirlikçitop d1öndijitaltektop d1önöğrenmestratejileritop WITH d1sonişbirliktop d1sondijitaltektop d1sonöğrstratejitop (PAIRED) /CRITERIA=CI(.9500) /MISSING=ANALYSIS.
Resources	Processor Time	00:00:00,02
	Elapsed Time	00:00:00,02

```
[DataSet1] C:\Users\Gizem\Pictures\NİHAL HOCAYLA ÇALIŞMA 2023\tersyüz-web
ana analiz\tersyüz-web ana analiz.sav
```

Paired Samples Statistics

		Mean	N	Std. Deviation	Std. Error Mean
Pair 1	d1önişbirlikçitop	64,4615	26	7,13992	1,40025
	d1sonişbirliktop	73,3846	26	11,11783	2,18039
Pair 2	d1öndijitaltektop	129,3846	26	13,84797	2,71581
	d1sondijitaltektop	135,1923	26	11,34203	2,22435
Pair 3	d1önöğrenmestratejileritop	272,2308	26	36,13564	7,08678
	d1sonöğrstratejitop	297,7308	26	37,95108	7,44282

Paired Samples Correlations

		N	Correlation	Sig.
Pair 1	d1önişbirlikçitop & d1sonişbirliktop	26	,569	,002
Pair 2	d1öndijitaltektop & d1sondijitaltektop	26	,641	,000
Pair 3	d1önöğrenmestratejileritop & d1sonöğrstratejitop	26	,757	,000

Paired Samples Test

		Paired Differences			
					95% Confidence ...
		Mean	Std. Deviation	Std. Error Mean	Lower
Pair 1	d1önişbirlikçitop - d1sonişbirliktop	-8,92308	9,18226	1,80079	-12,63187
Pair 2	d1öndijitaltektop - d1sondijitaltektop	-5,80769	10,90695	2,13903	-10,21310
Pair 3	d1önöğrenmestratejileritop - d1sonöğrstratejitop	-25,50000	25,89401	5,07823	-35,95882

Paired Samples Test

		Paired ...			
		95% Confidence ...			
		Upper	t	df	Sig. (2-tailed)
Pair 1	d1önişbirlikçitop - d1sonişbirliktop	-5,21428	-4,955	25	,000
Pair 2	d1öndijitaltektop - d1sondijitaltektop	-1,40228	-2,715	25	,012
Pair 3	d1önöğrenmestratejileritop - d1sonöğrstratejitop	-15,04118	-5,021	25	,000

```
 USE ALL.
COMPUTE filter_$=(grup = 2).
VARIABLE LABELS filter_$ 'grup = 2 (FILTER)'.
VALUE LABELS filter_$ 0 'Not Selected' 1 'Selected'.
FORMATS filter_$ (f1.0).
FILTER BY filter_$.
EXECUTE.
T-
TEST PAIRS=d1önişbirlikçitop d1öndijitaltektop d1önöğrenmestratejileritop WITH d1sonişbirlik
    d1sonöğrstratejitop (PAIRED)
  /CRITERIA=CI(.9500)
  /MISSING=ANALYSIS.
```

T-Test

Notes

Output Created		03-NOV-2024 21:10:48
Comments		
Input	Data	C: \Users\Gizem\Pictures\NİHAL HOCAYLA ÇALIŞMA 2023\tersyüz-web ana analiz\tersyüz-web ana analiz.sav
	Active Dataset	DataSet1
	Filter	grup = 2 (FILTER)
	Weight	<none>
	Split File	<none>
	N of Rows in Working Data File	24
Missing Value Handling	Definition of Missing	User defined missing values are treated as missing.
	Cases Used	Statistics for each analysis are based on the cases with no missing or out-of-range data for any variable in the analysis.
Syntax		T-TEST PAIRS=d1önişbirlikçitop d1öndijitaltektop d1önöğrenmestratejileritop WITH d1sonişbirliktop d1sondijitaltektop d1sonöğrstratejitop (PAIRED) /CRITERIA=CI(.9500) /MISSING=ANALYSIS.
Resources	Processor Time	00:00:00,02
	Elapsed Time	00:00:00,02

```
[DataSet1] C:\Users\Gizem\Pictures\NİHAL HOCAYLA ÇALIŞMA 2023\tersyüz-web
ana analiz\tersyüz-web ana analiz.sav
```

Paired Samples Statistics

		Mean	N	Std. Deviation	Std. Error Mean
Pair 1	d1önişbirlikçitop	61,7083	24	9,42928	1,92474
	d1sonişbirliktop	64,2917	24	13,13965	2,68212
Pair 2	d1öndijitaltektop	129,5000	24	8,20392	1,67462
	d1sondijitaltektop	144,5833	24	14,51211	2,96227
Pair 3	d1önöğrenmestratejileritop	262,4583	24	24,16426	4,93251
	d1sonöğrstratejitop	280,4583	24	29,97967	6,11958

Paired Samples Correlations

		N	Correlation	Sig.
Pair 1	d1önişbirlikçitop & d1sonişbirliktop	24	,956	,000
Pair 2	d1öndijitaltektop & d1sondijitaltektop	24	,767	,000
Pair 3	d1önöğrenmestratejileritop & d1sonöğrstratejitop	24	,914	,000

Paired Samples Test

		Paired Differences			
					95% Confidence ...
		Mean	Std. Deviation	Std. Error Mean	Lower
Pair 1	d1önişbirlikçitop - d1sonişbirliktop	-2,58333	4,97749	1,01602	-4,68514
Pair 2	d1öndijitaltektop - d1sondijitaltektop	-15,08333	9,76202	1,99266	-19,20547
Pair 3	d1önöğrenmestratejileritop - d1sonöğrstratejitop	-18,00000	12,60780	2,57356	-23,32381

Paired Samples Test

		Paired ...			
		95% Confidence ...			
		Upper	t	df	Sig. (2-tailed)
Pair 1	d1önişbirlikçitop - d1sonişbirliktop	-,48153	-2,543	23	,018
Pair 2	d1öndijitaltektop - d1sondijitaltektop	-10,96119	-7,569	23	,000
Pair 3	d1önöğrenmestratejileritop - d1sonöğrstratejitop	-12,67619	-6,994	23	,000

```
ONEWAY d1önişbirlikçitop d1öndijitaltektop d1önöğrenmestratejileritop d1sonişbirliktop d1son
    BY grup
  /MISSING ANALYSIS
  /POSTHOC=TUKEY SCHEFFE ALPHA(0.05).
```

Oneway

Notes

Output Created		03-NOV-2024 21:11:57
Comments		
Input	Data	C: \Users\Gizem\Pictures\NİHAL HOCAYLA ÇALIŞMA 2023\tersyüz-web ana analiz\tersyüz-web ana analiz.sav
	Active Dataset	DataSet1
	Filter	grup = 2 (FILTER)
	Weight	<none>
	Split File	<none>
	N of Rows in Working Data File	24
Missing Value Handling	Definition of Missing	User-defined missing values are treated as missing.
	Cases Used	Statistics for each analysis are based on cases with no missing data for any variable in the analysis.
Syntax		ONEWAY d1önişbirlikçitop d1öndijitaltektop d1önöğrenmestratejileritop d1sonişbirliktop d1sondijitaltektop d1sonöğrstratejitop BY grup /MISSING ANALYSIS /POSTHOC=TUKEY SCHEFFE ALPHA(0.05).
Resources	Processor Time	00:00:00,02
	Elapsed Time	00:00:00,02

```
[DataSet1] C:\Users\Gizem\Pictures\NİHAL HOCAYLA ÇALIŞMA 2023\tersyüz-web
ana analiz\tersyüz-web ana analiz.sav
```

Warnings

There are fewer than two groups for dependent variable d1önişbirlikçitop. No statistics are computed.
There are fewer than two groups for dependent variable d1öndijitaltektop. No statistics are computed.
There are fewer than two groups for dependent variable d1önöğrenmestratejileritop. No statistics are computed.
There are fewer than two groups for dependent variable d1sonişbirliktop. No statistics are computed.
There are fewer than two groups for dependent variable d1sondijitaltektop. No statistics are computed.
There are fewer than two groups for dependent variable d1sonöğrstratejitop. No statistics are computed.

```
 FILTER OFF.
USE ALL.
EXECUTE.
ONEWAY d1önişbirlikçitop d1öndijitaltektop d1önöğrenmestratejileritop d1sonişbirliktop d1son
    BY grup
  /MISSING ANALYSIS
  /POSTHOC=TUKEY SCHEFFE ALPHA(0.05).
```

Oneway

Notes

Output Created		03-NOV-2024 21:12:09
Comments		
Input	Data	C: \Users\Gizem\Pictures\NİHAL HOCAYLA ÇALIŞMA 2023\tersyüz-web ana analiz\tersyüz-web ana analiz.sav
	Active Dataset	DataSet1
	Filter	<none>
	Weight	<none>
	Split File	<none>
	N of Rows in Working Data File	74
Missing Value Handling	Definition of Missing	User-defined missing values are treated as missing.
	Cases Used	Statistics for each analysis are based on cases with no missing data for any variable in the analysis.
Syntax		ONEWAY d1önişbirlikçitop d1öndijitaltektop d1önöğrenmestratejileritop d1sonişbirliktop d1sondijitaltektop d1sonöğrstratejitop BY grup /MISSING ANALYSIS /POSTHOC=TUKEY SCHEFFE ALPHA(0.05).
Resources	Processor Time	00:00:00,08
	Elapsed Time	00:00:00,08

```
[DataSet1] C:\Users\Gizem\Pictures\NİHAL HOCAYLA ÇALIŞMA 2023\tersyüz-web
ana analiz\tersyüz-web ana analiz.sav
```

ANOVA

		Sum of Squares	df	Mean Square	F
d1önişbirlikçitop	Between Groups	334,757	2	167,378	2,567
	Within Groups	4630,378	71	65,217	
	Total	4965,135	73		
d1öndijitaltektop	Between Groups	580,388	2	290,194	2,526
	Within Groups	8156,112	71	114,875	
	Total	8736,500	73		
d1önöğrenmestratejilerito p	Between Groups	3543,877	2	1771,938	1,384
	Within Groups	90898,407	71	1280,259	
	Total	94442,284	73		
d1sonişbirliktop	Between Groups	2956,749	2	1478,375	12,717
	Within Groups	8253,737	71	116,250	
	Total	11210,486	73		
d1sondijitaltektop	Between Groups	7314,368	2	3657,184	26,968
	Within Groups	9628,497	71	135,613	
	Total	16942,865	73		
d1sonöğrstratejitop	Between Groups	21497,052	2	10748,526	9,608
	Within Groups	79428,407	71	1118,710	
	Total	100925,459	73		

ANOVA

		Sig.
d1önişbirlikçitop	Between Groups	,084
	Within Groups	
	Total	
d1öndijitaltektop	Between Groups	,087
	Within Groups	
	Total	
d1önöğrenmestratejilerito p	Between Groups	,257
	Within Groups	
	Total	
d1sonişbirliktop	Between Groups	,000
	Within Groups	
	Total	
d1sondijitaltektop	Between Groups	,000
	Within Groups	
	Total	
d1sonöğrstratejitop	Between Groups	,000
	Within Groups	
	Total	

Post Hoc Tests

Multiple Comparisons

Dependent Variable		(I) grup	(J) grup	Mean Difference (I-J)	Std. Error	Sig.
d1önişbirlikçitop	Tukey HSD	1,00	2,00	2,75321	2,28598	,455
			3,00	5,16987	2,28598	,068
		2,00	1,00	-2,75321	2,28598	,455
			3,00	2,41667	2,33125	,556
		3,00	1,00	-5,16987	2,28598	,068
			2,00	-2,41667	2,33125	,556
	Scheffe	1,00	2,00	2,75321	2,28598	,488
			3,00	5,16987	2,28598	,085
		2,00	1,00	-2,75321	2,28598	,488
			3,00	2,41667	2,33125	,587
		3,00	1,00	-5,16987	2,28598	,085
			2,00	-2,41667	2,33125	,587
d1öndijitaltektop	Tukey HSD	1,00	2,00	-,11538	3,03393	,999
			3,00	5,92628	3,03393	,131
		2,00	1,00	,11538	3,03393	,999
			3,00	6,04167	3,09401	,132
		3,00	1,00	-5,92628	3,03393	,131
			2,00	-6,04167	3,09401	,132
	Scheffe	1,00	2,00	-,11538	3,03393	,999
			3,00	5,92628	3,03393	,156
		2,00	1,00	,11538	3,03393	,999
			3,00	6,04167	3,09401	,156
		3,00	1,00	-5,92628	3,03393	,156
			2,00	-6,04167	3,09401	,156
d1önöğrenmestratejileritop	Tukey HSD	1,00	2,00	9,77244	10,12842	,601
			3,00	-7,35256	10,12842	,749
		2,00	1,00	-9,77244	10,12842	,601
			3,00	-17,12500	10,32900	,229
		3,00	1,00	7,35256	10,12842	,749
			2,00	17,12500	10,32900	,229
	Scheffe	1,00	2,00	9,77244	10,12842	,630
			3,00	-7,35256	10,12842	,769
		2,00	1,00	-9,77244	10,12842	,630
			3,00	-17,12500	10,32900	,260
		3,00	1,00	7,35256	10,12842	,769
			2,00	17,12500	10,32900	,260
d1sonişbirliktop	Tukey HSD	1,00	2,00	9,09295*	3,05203	,011
			3,00	15,25962*	3,05203	,000
		2,00	1,00	-9,09295*	3,05203	,011
			3,00	6,16667	3,11247	,124

Multiple Comparisons

Dependent Variable		(I) grup	(J) grup	95% Confidence Interval Lower Bound	Upper Bound
d1önişbirlikçitop	Tukey HSD	1,00	2,00	-2,7190	8,2254
			3,00	-,3024	10,6421
		2,00	1,00	-8,2254	2,7190
			3,00	-3,1639	7,9973
		3,00	1,00	-10,6421	,3024
			2,00	-7,9973	3,1639
	Scheffe	1,00	2,00	-2,9624	8,4688
			3,00	-,5458	10,8855
		2,00	1,00	-8,4688	2,9624
			3,00	-3,4122	8,2455
		3,00	1,00	-10,8855	,5458
			2,00	-8,2455	3,4122
d1öndijitaltektop	Tukey HSD	1,00	2,00	-7,3781	7,1473
			3,00	-1,3364	13,1890
		2,00	1,00	-7,1473	7,3781
			3,00	-1,3649	13,4482
		3,00	1,00	-13,1890	1,3364
			2,00	-13,4482	1,3649
	Scheffe	1,00	2,00	-7,7011	7,4704
			3,00	-1,6595	13,5120
		2,00	1,00	-7,4704	7,7011
			3,00	-1,6943	13,7776
		3,00	1,00	-13,5120	1,6595
			2,00	-13,7776	1,6943
d1önöğrenmestratejileritop	Tukey HSD	1,00	2,00	-14,4733	34,0182
			3,00	-31,5983	16,8932
		2,00	1,00	-34,0182	14,4733
			3,00	-41,8509	7,6009
		3,00	1,00	-16,8932	31,5983
			2,00	-7,6009	41,8509
	Scheffe	1,00	2,00	-15,5517	35,0966
			3,00	-32,6767	17,9716
		2,00	1,00	-35,0966	15,5517
			3,00	-42,9507	8,7007
		3,00	1,00	-17,9716	32,6767
			2,00	-8,7007	42,9507
d1sonişbirliktop	Tukey HSD	1,00	2,00	1,7869	16,3990
			3,00	7,9536	22,5657
		2,00	1,00	-16,3990	-1,7869
			3,00	-1,2841	13,6174

Multiple Comparisons

Dependent Variable		(I) grup	(J) grup	Mean Difference (I-J)	Std. Error	Sig.
		3,00	1,00	-15,25962*	3,05203	,000
			2,00	-6,16667	3,11247	,124
	Scheffe	1,00	2,00	9,09295*	3,05203	,015
			3,00	15,25962*	3,05203	,000
		2,00	1,00	-9,09295*	3,05203	,015
			3,00	6,16667	3,11247	,148
		3,00	1,00	-15,25962*	3,05203	,000
			2,00	-6,16667	3,11247	,148
d1sondijitaltektop	Tukey HSD	1,00	2,00	-9,39103*	3,29642	,016
			3,00	15,06731*	3,29642	,000
		2,00	1,00	9,39103*	3,29642	,016
			3,00	24,45833*	3,36170	,000
		3,00	1,00	-15,06731*	3,29642	,000
			2,00	-24,45833*	3,36170	,000
	Scheffe	1,00	2,00	-9,39103*	3,29642	,021
			3,00	15,06731*	3,29642	,000
		2,00	1,00	9,39103*	3,29642	,021
			3,00	24,45833*	3,36170	,000
		3,00	1,00	-15,06731*	3,29642	,000
			2,00	-24,45833*	3,36170	,000
d1sonöğrstratejitop	Tukey HSD	1,00	2,00	17,27244	9,46785	,169
			3,00	41,39744*	9,46785	,000
		2,00	1,00	-17,27244	9,46785	,169
			3,00	24,12500*	9,65535	,039
		3,00	1,00	-41,39744*	9,46785	,000
			2,00	-24,12500*	9,65535	,039
	Scheffe	1,00	2,00	17,27244	9,46785	,197
			3,00	41,39744*	9,46785	,000
		2,00	1,00	-17,27244	9,46785	,197
			3,00	24,12500	9,65535	,050
		3,00	1,00	-41,39744*	9,46785	,000
			2,00	-24,12500	9,65535	,050

Multiple Comparisons

Dependent Variable		(I) grup	(J) grup	95% Confidence Interval Lower Bound	Upper Bound
		3,00	1,00	-22,5657	-7,9536
			2,00	-13,6174	1,2841
	Scheffe	1,00	2,00	1,4619	16,7240
			3,00	7,6286	22,8906
		2,00	1,00	-16,7240	-1,4619
			3,00	-1,6155	13,9488
		3,00	1,00	-22,8906	-7,6286
			2,00	-13,9488	1,6155
d1sondijitaltektop	Tukey HSD	1,00	2,00	-17,2821	-1,4999
			3,00	7,1762	22,9584
		2,00	1,00	1,4999	17,2821
			3,00	16,4110	32,5057
		3,00	1,00	-22,9584	-7,1762
			2,00	-32,5057	-16,4110
	Scheffe	1,00	2,00	-17,6331	-1,1490
			3,00	6,8252	23,3094
		2,00	1,00	1,1490	17,6331
			3,00	16,0530	32,8636
		3,00	1,00	-23,3094	-6,8252
			2,00	-32,8636	-16,0530
d1sonöğrstratejitop	Tukey HSD	1,00	2,00	-5,3920	39,9369
			3,00	18,7330	64,0619
		2,00	1,00	-39,9369	5,3920
			3,00	1,0117	47,2383
		3,00	1,00	-64,0619	-18,7330
			2,00	-47,2383	-1,0117
	Scheffe	1,00	2,00	-6,4001	40,9450
			3,00	17,7249	65,0700
		2,00	1,00	-40,9450	6,4001
			3,00	-,0163	48,2663
		3,00	1,00	-65,0700	-17,7249
			2,00	-48,2663	,0163

*. The mean difference is significant at the 0.05 level.

Homogeneous Subsets

d1önişbirlikçitop

	grup	N	Subset for alpha = 0.05
			1
Tukey HSD[a,b]	3,00	24	59,2917
	2,00	24	61,7083
	1,00	26	64,4615
	Sig.		,070
Scheffe[a,b]	3,00	24	59,2917
	2,00	24	61,7083
	1,00	26	64,4615
	Sig.		,087

Means for groups in homogeneous subsets are displayed.

a. Uses Harmonic Mean Sample Size = 24,632.

b. The group sizes are unequal. The harmonic mean of the group sizes is used. Type I error levels are not guaranteed.

d1öndijitaltektop

	grup	N	Subset for alpha = 0.05
			1
Tukey HSD[a,b]	3,00	24	123,4583
	1,00	26	129,3846
	2,00	24	129,5000
	Sig.		,125
Scheffe[a,b]	3,00	24	123,4583
	1,00	26	129,3846
	2,00	24	129,5000
	Sig.		,149

Means for groups in homogeneous subsets are displayed.

a. Uses Harmonic Mean Sample Size = 24,632.

b. The group sizes are unequal. The harmonic mean of the group sizes is used. Type I error levels are not guaranteed.

d1önöğrenmestratejileritop

	grup	N	Subset for alpha = 0.05
			1
Tukey HSD[a,b]	2,00	24	262,4583
	1,00	26	272,2308
	3,00	24	279,5833
	Sig.		,220
Scheffe[a,b]	2,00	24	262,4583
	1,00	26	272,2308
	3,00	24	279,5833
	Sig.		,251

Means for groups in homogeneous subsets are displayed.

a. Uses Harmonic Mean Sample Size = 24,632.

b. The group sizes are unequal. The harmonic mean of the group sizes is used. Type I error levels are not guaranteed.

d1sonişbirliktop

	grup	N	Subset for alpha = 0.05	
			1	2
Tukey HSD[a,b]	3,00	24	58,1250	
	2,00	24	64,2917	
	1,00	26		73,3846
	Sig.		,118	1,000
Scheffe[a,b]	3,00	24	58,1250	
	2,00	24	64,2917	
	1,00	26		73,3846
	Sig.		,141	1,000

Means for groups in homogeneous subsets are displayed.

a. Uses Harmonic Mean Sample Size = 24,632.

b. The group sizes are unequal. The harmonic mean of the group sizes is used. Type I error levels are not guaranteed.

d1sondijitaltektop

	grup	N	Subset for alpha = 0.05		
			1	2	3
Tukey HSD[a,b]	3,00	24	120,1250		
	1,00	26		135,1923	
	2,00	24			144,5833
	Sig.		1,000	1,000	1,000
Scheffe[a,b]	3,00	24	120,1250		
	1,00	26		135,1923	
	2,00	24			144,5833
	Sig.		1,000	1,000	1,000

Means for groups in homogeneous subsets are displayed.

a. Uses Harmonic Mean Sample Size = 24,632.

b. The group sizes are unequal. The harmonic mean of the group sizes is used. Type I error levels are not guaranteed.

d1sonöğrstratejitop

	grup	N	Subset for alpha = 0.05	
			1	2
Tukey HSD[a,b]	3,00	24	256,3333	
	2,00	24		280,4583
	1,00	26		297,7308
	Sig.		1,000	,173
Scheffe[a,b]	3,00	24	256,3333	
	2,00	24		280,4583
	1,00	26		297,7308
	Sig.		1,000	,201

Means for groups in homogeneous subsets are displayed.

a. Uses Harmonic Mean Sample Size = 24,632.

b. The group sizes are unequal. The harmonic mean of the group sizes is used. Type I error levels are not guaranteed.

```
ONEWAY d1önişbirlikçitop d1öndijitaltektop d1önöğrenmestratejileritop BY grup
 /STATISTICS DESCRIPTIVES HOMOGENEITY
 /MISSING ANALYSIS.
```

Oneway

Notes

Output Created		03-NOV-2024 21:15:01
Comments		
Input	Data	C: \Users\Gizem\Pictures\NİHAL HOCAYLA ÇALIŞMA 2023\tersyüz-web ana analiz\tersyüz-web ana analiz.sav
	Active Dataset	DataSet1
	Filter	<none>
	Weight	<none>
	Split File	<none>
	N of Rows in Working Data File	74
Missing Value Handling	Definition of Missing	User-defined missing values are treated as missing.
	Cases Used	Statistics for each analysis are based on cases with no missing data for any variable in the analysis.
Syntax		ONEWAY d1önişbirlikçitop d1öndijitaltektop d1önöğrenmestratejileritop BY grup /STATISTICS DESCRIPTIVES HOMOGENEITY /MISSING ANALYSIS.
Resources	Processor Time	00:00:00,00
	Elapsed Time	00:00:00,00

```
[DataSet1] C:\Users\Gizem\Pictures\NİHAL HOCAYLA ÇALIŞMA 2023\tersyüz-web
ana analiz\tersyüz-web ana analiz.sav
```

Descriptives

		N	Mean	Std. Deviation	Std. Error
d1önişbirlikçitop	1,00	26	64,4615	7,13992	1,40025
	2,00	24	61,7083	9,42928	1,92474
	3,00	24	59,2917	7,54971	1,54108
	Total	74	61,8919	8,24715	,95871
d1öndijitaltektop	1,00	26	129,3846	13,84797	2,71581
	2,00	24	129,5000	8,20392	1,67462
	3,00	24	123,4583	8,88075	1,81278
	Total	74	127,5000	10,93975	1,27172
d1önöğrenmestratejilerito p	1,00	26	272,2308	36,13564	7,08678
	2,00	24	262,4583	24,16426	4,93251
	3,00	24	279,5833	44,14592	9,01125
	Total	74	271,4459	35,96846	4,18125

Descriptives

		95% Confidence Interval for Mean		Minimum	Maximum
		Lower Bound	Upper Bound		
d1önişbirlikçitop	1,00	61,5777	67,3454	51,00	77,00
	2,00	57,7267	65,6900	36,00	75,00
	3,00	56,1037	62,4796	46,00	76,00
	Total	59,9812	63,8026	36,00	77,00
d1öndijitaltektop	1,00	123,7913	134,9779	94,00	152,00
	2,00	126,0358	132,9642	116,00	149,00
	3,00	119,7083	127,2083	111,00	148,00
	Total	124,9655	130,0345	94,00	152,00
d1önöğrenmestratejilerito p	1,00	257,6353	286,8263	205,00	348,00
	2,00	252,2547	272,6620	202,00	303,00
	3,00	260,9421	298,2245	114,00	342,00
	Total	263,1127	279,7792	114,00	348,00

Test of Homogeneity of Variances

	Levene Statistic	df1	df2	Sig.
d1önişbirlikçitop	,675	2	71	,513
d1öndijitaltektop	3,938	2	71	,024
d1önöğrenmestratejilerito p	1,041	2	71	,358

ANOVA

		Sum of Squares	df	Mean Square	F
d1önişbirlikçitop	Between Groups	334,757	2	167,378	2,567
	Within Groups	4630,378	71	65,217	
	Total	4965,135	73		
d1öndijitaltektop	Between Groups	580,388	2	290,194	2,526
	Within Groups	8156,112	71	114,875	
	Total	8736,500	73		
d1önöğrenmestratejilerito p	Between Groups	3543,877	2	1771,938	1,384
	Within Groups	90898,407	71	1280,259	
	Total	94442,284	73		

ANOVA

		Sig.
d1önişbirlikçitop	Between Groups	,084
	Within Groups	
	Total	
d1öndijitaltektop	Between Groups	,087
	Within Groups	
	Total	
d1önöğrenmestratejilerito p	Between Groups	,257
	Within Groups	
	Total	

```
ONEWAY d1önişbirlikçitop d1öndijitaltektop d1önöğrenmestratejileritop BY grup
  /STATISTICS DESCRIPTIVES HOMOGENEITY
  /MISSING ANALYSIS
  /POSTHOC=LSD ALPHA(0.05).
```

Oneway

Notes

Output Created		03-NOV-2024 21:16:22
Comments		
Input	Data	C:\Users\Gizem\Pictures\NİHAL HOCAYLA ÇALIŞMA 2023\tersyüz-web ana analiz\tersyüz-web ana analiz.sav
	Active Dataset	DataSet1
	Filter	<none>
	Weight	<none>
	Split File	<none>
	N of Rows in Working Data File	74
Missing Value Handling	Definition of Missing	User-defined missing values are treated as missing.
	Cases Used	Statistics for each analysis are based on cases with no missing data for any variable in the analysis.
Syntax		ONEWAY d1önişbirlikçitop d1öndijitaltektop d1önöğrenmestratejileritop BY grup /STATISTICS DESCRIPTIVES HOMOGENEITY /MISSING ANALYSIS /POSTHOC=LSD ALPHA (0.05).
Resources	Processor Time	00:00:00,00
	Elapsed Time	00:00:00,00

```
[DataSet1] C:\Users\Gizem\Pictures\NİHAL HOCAYLA ÇALIŞMA 2023\tersyüz-web
ana analiz\tersyüz-web ana analiz.sav
```

Descriptives

		N	Mean	Std. Deviation	Std. Error
d1önişbirlikçitop	1,00	26	64,4615	7,13992	1,40025
	2,00	24	61,7083	9,42928	1,92474
	3,00	24	59,2917	7,54971	1,54108
	Total	74	61,8919	8,24715	,95871
d1öndijitaltektop	1,00	26	129,3846	13,84797	2,71581
	2,00	24	129,5000	8,20392	1,67462
	3,00	24	123,4583	8,88075	1,81278
	Total	74	127,5000	10,93975	1,27172
d1önöğrenmestratejilerito p	1,00	26	272,2308	36,13564	7,08678
	2,00	24	262,4583	24,16426	4,93251
	3,00	24	279,5833	44,14592	9,01125
	Total	74	271,4459	35,96846	4,18125

Descriptives

		95% Confidence Interval for Mean		Minimum	Maximum
		Lower Bound	Upper Bound		
d1önişbirlikçitop	1,00	61,5777	67,3454	51,00	77,00
	2,00	57,7267	65,6900	36,00	75,00
	3,00	56,1037	62,4796	46,00	76,00
	Total	59,9812	63,8026	36,00	77,00
d1öndijitaltektop	1,00	123,7913	134,9779	94,00	152,00
	2,00	126,0358	132,9642	116,00	149,00
	3,00	119,7083	127,2083	111,00	148,00
	Total	124,9655	130,0345	94,00	152,00
d1önöğrenmestratejilerito p	1,00	257,6353	286,8263	205,00	348,00
	2,00	252,2547	272,6620	202,00	303,00
	3,00	260,9421	298,2245	114,00	342,00
	Total	263,1127	279,7792	114,00	348,00

Test of Homogeneity of Variances

	Levene Statistic	df1	df2	Sig.
d1önişbirlikçitop	,675	2	71	,513
d1öndijitaltektop	3,938	2	71	,024
d1önöğrenmestratejilerito p	1,041	2	71	,358

ANOVA

		Sum of Squares	df	Mean Square	F
d1önişbirlikçitop	Between Groups	334,757	2	167,378	2,567
	Within Groups	4630,378	71	65,217	
	Total	4965,135	73		
d1öndijitaltektop	Between Groups	580,388	2	290,194	2,526
	Within Groups	8156,112	71	114,875	
	Total	8736,500	73		
d1önöğrenmestratejileritop	Between Groups	3543,877	2	1771,938	1,384
	Within Groups	90898,407	71	1280,259	
	Total	94442,284	73		

ANOVA

		Sig.
d1önişbirlikçitop	Between Groups	,084
	Within Groups	
	Total	
d1öndijitaltektop	Between Groups	,087
	Within Groups	
	Total	
d1önöğrenmestratejileritop	Between Groups	,257
	Within Groups	
	Total	

Post Hoc Tests

Multiple Comparisons

LSD

Dependent Variable	(I) grup	(J) grup	Mean Difference (I-J)	Std. Error	Sig.
d1önişbirlikçitop	1,00	2,00	2,75321	2,28598	,232
		3,00	5,16987*	2,28598	,027
	2,00	1,00	-2,75321	2,28598	,232
		3,00	2,41667	2,33125	,303
	3,00	1,00	-5,16987*	2,28598	,027
		2,00	-2,41667	2,33125	,303
d1öndijitaltektop	1,00	2,00	-,11538	3,03393	,970
		3,00	5,92628	3,03393	,055
	2,00	1,00	,11538	3,03393	,970
		3,00	6,04167	3,09401	,055
	3,00	1,00	-5,92628	3,03393	,055
		2,00	-6,04167	3,09401	,055
d1önöğrenmestratejileritop	1,00	2,00	9,77244	10,12842	,338
		3,00	-7,35256	10,12842	,470
	2,00	1,00	-9,77244	10,12842	,338
		3,00	-17,12500	10,32900	,102
	3,00	1,00	7,35256	10,12842	,470
		2,00	17,12500	10,32900	,102

Multiple Comparisons

LSD

Dependent Variable	(I) grup	(J) grup	95% Confidence Interval Lower Bound	Upper Bound
d1önişbirlikçitop	1,00	2,00	-1,8049	7,3113
		3,00	,6118	9,7280
	2,00	1,00	-7,3113	1,8049
		3,00	-2,2317	7,0650
	3,00	1,00	-9,7280	-,6118
		2,00	-7,0650	2,2317
d1öndijitaltektop	1,00	2,00	-6,1649	5,9341
		3,00	-,1232	11,9758
	2,00	1,00	-5,9341	6,1649
		3,00	-,1276	12,2109
	3,00	1,00	-11,9758	,1232
		2,00	-12,2109	,1276
d1önöğrenmestratejilerito p	1,00	2,00	-10,4231	29,9679
		3,00	-27,5481	12,8429
	2,00	1,00	-29,9679	10,4231
		3,00	-37,7204	3,4704
	3,00	1,00	-12,8429	27,5481
		2,00	-3,4704	37,7204

*. The mean difference is significant at the 0.05 level.

```
ONEWAY d1sonişbirliktop d1sondijitaltektop d1sonöğrstratejitop BY grup
  /STATISTICS DESCRIPTIVES HOMOGENEITY
  /MISSING ANALYSIS.
```

Oneway

Notes

Output Created		03-NOV-2024 21:17:11
Comments		
Input	Data	C:\Users\Gizem\Pictures\NİHAL HOCAYLA ÇALIŞMA 2023\tersyüz-web ana analiz\tersyüz-web ana analiz.sav
	Active Dataset	DataSet1
	Filter	<none>
	Weight	<none>
	Split File	<none>
	N of Rows in Working Data File	74
Missing Value Handling	Definition of Missing	User-defined missing values are treated as missing.
	Cases Used	Statistics for each analysis are based on cases with no missing data for any variable in the analysis.
Syntax		ONEWAY d1sonişbirliktop d1sondijitaltektop d1sonöğrstratejitop BY grup /STATISTICS DESCRIPTIVES HOMOGENEITY /MISSING ANALYSIS.
Resources	Processor Time	00:00:00,02
	Elapsed Time	00:00:00,02

```
[DataSet1] C:\Users\Gizem\Pictures\NİHAL HOCAYLA ÇALIŞMA 2023\tersyüz-web
ana analiz\tersyüz-web ana analiz.sav
```

Descriptives

		N	Mean	Std. Deviation	Std. Error	95% Confidence ...
						Lower Bound
d1sonişbirliktop	1,00	26	73,3846	11,11783	2,18039	68,8940
	2,00	24	64,2917	13,13965	2,68212	58,7433
	3,00	24	58,1250	7,20092	1,46988	55,0843
	Total	74	65,4865	12,39227	1,44057	62,6154
d1sondijitaltektop	1,00	26	135,1923	11,34203	2,22435	130,6112
	2,00	24	144,5833	14,51211	2,96227	138,4554
	3,00	24	120,1250	8,25839	1,68574	116,6378
	Total	74	133,3514	15,23463	1,77099	129,8218
d1sonöğrstratejitop	1,00	26	297,7308	37,95108	7,44282	282,4020
	2,00	24	280,4583	29,97967	6,11958	267,7990
	3,00	24	256,3333	31,44998	6,41970	243,0532
	Total	74	278,7027	37,18253	4,32238	270,0882

Descriptives

		95% Confidence ...	Minimum	Maximum
		Upper Bound		
d1sonişbirliktop	1,00	77,8752	51,00	94,00
	2,00	69,8401	36,00	86,00
	3,00	61,1657	42,00	72,00
	Total	68,3575	36,00	94,00
d1sondijitaltektop	1,00	139,7735	112,00	156,00
	2,00	150,7113	120,00	184,00
	3,00	123,6122	103,00	134,00
	Total	136,8809	103,00	184,00
d1sonöğrstratejitop	1,00	313,0595	225,00	378,00
	2,00	293,1176	206,00	333,00
	3,00	269,6135	142,00	313,00
	Total	287,3172	142,00	378,00

Test of Homogeneity of Variances

	Levene Statistic	df1	df2	Sig.
d1sonişbirliktop	2,834	2	71	,065
d1sondijitaltektop	1,154	2	71	,321
d1sonöğrstratejitop	1,289	2	71	,282

ANOVA

		Sum of Squares	df	Mean Square	F
d1sonişbirliktop	Between Groups	2956,749	2	1478,375	12,717
	Within Groups	8253,737	71	116,250	
	Total	11210,486	73		
d1sondijitaltektop	Between Groups	7314,368	2	3657,184	26,968
	Within Groups	9628,497	71	135,613	
	Total	16942,865	73		
d1sonöğrstratejitop	Between Groups	21497,052	2	10748,526	9,608
	Within Groups	79428,407	71	1118,710	
	Total	100925,459	73		

ANOVA

		Sig.
d1sonişbirliktop	Between Groups	,000
	Within Groups	
	Total	
d1sondijitaltektop	Between Groups	,000
	Within Groups	
	Total	
d1sonöğrstratejitop	Between Groups	,000
	Within Groups	
	Total	

```
ONEWAY d1sonişbirliktop d1sondijitaltektop d1sonöğrstratejitop BY grup
  /STATISTICS DESCRIPTIVES HOMOGENEITY
  /MISSING ANALYSIS
  /POSTHOC=TUKEY SCHEFFE ALPHA(0.05).
```

Oneway

Notes

Output Created		03-NOV-2024 21:17:45
Comments		
Input	Data	C: \Users\Gizem\Pictures\NİHAL HOCAYLA ÇALIŞMA 2023\tersyüz-web ana analiz\tersyüz-web ana analiz.sav
	Active Dataset	DataSet1
	Filter	<none>
	Weight	<none>
	Split File	<none>
	N of Rows in Working Data File	74
Missing Value Handling	Definition of Missing	User-defined missing values are treated as missing.
	Cases Used	Statistics for each analysis are based on cases with no missing data for any variable in the analysis.
Syntax		ONEWAY d1sonişbirliktop d1sondijitaltektop d1sonöğrstratejitop BY grup<br>/STATISTICS DESCRIPTIVES HOMOGENEITY<br>/MISSING ANALYSIS<br>/POSTHOC=TUKEY SCHEFFE ALPHA(0.05).
Resources	Processor Time	00:00:00,05
	Elapsed Time	00:00:00,05

```
[DataSet1] C:\Users\Gizem\Pictures\NİHAL HOCAYLA ÇALIŞMA 2023\tersyüz-web
ana analiz\tersyüz-web ana analiz.sav
```

Descriptives

		N	Mean	Std. Deviation	Std. Error	95% Confidence ... Lower Bound
d1sonişbirliktop	1,00	26	73,3846	11,11783	2,18039	68,8940
	2,00	24	64,2917	13,13965	2,68212	58,7433
	3,00	24	58,1250	7,20092	1,46988	55,0843
	Total	74	65,4865	12,39227	1,44057	62,6154
d1sondijitaltektop	1,00	26	135,1923	11,34203	2,22435	130,6112
	2,00	24	144,5833	14,51211	2,96227	138,4554
	3,00	24	120,1250	8,25839	1,68574	116,6378
	Total	74	133,3514	15,23463	1,77099	129,8218
d1sonöğrstratejitop	1,00	26	297,7308	37,95108	7,44282	282,4020
	2,00	24	280,4583	29,97967	6,11958	267,7990
	3,00	24	256,3333	31,44998	6,41970	243,0532
	Total	74	278,7027	37,18253	4,32238	270,0882

Descriptives

		95% Confidence ... Upper Bound	Minimum	Maximum
d1sonişbirliktop	1,00	77,8752	51,00	94,00
	2,00	69,8401	36,00	86,00
	3,00	61,1657	42,00	72,00
	Total	68,3575	36,00	94,00
d1sondijitaltektop	1,00	139,7735	112,00	156,00
	2,00	150,7113	120,00	184,00
	3,00	123,6122	103,00	134,00
	Total	136,8809	103,00	184,00
d1sonöğrstratejitop	1,00	313,0595	225,00	378,00
	2,00	293,1176	206,00	333,00
	3,00	269,6135	142,00	313,00
	Total	287,3172	142,00	378,00

Test of Homogeneity of Variances

	Levene Statistic	df1	df2	Sig.
d1sonişbirliktop	2,834	2	71	,065
d1sondijitaltektop	1,154	2	71	,321
d1sonöğrstratejitop	1,289	2	71	,282

ANOVA

		Sum of Squares	df	Mean Square	F
d1sonişbirliktop	Between Groups	2956,749	2	1478,375	12,717
	Within Groups	8253,737	71	116,250	
	Total	11210,486	73		
d1sondijitaltektop	Between Groups	7314,368	2	3657,184	26,968
	Within Groups	9628,497	71	135,613	
	Total	16942,865	73		
d1sonöğrstratejitop	Between Groups	21497,052	2	10748,526	9,608
	Within Groups	79428,407	71	1118,710	
	Total	100925,459	73		

ANOVA

		Sig.
d1sonişbirliktop	Between Groups	,000
	Within Groups	
	Total	
d1sondijitaltektop	Between Groups	,000
	Within Groups	
	Total	
d1sonöğrstratejitop	Between Groups	,000
	Within Groups	
	Total	

Post Hoc Tests

Multiple Comparisons

Dependent Variable		(I) grup	(J) grup	Mean Difference (I-J)	Std. Error	Sig.
d1sonişbirliktop	Tukey HSD	1,00	2,00	9,09295*	3,05203	,011
			3,00	15,25962*	3,05203	,000
		2,00	1,00	-9,09295*	3,05203	,011
			3,00	6,16667	3,11247	,124
		3,00	1,00	-15,25962*	3,05203	,000
			2,00	-6,16667	3,11247	,124
	Scheffe	1,00	2,00	9,09295*	3,05203	,015
			3,00	15,25962*	3,05203	,000
		2,00	1,00	-9,09295*	3,05203	,015
			3,00	6,16667	3,11247	,148
		3,00	1,00	-15,25962*	3,05203	,000
			2,00	-6,16667	3,11247	,148
d1sondijitaltektop	Tukey HSD	1,00	2,00	-9,39103*	3,29642	,016
			3,00	15,06731*	3,29642	,000
		2,00	1,00	9,39103*	3,29642	,016
			3,00	24,45833*	3,36170	,000
		3,00	1,00	-15,06731*	3,29642	,000
			2,00	-24,45833*	3,36170	,000
	Scheffe	1,00	2,00	-9,39103*	3,29642	,021
			3,00	15,06731*	3,29642	,000
		2,00	1,00	9,39103*	3,29642	,021
			3,00	24,45833*	3,36170	,000
		3,00	1,00	-15,06731*	3,29642	,000
			2,00	-24,45833*	3,36170	,000
d1sonöğrstratejitop	Tukey HSD	1,00	2,00	17,27244	9,46785	,169
			3,00	41,39744*	9,46785	,000
		2,00	1,00	-17,27244	9,46785	,169
			3,00	24,12500*	9,65535	,039
		3,00	1,00	-41,39744*	9,46785	,000
			2,00	-24,12500*	9,65535	,039
	Scheffe	1,00	2,00	17,27244	9,46785	,197
			3,00	41,39744*	9,46785	,000
		2,00	1,00	-17,27244	9,46785	,197
			3,00	24,12500	9,65535	,050
		3,00	1,00	-41,39744*	9,46785	,000
			2,00	-24,12500	9,65535	,050

Multiple Comparisons

Dependent Variable		(I) grup	(J) grup	95% Confidence Interval Lower Bound	Upper Bound
d1sonişbirliktop	Tukey HSD	1,00	2,00	1,7869	16,3990
			3,00	7,9536	22,5657
		2,00	1,00	-16,3990	-1,7869
			3,00	-1,2841	13,6174
		3,00	1,00	-22,5657	-7,9536
			2,00	-13,6174	1,2841
	Scheffe	1,00	2,00	1,4619	16,7240
			3,00	7,6286	22,8906
		2,00	1,00	-16,7240	-1,4619
			3,00	-1,6155	13,9488
		3,00	1,00	-22,8906	-7,6286
			2,00	-13,9488	1,6155
d1sondijitaltektop	Tukey HSD	1,00	2,00	-17,2821	-1,4999
			3,00	7,1762	22,9584
		2,00	1,00	1,4999	17,2821
			3,00	16,4110	32,5057
		3,00	1,00	-22,9584	-7,1762
			2,00	-32,5057	-16,4110
	Scheffe	1,00	2,00	-17,6331	-1,1490
			3,00	6,8252	23,3094
		2,00	1,00	1,1490	17,6331
			3,00	16,0530	32,8636
		3,00	1,00	-23,3094	-6,8252
			2,00	-32,8636	-16,0530
d1sonöğrstratejitop	Tukey HSD	1,00	2,00	-5,3920	39,9369
			3,00	18,7330	64,0619
		2,00	1,00	-39,9369	5,3920
			3,00	1,0117	47,2383
		3,00	1,00	-64,0619	-18,7330
			2,00	-47,2383	-1,0117
	Scheffe	1,00	2,00	-6,4001	40,9450
			3,00	17,7249	65,0700
		2,00	1,00	-40,9450	6,4001
			3,00	-,0163	48,2663
		3,00	1,00	-65,0700	-17,7249
			2,00	-48,2663	,0163

*. The mean difference is significant at the 0.05 level.

Homogeneous Subsets

d1sonişbirliktop

	grup	N	Subset for alpha = 0.05	
			1	2
Tukey HSD[a,b]	3,00	24	58,1250	
	2,00	24	64,2917	
	1,00	26		73,3846
	Sig.		,118	1,000
Scheffe[a,b]	3,00	24	58,1250	
	2,00	24	64,2917	
	1,00	26		73,3846
	Sig.		,141	1,000

Means for groups in homogeneous subsets are displayed.

a. Uses Harmonic Mean Sample Size = 24,632.

b. The group sizes are unequal. The harmonic mean of the group sizes is used. Type I error levels are not guaranteed.

d1sondijitaltektop

	grup	N	Subset for alpha = 0.05		
			1	2	3
Tukey HSD[a,b]	3,00	24	120,1250		
	1,00	26		135,1923	
	2,00	24			144,5833
	Sig.		1,000	1,000	1,000
Scheffe[a,b]	3,00	24	120,1250		
	1,00	26		135,1923	
	2,00	24			144,5833
	Sig.		1,000	1,000	1,000

Means for groups in homogeneous subsets are displayed.

a. Uses Harmonic Mean Sample Size = 24,632.

b. The group sizes are unequal. The harmonic mean of the group sizes is used. Type I error levels are not guaranteed.

d1sonöğrstratejitop

	grup	N	Subset for alpha = 0.05	
			1	2
Tukey HSD[a,b]	3,00	24	256,3333	
	2,00	24		280,4583
	1,00	26		297,7308
	Sig.		1,000	,173
Scheffe[a,b]	3,00	24	256,3333	
	2,00	24		280,4583
	1,00	26		297,7308
	Sig.		1,000	,201

Means for groups in homogeneous subsets are displayed.

a. Uses Harmonic Mean Sample Size = 24,632.

b. The group sizes are unequal. The harmonic mean of the group sizes is used. Type I error levels are not guaranteed.

```
ONEWAY d1sonişbirliktop d1sondijitaltektop d1sonöğrstratejitop BY grup
 /STATISTICS DESCRIPTIVES HOMOGENEITY
 /MISSING ANALYSIS.
```

Oneway

Notes

Output Created		03-NOV-2024 23:29:20
Comments		
Input	Data	C: \Users\Gizem\Pictures\NİHAL HOCAYLA ÇALIŞMA 2023\tersyüz-web ana analiz\tersyüz-web ana analiz.sav
	Active Dataset	DataSet1
	Filter	<none>
	Weight	<none>
	Split File	<none>
	N of Rows in Working Data File	74
Missing Value Handling	Definition of Missing	User-defined missing values are treated as missing.
	Cases Used	Statistics for each analysis are based on cases with no missing data for any variable in the analysis.
Syntax		ONEWAY d1sonişbirliktop d1sondijitaltektop d1sonöğrstratejitop BY grup /STATISTICS DESCRIPTIVES HOMOGENEITY /MISSING ANALYSIS.
Resources	Processor Time	00:00:00,00
	Elapsed Time	00:00:00,00

```
[DataSet1] C:\Users\Gizem\Pictures\NİHAL HOCAYLA ÇALIŞMA 2023\tersyüz-web
ana analiz\tersyüz-web ana analiz.sav
```

Descriptives

		N	Mean	Std. Deviation	Std. Error	95% Confidence ...
						Lower Bound
d1sonişbirliktop	1,00	26	73,3846	11,11783	2,18039	68,8940
	2,00	24	64,2917	13,13965	2,68212	58,7433
	3,00	24	58,1250	7,20092	1,46988	55,0843
	Total	74	65,4865	12,39227	1,44057	62,6154
d1sondijitaltektop	1,00	26	135,1923	11,34203	2,22435	130,6112
	2,00	24	144,5833	14,51211	2,96227	138,4554
	3,00	24	120,1250	8,25839	1,68574	116,6378
	Total	74	133,3514	15,23463	1,77099	129,8218
d1sonöğrstratejitop	1,00	26	297,7308	37,95108	7,44282	282,4020
	2,00	24	280,4583	29,97967	6,11958	267,7990
	3,00	24	256,3333	31,44998	6,41970	243,0532
	Total	74	278,7027	37,18253	4,32238	270,0882

Descriptives

		95% Confidence ...	Minimum	Maximum
		Upper Bound		
d1sonişbirliktop	1,00	77,8752	51,00	94,00
	2,00	69,8401	36,00	86,00
	3,00	61,1657	42,00	72,00
	Total	68,3575	36,00	94,00
d1sondijitaltektop	1,00	139,7735	112,00	156,00
	2,00	150,7113	120,00	184,00
	3,00	123,6122	103,00	134,00
	Total	136,8809	103,00	184,00
d1sonöğrstratejitop	1,00	313,0595	225,00	378,00
	2,00	293,1176	206,00	333,00
	3,00	269,6135	142,00	313,00
	Total	287,3172	142,00	378,00

Test of Homogeneity of Variances

	Levene Statistic	df1	df2	Sig.
d1sonişbirliktop	2,834	2	71	,065
d1sondijitaltektop	1,154	2	71	,321
d1sonöğrstratejitop	1,289	2	71	,282

ANOVA

		Sum of Squares	df	Mean Square	F
d1sonişbirliktop	Between Groups	2956,749	2	1478,375	12,717
	Within Groups	8253,737	71	116,250	
	Total	11210,486	73		
d1sondijitaltektop	Between Groups	7314,368	2	3657,184	26,968
	Within Groups	9628,497	71	135,613	
	Total	16942,865	73		
d1sonöğrstratejitop	Between Groups	21497,052	2	10748,526	9,608
	Within Groups	79428,407	71	1118,710	
	Total	100925,459	73		

ANOVA

		Sig.
d1sonişbirliktop	Between Groups	,000
	Within Groups	
	Total	
d1sondijitaltektop	Between Groups	,000
	Within Groups	
	Total	
d1sonöğrstratejitop	Between Groups	,000
	Within Groups	
	Total	

```
ONEWAY d1sonişbirliktop d1sondijitaltektop d1sonöğrstratejitop BY grup
  /STATISTICS DESCRIPTIVES HOMOGENEITY
  /MISSING ANALYSIS
  /POSTHOC=TUKEY ALPHA(0.05).
```

Oneway

Notes

Output Created		03-NOV-2024 23:42:27
Comments		
Input	Data	C:\Users\Gizem\Pictures\NİHAL HOCAYLA ÇALIŞMA 2023\tersyüz-web ana analiz\tersyüz-web ana analiz.sav
	Active Dataset	DataSet1
	Filter	<none>
	Weight	<none>
	Split File	<none>
	N of Rows in Working Data File	74
Missing Value Handling	Definition of Missing	User-defined missing values are treated as missing.
	Cases Used	Statistics for each analysis are based on cases with no missing data for any variable in the analysis.
Syntax		ONEWAY d1sonişbirliktop d1sondijitaltektop d1sonöğrstratejitop BY grup /STATISTICS DESCRIPTIVES HOMOGENEITY /MISSING ANALYSIS /POSTHOC=TUKEY ALPHA(0.05).
Resources	Processor Time	00:00:00,03
	Elapsed Time	00:00:00,03

```
[DataSet1] C:\Users\Gizem\Pictures\NİHAL HOCAYLA ÇALIŞMA 2023\tersyüz-web
ana analiz\tersyüz-web ana analiz.sav
```

Descriptives

		N	Mean	Std. Deviation	Std. Error	95% Confidence ... Lower Bound
d1sonişbirliktop	1,00	26	73,3846	11,11783	2,18039	68,8940
	2,00	24	64,2917	13,13965	2,68212	58,7433
	3,00	24	58,1250	7,20092	1,46988	55,0843
	Total	74	65,4865	12,39227	1,44057	62,6154
d1sondijitaltektop	1,00	26	135,1923	11,34203	2,22435	130,6112
	2,00	24	144,5833	14,51211	2,96227	138,4554
	3,00	24	120,1250	8,25839	1,68574	116,6378
	Total	74	133,3514	15,23463	1,77099	129,8218
d1sonöğrstratejitop	1,00	26	297,7308	37,95108	7,44282	282,4020
	2,00	24	280,4583	29,97967	6,11958	267,7990
	3,00	24	256,3333	31,44998	6,41970	243,0532
	Total	74	278,7027	37,18253	4,32238	270,0882

Descriptives

		95% Confidence ... Upper Bound	Minimum	Maximum
d1sonişbirliktop	1,00	77,8752	51,00	94,00
	2,00	69,8401	36,00	86,00
	3,00	61,1657	42,00	72,00
	Total	68,3575	36,00	94,00
d1sondijitaltektop	1,00	139,7735	112,00	156,00
	2,00	150,7113	120,00	184,00
	3,00	123,6122	103,00	134,00
	Total	136,8809	103,00	184,00
d1sonöğrstratejitop	1,00	313,0595	225,00	378,00
	2,00	293,1176	206,00	333,00
	3,00	269,6135	142,00	313,00
	Total	287,3172	142,00	378,00

Test of Homogeneity of Variances

	Levene Statistic	df1	df2	Sig.
d1sonişbirliktop	2,834	2	71	,065
d1sondijitaltektop	1,154	2	71	,321
d1sonöğrstratejitop	1,289	2	71	,282

ANOVA

		Sum of Squares	df	Mean Square	F
d1sonişbirliktop	Between Groups	2956,749	2	1478,375	12,717
	Within Groups	8253,737	71	116,250	
	Total	11210,486	73		
d1sondijitaltektop	Between Groups	7314,368	2	3657,184	26,968
	Within Groups	9628,497	71	135,613	
	Total	16942,865	73		
d1sonöğrstratejitop	Between Groups	21497,052	2	10748,526	9,608
	Within Groups	79428,407	71	1118,710	
	Total	100925,459	73		

ANOVA

		Sig.
d1sonişbirliktop	Between Groups	,000
	Within Groups	
	Total	
d1sondijitaltektop	Between Groups	,000
	Within Groups	
	Total	
d1sonöğrstratejitop	Between Groups	,000
	Within Groups	
	Total	

Post Hoc Tests

Multiple Comparisons

Tukey HSD

Dependent Variable	(I) grup	(J) grup	Mean Difference (I-J)	Std. Error	Sig.	95% ... Lower Bound
d1sonişbirliktop	1,00	2,00	9,09295*	3,05203	,011	1,7869
		3,00	15,25962*	3,05203	,000	7,9536
	2,00	1,00	-9,09295*	3,05203	,011	-16,3990
		3,00	6,16667	3,11247	,124	-1,2841
	3,00	1,00	-15,25962*	3,05203	,000	-22,5657
		2,00	-6,16667	3,11247	,124	-13,6174
d1sondijitaltektop	1,00	2,00	-9,39103*	3,29642	,016	-17,2821
		3,00	15,06731*	3,29642	,000	7,1762
	2,00	1,00	9,39103*	3,29642	,016	1,4999
		3,00	24,45833*	3,36170	,000	16,4110
	3,00	1,00	-15,06731*	3,29642	,000	-22,9584
		2,00	-24,45833*	3,36170	,000	-32,5057
d1sonöğrstratejitop	1,00	2,00	17,27244	9,46785	,169	-5,3920
		3,00	41,39744*	9,46785	,000	18,7330
	2,00	1,00	-17,27244	9,46785	,169	-39,9369
		3,00	24,12500*	9,65535	,039	1,0117
	3,00	1,00	-41,39744*	9,46785	,000	-64,0619
		2,00	-24,12500*	9,65535	,039	-47,2383

Multiple Comparisons

Tukey HSD

Dependent Variable	(I) grup	(J) grup	95% ... Upper Bound
d1sonişbirliktop	1,00	2,00	16,3990
		3,00	22,5657
	2,00	1,00	-1,7869
		3,00	13,6174
	3,00	1,00	-7,9536
		2,00	1,2841
d1sondijitaltektop	1,00	2,00	-1,4999
		3,00	22,9584
	2,00	1,00	17,2821
		3,00	32,5057
	3,00	1,00	-7,1762
		2,00	-16,4110
d1sonöğrstratejitop	1,00	2,00	39,9369
		3,00	64,0619
	2,00	1,00	5,3920
		3,00	47,2383
	3,00	1,00	-18,7330
		2,00	-1,0117

*. The mean difference is significant at the 0.05 level.

Homogeneous Subsets

d1sonişbirliktop

Tukey HSD[a,b]

grup	N	Subset for alpha = 0.05	
		1	2
3,00	24	58,1250	
2,00	24	64,2917	
1,00	26		73,3846
Sig.		,118	1,000

Means for groups in homogeneous subsets are displayed.

a. Uses Harmonic Mean Sample Size = 24,632.

b. The group sizes are unequal. The harmonic mean of the group sizes is used. Type I error levels are not guaranteed.

d1sondijitaltektop

Tukey HSD[a,b]

grup	N	Subset for alpha = 0.05		
		1	2	3
3,00	24	120,1250		
1,00	26		135,1923	
2,00	24			144,5833
Sig.		1,000	1,000	1,000

Means for groups in homogeneous subsets are displayed.

a. Uses Harmonic Mean Sample Size = 24,632.

b. The group sizes are unequal. The harmonic mean of the group sizes is used. Type I error levels are not guaranteed.

d1sonöğrstratejitop

Tukey HSD[a,b]

grup	N	Subset for alpha = 0.05	
		1	2
3,00	24	256,3333	
2,00	24		280,4583
1,00	26		297,7308
Sig.		1,000	,173

Means for groups in homogeneous subsets are displayed.

a. Uses Harmonic Mean Sample Size = 24,632.

b. The group sizes are unequal. The harmonic mean of the group sizes is used. Type I error levels are not guaranteed.

```
ONEWAY d1sonişbirliktop d1sondijitaltektop d1sonöğrstratejitop BY grup
  /STATISTICS DESCRIPTIVES HOMOGENEITY
  /MISSING ANALYSIS.
```

Oneway

Notes

Output Created		03-NOV-2024 23:50:01
Comments		
Input	Data	C:\Users\Gizem\Pictures\NİHAL HOCAYLA ÇALIŞMA 2023\tersyüz-web ana analiz\tersyüz-web ana analiz.sav
	Active Dataset	DataSet1
	Filter	<none>
	Weight	<none>
	Split File	<none>
	N of Rows in Working Data File	74
Missing Value Handling	Definition of Missing	User-defined missing values are treated as missing.
	Cases Used	Statistics for each analysis are based on cases with no missing data for any variable in the analysis.
Syntax		ONEWAY d1sonişbirliktop d1sondijitaltektop d1sonöğrstratejitop BY grup /STATISTICS DESCRIPTIVES HOMOGENEITY /MISSING ANALYSIS.
Resources	Processor Time	00:00:00,00
	Elapsed Time	00:00:00,00

```
[DataSet1] C:\Users\Gizem\Pictures\NİHAL HOCAYLA ÇALIŞMA 2023\tersyüz-web
ana analiz\tersyüz-web ana analiz.sav
```

Descriptives

		N	Mean	Std. Deviation	Std. Error	95% Confidence ...
						Lower Bound
d1sonişbirliktop	1,00	26	73,3846	11,11783	2,18039	68,8940
	2,00	24	64,2917	13,13965	2,68212	58,7433
	3,00	24	58,1250	7,20092	1,46988	55,0843
	Total	74	65,4865	12,39227	1,44057	62,6154
d1sondijitaltektop	1,00	26	135,1923	11,34203	2,22435	130,6112
	2,00	24	144,5833	14,51211	2,96227	138,4554
	3,00	24	120,1250	8,25839	1,68574	116,6378
	Total	74	133,3514	15,23463	1,77099	129,8218
d1sonöğrstratejitop	1,00	26	297,7308	37,95108	7,44282	282,4020
	2,00	24	280,4583	29,97967	6,11958	267,7990
	3,00	24	256,3333	31,44998	6,41970	243,0532
	Total	74	278,7027	37,18253	4,32238	270,0882

Descriptives

		95% Confidence ...	Minimum	Maximum
		Upper Bound		
d1sonişbirliktop	1,00	77,8752	51,00	94,00
	2,00	69,8401	36,00	86,00
	3,00	61,1657	42,00	72,00
	Total	68,3575	36,00	94,00
d1sondijitaltektop	1,00	139,7735	112,00	156,00
	2,00	150,7113	120,00	184,00
	3,00	123,6122	103,00	134,00
	Total	136,8809	103,00	184,00
d1sonöğrstratejitop	1,00	313,0595	225,00	378,00
	2,00	293,1176	206,00	333,00
	3,00	269,6135	142,00	313,00
	Total	287,3172	142,00	378,00

Test of Homogeneity of Variances

	Levene Statistic	df1	df2	Sig.
d1sonişbirliktop	2,834	2	71	,065
d1sondijitaltektop	1,154	2	71	,321
d1sonöğrstratejitop	1,289	2	71	,282

ANOVA

		Sum of Squares	df	Mean Square	F
d1sonişbirliktop	Between Groups	2956,749	2	1478,375	12,717
	Within Groups	8253,737	71	116,250	
	Total	11210,486	73		
d1sondijitaltektop	Between Groups	7314,368	2	3657,184	26,968
	Within Groups	9628,497	71	135,613	
	Total	16942,865	73		
d1sonöğrstratejitop	Between Groups	21497,052	2	10748,526	9,608
	Within Groups	79428,407	71	1118,710	
	Total	100925,459	73		

ANOVA

		Sig.
d1sonişbirliktop	Between Groups	,000
	Within Groups	
	Total	
d1sondijitaltektop	Between Groups	,000
	Within Groups	
	Total	
d1sonöğrstratejitop	Between Groups	,000
	Within Groups	
	Total	

```
ONEWAY d1sonişbirliktop d1sondijitaltektop d1sonöğrstratejitop BY grup
  /STATISTICS DESCRIPTIVES HOMOGENEITY
  /MISSING ANALYSIS
  /POSTHOC=TUKEY ALPHA(0.05).
```

Oneway

Notes

Output Created		03-NOV-2024 23:50:10
Comments		
Input	Data	C:\Users\Gizem\Pictures\NİHAL HOCAYLA ÇALIŞMA 2023\tersyüz-web ana analiz\tersyüz-web ana analiz.sav
	Active Dataset	DataSet1
	Filter	<none>
	Weight	<none>
	Split File	<none>
	N of Rows in Working Data File	74
Missing Value Handling	Definition of Missing	User-defined missing values are treated as missing.
	Cases Used	Statistics for each analysis are based on cases with no missing data for any variable in the analysis.
Syntax		ONEWAY d1sonişbirliktop d1sondijitaltektop d1sonöğrstratejitop BY grup /STATISTICS DESCRIPTIVES HOMOGENEITY /MISSING ANALYSIS /POSTHOC=TUKEY ALPHA(0.05).
Resources	Processor Time	00:00:00,05
	Elapsed Time	00:00:00,05

```
[DataSet1] C:\Users\Gizem\Pictures\NİHAL HOCAYLA ÇALIŞMA 2023\tersyüz-web
ana analiz\tersyüz-web ana analiz.sav
```

Descriptives

		N	Mean	Std. Deviation	Std. Error	95% Confidence ...
						Lower Bound
d1sonişbirliktop	1,00	26	73,3846	11,11783	2,18039	68,8940
	2,00	24	64,2917	13,13965	2,68212	58,7433
	3,00	24	58,1250	7,20092	1,46988	55,0843
	Total	74	65,4865	12,39227	1,44057	62,6154
d1sondijitaltektop	1,00	26	135,1923	11,34203	2,22435	130,6112
	2,00	24	144,5833	14,51211	2,96227	138,4554
	3,00	24	120,1250	8,25839	1,68574	116,6378
	Total	74	133,3514	15,23463	1,77099	129,8218
d1sonöğrstratejitop	1,00	26	297,7308	37,95108	7,44282	282,4020
	2,00	24	280,4583	29,97967	6,11958	267,7990
	3,00	24	256,3333	31,44998	6,41970	243,0532
	Total	74	278,7027	37,18253	4,32238	270,0882

Descriptives

		95% Confidence ...	Minimum	Maximum
		Upper Bound		
d1sonişbirliktop	1,00	77,8752	51,00	94,00
	2,00	69,8401	36,00	86,00
	3,00	61,1657	42,00	72,00
	Total	68,3575	36,00	94,00
d1sondijitaltektop	1,00	139,7735	112,00	156,00
	2,00	150,7113	120,00	184,00
	3,00	123,6122	103,00	134,00
	Total	136,8809	103,00	184,00
d1sonöğrstratejitop	1,00	313,0595	225,00	378,00
	2,00	293,1176	206,00	333,00
	3,00	269,6135	142,00	313,00
	Total	287,3172	142,00	378,00

Test of Homogeneity of Variances

	Levene Statistic	df1	df2	Sig.
d1sonişbirliktop	2,834	2	71	,065
d1sondijitaltektop	1,154	2	71	,321
d1sonöğrstratejitop	1,289	2	71	,282

ANOVA

		Sum of Squares	df	Mean Square	F
d1sonişbirliktop	Between Groups	2956,749	2	1478,375	12,717
	Within Groups	8253,737	71	116,250	
	Total	11210,486	73		
d1sondijitaltektop	Between Groups	7314,368	2	3657,184	26,968
	Within Groups	9628,497	71	135,613	
	Total	16942,865	73		
d1sonöğrstratejitop	Between Groups	21497,052	2	10748,526	9,608
	Within Groups	79428,407	71	1118,710	
	Total	100925,459	73		

ANOVA

		Sig.
d1sonişbirliktop	Between Groups	,000
	Within Groups	
	Total	
d1sondijitaltektop	Between Groups	,000
	Within Groups	
	Total	
d1sonöğrstratejitop	Between Groups	,000
	Within Groups	
	Total	

Post Hoc Tests

Multiple Comparisons

Tukey HSD

Dependent Variable	(I) grup	(J) grup	Mean Difference (I-J)	Std. Error	Sig.	95% ... Lower Bound
d1sonişbirliktop	1,00	2,00	9,09295*	3,05203	,011	1,7869
		3,00	15,25962*	3,05203	,000	7,9536
	2,00	1,00	-9,09295*	3,05203	,011	-16,3990
		3,00	6,16667	3,11247	,124	-1,2841
	3,00	1,00	-15,25962*	3,05203	,000	-22,5657
		2,00	-6,16667	3,11247	,124	-13,6174
d1sondijitaltektop	1,00	2,00	-9,39103*	3,29642	,016	-17,2821
		3,00	15,06731*	3,29642	,000	7,1762
	2,00	1,00	9,39103*	3,29642	,016	1,4999
		3,00	24,45833*	3,36170	,000	16,4110
	3,00	1,00	-15,06731*	3,29642	,000	-22,9584
		2,00	-24,45833*	3,36170	,000	-32,5057
d1sonöğrstratejitop	1,00	2,00	17,27244	9,46785	,169	-5,3920
		3,00	41,39744*	9,46785	,000	18,7330
	2,00	1,00	-17,27244	9,46785	,169	-39,9369
		3,00	24,12500*	9,65535	,039	1,0117
	3,00	1,00	-41,39744*	9,46785	,000	-64,0619
		2,00	-24,12500*	9,65535	,039	-47,2383

Multiple Comparisons

Tukey HSD

Dependent Variable	(I) grup	(J) grup	95% ... Upper Bound
d1sonişbirliktop	1,00	2,00	16,3990
		3,00	22,5657
	2,00	1,00	-1,7869
		3,00	13,6174
	3,00	1,00	-7,9536
		2,00	1,2841
d1sondijitaltektop	1,00	2,00	-1,4999
		3,00	22,9584
	2,00	1,00	17,2821
		3,00	32,5057
	3,00	1,00	-7,1762
		2,00	-16,4110
d1sonöğrstratejitop	1,00	2,00	39,9369
		3,00	64,0619
	2,00	1,00	5,3920
		3,00	47,2383
	3,00	1,00	-18,7330
		2,00	-1,0117

*. The mean difference is significant at the 0.05 level.

Homogeneous Subsets

d1sonişbirliktop

Tukey HSD[a,b]

grup	N	Subset for alpha = 0.05 1	2
3,00	24	58,1250	
2,00	24	64,2917	
1,00	26		73,3846
Sig.		,118	1,000

Means for groups in homogeneous subsets are displayed.

a. Uses Harmonic Mean Sample Size = 24,632.

b. The group sizes are unequal. The harmonic mean of the group sizes is used. Type I error levels are not guaranteed.

d1sondijitaltektop

Tukey HSD[a,b]

grup	N	Subset for alpha = 0.05		
		1	2	3
3,00	24	120,1250		
1,00	26		135,1923	
2,00	24			144,5833
Sig.		1,000	1,000	1,000

Means for groups in homogeneous subsets are displayed.

a. Uses Harmonic Mean Sample Size = 24,632.

b. The group sizes are unequal. The harmonic mean of the group sizes is used. Type I error levels are not guaranteed.

d1sonöğrstratejitop

Tukey HSD[a,b]

grup	N	Subset for alpha = 0.05	
		1	2
3,00	24	256,3333	
2,00	24		280,4583
1,00	26		297,7308
Sig.		1,000	,173

Means for groups in homogeneous subsets are displayed.

a. Uses Harmonic Mean Sample Size = 24,632.

b. The group sizes are unequal. The harmonic mean of the group sizes is used. Type I error levels are not guaranteed.

```
ONEWAY d1önişbirlikçitop d1öndijitaltektop d1önöğrenmestratejileritop BY grup
 /STATISTICS DESCRIPTIVES HOMOGENEITY
 /MISSING ANALYSIS.
```

Oneway

Notes

Output Created		03-NOV-2024 23:56:00
Comments		
Input	Data	C: \Users\Gizem\Pictures\NİHAL HOCAYLA ÇALIŞMA 2023\tersyüz-web ana analiz\tersyüz-web ana analiz.sav
	Active Dataset	DataSet1
	Filter	<none>
	Weight	<none>
	Split File	<none>
	N of Rows in Working Data File	74
Missing Value Handling	Definition of Missing	User-defined missing values are treated as missing.
	Cases Used	Statistics for each analysis are based on cases with no missing data for any variable in the analysis.
Syntax		ONEWAY d1önişbirlikçitop d1öndijitaltektop d1önöğrenmestratejileritop BY grup /STATISTICS DESCRIPTIVES HOMOGENEITY /MISSING ANALYSIS.
Resources	Processor Time	00:00:00,00
	Elapsed Time	00:00:00,00

```
[DataSet1] C:\Users\Gizem\Pictures\NİHAL HOCAYLA ÇALIŞMA 2023\tersyüz-web
ana analiz\tersyüz-web ana analiz.sav
```

Descriptives

		N	Mean	Std. Deviation	Std. Error
d1önişbirlikçitop	1,00	26	64,4615	7,13992	1,40025
	2,00	24	61,7083	9,42928	1,92474
	3,00	24	59,2917	7,54971	1,54108
	Total	74	61,8919	8,24715	,95871
d1öndijitaltektop	1,00	26	129,3846	13,84797	2,71581
	2,00	24	129,5000	8,20392	1,67462
	3,00	24	123,4583	8,88075	1,81278
	Total	74	127,5000	10,93975	1,27172
d1önöğrenmestratejileritop	1,00	26	272,2308	36,13564	7,08678
	2,00	24	262,4583	24,16426	4,93251
	3,00	24	279,5833	44,14592	9,01125
	Total	74	271,4459	35,96846	4,18125

Descriptives

		95% Confidence Interval for Mean		Minimum	Maximum
		Lower Bound	Upper Bound		
d1önişbirlikçitop	1,00	61,5777	67,3454	51,00	77,00
	2,00	57,7267	65,6900	36,00	75,00
	3,00	56,1037	62,4796	46,00	76,00
	Total	59,9812	63,8026	36,00	77,00
d1öndijitaltektop	1,00	123,7913	134,9779	94,00	152,00
	2,00	126,0358	132,9642	116,00	149,00
	3,00	119,7083	127,2083	111,00	148,00
	Total	124,9655	130,0345	94,00	152,00
d1önöğrenmestratejileritop	1,00	257,6353	286,8263	205,00	348,00
	2,00	252,2547	272,6620	202,00	303,00
	3,00	260,9421	298,2245	114,00	342,00
	Total	263,1127	279,7792	114,00	348,00

Test of Homogeneity of Variances

	Levene Statistic	df1	df2	Sig.
d1önişbirlikçitop	,675	2	71	,513
d1öndijitaltektop	3,938	2	71	,024
d1önöğrenmestratejileritop	1,041	2	71	,358

ANOVA

		Sum of Squares	df	Mean Square	F
d1önişbirlikçitop	Between Groups	334,757	2	167,378	2,567
	Within Groups	4630,378	71	65,217	
	Total	4965,135	73		
d1öndijitaltektop	Between Groups	580,388	2	290,194	2,526
	Within Groups	8156,112	71	114,875	
	Total	8736,500	73		
d1önöğrenmestratejilerito p	Between Groups	3543,877	2	1771,938	1,384
	Within Groups	90898,407	71	1280,259	
	Total	94442,284	73		

ANOVA

		Sig.
d1önişbirlikçitop	Between Groups	,084
	Within Groups	
	Total	
d1öndijitaltektop	Between Groups	,087
	Within Groups	
	Total	
d1önöğrenmestratejilerito p	Between Groups	,257
	Within Groups	
	Total	

```
GRAPH
  /SCATTERPLOT(BIVAR)=d1önişbirlikçitop WITH d1sonişbirliktop BY grup
  /MISSING=LISTWISE.
```

Graph

Notes

Output Created		04-NOV-2024 00:00:24
Comments		
Input	Data	C:\Users\Gizem\Pictures\NİHAL HOCAYLA ÇALIŞMA 2023\tersyüz-web ana analiz\tersyüz-web ana analiz.sav
	Active Dataset	DataSet1
	Filter	<none>
	Weight	<none>
	Split File	<none>
	N of Rows in Working Data File	74
Syntax		GRAPH /SCATTERPLOT(BIVAR)=d1önişbirlikçitop WITH d1sonişbirliktop BY grup /MISSING=LISTWISE.
Resources	Processor Time	00:00:01,91
	Elapsed Time	00:00:01,36

```
[DataSet1] C:\Users\Gizem\Pictures\NİHAL HOCAYLA ÇALIŞMA 2023\tersyüz-web
ana analiz\tersyüz-web ana analiz.sav
```

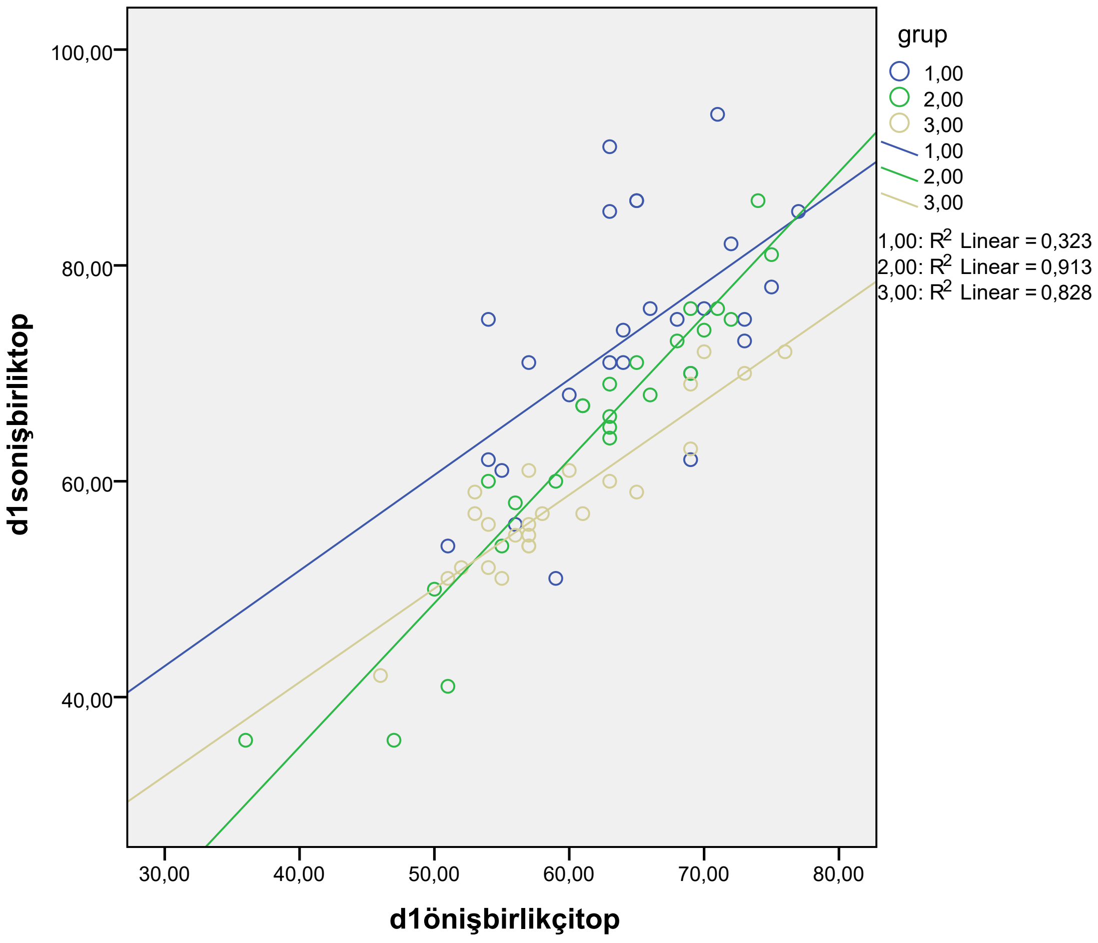


```
GRAPH
  /SCATTERPLOT(BIVAR)=d1öndijitaltektop WITH d1sondijitaltektop BY grup
  /MISSING=LISTWISE.
```

Graph

Notes

Output Created		04-NOV-2024 00:03:37
Comments		
Input	Data	C: \Users\Gizem\Pictures\NİHAL HOCAYLA ÇALIŞMA 2023\tersyüz-web ana analiz\tersyüz-web ana analiz.sav
	Active Dataset	DataSet1
	Filter	<none>
	Weight	<none>
	Split File	<none>
	N of Rows in Working Data File	74
Syntax		GRAPH /SCATTERPLOT(BIVAR) =d1öndijitaltektop WITH d1sondijitaltektop BY grup /MISSING=LISTWISE.
Resources	Processor Time	00:00:00,25
	Elapsed Time	00:00:00,22

```
[DataSet1] C:\Users\Gizem\Pictures\NİHAL HOCAYLA ÇALIŞMA 2023\tersyüz-web
ana analiz\tersyüz-web ana analiz.sav
```

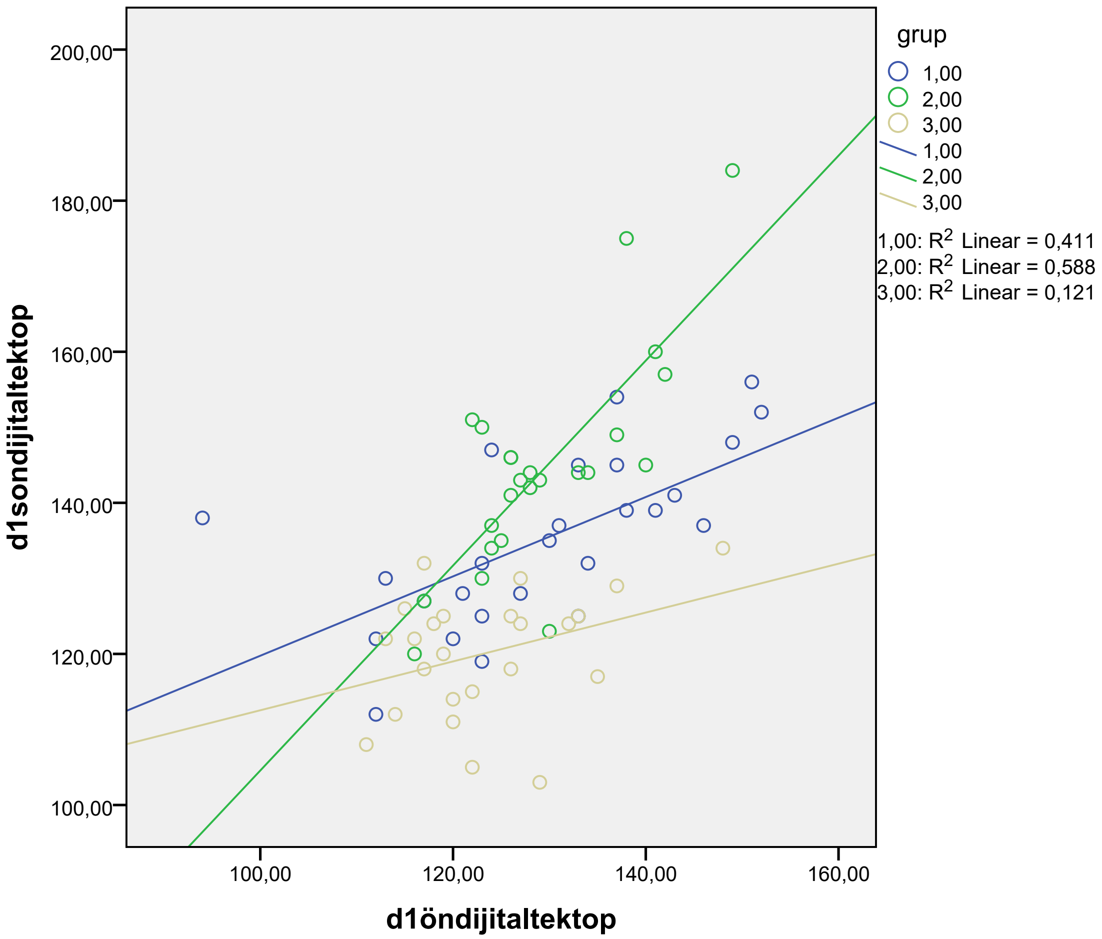


```
GRAPH
 /SCATTERPLOT(BIVAR)=d1önöğrenmestratejileritop WITH d1sonöğrstratejitop BY grup
 /MISSING=LISTWISE.
```

Graph

Notes

Output Created		04-NOV-2024 00:04:18
Comments		
Input	Data	C: \Users\Gizem\Pictures\NİHAL HOCAYLA ÇALIŞMA 2023\tersyüz-web ana analiz\tersyüz-web ana analiz.sav
	Active Dataset	DataSet1
	Filter	<none>
	Weight	<none>
	Split File	<none>
	N of Rows in Working Data File	74
Syntax		GRAPH /SCATTERPLOT(BIVAR) =d1önöğrenmestratejileritop WITH d1sonöğrstratejitop BY grup /MISSING=LISTWISE.
Resources	Processor Time	00:00:00,23
	Elapsed Time	00:00:00,27

```
[DataSet1] C:\Users\Gizem\Pictures\NİHAL HOCAYLA ÇALIŞMA 2023\tersyüz-web
ana analiz\tersyüz-web ana analiz.sav
```

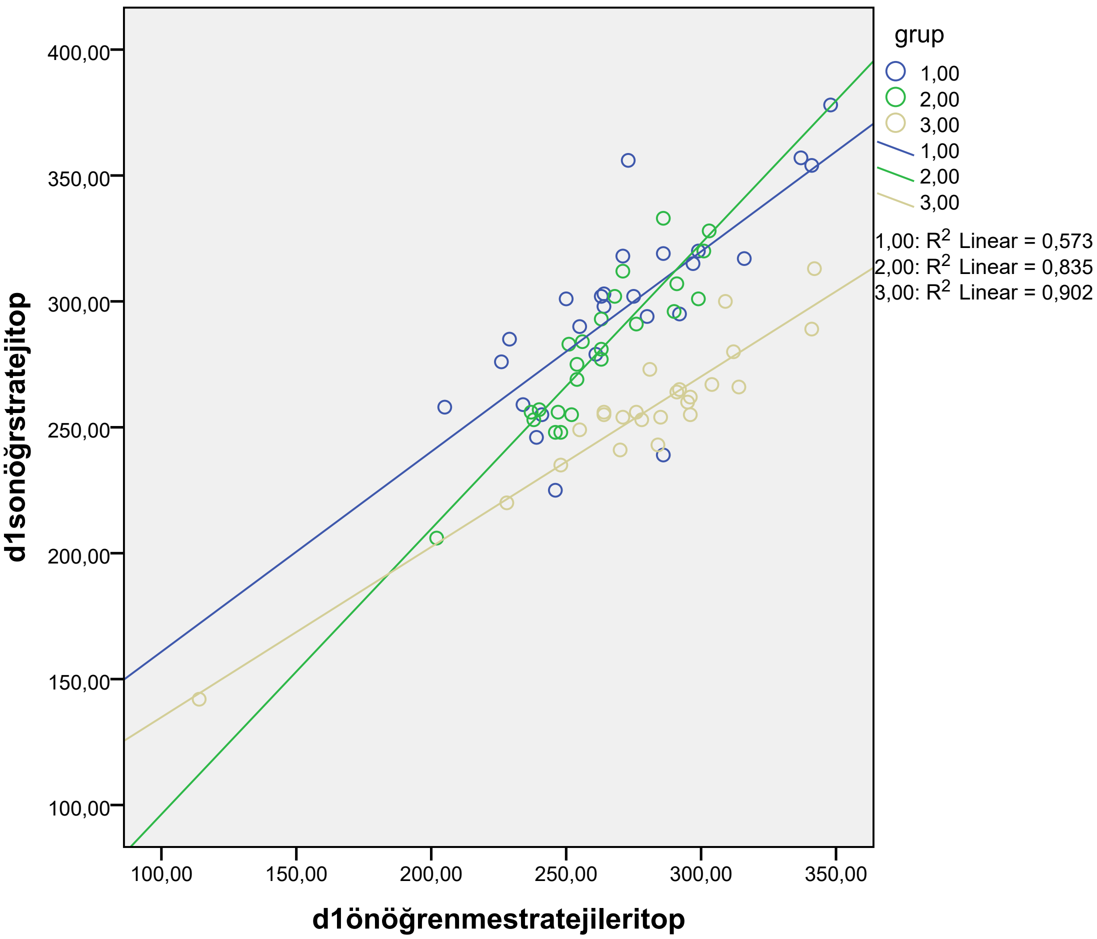


```
UNIANOVA d1sonişbirliktop BY grup WITH d1önişbirlikçitop
  /CONTRAST(grup)=Simple(1)
  /METHOD=SSTYPE(3)
  /INTERCEPT=INCLUDE
  /PLOT=PROFILE(grup)
  /EMMEANS=TABLES(grup) WITH(d1önişbirlikçitop=MEAN) COMPARE ADJ(BONFERRONI)
  /PRINT=ETASQ HOMOGENEITY DESCRIPTIVE
  /CRITERIA=ALPHA(.05)
  /DESIGN=d1önişbirlikçitop grup.
```

Univariate Analysis of Variance

Notes

Output Created		04-NOV-2024 00:10:57
Comments		
Input	Data	C:\Users\Gizem\Pictures\NİHAL HOCAYLA ÇALIŞMA 2023\tersyüz-web ana analiz\tersyüz-web ana analiz.sav
	Active Dataset	DataSet1
	Filter	<none>
	Weight	<none>
	Split File	<none>
	N of Rows in Working Data File	74
Missing Value Handling	Definition of Missing	User-defined missing values are treated as missing.
	Cases Used	Statistics are based on all cases with valid data for all variables in the model.
Syntax		UNIANOVA d1sonişbirliktop BY grup WITH d1önişbirlikçitop /CONTRAST(grup)=Simple(1) /METHOD=SSTYPE(3) /INTERCEPT=INCLUDE /PLOT=PROFILE(grup) /EMMEANS=TABLES(grup) WITH(d1önişbirlikçitop=MEAN) COMPARE ADJ(BONFERRONI) /PRINT=ETASQ HOMOGENEITY DESCRIPTIVE /CRITERIA=ALPHA(.05) /DESIGN=d1önişbirlikçitop grup.
Resources	Processor Time	00:00:00,27
	Elapsed Time	00:00:00,24

```
[DataSet1] C:\Users\Gizem\Pictures\NİHAL HOCAYLA ÇALIŞMA 2023\tersyüz-web
ana analiz\tersyüz-web ana analiz.sav
```

Between-Subjects Factors

		N
grup	1,00	26
	2,00	24
	3,00	24

Descriptive Statistics

Dependent Variable: d1sonişbirliktop

grup	Mean	Std. Deviation	N
1,00	73,3846	11,11783	26
2,00	64,2917	13,13965	24
3,00	58,1250	7,20092	24
Total	65,4865	12,39227	74

Levene's Test of Equality of Error Variances[a]

Dependent Variable: d1sonişbirliktop

F	df1	df2	Sig.
8,635	2	71	,000

Tests the null hypothesis that the error variance of the dependent variable is equal across groups.

a. Design: Intercept + d1önişbirlikçitop + grup

Tests of Between-Subjects Effects

Dependent Variable: d1sonişbirliktop

Source	Type III Sum of Squares	df	Mean Square	F	Sig.	Partial Eta Squared
Corrected Model	8333,334[a]	3	2777,778	67,582	,000	,743
Intercept	2,169	1	2,169	,053	,819	,001
d1önişbirlikçitop	5376,585	1	5376,585	130,810	,000	,651
grup	1126,475	2	563,237	13,703	,000	,281
Error	2877,152	70	41,102			
Total	328558,000	74				
Corrected Total	11210,486	73				

a. R Squared = ,743 (Adjusted R Squared = ,732)

Custom Hypothesis Tests

Contrast Results (K Matrix)

grup Simple Contrast[a]			Dependent Variable d1sonişbirliktop
Level 2 vs. Level 1	Contrast Estimate		-6,126
	Hypothesized Value		0
	Difference (Estimate - Hypothesized)		-6,126
	Std. Error		1,833
	Sig.		,001
	95% Confidence Interval for Difference	Lower Bound	-9,782
		Upper Bound	-2,470
Level 3 vs. Level 1	Contrast Estimate		-9,689
	Hypothesized Value		0
	Difference (Estimate - Hypothesized)		-9,689
	Std. Error		1,879
	Sig.		,000
	95% Confidence Interval for Difference	Lower Bound	-13,436
		Upper Bound	-5,941

a. Reference category = 1

Test Results

Dependent Variable: d1sonişbirliktop

Source	Sum of Squares	df	Mean Square	F	Sig.	Partial Eta Squared
Contrast	1126,475	2	563,237	13,703	,000	,281
Error	2877,152	70	41,102			

Estimated Marginal Means

grup

Estimates

Dependent Variable: d1sonişbirliktop

grup	Mean	Std. Error	95% Confidence Interval Lower Bound	Upper Bound
1,00	70,616[a]	1,280	68,062	73,169
2,00	64,489[a]	1,309	61,879	67,100
3,00	60,927[a]	1,331	58,272	63,582

a. Covariates appearing in the model are evaluated at the following values: d1önişbirlikçitop = 61,8919.

Pairwise Comparisons

Dependent Variable: d1sonişbirliktop

(I) grup	(J) grup	Mean Difference (I-J)	Std. Error	Sig.[b]	95% Confidence Interval for Difference[b]	
					Lower Bound	Upper Bound
1,00	2,00	6,126*	1,833	,004	1,630	10,623
	3,00	9,689*	1,879	,000	5,080	14,298
2,00	1,00	-6,126*	1,833	,004	-10,623	-1,630
	3,00	3,563	1,865	,180	-1,011	8,136
3,00	1,00	-9,689*	1,879	,000	-14,298	-5,080
	2,00	-3,563	1,865	,180	-8,136	1,011

Based on estimated marginal means

*. The mean difference is significant at the ,05 level.

b. Adjustment for multiple comparisons: Bonferroni.

Univariate Tests

Dependent Variable: d1sonişbirliktop

	Sum of Squares	df	Mean Square	F	Sig.	Partial Eta Squared
Contrast	1126,475	2	563,237	13,703	,000	,281
Error	2877,152	70	41,102			

The F tests the effect of grup. This test is based on the linearly independent pairwise comparisons among the estimated marginal means.

Profile Plots

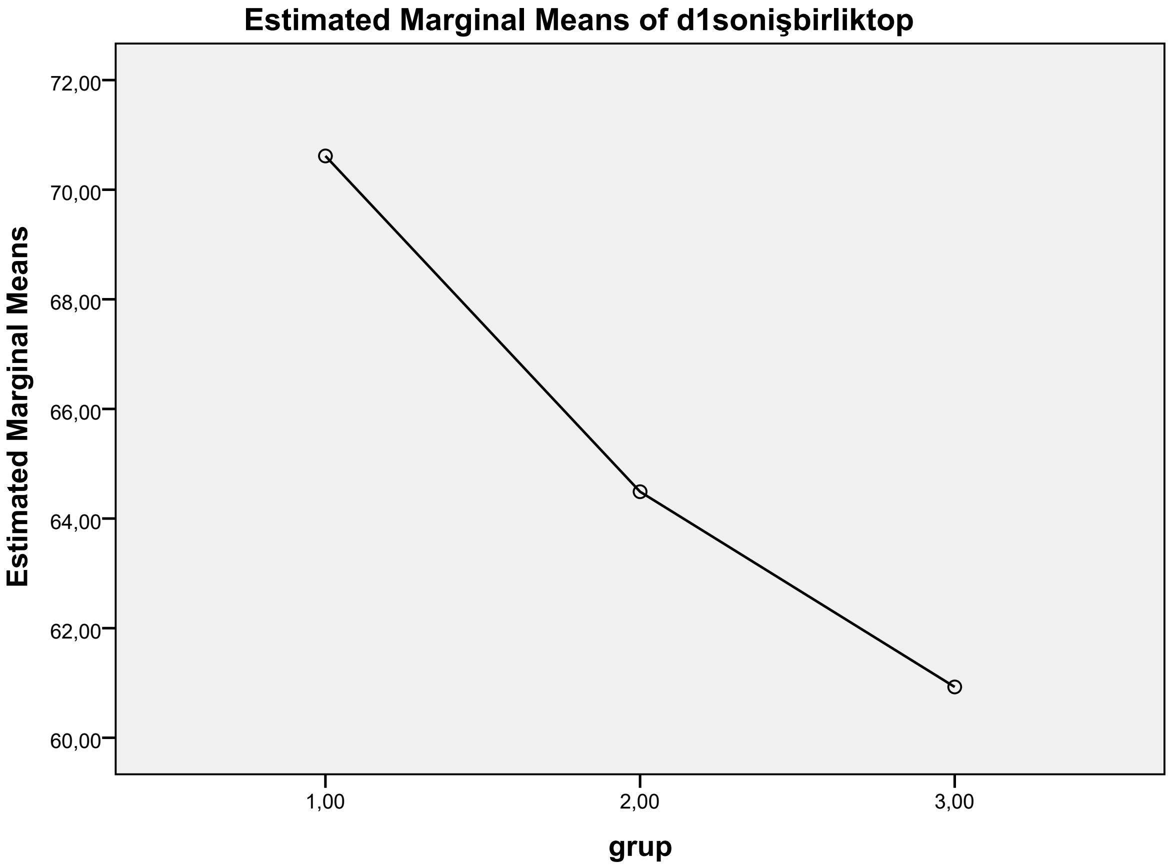

Covariates appearing in the model are evaluated at the following values: d1önişbirlikçitop = 61,8919

```
UNIANOVA d1sondijitaltektop BY grup WITH d1öndijitaltektop
  /CONTRAST(grup)=Simple(1)
  /METHOD=SSTYPE(3)
  /INTERCEPT=INCLUDE
  /PLOT=PROFILE(grup)
  /EMMEANS=TABLES(grup) WITH(d1öndijitaltektop=MEAN) COMPARE ADJ(BONFERRONI)
  /PRINT=ETASQ HOMOGENEITY DESCRIPTIVE
  /CRITERIA=ALPHA(.05)
  /DESIGN=d1öndijitaltektop grup.
```

Univariate Analysis of Variance

Notes

Output Created		04-NOV-2024 00:16:22
Comments		
Input	Data	C:<br>\Users\Gizem\Pictures\NİHAL HOCAYLA ÇALIŞMA 2023\tersyüz-web ana analiz\tersyüz-web ana analiz.sav
	Active Dataset	DataSet1
	Filter	<none>
	Weight	<none>
	Split File	<none>
	N of Rows in Working Data File	74
Missing Value Handling	Definition of Missing	User-defined missing values are treated as missing.
	Cases Used	Statistics are based on all cases with valid data for all variables in the model.
Syntax		UNIANOVA d1sondijitaltektop BY grup WITH d1öndijitaltektop<br>/CONTRAST(grup)=Simple(1)<br>/METHOD=SSTYPE(3)<br>/INTERCEPT=INCLUDE<br>/PLOT=PROFILE(grup)<br>/EMMEANS=TABLES(grup) WITH(d1öndijitaltektop=MEAN) COMPARE ADJ(BONFERRONI)<br>/PRINT=ETASQ HOMOGENEITY DESCRIPTIVE<br>/CRITERIA=ALPHA(.05)<br>/DESIGN=d1öndijitaltektop grup.
Resources	Processor Time	00:00:00,27
	Elapsed Time	00:00:00,25

```
[DataSet1] C:\Users\Gizem\Pictures\NİHAL HOCAYLA ÇALIŞMA 2023\tersyüz-web
ana analiz\tersyüz-web ana analiz.sav
```

Between-Subjects Factors

		N
grup	1,00	26
	2,00	24
	3,00	24

Descriptive Statistics

Dependent Variable: d1sondijitaltektop

grup	Mean	Std. Deviation	N
1,00	135,1923	11,34203	26
2,00	144,5833	14,51211	24
3,00	120,1250	8,25839	24
Total	133,3514	15,23463	74

Levene's Test of Equality of Error Variances[a]

Dependent Variable: d1sondijitaltektop

F	df1	df2	Sig.
,401	2	71	,671

Tests the null hypothesis that the error variance of the dependent variable is equal across groups.

a. Design: Intercept + d1öndijitaltektop + grup

Tests of Between-Subjects Effects

Dependent Variable: d1sondijitaltektop

Source	Type III Sum of Squares	df	Mean Square	F	Sig.	Partial Eta Squared
Corrected Model	10635,671[a]	3	3545,224	39,346	,000	,628
Intercept	1347,122	1	1347,122	14,951	,000	,176
d1öndijitaltektop	3321,303	1	3321,303	36,861	,000	,345
grup	4837,962	2	2418,981	26,847	,000	,434
Error	6307,194	70	90,103			
Total	1332854,000	74				
Corrected Total	16942,865	73				

a. R Squared = ,628 (Adjusted R Squared = ,612)

Custom Hypothesis Tests

Contrast Results (K Matrix)

grup Simple Contrast[a]				Dependent Variable d1sondijitaltektop
Level 2 vs. Level 1	Contrast Estimate			9,317
	Hypothesized Value			0
	Difference (Estimate - Hypothesized)			9,317
	Std. Error			2,687
	Sig.			,001
	95% Confidence Interval for Difference	Lower Bound		3,958
		Upper Bound		14,676
Level 3 vs. Level 1	Contrast Estimate			-11,286
	Hypothesized Value			0
	Difference (Estimate - Hypothesized)			-11,286
	Std. Error			2,758
	Sig.			,000
	95% Confidence Interval for Difference	Lower Bound		-16,787
		Upper Bound		-5,784

a. Reference category = 1

Test Results

Dependent Variable: d1sondijitaltektop

Source	Sum of Squares	df	Mean Square	F	Sig.	Partial Eta Squared
Contrast	4837,962	2	2418,981	26,847	,000	,434
Error	6307,194	70	90,103			

Estimated Marginal Means

grup

Estimates

Dependent Variable: d1sondijitaltektop

grup	Mean	Std. Error	95% Confidence Interval Lower Bound	95% Confidence Interval Upper Bound
1,00	133,990[a]	1,872	130,256	137,723
2,00	143,307[a]	1,949	139,420	147,194
3,00	122,704[a]	1,984	118,748	126,660

a. Covariates appearing in the model are evaluated at the following values: d1öndijitaltektop = 127,5000.

Pairwise Comparisons

Dependent Variable: d1sondijitaltektop

(I) grup	(J) grup	Mean Difference (I-J)	Std. Error	Sig.[b]	95% Confidence Interval for Difference[b]	
					Lower Bound	Upper Bound
1,00	2,00	-9,317*	2,687	,003	-15,908	-2,727
	3,00	11,286*	2,758	,000	4,520	18,051
2,00	1,00	9,317*	2,687	,003	2,727	15,908
	3,00	20,603*	2,813	,000	13,704	27,502
3,00	1,00	-11,286*	2,758	,000	-18,051	-4,520
	2,00	-20,603*	2,813	,000	-27,502	-13,704

Based on estimated marginal means

*. The mean difference is significant at the ,05 level.

b. Adjustment for multiple comparisons: Bonferroni.

Univariate Tests

Dependent Variable: d1sondijitaltektop

	Sum of Squares	df	Mean Square	F	Sig.	Partial Eta Squared
Contrast	4837,962	2	2418,981	26,847	,000	,434
Error	6307,194	70	90,103			

The F tests the effect of grup. This test is based on the linearly independent pairwise comparisons among the estimated marginal means.

Profile Plots

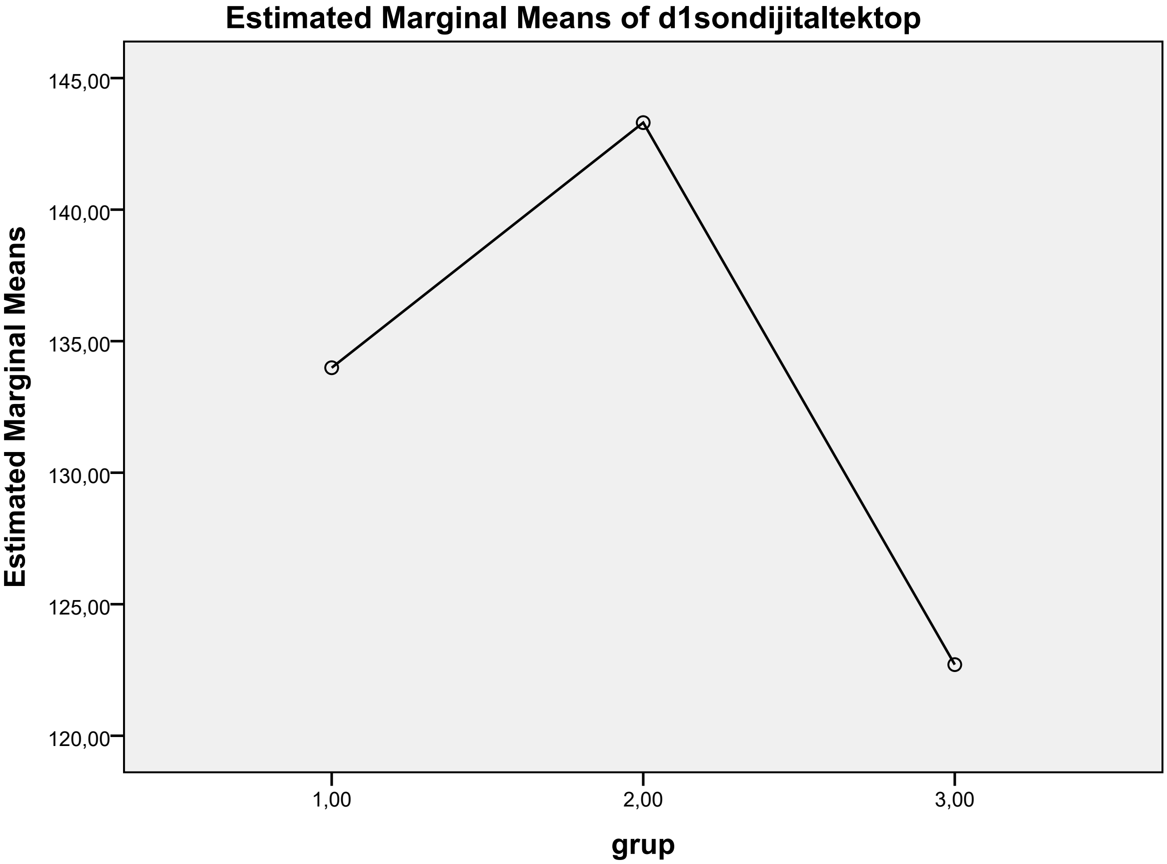

Covariates appearing in the model are evaluated at the following values: d1öndijitaltektop = 127,5000

```
UNIANOVA d1sonöğrstratejitop BY grup WITH d1önöğrenmestratejileritop
  /CONTRAST(grup)=Simple(1)
  /METHOD=SSTYPE(3)
  /INTERCEPT=INCLUDE
  /PLOT=PROFILE(grup)
  /EMMEANS=TABLES(grup) WITH(d1önöğrenmestratejileritop=MEAN) COMPARE ADJ(BONFERRONI)
  /PRINT=ETASQ HOMOGENEITY DESCRIPTIVE
  /CRITERIA=ALPHA(.05)
  /DESIGN=d1önöğrenmestratejileritop grup.
```

Univariate Analysis of Variance

Notes

Output Created		04-NOV-2024 00:17:21
Comments		
Input	Data	C:\Users\Gizem\Pictures\NİHAL HOCAYLA ÇALIŞMA 2023\tersyüz-web ana analiz\tersyüz-web ana analiz.sav
	Active Dataset	DataSet1
	Filter	<none>
	Weight	<none>
	Split File	<none>
	N of Rows in Working Data File	74
Missing Value Handling	Definition of Missing	User-defined missing values are treated as missing.
	Cases Used	Statistics are based on all cases with valid data for all variables in the model.
Syntax		UNIANOVA d1sonöğrstratejitop BY grup WITH d1önöğrenmestratejileritop /CONTRAST(grup)=Simple(1) /METHOD=SSTYPE(3) /INTERCEPT=INCLUDE /PLOT=PROFILE(grup) /EMMEANS=TABLES(grup) WITH(d1önöğrenmestratejileritop=MEAN) COMPARE ADJ(BONFERRONI) /PRINT=ETASQ HOMOGENEITY DESCRIPTIVE /CRITERIA=ALPHA(.05) /DESIGN=d1önöğrenmestratejileritop grup.
Resources	Processor Time	00:00:00,23
	Elapsed Time	00:00:00,23

```
[DataSet1] C:\Users\Gizem\Pictures\NİHAL HOCAYLA ÇALIŞMA 2023\tersyüz-web
ana analiz\tersyüz-web ana analiz.sav
```

Between-Subjects Factors

		N
grup	1,00	26
	2,00	24
	3,00	24

Descriptive Statistics

Dependent Variable: d1sonöğrstratejitop

grup	Mean	Std. Deviation	N
1,00	297,7308	37,95108	26
2,00	280,4583	29,97967	24
3,00	256,3333	31,44998	24
Total	278,7027	37,18253	74

Levene's Test of Equality of Error Variances[a]

Dependent Variable: d1sonöğrstratejitop

F	df1	df2	Sig.
3,506	2	71	,035

Tests the null hypothesis that the error variance of the dependent variable is equal across groups.

a. Design: Intercept + d1önöğrenmestratejileritop + grup

Tests of Between-Subjects Effects

Dependent Variable: d1sonöğrstratejitop

Source	Type III Sum of Squares	df	Mean Square	F	Sig.
Corrected Model	77722,535[a]	3	25907,512	78,159	,000
Intercept	5080,790	1	5080,790	15,328	,000
d1önöğrenmestratejileritop	56225,483	1	56225,483	169,624	,000
grup	30005,077	2	15002,538	45,261	,000
Error	23202,924	70	331,470		
Total	5848890,000	74			
Corrected Total	100925,459	73			

Tests of Between-Subjects Effects

Dependent Variable: d1sonöğrstratejitop

Source	Partial Eta Squared
Corrected Model	,770
Intercept	,180
d1önöğrenmestratejilerito p	,708
grup	,564
Error	
Total	
Corrected Total	

a. R Squared = ,770 (Adjusted R Squared = ,760)

Custom Hypothesis Tests

Contrast Results (K Matrix)

grup Simple Contrast[a]			Dependent Variable d1sonöğrstratejitop
Level 2 vs. Level 1	Contrast Estimate		-9,587
	Hypothesized Value		0
	Difference (Estimate - Hypothesized)		-9,587
	Std. Error		5,187
	Sig.		,069
	95% Confidence Interval for Difference	Lower Bound	-19,932
		Upper Bound	,759
Level 3 vs. Level 1	Contrast Estimate		-47,180
	Hypothesized Value		0
	Difference (Estimate - Hypothesized)		-47,180
	Std. Error		5,173
	Sig.		,000
	95% Confidence Interval for Difference	Lower Bound	-57,497
		Upper Bound	-36,863

a. Reference category = 1

Test Results

Dependent Variable: d1sonöğrstratejitop

Source	Sum of Squares	df	Mean Square	F	Sig.	Partial Eta Squared
Contrast	30005,077	2	15002,538	45,261	,000	,564
Error	23202,924	70	331,470			

Estimated Marginal Means

grup

Estimates

Dependent Variable: d1sonöğrstratejitop

grup	Mean	Std. Error	95% Confidence Interval	
			Lower Bound	Upper Bound
1,00	297,114[a]	3,571	289,992	304,235
2,00	287,527[a]	3,756	280,036	295,018
3,00	249,933[a]	3,749	242,457	257,410

a. Covariates appearing in the model are evaluated at the following values: d1önöğrenmestratejileritop = 271,4459.

Pairwise Comparisons

Dependent Variable: d1sonöğrstratejitop

(I) grup	(J) grup	Mean Difference (I-J)	Std. Error	Sig.[b]	95% Confidence Interval for Difference[b]	
					Lower Bound	Upper Bound
1,00	2,00	9,587	5,187	,206	-3,137	22,310
	3,00	47,180*	5,173	,000	34,492	59,868
2,00	1,00	-9,587	5,187	,206	-22,310	3,137
	3,00	37,593*	5,356	,000	24,455	50,732
3,00	1,00	-47,180*	5,173	,000	-59,868	-34,492
	2,00	-37,593*	5,356	,000	-50,732	-24,455

Based on estimated marginal means

*. The mean difference is significant at the ,05 level.

b. Adjustment for multiple comparisons: Bonferroni.

Univariate Tests

Dependent Variable: d1sonöğrstratejitop

	Sum of Squares	df	Mean Square	F	Sig.	Partial Eta Squared
Contrast	30005,077	2	15002,538	45,261	,000	,564
Error	23202,924	70	331,470			

The F tests the effect of grup. This test is based on the linearly independent pairwise comparisons among the estimated marginal means.

Profile Plots

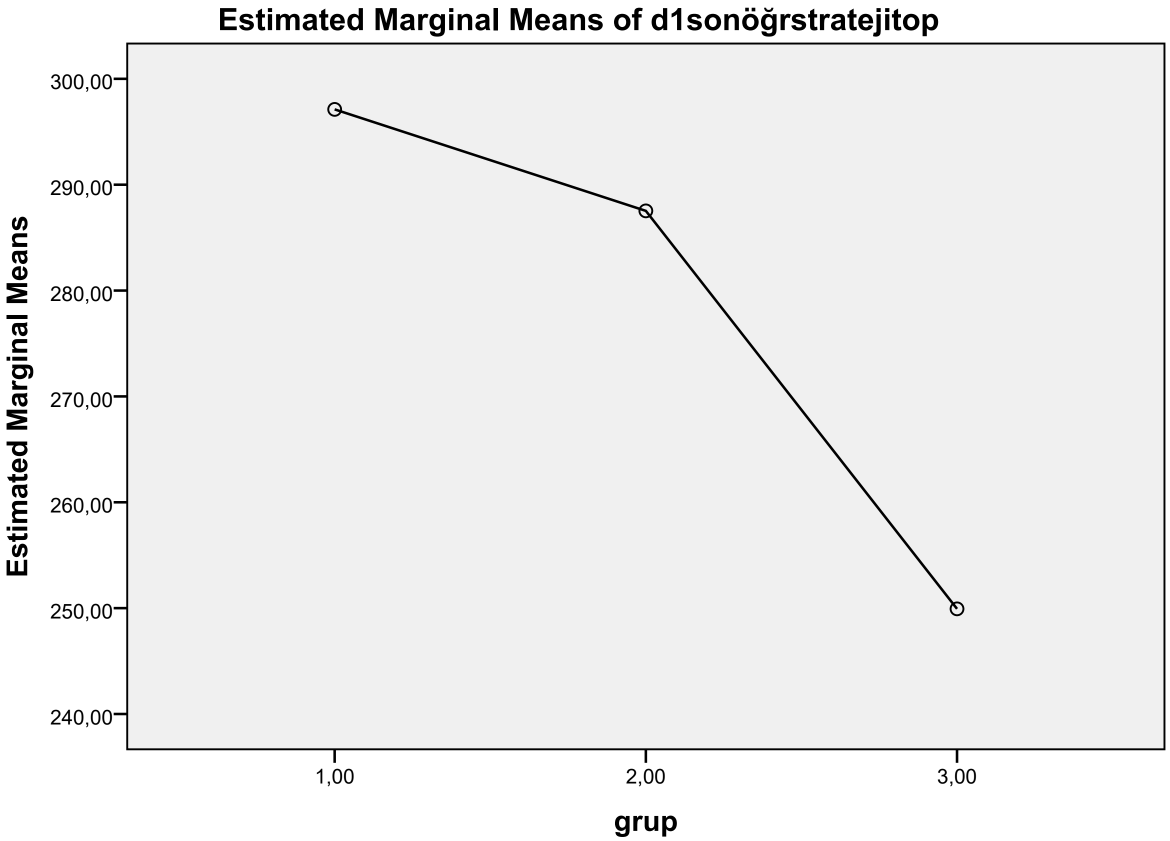

Covariates appearing in the model are evaluated at the following values: d1önöğrenmestratejileritop = 271,4459

```
ONEWAY d1önişbirlikçitop d1önöğrenmestratejileritop BY grup
  /MISSING ANALYSIS.
```

Oneway

Notes

Output Created		19-APR-2026 20:39:00
Comments		
Input	Data	C:\Users\GİZEM TABARU ÖRNEK\Drive'ım\ÇALIŞMALARIM 2025\Flip model ve web aracı makalesi\flip analiz vs\tersyüz-web ana analiz\tersyüz-web ana analiz.sav
	Active Dataset	DataSet1
	Filter	<none>
	Weight	<none>
	Split File	<none>
	N of Rows in Working Data File	74
Missing Value Handling	Definition of Missing	User-defined missing values are treated as missing.
	Cases Used	Statistics for each analysis are based on cases with no missing data for any variable in the analysis.
Syntax		ONEWAY d1önişbirlikçitop d1önöğrenmestratejileritop BY grup /MISSING ANALYSIS.
Resources	Processor Time	00:00:00,02
	Elapsed Time	00:00:00,01

```
[DataSet1] C:\Users\GİZEM TABARU ÖRNEK\Drive'ım\ÇALIŞMALARIM 2025\Flip mod
el ve web aracı makalesi\flip analiz vs\tersyüz-web ana analiz\tersyüz-web
 ana analiz.sav
```

ANOVA

		Sum of Squares	df	Mean Square	F
d1önişbirlikçitop	Between Groups	334,757	2	167,378	2,567
	Within Groups	4630,378	71	65,217	
	Total	4965,135	73		
d1önöğrenmestratejileritop	Between Groups	3543,877	2	1771,938	1,384
	Within Groups	90898,407	71	1280,259	
	Total	94442,284	73		

ANOVA

		Sig.
d1önişbirlikçitop	Between Groups	,084
	Within Groups	
	Total	
d1önöğrenmestratejileritop	Between Groups	,257
	Within Groups	
	Total	

```
UNIANOVA d1sonişbirliktop BY grup WITH d1önişbirlikçitop
  /METHOD=SSTYPE(3)
  /INTERCEPT=INCLUDE
  /CRITERIA=ALPHA(0.05)
  /DESIGN=grup d1önişbirlikçitop.
```

Univariate Analysis of Variance

Notes

Output Created		19-APR-2026 21:07:52
Comments		
Input	Data	C:\Users\GİZEM TABARU ÖRNEK\Drive'ım\ÇALIŞMALARIM 2025\Flip model ve web aracı makalesi\flip analiz vs\tersyüz-web ana analiz\tersyüz-web ana analiz.sav
	Active Dataset	DataSet1
	Filter	<none>
	Weight	<none>
	Split File	<none>
	N of Rows in Working Data File	74
Missing Value Handling	Definition of Missing	User-defined missing values are treated as missing.
	Cases Used	Statistics are based on all cases with valid data for all variables in the model.
Syntax		UNIANOVA d1sonişbirliktop BY grup WITH d1önişbirlikçitop /METHOD=SSTYPE(3) /INTERCEPT=INCLUDE /CRITERIA=ALPHA(0.05) /DESIGN=grup d1önişbirlikçitop.
Resources	Processor Time	00:00:00,00
	Elapsed Time	00:00:00,01

```
[DataSet1] C:\Users\GİZEM TABARU ÖRNEK\Drive'ım\ÇALIŞMALARIM 2025\Flip mod
el ve web aracı makalesi\flip analiz vs\tersyüz-web ana analiz\tersyüz-web
 ana analiz.sav
```

Between-Subjects Factors

		N
grup	1,00	26
	2,00	24
	3,00	24

Tests of Between-Subjects Effects

Dependent Variable: d1sonişbirliktop

Source	Type III Sum of Squares	df	Mean Square	F	Sig.
Corrected Model	8333,334[a]	3	2777,778	67,582	,000
Intercept	2,169	1	2,169	,053	,819
grup	1126,475	2	563,237	13,703	,000
d1önişbirlikçitop	5376,585	1	5376,585	130,810	,000
Error	2877,152	70	41,102		
Total	328558,000	74			
Corrected Total	11210,486	73			

a. R Squared = ,743 (Adjusted R Squared = ,732)

```
UNIANOVA d1sonişbirliktop BY grup WITH d1önişbirlikçitop
  /METHOD=SSTYPE(3)
  /INTERCEPT=INCLUDE
  /CRITERIA=ALPHA(0.05)
  /DESIGN=grup d1önişbirlikçitop d1önişbirlikçitop*grup.
```

Univariate Analysis of Variance

Notes

Output Created		19-APR-2026 21:37:25
Comments		
Input	Data	C:\Users\GİZEM TABARU ÖRNEK\Drive'ım\ÇALIŞMALARIM 2025\Flip model ve web aracı makalesi\flip analiz vs\tersyüz-web ana analiz\tersyüz-web ana analiz.sav
	Active Dataset	DataSet1
	Filter	<none>
	Weight	<none>
	Split File	<none>
	N of Rows in Working Data File	74
Missing Value Handling	Definition of Missing	User-defined missing values are treated as missing.
	Cases Used	Statistics are based on all cases with valid data for all variables in the model.
Syntax		UNIANOVA d1sonişbirliktop BY grup WITH d1önişbirlikçitop /METHOD=SSTYPE(3) /INTERCEPT=INCLUDE /CRITERIA=ALPHA(0.05) /DESIGN=grup d1önişbirlikçitop d1önişbirlikçitop*grup.
Resources	Processor Time	00:00:00,02
	Elapsed Time	00:00:00,02

```
[DataSet1] C:\Users\GİZEM TABARU ÖRNEK\Drive'ım\ÇALIŞMALARIM 2025\Flip mod
el ve web aracı makalesi\flip analiz vs\tersyüz-web ana analiz\tersyüz-web
 ana analiz.sav
```

Between-Subjects Factors

		N
grup	1,00	26
	2,00	24
	3,00	24

Tests of Between-Subjects Effects

Dependent Variable: d1sonişbirliktop

Source	Type III Sum of Squares	df	Mean Square	F	Sig.
Corrected Model	8569,847[a]	5	1713,969	44,137	,000
Intercept	3,260	1	3,260	,084	,773
grup	261,430	2	130,715	3,366	,040
d1önişbirlikçitop	4673,831	1	4673,831	120,357	,000
grup * d1önişbirlikçitop	236,513	2	118,256	3,045	,054
Error	2640,640	68	38,833		
Total	328558,000	74			
Corrected Total	11210,486	73			

a. R Squared = ,764 (Adjusted R Squared = ,747)

```
GRAPH
  /SCATTERPLOT(BIVAR)=d1önişbirlikçitop WITH d1sonişbirliktop
  /MISSING=LISTWISE.
```

Graph

Notes

Output Created		19-APR-2026 21:39:52
Comments		
Input	Data	C:\Users\GİZEM TABARU ÖRNEK\Drive'ım\ÇALIŞMALARIM 2025\Flip model ve web aracı makalesi\flip analiz vs\tersyüz-web ana analiz\tersyüz-web ana analiz.sav
	Active Dataset	DataSet1
	Filter	<none>
	Weight	<none>
	Split File	<none>
	N of Rows in Working Data File	74
Syntax		GRAPH /SCATTERPLOT(BIVAR) =d1önişbirlikçitop WITH d1sonişbirliktop /MISSING=LISTWISE.
Resources	Processor Time	00:00:01,09
	Elapsed Time	00:00:01,22

```
[DataSet1] C:\Users\GİZEM TABARU ÖRNEK\Drive'ım\ÇALIŞMALARIM 2025\Flip mod
el ve web aracı makalesi\flip analiz vs\tersyüz-web ana analiz\tersyüz-web
 ana analiz.sav
```

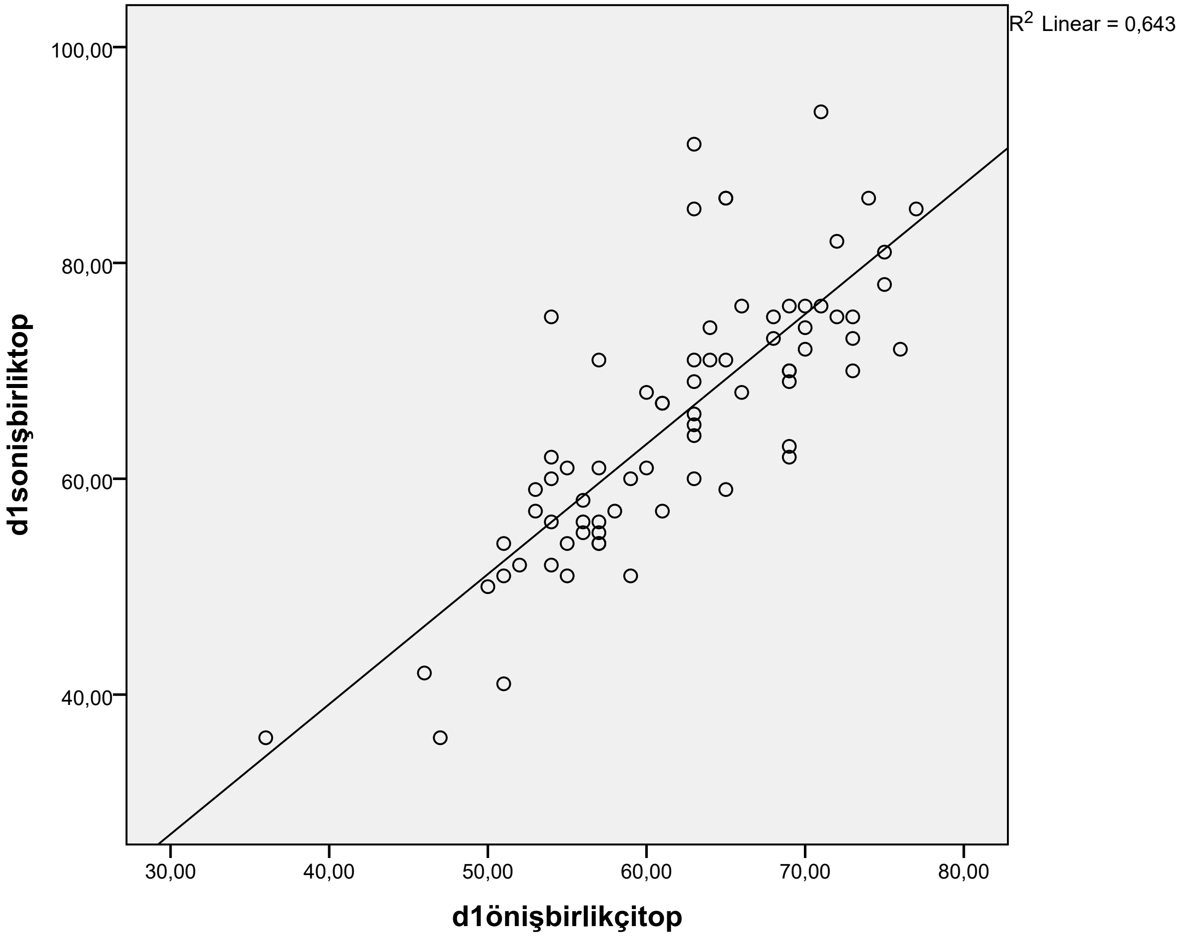


```
UNIANOVA d1sonöğrstratejitop BY grup WITH d1önöğrenmestratejileritop
  /METHOD=SSTYPE(3)
  /INTERCEPT=INCLUDE
  /CRITERIA=ALPHA(0.05)
  /DESIGN=grup d1önöğrenmestratejileritop d1önöğrenmestratejileritop*grup.
```

Univariate Analysis of Variance

Notes

Output Created		19-APR-2026 21:41:25
Comments		
Input	Data	C:\Users\GİZEM TABARU ÖRNEK\Drive'ım\ÇALIŞMALARIM 2025\Flip model ve web aracı makalesi\flip analiz vs\tersyüz-web ana analiz\tersyüz-web ana analiz.sav
	Active Dataset	DataSet1
	Filter	<none>
	Weight	<none>
	Split File	<none>
	N of Rows in Working Data File	74
Missing Value Handling	Definition of Missing	User-defined missing values are treated as missing.
	Cases Used	Statistics are based on all cases with valid data for all variables in the model.
Syntax		UNIANOVA d1sonöğrstratejitop BY grup WITH d1önöğrenmestratejileritop /METHOD=SSTYPE(3) /INTERCEPT=INCLUDE /CRITERIA=ALPHA(0.05) /DESIGN=grup d1önöğrenmestratejileritop d1önöğrenmestratejileritop*grup.
Resources	Processor Time	00:00:00,02
	Elapsed Time	00:00:00,01

```
[DataSet1] C:\Users\GİZEM TABARU ÖRNEK\Drive'ım\ÇALIŞMALARIM 2025\Flip model ve web aracı makalesi\flip analiz vs\tersyüz-web ana analiz\tersyüz-web ana analiz.sav
```

Between-Subjects Factors

		N
grup	1,00	26
	2,00	24
	3,00	24

Tests of Between-Subjects Effects

Dependent Variable: d1sonöğrstratejitop

Source	Type III Sum of Squares	df	Mean Square	F	Sig.
Corrected Model	79884,506[a]	5	15976,901	51,634	,000
Intercept	1867,369	1	1867,369	6,035	,017
grup	1372,550	2	686,275	2,218	,117
d1önöğrenmestratejileritop	53253,864	1	53253,864	172,105	,000
grup * d1önöğrenmestratejileritop	2161,971	2	1080,985	3,494	,036
Error	21040,954	68	309,426		
Total	5848890,000	74			
Corrected Total	100925,459	73			

a. R Squared = ,792 (Adjusted R Squared = ,776)

```
GRAPH
  /SCATTERPLOT(BIVAR)=d1önöğrenmestratejileritop WITH d1sonöğrstratejitop
  /MISSING=LISTWISE.
```

Graph

Notes

Output Created		19-APR-2026 21:42:50
Comments		
Input	Data	C:\Users\GİZEM TABARU ÖRNEK\Drive'ım\ÇALIŞMALARIM 2025\Flip model ve web aracı makalesi\flip analiz vs\tersyüz-web ana analiz\tersyüz-web ana analiz.sav
	Active Dataset	DataSet1
	Filter	<none>
	Weight	<none>
	Split File	<none>
	N of Rows in Working Data File	74
Syntax		GRAPH /SCATTERPLOT(BIVAR) =d1önöğrenmestratejileritop WITH d1sonöğrstratejitop /MISSING=LISTWISE.
Resources	Processor Time	00:00:00,19
	Elapsed Time	00:00:00,19

[DataSet1] C:\Users\GİZEM TABARU ÖRNEK\Drive'ım\ÇALIŞMALARIM 2025\Flip model ve web aracı makalesi\flip analiz vs\tersyüz-web ana analiz\tersyüz-web ana analiz.sav

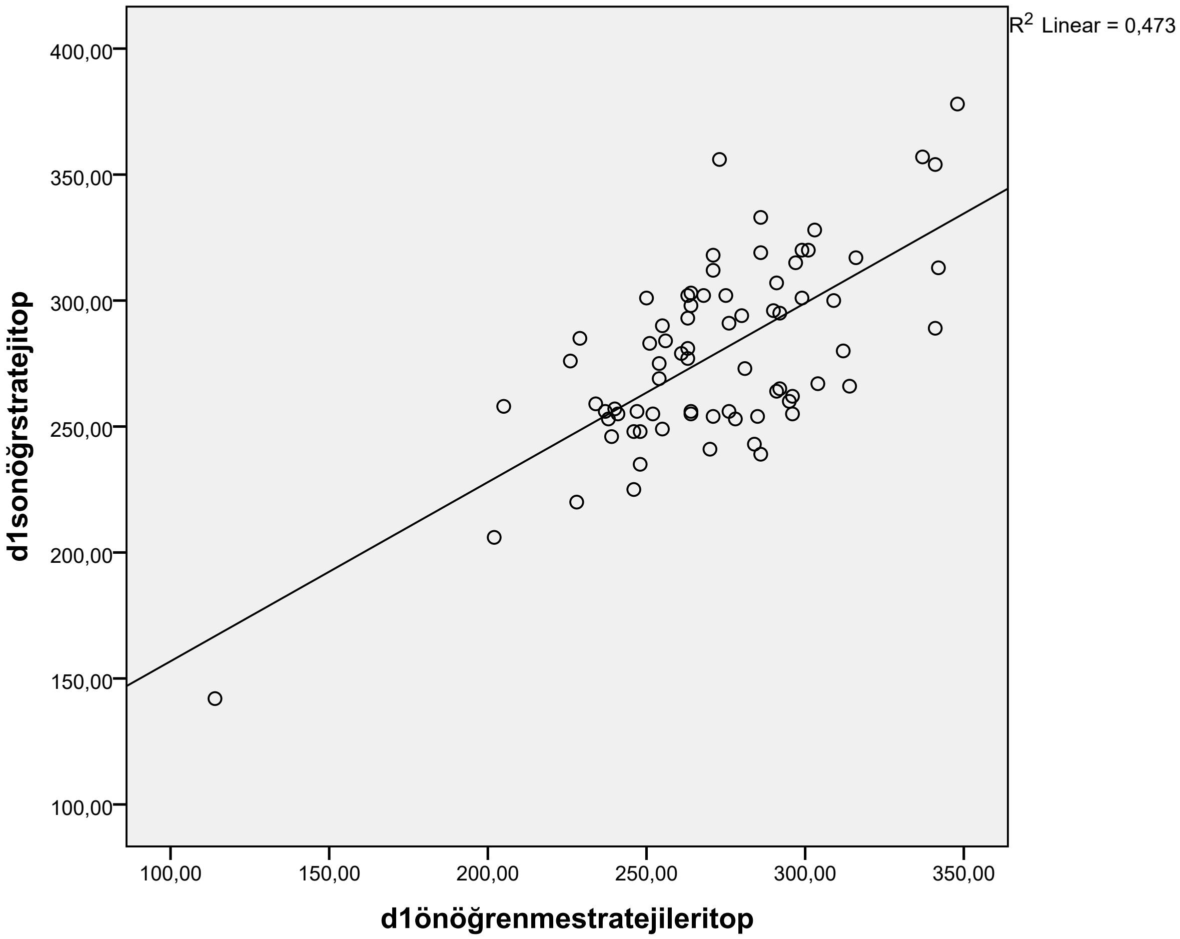

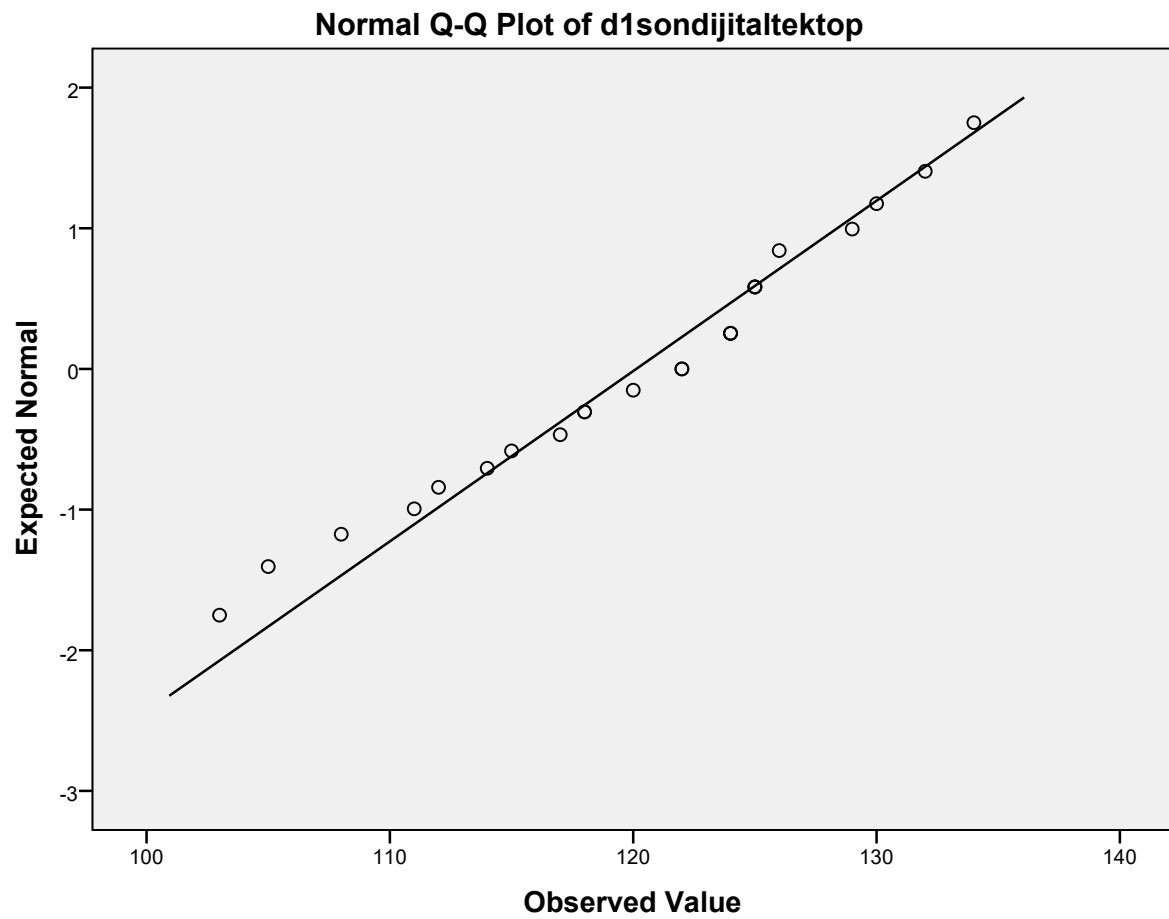

Supplement: Supplementary file 2 [file Data_Sheet_1.pdf]
